# L-type lectin receptor kinases in *Nicotiana benthamiana* and tomato and their role in *Phytophthora* resistance

**DOI:** 10.1093/jxb/erv379

**Published:** 2015-08-05

**Authors:** Yan Wang, Rob Weide, Francine Govers, Klaas Bouwmeester

**Affiliations:** ^1^Laboratory of Phytopathology, Plant Sciences Group, Wageningen University, 6708 PB Wageningen, The Netherlands; ^2^Plant–Microbe Interactions, Department of Biology, Faculty of Science, Utrecht University, 3584 CH Utrecht, The Netherlands

**Keywords:** Immune receptors, LecRKs, phylogenetic analysis, *Phytophthora* pathogens, plant resistance, RLK.

## Abstract

Membrane-bound receptors play crucial roles as sentinels of plant immunity against a large variety of invading microbes. One class of receptors known to be involved in self/non-self-surveillance and plant resistance comprises the L-type lectin receptor kinases (LecRKs). Previously, we reported that several *Arabidopsis* LecRKs play a role in resistance to *Phytophthora* pathogens. In this study, we determined whether homologues of these LecRKs from the Solanaceous plants *Nicotiana benthamiana* and tomato (*Solanum lycopersicum*) play similar roles in defence against *Phytophthora*. In genome-wide screenings, a total of 38 (Nb)LecRKs were identified in *N. benthamiana* and 22 (Sl)LecRKs in tomato, each consisting of both a lectin and a kinase domain. Phylogenetic analysis revealed that, in contrast to *Arabidopsis*, which has a LecRK family comprising nine clades, Solanaceous species have just five of these nine clades (i.e. IV, VI, VII, VIII, and IX), plus four additional clades that lack *Arabidopsis* homologues. Several of the Solanaceous *LecRK*s were selected for functional analysis using virus-induced gene silencing. Infection assays with *Phytophthora capsici* and *Phytophthora infestans* on *LecRK*-silenced plants revealed that *N. benthamiana* and tomato homologues in clade IX play a role in *Phytophthora* resistance similar to the two *Arabidopsis* LecRKs in this clade, suggesting conserved functions of clade IX LecRKs across different plant families. This study provides a first insight into the diversity of Solanaceous LecRKs and their role in plant immunity, and shows the potential of *LecRK*s for *Phytophthora* resistance breeding.

## Highlight

The diversity of L-type lectin receptor kinases (LecRKs) was examined in tomato and *Nicotiana benthamiana* together with their role in resistance to *Phytophthora* pathogens.

## Introduction

Plant diseases caused by *Phytophthora* pathogens are a major constraint to the production of a large variety of Solanaceous crops (Kroon *et al.*, 2012). Renowned are *Phytophthora infestans*, the causal agent of late blight disease on potato and tomato, and *Phytophthora capsici,* which is highly destructive to multiple Solanaceous crops, including tomato, aubergine, and pepper (Fry, 2008; Bouwmeester *et al.*, 2009; Lamour *et al.*, 2012). Breeding for *Phytophthora* resistance has been focused largely on the introgression of resistance (*R*) genes encoding the intracellular nucleotide-binding leucine-rich repeat receptors (NLRs), which mediate effector-triggered immunity (ETI) upon recognition of cognate effectors (Vleeshouwers *et al.*, 2011). Since *Phytophthora* pathogens can quickly adapt, NLR-mediated resistance is often not durable. In the pathogen population, new races emerge that have inactivated or modified effector genes and can thus circumvent *R* gene recognition (Vleeshouwers *et al.*, 2011; Kasuga and Gijzen, 2013).

Besides ETI, plants rely on defence mediated by plasma membrane-localized receptor-like kinases (RLKs), which play pivotal roles in the surveillance of ‘non-self’ (e.g. microbe-associated molecular patterns; MAMPs) and ‘modified self’ (e.g. damage-associated molecular patterns; DAMPs) molecules as exogenous stress signals to initiate non-race-specific immunity. These so-called pattern recognition receptors (PRRs) have been suggested to mediate basal defence, which is thought to have a high potential to confer broad-spectrum disease resistance in plants (Boller and Felix, 2009). So far, PRRs have received limited attention in resistance breeding.

One class of RLKs that has been suggested to function as PRRs in recognition of stress signals and subsequent initiation of plant defence comprises the L-type lectin receptor kinases (LecRKs). *Arabidopsis* has 45 *LecRK* genes that are distributed over nine clades (clades I–IX) and seven singletons, with several showing induced expression upon pathogen attack and in response to pathogen-associated elicitors and MAMPs (Bouwmeester and Govers, 2009). One of these is LecRK-I.9, which functions in maintaining cell wall integrity and plays a crucial role in *Phytophthora* resistance in *Arabidopsis* (Gouget *et al.*, 2006; Bouwmeester *et al.*, 2011). Interfamily gene transfer of *Arabidopsis LecRK-I.9* to the Solanaceous plants *Nicotiana benthamiana* and potato conferred enhanced resistance to *P. infestans* (Bouwmeester *et al.*, 2014), suggesting a conserved functionality in stress signal recognition and immunity among plant species. Recently, it was shown that LecRK-I.9 (also known as DORN1) also functions as a receptor of extracellular ATP (eATP) (Choi *et al.*, 2014). Possibly, eATP is released upon pathogen attack or wounding and as such may function as a DAMP (Choi *et al.*, 2014). Two other *Arabidopsis* LecRKs that have been studied for their roles in plant defence are LecRK-V.5 and LecRK-VI.2. Both were found to play a role in bacterial resistance by mediating stomatal immunity (Desclos-Theveniau *et al.*, 2012; Singh *et al.*, 2012). Ectopic expression of *LecRK-VI.2* in *N. benthamiana* primes MAMP-mediated defence and increases resistance against various hemibiotrophic and necrotrophic bacterial pathogens (Huang and Zimmerli, 2014). Another example is *Arabidopsis* LecRK-I.8, which is required for proper *PR-1* induction upon treatment with egg-derived elicitors of the cabbage butterfly *Pieris brassicae* (Gouhier-Darimont *et al.*, 2013). Recently, we published a study that was aimed at investigating the role of *Arabidopsis* LecRKs in defence against a variety of plant pathogens. This revealed that,next to LecRK-I.9, there are 14 other LecRKs that have a putative role in resistance against *Phytophthora* pathogens in *Arabidopsis* (Wang *et al.*, 2014). *Arabidopsis* lines with T-DNA insertions in these 14 *LecRK* genes showed altered susceptibility when challenged with *P. capsici* and *Phytophthora brassicae*, suggesting that LecRK family members collectively build up a basal level of *Phytophthora* resistance.

Analysis of LecRKs for a potential role in defence in plant species other than *Arabidopsis* has so far been limited. NbLRK1 from *N. benthamiana* was reported to interact with INF1, an elicitin secreted by *P. infestans*, and suggested to play a role in mediating INF1-induced cell death (Kanzaki *et al.*, 2008). In addition, several *LecRK*s were indicated to be involved in plant defence since they are induced upon treatment with pathogens or pathogen-derived elicitors. Multiple cucumber (*Cucumis sativus*) *LecRK*s were found to be induced upon infection by *P. capsici* and *Phytophthora melonis* (Wu *et al.*, 2014), and a cotton (*Gossypium hirsutum*) *LecRK*, *GhLecRK-2*, was shown to be upregulated upon treatment with a cell-wall-derived fraction of the vascular wilt fungus *Verticillium dahliae* (Phillips *et al.*, 2013).

Characterization of defence mechanisms in model plants, such as *Arabidopsis*, paves the way to study similar processes in crops (Koornneef and Meinke, 2010). Disrupting homologous genes encoding proteins with a conserved physiological function often results in similar phenotypes in different plant species. For example, the role of the PRR FLS2 in perception of bacterial flagellin was first discovered in *Arabidopsis* and thereafter found to be largely conserved in various plant lineages (Gomez-Gomez and Boller, 2000; Hann and Rathjen, 2007; Robatzek *et al.*, 2007; Takai *et al.*, 2008).

To determine whether LecRKs are functionally conserved in Solanaceous species, we set out to investigate the function of Solanaceous LecRKs in *Phytophthora* resistance. In this study, we performed a genome-wide identification of LecRKs in *N. benthamiana* and tomato (*Solanum lycopersicum*) and analysed the phylogenetic relationship of these LecRKs with *Arabidopsis* LecRKs. Subsequently, several Solanaceous *LecRK*s were selected for functional analysis using virus-induced gene silencing and infection assays to pinpoint their role in *Phytophthora* resistance.

## Materials and methods

### Sequence identification and gene analysis

Protein sequences of *Arabidopsis* LecRKs analysed by Bouwmeester and Govers (2009) were retrieved from the TAIR website (http://www.arabidopsis.org, last accessed 27 July 2015). Protein sequences were used as queries for reciprocal BLAST searches via the Sol Genomic Network (SGN) website (http://solgenomics.net; last accessed 27 July 2015) against the genomic databases of *N. benthamiana* and tomato. Obtained LecRK sequences were further analysed by comparative analysis using publicly available expressed sequence tags (ESTs), RNA sequencing (RNA-seq) data derived from *Nicotiana benthamiana* Genome Page of the University of Sydney (http://sydney.edu.au/science/molecular_bioscience/sites/benthamiana/, last accessed July 27, 2015; Naim *et al.*, 2012; Nakasugi *et al.*, 2013) and tomato RNA-seq data (L. Faino, personal communication). NbS00026192g0010.1 was verified by sequencing after amplification of the entire cDNA sequence using *Pfu* DNA polymerase (Promega) and gene-specific primers (Supplementary Table S1, available at *JXB* online). All the retrieved cDNA sequences were compared with the genomic DNA sequences, followed by manual validation of the open reading frame and presence of introns (Table 1). Amino acid sequences were subjected to the protein domain and motif annotation webtools SMART (http://smart.embl-heidelberg.de; last accessed July 27, 2015), SignalP 3.0 (http://www.cbs.dtu.dk/services/SignalP-3.0; last accessed 27 July 2015) and TMHMM Server v. 2.0 (http://www.cbs.dtu.dk/services/TMHMM; last accessed 27 July 2015). Predicted kinase domain sequences were aligned by ClustalW and manually checked for subdomains according to those defined based on LecRK-VI.2 and NtCPK5 (Wang *et al.*, 2005; Singh *et al.*, 2013).

**Table 1.** *Overview of LecRKs in* Arabidopsis, *N. benthamiana and tomato*

Clade^*a*^				No. of introns	Protein length (aa)	SP^*b*^	Lectin	TM	Kinase	RD motif^*c*^	Remarks
**I**			AtLecRK-I.1–11								
**II**			AtLecRK-II.1–2								
**III**			AtLecRK-III.1–2								
**IV**			AtLecRK-IV.1–4								
	**IV**		NbS00015931g0001.1	0	666	1–19	20–257	285–307	342–612	+	
			NbS00029393g0102.1	0	669	1–21	22–259	286–308	342–612	+	
			Solyc09g012000.1.1^*d*^	0	669	1–21	22–260	285–307	342–612	+	
			Solyc09g011990.1.1^*d*^	0	668	1–21	23–260	286–308	342–612	+	
			Solyc09g011060.2.1^*e*^	1	832	1–21	22–259	284–306	341–611	+	Contains a OB-NTP-binding domain
**V**			AtLecRK-V.1–9								
**VI**			AtLecRK-VI.1–4								
	**VI**		NbS00010453g0003.1	0	679	1–21	22–265	297–319	351–622	+	Corrected ORF
			NbS00005128g0014.1	0	678	1–20	21–264	296–318	350–621	+	Corrected ORF
			Solyc09g005000.1.1	0	674	1–22	23–262	294–313	345–616	+	
**VII**			AtLecRK-VII.1–2								
	**VII**		NbS00024573g0008.1	0	698	1–26	27–271	303–325	362–632	+	Corrected ORF
			NbS00025337g0001.1	0	695	1–23	24–268	300–322	359–629	+	
			NbS00032834g0007.1	1	678	1–25	26–271	303–325	362–631	+	Corrected ORF
			Solyc02g078170.1.1	0	698	1–25	26–271	303–325	362–632	+	Corrected ORF
**VIII**			LecRK-VIII.1–2								
	**VIII**		NbS00026087g0010.1	0	716	1–29	30–257	325–347	384–657	+	Corrected ORF
			NbS00026192g0010.1	0	716	1–23	25–257	326–348	384–657	+	Corrected ORF; alias NbLRK1
			NbS00008527g0008.1	0	709	1–22	23–249	318–340	377–650	+	Corrected ORF
			NbS00016101g0013.1	0	710	1–18	23–250	320–342	378–651	+	Corrected ORF
			Solyc10g084250.1.1	0	721	1–30	31–257	326–348	386–658	+	
			Solyc09g007510.1.1	0	709	1–20	21–236	313–335	373–645	+	Corrected ORF
**IX**			LecRK-IX.1–2								
	**IX**		NbS00034752g0003.1	2^*f*^	485	1–18	19–196	–	220–441	+	Lacking STK subdomains V, VIa
			NbS00059538g0001.1	0	649	1–26	27–251	269–291	336–605	+	Corrected ORF
			Solyc03g043710.1.1	0	647	1–22	23–249	267–289	333–602	+	
X			AtLecRK-S.1								
	**X**		NbS00001559g0001.1	0	671	1–19	20–264	295–317	353–626	+	
			Solyc03g112310.1.1	0	662	1–21	22–266	296–318	352–622	+	
XI			AtLecRK-S.4								
	**XI**		NbS00015570g0009.1	0	691	1–23	24–263	291–313	345–615	+	Corrected ORF
			NbS00002771g0001.1	0	691	1–23	24–263	291–313	345–615	+	Corrected ORF
			NbS00043874g0006.1	1	424	–	1–24	50–72	106–351	+	Truncated lectin domain; lacking STK subdomains III, IV
			NbS00000505g0005.1	2^*f*^	557	–	7–204	–	267–484	+	Corrected ORF; lacking STK subdomain X
			NbS00056619g0001.1	0	681	1–23	25–263	289–311	345–615	+	Corrected ORF
			NbS00027351g0001.1	0	674	1–16	18–256	282–304	338–608	+	Corrected ORF
			NbS00029393g0011.1	0	681	1–23	25–263	289–311	345–615	+	Corrected ORF
			Solyc05g053010.1.1	0	690	1–23	24–262	287–309	344–614	+	
			Solyc10g084860.1.1	1^*f*^	667	1–20	21–260	286–308	342–593	+	Lacking STK subdomain X
			Solyc09g011070.1.1^*e*^	0	681	1–21	23–261	287–309	343–613	+	
XII			AtLecRK-S.5								
	**XII**		NbS00003611g0313.1	0	679	1–28	29–268	282–304	348–625	+	Corrected ORF
			NbS00005288g0011.1	3^*f*^	540	1–23	24–253	–	309–528	+	Corrected ORF; lacking STK subdomains VIa, X
			Solyc10g080510.1.1	0	670	1–29	30–268	282–304	348–623	+	
XIII			AtLecRK-S.6								
	**XIII**		NbS00006201g0004.1	0	669	1–25	26–238	286–308	348–618	+	Corrected ORF
			NbS00021029g0001.1	0	669	1–25	26–238	286–308	348–618	+	Corrected ORF
			Solyc04g071000.1.1	0	677	1–25	26–241	294–316	356–626	+	
XIV			AtLecRK-S.7								
	**XIV**		NbS00007030g0016.1	0	671	1–22	23–251	291–313	350–622	+	Corrected ORF
			NbS00020348g0007.1	0	671	1–22	23–251	291–313	350–622	+	Corrected ORF
			Solyc07g065610.1.1	0	666	1–20	21–249	289–311	346–618	+	
	**XV**		Solyc03g080060.1.1	0	663	1–16	29–279	317–339	379–626	‒/KN	Corrected ORF; lacking STK subdomain VIII
			NbS00029224g0003.1	0	661	1–20	29–279	317–339	379–626	–/KN	Lacking STK subdomain VIII
			NbS00001395g0006.1	1	662	1–20	29–279	317–339	379–626	–/KN	Lacking STK subdomain VIII
	**XVI**		NbS00007832g0008.1	1	676	1–24	27–276	289–311	350–621	+	
			NbS00001007g0015.1	4^*f*^	454	1–24	30–266	–	323–454	+	Lacking STK subdomains VIa, VII–XI
			NbS00012093g0021.1	1	688	1–24	28–277	291–313	352–623	+	Corrected ORF
			NbS00020337g0016.1	1	688	1–24	29–277	291–313	352–623	+	
			Solyc02g068300.2.1	1	688	1–25	29–278	292–314	353–625	+	
			Solyc03g031980.2.1	1	678	1–20	30–270	289–311	351–614	+	
	**XVII**		NbS00048421g0010.1^*g*^	0	707	1–26	27–261	294–316	362–632	+	Corrected ORF
			NbS00051756g0005.1^*g*^	0	707	1–25	26–263	293–315	361–630	+	Corrected ORF
			Solyc10g047810.1.1^*g*^	0	702	1–19	20–256	288–310	358–627	+	
			Solyc10g047680.1.1^*g,h*^	5^*f*^	520	1–21	42–113	217–239	259–431	+	Truncated lectin domain; lacking STK subdomains III, IV, X, XI
	**XVIII**		NbS00000562g0002.1^*g*^	0	707	1–23	24–257	301–323	367–636	+	
			Solyc01g106160.1.1^*g*^	0	720	1–23	24–256	321–343	387–656	+	
			NbC25369236g0004.1	1^*f*^	362	–	1–162	214–236	279–362	‒	Lacking STK subdomains V–XI
			Solyc10g047700.1.1^*h*^	4^*f*^	351	–	62–106	–	176–281	‒	Truncated lectin domain; lacking STK subdomains VIa–XI
			NbS00037263g0008.1	2^*f*^	655	1–21	22–253	269–291	332–593	+	Corrected ORF

–/KN, ORF, open reading frame; OB-NTP, oligosaccharide/oligonucleotide-binding nucleoside triphosphate; STK, serine/threonine kinase.

^*a*^ Grey shading represents a clade with Solanaceous LecRKs sharing over 50% similarity at the amino acid level. The *Arabidopsis* clades were delineated by Bouwmeester and Govers (2009).

^*b*^ Signal peptide prediction based on SignalP 3.0.

^*c*^ +, present; –, absent; KN, lysine/asparagine substitution.

^*d, e*, *h*^ Tandem duplicated LecRKs.

^*g*^ LecRKs sharing over 50% similarities at the amino acid level.

^*f*^ No RNA-seq data available.

### Multiple sequence alignment and phylogenetic analysis

Protein sequences of full-length LecRKs or either lectin or kinase domains were aligned with ClustalW using the protein weight matrix GONNET with a penalty gap opening of 10 and a gap extension of 0.1. The obtained sequence alignments were subsequently used as input to construct neighbour-joining trees and maximum-likelihood trees with 10 000 bootstrap replicates using the Jones–Taylor–Thornton substitution model (Hall, 2013) in MEGA 5.1. Branches corresponding to partitions reproduced in <60% of bootstrap replicates were collapsed in the phylogenetic trees.

### Plasmid construction

Fragments for gene silencing were chosen to contain stretches of at least 25 nt with 100% identity to the target gene, and at most 20 to off-target genes (Supplementary File S2, available at *JXB* online). Silencing specificity of the gene fragments was verified by BLAST analysis and the virus-induced gene silencing tool at the Sol Genomics Network website (http://vigs.solgenomics.net/; last accessed 27 July 2015). Gene fragments used to generate silencing constructs were synthesized by Eurofins Genomics and subsequently cloned into the gene-silencing vector pTRV-RNA2. pTRV-RNA2 derivatives and pTRV-RNA1 vectors were transformed into *Agrobacterium tumefaciens* strain GV3101.

### Plant growth conditions

*N. benthamiana* and tomato (cultivar Moneymaker) were grown in soil in a conditioned greenhouse at 19–21 °C with a 16h photoperiod and a relative humidity of 75–78%. Supplementary light (100W m^–2^) was applied when the light intensity dropped below 150W m^–2^.

### Agroinfiltration and tobacco rattle virus (TRV)-mediated silencing assays

*A. tumefaciens* strains carrying binary vectors were grown overnight at 28 °C in yeast extract broth with appropriate antibiotics. *A. tumefaciens* cells were pelleted, resuspended, and incubated in induction medium (10mM MES, 10mM MgCl_2_, 50 μM acetosyringone, pH 5.6) for 3–4h and thereafter for 1h in infiltration medium (10mM MES, 10mM MgCl_2_, 200 μM acetosyringone, pH 5.6). For gene silencing, *A. tumefaciens* cultures carrying pTRV-RNA2 constructs and *A. tumefaciens* carrying pTRV1 were mixed at a ratio of 1:1 to a final OD_600_ of 1.0 or 2.0 before infiltration into cotyledons of 2-week-old *N. benthamiana* and 10-d-old tomato seedlings, respectively. TRV:*PDS* and TRV:*GUS* were used as controls. For transient expression of the elicitor genes, *A. tumefaciens* suspensions with appropriate concentrations were syringe infiltrated into *N. benthamiana* leaves. To induce elicitor-triggered cell death, *N. benthamiana* leaves were infiltrated with 100nM INF1.

### RNA isolation and quantitative reverse transcription PCR (qRT-PCR)

For each silencing construct, six leaves (the fifth and sixth true leaves) from three individual plants were harvested and ground in liquid nitrogen. Total RNA was isolated from 100mg of leaf material with a NucleoSpin RNA Plant kit (Macherey-Nagel), and subsequently used as template for first-strand cDNA synthesis using a Moloney murine leukemia virus reverse transcriptase kit (Promega) with an oligo(dT) primer (Supplementary Table S1). qRT-PCR was performed as described by Wang *et al.* (2014) using *Actin* as endogenous control.

### *Phytophthora* cultivation and infection assays

Culturing of *P. capsici* isolate LT263 and *P. infestans* isolate 14-3-GFP and production of zoospores were performed as described previously (Champouret *et al.*, 2009; Wang *et al.*, 2013; Bouwmeester *et al.*, 2014).

*N. benthamiana* and tomato leaves were collected 3–4 weeks after TRV treatment and placed in water-saturated floral foam in trays as described by Vleeshouwers *et al.* (1999). Leaves were inoculated on the abaxial sides with fresh mycelial plugs (diameter 0.5cm), or 10 µl droplets containing 1×10^5^ ml^–1^ *P. capsici* zoospores or 5×10^5^ ml^–1^ *P. infestans* zoospores. Inoculated leaves were kept in transparent plastic boxes to maintain high humidity and placed in a climate chamber with a 12h photoperiod and appropriate temperature settings. Boxes were kept in the dark for the first 24h. The diameters of *P. capsici* lesions were measured at 3 d post-inoculation (dpi) and *P. infestans* lesions at 4 or 6 dpi. Lesion sizes were calculated as described previously (Vleeshouwers *et al.*, 1999).

## Results and discussion

### Identification of LecRKs in N. benthamiana and tomato

**Fig. 1.**
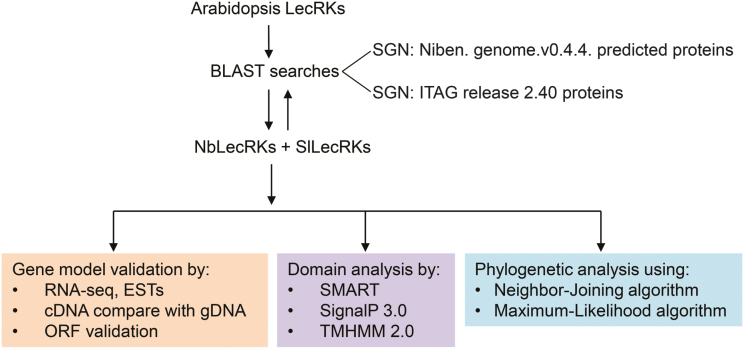
Pipeline depicting the procedures used for identification and analysis of LecRKs in *N. benthamiana* and tomato. (This figure is available in colour at *JXB* online.)

LecRKs in *N. benthamiana* and tomato were identified following the pipeline depicted in Fig. 1. Protein sequences of *Arabidopsis* LecRKs (AtLecRKs) were used as queries for BLAST searches against the predicted protein databases of *N. benthamiana* and tomato via the SGN website. The presence of both a lectin domain and a kinase domain was used as the criterion for the selection of putative LecRKs. Using reciprocal BLAST searches, we identified 37 (Nb)LecRKs and 22 (Sl)LecRKs in *N. benthamiana* and tomato, respectively (Table 1). The predicted cDNA sequences were retrieved and, in combination with EST data, RNA-seq data, and genomic DNA sequences, were used for gene model validation. Strikingly, over half of the *NbLecRK* gene models were found to be incorrect due to erroneous open reading frame prediction and were corrected (Table 1). An additional full-length NbLecRK, NbS00021029g0001.1, that is annotated to lack the kinase domain in the SGN database, was identified by gene model verification using RNA-seq data (Table 1). The coding sequence of the previously described *NbLRK1* (deposited in GenBank as AB247455.1; Kanzaki *et al.*, 2008) was found to contain multiple single-nucleotide polymorphisms when compared with *N. benthamiana* genomic DNA or RNA-seq data. Hence, we PCR amplified and sequenced the coding sequence and found that it matched the corrected gene model of NbS00026192g0010.1. For the *SlLecRK*s, three were found to be erroneous and these were corrected according to the RNA-seq data (Table 1). The obtained LecRK sequences, including those revised, are listed in Supplementary File S1 (available at *JXB* online) and have been deposited in GenBank.

In *Arabidopsis*, only six out of the 45 *LecRK*s contain introns. Five of these have one intron and the other one contains two introns. By comparing Solanaceous *LecRK* cDNA sequences with genomic DNA sequences, 12 *NbLecRK*s were found to contain introns ranging from one to four per gene (Table 1). For six of these, the presence of the intron could be confirmed based on RNA-seq data, but for the other six there was no RNA-seq data available. Three out of the 22 *SlLecRK*s were confirmed to contain one intron based on RNA-seq data, whereas another three *SlLecRK*s were predicted to contain one, four, and five introns, respectively (Table 1).

### Chromosomal location of tomato LecRKs

To investigate tandem duplication events, we examined the chromosomal distribution of the *SlLecRK*s in tomato. For *N. benthamiana*, no such information is available. The 22 *SlLecRK*s were distributed over eight of the 12 tomato chromosomes (chromosomes 1, 2, 3, 4, 5, 7, 9, and 10), with the number of *LecRK*s ranging from one to six per chromosome (Table 1). Most of the *SlLecRK*s were located on chromosomes 9 and 10, with six *SlLecRK*s on each chromosome. In addition, two tandem duplicated *SlLecRK* gene pairs were found on chromosome 9 and one on chromosome 10 based on the criterion that tandem duplicated genes are located within 10 adjacent gene models (Shiu and Bleecker, 2003). In comparison, the *LecRK*s in *Arabidopsis* show a much higher tandem duplication rate; there are nine distinct clusters, each with two to six *AtLecRK*s (Bouwmeester and Govers, 2009).

### Domain composition of NbLecRKs and SlLecRKs

Domain analysis, which was performed using multiple bioinformatics webtools, revealed that, of the 38 identified NbLecRKs, 32 contained the typical composition of a LecRK, i.e. a signal peptide (SP), an extracellular L-type lectin domain, a single transmembrane (TM) domain, and a cytosolic serine/threonine kinase domain. Of the remaining six, three had no clear TM domain, and two had no SP, whereas one lacked both the SP and the TM domain. Most of the 22 SlLecRKs contained all representative LecRK features; the remaining one was predicted to lack both the SP and the TM domain (Table 1). One SlLecRK (Solyc09g011060.2.1) contained an additional domain, namely an oligosaccharide/oligonucleotide-binding domain (Pfam accession: PF07717) at the C terminus adjacent to the kinase domain. Although NbS00043874g0006.1, Solyc10g047680.1.1, and Solyc10g047700.1.1 are predicted to contain a lectin signature, the lectin domain is truncated (Table 1). Kinase domains of RLKs are in general highly conserved and contain 12 subdomains that are essential for kinase activity (Shiu and Bleecker, 2001; Hanks, 2003; Afzal *et al.*, 2008). Alignment of the predicted kinase domains revealed that six NbLecRKs and three SlLecRKs lacked one or more subdomains, which could impair kinase activity (Table 1). Protein kinases that are activated by phosphorylation in the activation loop typically carry a conserved arginine (R) that precedes the catalytic aspartate (D) in subdomain VIb (Nolen *et al.*, 2004; Kornev *et al.*, 2006). They are therefore known as RD kinases, whereas kinases that lack the RD motif are collectively termed non-RD kinases. It has been found that activation of non-RD kinases does not require phosphorylation of the activation loop (Dardick and Ronald, 2006). Nearly all identified Solanaceous LecRKs contained a RD motif with the exception of three NbLecRKs and two SlLecRKs. NbC25369236g0004.1 and Solyc10g047700.1.1 did not contain an RD motif due to absence of the kinase subdomain VIb, whereas in NbS00029224g0003.1, NbS00001395g0006.1, and Solyc03g080060.1.1 RD was substituted by KN (Table 1). This is also the case for some of the *Arabidopsis* LecRKs: AtLecRK-I.2 lacks the kinase subdomain VIb, while AtLecRK-III.1 and AtLecRK-III.2 contain GN residues instead of RD.

### Sequence divergence of *Arabidopsis*, *N. benthamiana*, and tomato LecRKs

Sequence alignment of LecRKs of the three plant species revealed high levels of sequence divergence. At the protein level, the identity of the most similar AtLecRK homologues in *N. benthamiana* and tomato was only 66 and 58%, respectively. At the nucleotide level, the identities between AtLecRKs and SolLecRKs were even lower, at the most 56%. Between LecRK homologues of the two Solanaceous species, the identity was much higher, reaching up to 87% at the protein level and 73% at the nucleotide level.

In *Arabidopsis*, LecRKs are divided into nine clades based on the definition that a clade contains at least two homologues with a minimum of 50% similarity at the nucleotide level (Bouwmeester and Govers, 2009). However, due to high sequence divergence, this criterion is not applicable for the Solanaceous LecRKs (data not shown). Hence, Solanaceous LecRKs were grouped in one clade if they share over 50% similarity at the amino acid level. In this way, 17 clades were found by means of pairwise alignment (Table 1), with three to 10 members per clade.

### Phylogeny of LecRKs in *Arabidopsis*, *N. benthamiana*, and tomato

**Fig. 2.**
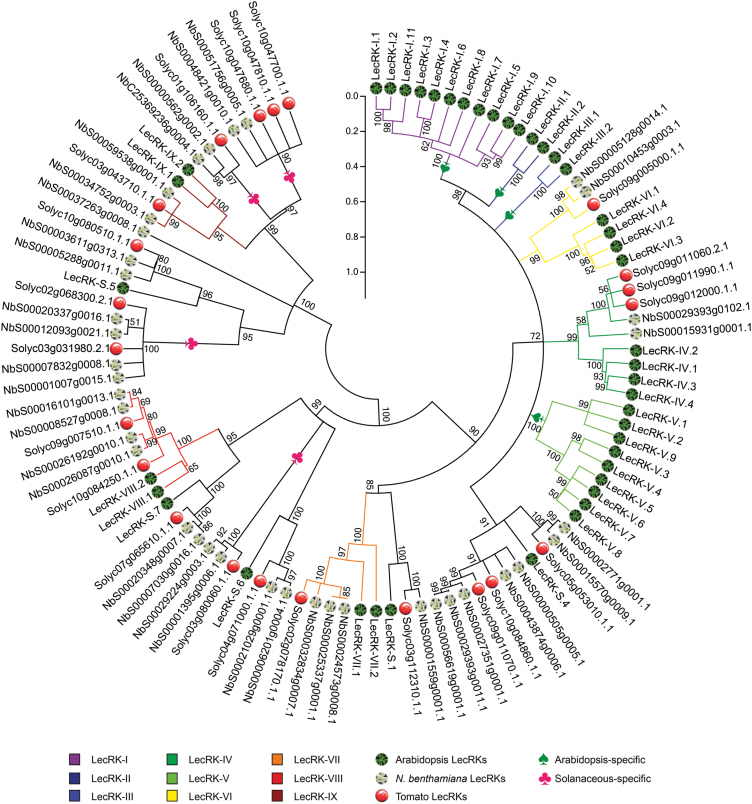
Phylogenetic tree of LecRKs from *Arabidopsis*, *N. benthamiana*, and tomato. A neighbour-joining tree based on predicted full-length amino acid sequences, comprising 43 AtLecRKs, 38 NbLecRKs, and 22 SlLecRKs. Branches are coloured according to the clades delineated by Bouwmeester and Govers (2009). Numbers above the branches represent the level of clade support inferred by 10 000 bootstrap replicates. The vertical branch-length scale bar represents 0.2 amino acid substitutions per site. (This figure is available in colour at *JXB* online.)

To evaluate the evolutionary relationship among LecRKs from different plant species, an unrooted phylogenetic tree containing 43 full-length AtLecRKs, 38 NbLecRKs and 22 SlLecRKs was generated using the neighbour-joining algorithm (Fig. 2). The reliability of the phylogram was determined by bootstrap analysis with 10 000 replicates. A comparable consensus tree was obtained using the maximum-likelihood algorithm with the same bootstrap replicates (data not shown). Similar phylogenetic analyses were also performed using solely the extracellular lectin domains or intracellular kinase domains, and this resulted in tree topologies that were in overall agreement with the phylogenetic tree obtained with the full-length LecRK protein sequences (Fig. 2 and Supplementary Fig. S1, available at *JXB* online). It thus seems that LecRKs that cluster in one clade based on the full-length phylogeny also have similar extracellular domains and intracellular kinase domains, and this is in line with the finding that plant RLKs containing similar extracellular domains are prone to have similar kinase domains (Shiu and Bleecker, 2003).

In the phylogenetic tree, 38 of the 43 AtLecRKs fall into nine distinct clades (I–IX) and the other five are singletons, which is in agreement with the tree constructed by Bouwmeester and Govers (2009), which included only the AtLecRKs. Moreover, the degree of similarity between LecRKs was found to be indicative of the phylogenetic relationship between the three plant species (Table 1, Fig. 2). Most of the NbLecRKs and SlLecRKs grouped together with AtLecRKs in five of the nine clades, i.e. IV, VI, VII, VIII, and IX, and with the five AtLecRK singletons. In these cases, often one AtLecRK clade member or an AtLecRK singleton grouped with one SlLecRK and two NbLecRKs in one subbranch. In such a subbranch, the NbLecRKs and SlLecRK were often closer to each other than to their *Arabidopsis* counterpart, as expected. The duplicated number of NbLecRKs is most probably due to the allotetraploid nature of *N. benthamiana*. Next to the subbranch that contained LecRK-S.4 homologues from all three species, there was a subbranch that comprised only Solanaceous LecRKs, two from tomato and five from *N. benthamiana*. In contrast to these expansions of certain clades and on certain branches of the tree, there were four clades that were expanded. None of the Solanaceous LecRKs fell into clades I, II, III, and V and similarly none of the LecRKs identified in cucumber by Wu *et al.* (2014) belonged to any of these four clades. It should be noted that the *AtLecRK*s in clades I, II, III, and V exhibit a high frequency of tandem duplication (Bouwmeester and Govers, 2009). Moreover, these four LecRK clades are well conserved among various plant species of the Brassicaceae family (Hofberger *et al.*, 2015), pointing to a lineage-specific expansion of these LecRKs after the split of the Brassicales. Several LecRKs in *Arabidopsis* belonging to clades I, II, III, and V were found to be involved in resistance to *Phytophthora*, *Alternaria brassicicola*, and bacterial pathogens (Bouwmeester *et al.*, 2011; Wang *et al.*, 2014), suggesting that this lineage-specific expansion of LecRKs is important for *Arabidopsis* adaptation. Next to the *Arabidopsis*-specific clades, the phylogenetic analysis revealed four Solanaceous-specific clades and one NbLecRK that ended up as a singleton (Fig. 2). The three Solanaceous LecRKs that carry a substitution in the RD motif formed a well-supported clade separated from other LecRKs.

### *N. benthamiana* homologues of clade IX AtLecRKs function in *Phytophthora* resistance

**Fig. 3.**
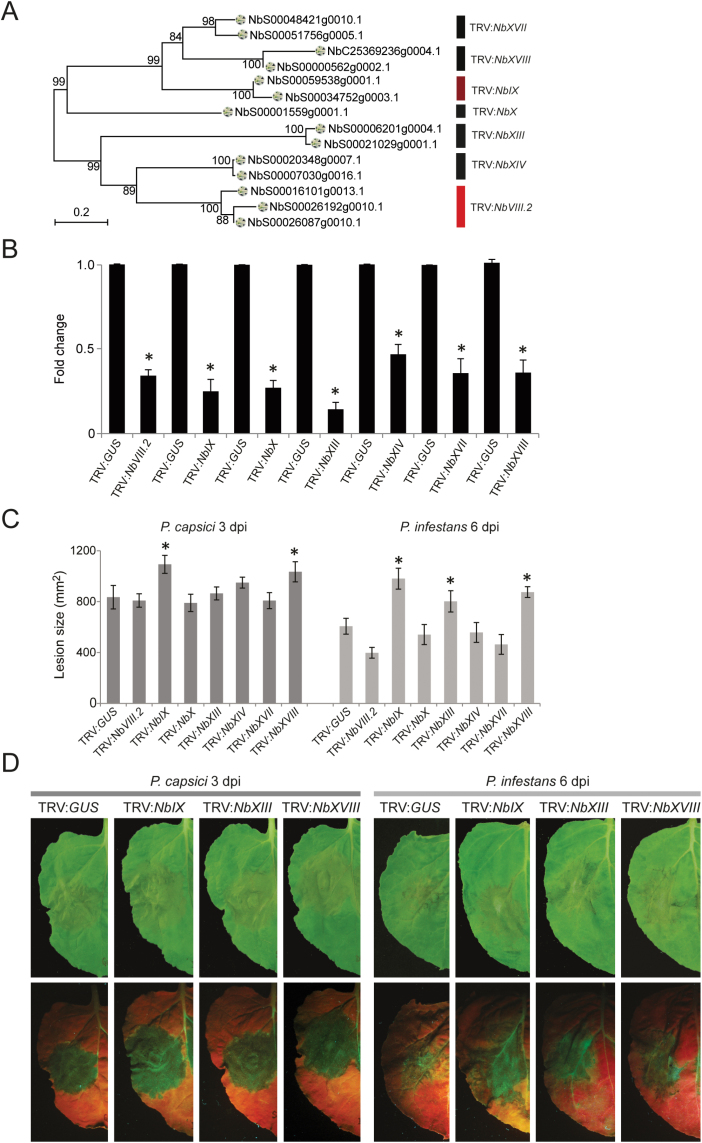
Response of *NbLecRK*-silenced *N. benthamiana* to *Phytophthora* infection. (A) *NbLecRK*s targeted by different TRV constructs. Colours refer to clades as represented in Table 1. (B) Relative *NbLecRK* transcript levels in TRV:*GUS*- and TRV:*NbLecRK*-treated *N. benthamiana* leaves. Transcript levels were normalized with *NbActin* and expressed as mean fold changes across four biological replicates (±SD) relative to the transcript level in TRV:*GUS*-treated leaves, which was arbitrarily set as 1. * indicates significant difference in expression levels (*P*<0.05, two-tailed *t*-test) between TRV:*GUS*- and TRV:*NbLecRK*-treated plants. (C) Lesion sizes on TRV:*GUS*- and TRV:*NbLecRK*-treated *N. benthamiana* leaves upon inoculation with *P. capsici* LT263 (1×10^5^ zoospores ml^–1^) and *P. infestans* 14-3-GFP (5×10^5^ zoospores ml^–1^) at 3 and 6 dpi, respectively. Bars represent mean lesion sizes (±SE) of over 20 inoculation sites from six independent plants. * indicates significant difference in lesion sizes (*P*<0.05, two-tailed *t*-test) between TRV:*GUS*- and TRV:*NbLecRK*-treated plants. This experiment was repeated four times with similar results. (D) Disease symptoms on TRV:*GUS*-, TRV:*NbIX*- TRV:*NbXIII*-, and TRV:*NbXVIII*-treated *N. benthamiana* leaves after inoculation with *P*. *capsici* at 3 dpi and *P*. *infestans* at 6 dpi. The pictures in the bottom row were taken under UV light. (This figure is available in colour at *JXB* online.)

To determine the role of NbLecRKs in *Phytophthora* resistance we selected homologues of AtLecRKs that, in a previous study, were identified as putative resistance components in *Arabidopsis* (Wang *et al.*, 2014). That study revealed 14 AtLecRKs implicated in *Phytophthora* resistance. TRV-based constructs were designed to silence *NbLecRK*s that are homologous to six of these *AtLecRK*s (i.e. *AtLecRK-VIII.2*, *AtLecRK-IX.1*, *AtLecRK-IX.2*, *AtLecRK-S.1*, *AtLecRK-S.6* and *AtLecRK-S.7*) and *NbLecRKs*, belonging to two Solanaceous-specific clades, clades XVII and XVIII. Loss of function of a single gene might fail to cause any phenotypic change as its function can be either completely or partially complemented by the presence of homologous genes. To avoid this problem, we made TRV-based constructs to simultaneously silence several *LecRK*s with high sequence similarity. As shown in Fig. 3A, seven TRV constructs were generated, each of which was designed to target only LecRKs belonging to an assigned clade. Expression levels were determined using clade-specific primers (Supplementary Table S1). In all cases, at least a 50% reduction in transcript levels of the targeted *NbLecRK*s was observed in the *NbLecRK*-silenced *N. benthamiana* plants when compared with TRV:*GUS*-treated plants (Fig. 3B). *NbLecRK* silencing did not affect plant growth and development, as none of the *NbLecRK*-silenced plants showed consistent phenotypic changes in terms of plant size, leaf colour, leaf morphology, or shoot growth (Supplementary Fig. S2, available at *JXB* online). Upon inoculation with *P. capsici* LT263 or *P. infestans* 14-3-GFP, TRV:*NbIX*- and TRV:*NbXVIII*-treated *N. benthamiana* leaves showed significantly larger lesions compared with those observed on TRV:*GUS*-treated plants (Fig. 3C, D). The increased susceptibility of TRV:*NbIX*- and TRV:*NbXVIII*-treated *N. benthamiana* plants indicated that the corresponding silenced *LecRK*s are involved in *Phytophthora* resistance. In contrast, none of the plants treated by TRV*:NbVIII.2*, TRV*:NbX*, TRV:*NbXIV*, or TRV:*NbXVII* showed significant differences in susceptibility to either *P. capsici* or *P. infestans* when compared with TRV:*GUS*-treated plants (Fig. 3C). TRV:*NbXIII*-treated plant, however, showed increased susceptibility upon infection by *P. infestans* 14-3-GFP but not when inoculated with *P. capsici* LT263 (Fig. 3C). It is possible that the activity of some of these NbLecRKs was simply too weak to prevent *Phytophthora* infection. This is exemplified by the fact that TRV:*NbXIII*-treated plants allowed *P. infestans* isolate 14-3-GFP to expand but not *P. capsici* LT263, an isolate with relatively higher virulence (Fig. 3D). Also, the sequence divergence between the AtLecRKs and their *N. benthamiana* homologues may contribute to the functional divergence that we observed.

### NbLecRK silencing does not alter elicitor-induced cell death in *N. benthamiana*

**Fig. 4.**
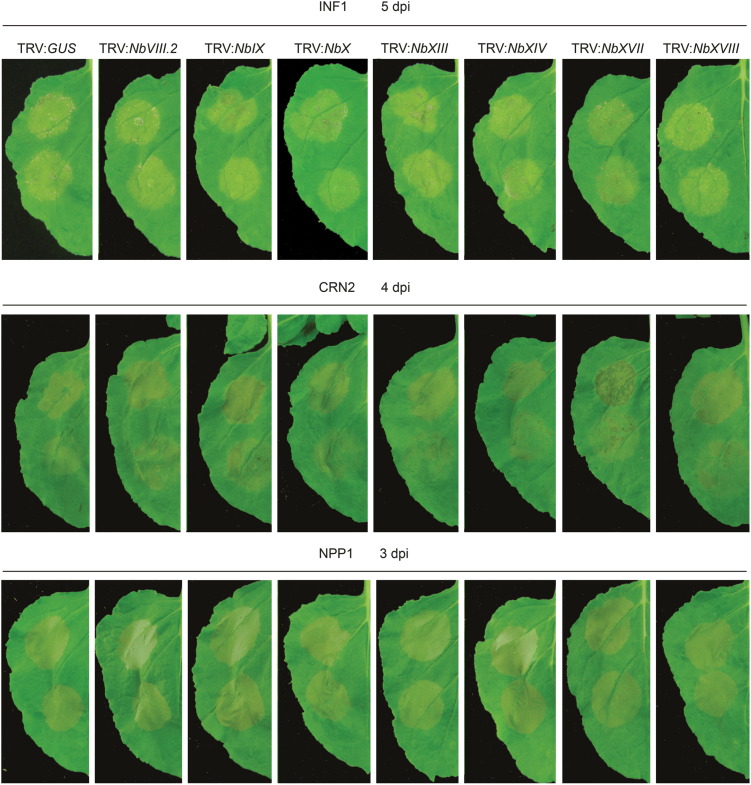
Silencing of *NbLecRK*s does not affect cell death induced by *Phytophthora* elicitors. Cell death in TRV:*GUS*- and TRV:*NbLecRK*-treated *N. benthamiana* leaves expressing *inf1*, *crn2*, and *npp1*. Each experiment included at least six leaves from three independent plants per construct. This experiment was repeated three times with similar results. (This figure is available in colour at *JXB* online.)

NbLRK1 was found previously to interact with the *P. infestans* elicitor INF1 via its kinase domain (Kanzaki *et al.*, 2008). In that study, silencing of *NbLRK1* in *N. benthamiana* compromised INF1-induced cell death and was proposed to be an important component of a host receptor complex that recognizes INF1 (Kanzaki *et al.*, 2008). We found four LecRKs in *N. benthamiana* that were homologous to NbLRK1 (Table 1). Sequence alignment of the cDNA sequences revealed that three of these four were probably silenced by the fragment used by Kanzaki *et al.* (2008) (termed *NbVIII.2-2* in Supplementary Fig. S3, available at *JXB* online), raising the question of which of the three genes is responsible for the compromised cell death that they observed. In this study, we used another fragment, named TRV:*NbVIII.2*, to simultaneously silence these three *NbLecRK*s (Fig. 3A and Supplementary Fig. S3) and confirmed by qRT-PCR using clade-specific primers that the overall *NbVIII.2* transcript level was reduced (Fig. 3B). In contrast to the results of Kanzaki *et al.* (2008), we found no difference in the appearance of cell death upon transient expression of *inf1* between *N. benthamiana* plants treated with TRV:*NbVIII.2* and TRV:*GUS* (Fig. 4). This discrepancy could be due to the fact that Kanzaki *et al.* (2008) infiltrated the leaves with INF1 protein, whereas we expressed the *inf1* gene *in planta* by *Agrobacterium*-mediated transient expression. To exclude this, we also performed the assay in a similar way as described by Kanzaki *et al.* (2008), namely by infiltrating INF1 protein into silenced *N. benthamiana* leaves, but again no differences in hypersensitive response were observed between *N. benthamiana* plants treated with TRV:*NbVIII.2* and TRV:*GUS* at 3 or 6 dpi (Supplementary Fig. S4, available at *JXB* online). In the natural situation, INF1 is secreted by *P*. *infestans* into the plant apoplast and is assumed to remain in the apoplastic space (Kamoun, 2006). Hence, it is puzzling how INF1 can interact with the cytoplasmic kinase domain of NbLRK1 to mediate cell death induction. Kanzaki *et al.* (2008) hypothesized that INF1 could be translocated into plant cells via receptor-mediated endocytosis, but they were not able to show an interaction between INF1 and NbLRK1 *in planta*. More recently, it was shown that INF1 recognition in *Solanum microdontum* is mediated by the receptor-like protein ELR (Du *et al.* 2015).

We also investigated whether other NbLecRKs affected cell death triggered by INF1 and whether NbLecRKs play a role in cell death triggered by two other *Phytophthora* elicitors, CRN2 and NPP1. Transient expression of *inf1*, *crn2*, and *npp1* in *NbLecRK*-silenced plants resulted in cell death in all cases, and no visible differences in the appearance of cell death were observed when compared with TRV:*GUS*-treated leaves (Fig. 4). These results suggested that none of the tested NbLecRKs plays an essential role in cell death induction trigged by these three elicitors.

### Silencing of the tomato LecRK homologue of *AtLecRK-IX.1*/*LecRK-IX.2* reduces *Phytophthora* resistance

**Fig. 5.**
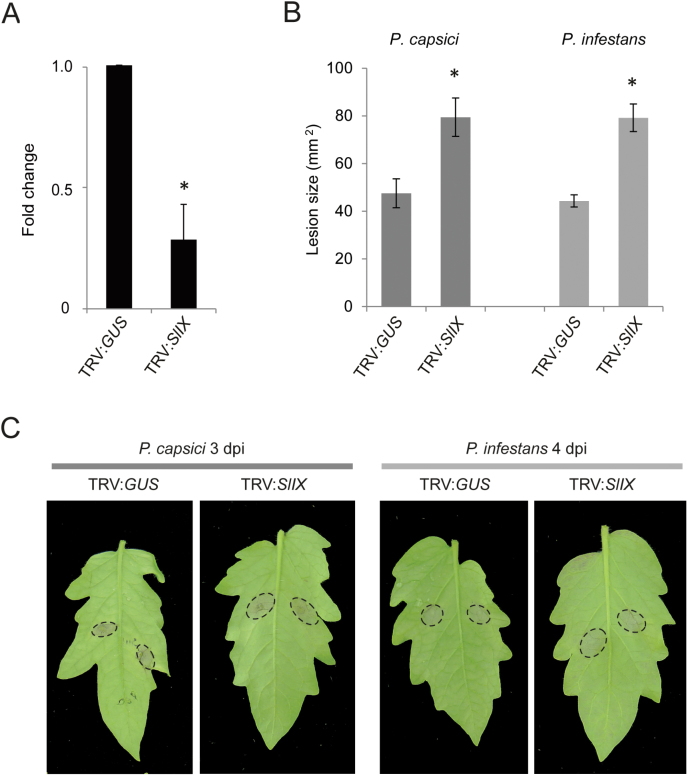
Silencing of *SlLecRK-IX.1/LecRK-IX.2* compromises *Phytophthora* resistance in tomato. (A) Relative *SlLecRK* transcript levels in TRV:*GUS*- and TRV:*SlIX*-treated tomato leaves. Transcript levels were normalized with *SlActin* and expressed as mean fold changes across four biological replicates (±SD) relative to the transcript level in TRV*:GUS*-treated leaves, which was arbitrarily set as 1. * indicates significant difference in expression levels (*P*<0.05, two-tailed *t*-test) between TRV:*GUS*- and TRV:*SlIX*-treated plants. (B, C) Disease symptoms (B) and quantified lesion sizes (C) on TRV:*GUS*- and TRV:*SlIX*-treated tomato leaves inoculated with *P. capsici* LT263 (1×10^5^ zoospores ml^–1^) and *P. infestans* 14-3-GFP (5×10^5^ zoospores ml^–1^). Each experiment included at least 20 leaves from four independent plants treated with each construct. Bars represent the mean lesion sizes (±SE). * indicates significant difference (*P*<0.05) in lesion sizes between TRV:*GUS*- and TRV:*SlIX*-treated plants according to a two-tailed *t*-test. The infection assay with *P. capsici* was performed twice, whereas infection assays with *P. infestans* were repeated four times with similar results. (This figure is available in colour at *JXB* online.)

To assess whether the homologue of *AtLecRK-IX.1/LecRK-IX.2* in tomato also plays a role in *Phytophthora* resistance, the TRV construct TRV:*SlIX* was generated to silence *Solyc03g043710.1.1*, the homologue closest to *LecRK-IX.1* and *LecRK-IX.2* (Fig. 2). Quantitative analysis of transcript levels in TRV:*SlIX*-treated plants revealed around a 60% reduction in expression in comparison with TRV:*GUS*-treated plants (Fig. 5A). Inoculation with zoospores of *P. capsici* LT263 or *P. infestans* 14-3-GFP on TRV:*SlIX*-treated plants resulted in lesions that were significantly larger than the lesions on TRV:*GUS*-treated plants (Fig. 5B, C). Larger lesions were also observed on TRV:*SlIX*-treated tomato leaves upon inoculation with mycelial plugs of *P. capsici* LT263 (Supplementary Fig. S5, available at *JXB* online). Taken together, these results indicated that the closely related tomato homologue of AtLecRK-IX.1 and AtLecRK-IX.2 plays a role in *Phytophthora* resistance similar to NbLecRK-IX.1/2, the homologue in *N. benthamiana*.

### Conclusions

In total, 38 and 22 LecRKs were identified in the genomes of *N. benthamiana* and tomato, respectively. Multiple Solanaceous *LecRK* gene models deposited in the SGN database were found to be erroneous due to mis-prediction of the open reading frame and were corrected based on transcriptome data. Domain composition analysis indicated that most of the identified LecRKs have a typical LecRK structure, but there are several LecRKs, especially those from *N. benthamiana*, that lack an SP domain, a TM domain, or both. Phylogenetic analysis revealed that most of the Solanaceous LecRKs group together with *Arabidopsis* LecRKs, whereas four clades seem to be Solanaceous specific. For TRV-mediated gene silencing, we designed constructs that targeted all genes within a specific clade. Functional analysis using these constructs demonstrated that homologues of *AtLecRK-IX.1*/*LecRK-IX.2* in both *N. benthamiana* and tomato function in resistance to different *Phytophthora* pathogens, and apparently the *Phytophthora* resistance function of clade IX LecRKs is conserved across different plant species. Although computational sequence analysis confirmed that the constructs were clade specific, we cannot fully exclude off-target silencing, nor can we predict to what extent the expression level of an individual gene within a clade is affected. Future research focused at unravelling the role of Solanaceous LecRKs in *Phytophthora* resistance requires more precise methods that allow functional analyses of each individual gene either within clade IX or within the Solanaceous-specific LecRK clades.

## Supplementary data

Supplementary data are available at *JXB* online.

Supplementary Table S1. Primers used in this study.

Supplementary Fig. S1. Neighbour-joining trees constructed based on the lectin domains (A) and kinase domains (B) of 43 AtLecRKs, 38 NbLecRKs and 22 SlLecRKs.

Supplementary Fig. S2. Morphology of *N. benthamiana* plants treated by TRV:*NbLecRKs*, TRV:*PDS* and TRV:*GUS*.

Supplementary Fig. S3. Sequence alignment of NbS00016101g0013.1, NbS00026192g0010.1, NbS00026 087g0010.1 and the fragments used for silencing.

Supplementary Fig. S4. Cell death induced by INF1 on TRV:*GUS*- and TRV:*NbVIII.2*-treated plants. Pictures were taken at three and six days after syringe-infiltration. Each experiment consisted of at least six infiltration sites. This experiment was repeated twice with similar results.

Supplementary Fig. S5. Quantified lesion sizes on TRV:*GUS*- and TRV:*SlIX*-treated tomato leaves 3 d after inoculation with *P. capsici* plugs (diameter 0.5cm). This experiment included 16 leaves from four independent plants treated with each construct. * indicates significant difference (*P* < 0.05) in lesion sizes between TRV:GUS- and TRV:*SlIX*-treated plants according to a two-tailed *t* test. This experiment was repeated twice with similar results.

Supplementary File S1. LecRK sequences of *N. benthamiana* and tomato. Highlighted protein sequences where shown to be inconsistent with RNA-seq data. Revised sequences are labelled ‘Corrected’. Introns within genomic sequences are underlined.

Supplementary File S2. DNA sequences of the fragments used for TRV-mediated silencing in *N. benthamiana* and tomato.

Supplementary Data

## Abbreviations:

DAMP: damage-associated molecular pattern
dpi: days post-inoculation
EST: expressed sequence tag
ETI: effector-triggered immunity
LecRK: L-type lectin receptor kinase
MAMP: microbe-associated molecular pattern
NLR: nucleotide-binding leucine-rich repeat receptor
PRR: pattern recognition receptor
qRT-PCR: quantitative reverse transcription PCR
RLK: receptor-like kinase
RNA-seq: RNA sequencing
SP: signal peptide
TM: transmembrane
TRV: tobacco rattle virus.

## References

[CIT0001] Afzal AJ Wood AJ Lightfoot DA 2008 Plant receptor-like serine threonine kinases: roles in signaling and plant defense. Molecular Plant–Microbe Interactions 21, 507–517. 18393610 10.1094/MPMI-21-5-0507

[CIT0002] Boller T Felix G 2009 A renaissance of elicitors: perception of microbe-associated molecular patterns and danger signals by pattern-recognition receptors. Annual Review of Plant Biology 60, 379–406. 10.1146/annurev.arplant.57.032905.105346 19400727

[CIT0003] Bouwmeester K de Sain M Weide R Gouget A Klamer S Canut H Govers F 2011 The lectin receptor kinase LecRK-I.9 is a novel *Phytophthora* resistance component and a potential host target for a RXLR effector. PLoS Pathogens 7, e1001327. 21483488 10.1371/journal.ppat.1001327 PMC3068997

[CIT0004] Bouwmeester K Govers F 2009 *Arabidopsis* L-type lectin receptor kinases: phylogeny, classification, and expression profiles. Journal of Experimental Botany 60, 4383–4396. 19773388 10.1093/jxb/erp277

[CIT0005] Bouwmeester K Han M Blanco-Portales R Song W Weide R Guo L van der Vossen EAG Govers F 2014 The *Arabidopsis* lectin receptor kinase LecRK-I.9 enhances resistance to *Phytophthora infestans* in Solanaceous plants. Plant Biotechnology Journal 12, 10–16. 23980842 10.1111/pbi.12111

[CIT0006] Bouwmeester K van Poppel PMJA Govers F 2009 Genome biology cracks enigmas of oomycete plant pathogens. In: Parker J, ed. Molecular aspects of plant disease resistance. Oxford, UK: Wiley-Blackwell, 102–133.

[CIT0007] Champouret N Bouwmeester K Rietman H 2009 *Phytophthora infestans* isolates lacking class I *ipiO* variants are virulent on *Rpi-blb1* potato. Molecular Plant–Microbe Interactions 22, 1535–1545. 19888819 10.1094/MPMI-22-12-1535

[CIT0008] Choi J Tanaka K Cao Y Qi Y Qiu J Liang Y Lee SY Stacey G 2014 Identification of a plant receptor for extracellular ATP. Science 343, 290–294. 24436418 10.1126/science.343.6168.290

[CIT0009] Dardick C Ronald P 2006 Plant and animal pathogen recognition receptors signal through non-RD kinases. PLoS Pathogens 2, 14–28. 10.1371/journal.ppat.0020002 PMC1331981 16424920

[CIT0010] Desclos-Theveniau M Arnaud D Huang TY Lin GJC Chen WY Lin YC Zimmerli L 2012 The *Arabidopsis* lectin receptor kinase LecRK-V.5 represses stomatal immunity induced by *Pseudomonas syringae* pv. *tomato* DC3000. PLoS Pathogens 8, e1002513. 22346749 10.1371/journal.ppat.1002513 PMC3276567

[CIT0011] Du J Verzaux E Chaparro-Garcia A 2015 Elicitin recognition confers enhanced resistance to *Phytophthora infestans* in potato. Nature Plants 1, doi:10.1038/nplants.2015.34. 10.1038/nplants.2015.34 27247034

[CIT0012] Fry W 2008 *Phytophthora infestans*: the plant (and *R* gene) destroyer. Molecular Plant Pathology 9, 385–402. 18705878 10.1111/j.1364-3703.2007.00465.x PMC6640234

[CIT0013] Gomez-Gomez L Boller T 2000 FLS2: An LRR receptor-like kinase involved in the perception of the bacterial elicitor flagellin in *Arabidopsis* . Molecular Cell 5, 1003–1011. 10911994 10.1016/s1097-2765(00)80265-8

[CIT0014] Gouget A Senchou V Govers F Sanson A Barre A Rouge P Pont-Lezica RP Canut H 2006 Lectin receptor kinases participate in protein-protein interactions to mediate plasma membrane-cell wall adhesions in *Arabidopsis* . Plant Physiology 140, 81–90. 16361528 10.1104/pp.105.066464 PMC1326033

[CIT0015] Gouhier-Darimont C Schmiesing A Bonnet C Lassueur S Reymond P 2013 Signalling of Arabidopsis thaliana response to Pieris brassicae eggs shares similarities with PAMP-triggered immunity. Journal of Experimental Botany 64, 665–674. 23264520 10.1093/jxb/ers362 PMC3542055

[CIT0016] Hall BG 2013 Building phylogenetic trees from molecular data with MEGA. Molecular Biology and Evolution 30, 1229–1235. 23486614 10.1093/molbev/mst012

[CIT0017] Hanks SK 2003 Genomic analysis of the eukaryotic protein kinase superfamily: a perspective. Genome Biology 4, 111. 12734000 10.1186/gb-2003-4-5-111 PMC156577

[CIT0018] Hann DR Rathjen JP 2007 Early events in the pathogenicity of *Pseudomonas syringae* on *Nicotiana benthamiana* . The Plant Journal 49, 607–618. 17217460 10.1111/j.1365-313X.2006.02981.x

[CIT0019] Hofberger JA Nsibo DL Govers F Bouwmeester K Schranz ME 2015 A complex interplay of tandem- and whole genome duplication drives expansion of the L-type lectin receptor kinase gene family in the Brassicaceae. Genome Biology and Evolution 7, 720–734. 25635042 10.1093/gbe/evv020 PMC5322546

[CIT0020] Huang PY Zimmerli L 2014 Enhancing crop innate immunity: new promising trends. Frontiers in Plant Science 5, 624. 25414721 10.3389/fpls.2014.00624 PMC4222232

[CIT0021] Kamoun S 2006 A catalogue of the effector secretome of plant pathogenic oomycetes. *Annual* Review of Phytopathology 44, 41–60. 10.1146/annurev.phyto.44.070505.143436 16448329

[CIT0022] Kanzaki H Saitoh H Takahashi Y Berberich T Ito A Kamoun S Terauchi R 2008 NbLRK1, a lectin-like receptor kinase protein of *Nicotiana benthamiana*, interacts with *Phytophthora infestans* INF1 elicitin and mediates INF1-induced cell death. Planta 228, 977–987. 18682978 10.1007/s00425-008-0797-y

[CIT0023] Kasuga T Gijzen M 2013 Epigenetics and the evolution of virulence. Trends in Microbiology 21, 575–582. 24095304 10.1016/j.tim.2013.09.003

[CIT0024] Koornneef M Meinke D 2010 The development of *Arabidopsis* as a model plant. The Plant Journal 61, 909–921. 20409266 10.1111/j.1365-313X.2009.04086.x

[CIT0025] Kornev AP Haste NM Taylor SS Ten Eyck LF 2006 Surface comparison of active and inactive protein kinases identifies a conserved activation mechanism. Proceedings of the National Academy of Sciences, USA 103, 17783–17788. 10.1073/pnas.0607656103 PMC1693824 17095602

[CIT0026] Kroon LPNM Brouwer H de Cock AWAM Govers F 2012 The genus *Phytophthora* anno 2012. Phytopathology 102, 348–364. 22185336 10.1094/PHYTO-01-11-0025

[CIT0027] Lamour KH Stam R Jupe J Huitema E 2012 The oomycete broad-host-range pathogen *Phytophthora capsici* . Molecular Plant Pathology 13, 329–430. 22013895 10.1111/j.1364-3703.2011.00754.x PMC6638677

[CIT0028] Naim F Nakasugi K Crowhurst RN Hilario E Zwart AB Hellens RP Taylor JM Waterhouse PM Wood CC 2012 Advanced engineering of lipid metabolism in *Nicotiana benthamiana* using a draft genome and the V2 viral silencing-suppressor protein. PLoS One 7, e52717. 23300750 10.1371/journal.pone.0052717 PMC3530501

[CIT0029] Nakasugi K Crowhurst RN Bally J Wood CC Hellens RP Waterhouse PM 2013 De novo transcriptome sequence assembly and analysis of RNA silencing genes of *Nicotiana benthamiana* . PLoS One 8, e59534. 23555698 10.1371/journal.pone.0059534 PMC3610648

[CIT0030] Nolen B Taylor S Ghosh G 2004 Regulation of protein kinases: controlling activity through activation segment conformation. Molecular Cell 15, 661–675. 15350212 10.1016/j.molcel.2004.08.024

[CIT0031] Phillips SM Dubery IA van Heerden H 2013 Identification and molecular characterisation of a lectin receptor-like kinase (GhLecRK-2) from cotton. Plant Molecular Biology Reporter 31, 9–20.

[CIT0032] Robatzek S Bittel P Chinchilla D Köchner P Felix G Shiu SH Boller T 2007 Molecular identification and characterization of the tomato flagellin receptor LeFLS2, an orthologue of *Arabidopsis* FLS2 exhibiting characteristically different perception specificities. Plant Molecular Biology 64, 539–547. 17530419 10.1007/s11103-007-9173-8

[CIT0033] Shiu SH Bleecker AB 2001 Receptor-like kinases from *Arabidopsis* form a monophyletic gene family related to animal receptor kinases. Proceedings of the National Academy of Sciences, USA 98, 10763–10768. 10.1073/pnas.181141598 PMC58549 11526204

[CIT0034] Shiu SH Bleecker AB 2003 Expansion of the receptor-like kinase/Pelle gene family and receptor-like proteins in *Arabidopsis* . Plant Physiology 132, 530–543. 12805585 10.1104/pp.103.021964 PMC166995

[CIT0035] Singh P Chien CC Mishra S Tsai CH Zimmerli L 2013 The *Arabidopsis* LECTIN RECEPTOR KINASE-VI.2 is a functional protein kinase and is dispensable for basal resistance to *Botrytis cinerea* . Plant Signaling & Behavior 8, 1–3. 10.4161/psb.22611 PMC3745566 23221759

[CIT0036] Singh P Kuo YC Mishra S 2012 The lectin receptor kinase-VI.2 is required for priming and positively regulates *Arabidopsis* pattern-triggered immunity. The Plant Cell 24, 1256–1270. 22427336 10.1105/tpc.112.095778 PMC3336125

[CIT0037] Takai R Isogai A Takayama S Che FS 2008 Analysis of flagellin perception mediated by flg22 receptor OsFLS2 in rice. Molecular Plant–Microbe Interactions 21, 1635–1642. 18986259 10.1094/MPMI-21-12-1635

[CIT0038] Vleeshouwers VGAA van Dooijeweert W Keizer LCP Sijpkes L Govers F Colon LT 1999 A laboratory assay for *Phytophthora infestans* resistance in various *Solanum* species reflects the field situation. European Journal of Plant Pathology 105, 241–250.

[CIT0039] Vleeshouwers VGAA Raffaele S Vossen JH 2011 Understanding and exploiting late blight resistance in the age of effectors. Annual Review of Phytopathology 49, 507–531. 10.1146/annurev-phyto-072910-095326 21663437

[CIT0040] Wang Y Bouwmeester K Beseh P Shan W Govers F 2014 Phenotypic analyses of *Arabidopsis* T-DNA insertion lines and expression profiling reveal that multiple L-type lectin receptor kinases are involved in plant immunity. Molecular Plant–Microbe Interactions 27, 1390–1402. 25083911 10.1094/MPMI-06-14-0191-R

[CIT0041] Wang Y Bouwmeester K van de Mortel JE Shan W Govers F 2013 A novel *Arabidopsis*-oomycete pathosystem: differential interactions with *Phytophthora capsici* reveal a role for camalexin, indole glucosinolates and salicylic acid in defense. Plant, Cell & Environment 36, 1192–1203. 10.1111/pce.12052 23237451

[CIT0042] Wang Y Zhang M Ke K Lu Y 2005 Cellular localization and biochemical characterization of a novel calcium-dependent protein kinase from tobacco. Cell Research 15, 604–612. 16117850 10.1038/sj.cr.7290330

[CIT0043] Wu T Wang R Xu X He X Sun B Zhong Y Liang Z Luo S Lin Y 2014 *Cucumis sativus* L-type lectin receptor kinase (CsLecRK) gene family respond to *Phytophthora melonis*, *Phytophthora capsici* and water immersion in disease resistant and susceptible cucumber cultivars. Gene 549, 214–222. 25065921 10.1016/j.gene.2014.07.058

