# Supplementary material for: L-type lectin receptor kinases in Nicotiana benthamiana and tomato and their role in Phytophthora resistance
Source: J Exp Bot. 2015 Aug 5;66(21):6731–43. doi: 10.1093/jxb/erv379 (PMC4623685; doi:10.1093/jxb/erv379)
Supplement: Supplementary Data [file supp_66_21_6731__index.html]

L-type lectin receptor kinases in Nicotiana benthamiana and tomato and their role in Phytophthora resistance — Supplementary Data 

# L-type lectin receptor kinases in *Nicotiana benthamiana* and tomato and their role in *Phytophthora* resistance

## Supplementary Data

Data files

- Supplementary Data - Supplementary Data
- Supplementary Data - Supplementary Data
- Supplementary Data - Supplementary Data
- Supplementary Data - Supplementary Data
- Supplementary Data - Supplementary Data
- Supplementary Data - Supplementary Data
- Supplementary Data - Supplementary Data
- Supplementary Data - Supplementary Data
