# Supplementary material for: L-type lectin receptor kinases in Nicotiana benthamiana and tomato and their role in Phytophthora resistance
Source: J Exp Bot. 2015 Aug 5;66(21):6731–43. doi: 10.1093/jxb/erv379 (PMC4623685; doi:10.1093/jxb/erv379)
Supplement: Supplementary Data [file supp_erv379_Figure_S5___Legend.pdf]

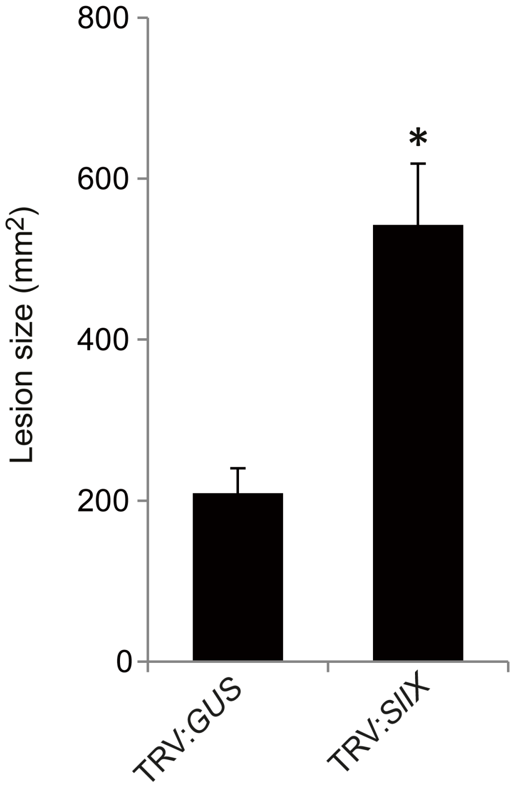

Supplementary Fig. S5.
Quantified lesion sizes on TRV:*GUS*- and TRV:*SlIX*-treated tomato leaves three days after inoculation with *P. capsici* plugs (Ø 0.5 cm). This experiment included 16 leaves from four independent plants treated with each construct. * indicates significant difference ($p < 0.05$) in lesion sizes between TRV:*GUS*- and TRV:*SlIX*-treated plants according to a two-tailed *t* test. This experiment was repeated twice with similar results.
