# Supplementary material for: L-type lectin receptor kinases in Nicotiana benthamiana and tomato and their role in Phytophthora resistance
Source: J Exp Bot. 2015 Aug 5;66(21):6731–43. doi: 10.1093/jxb/erv379 (PMC4623685; doi:10.1093/jxb/erv379)
Supplement: Supplementary Data [file supp_erv379_Figure_S1___Legend.pdf]

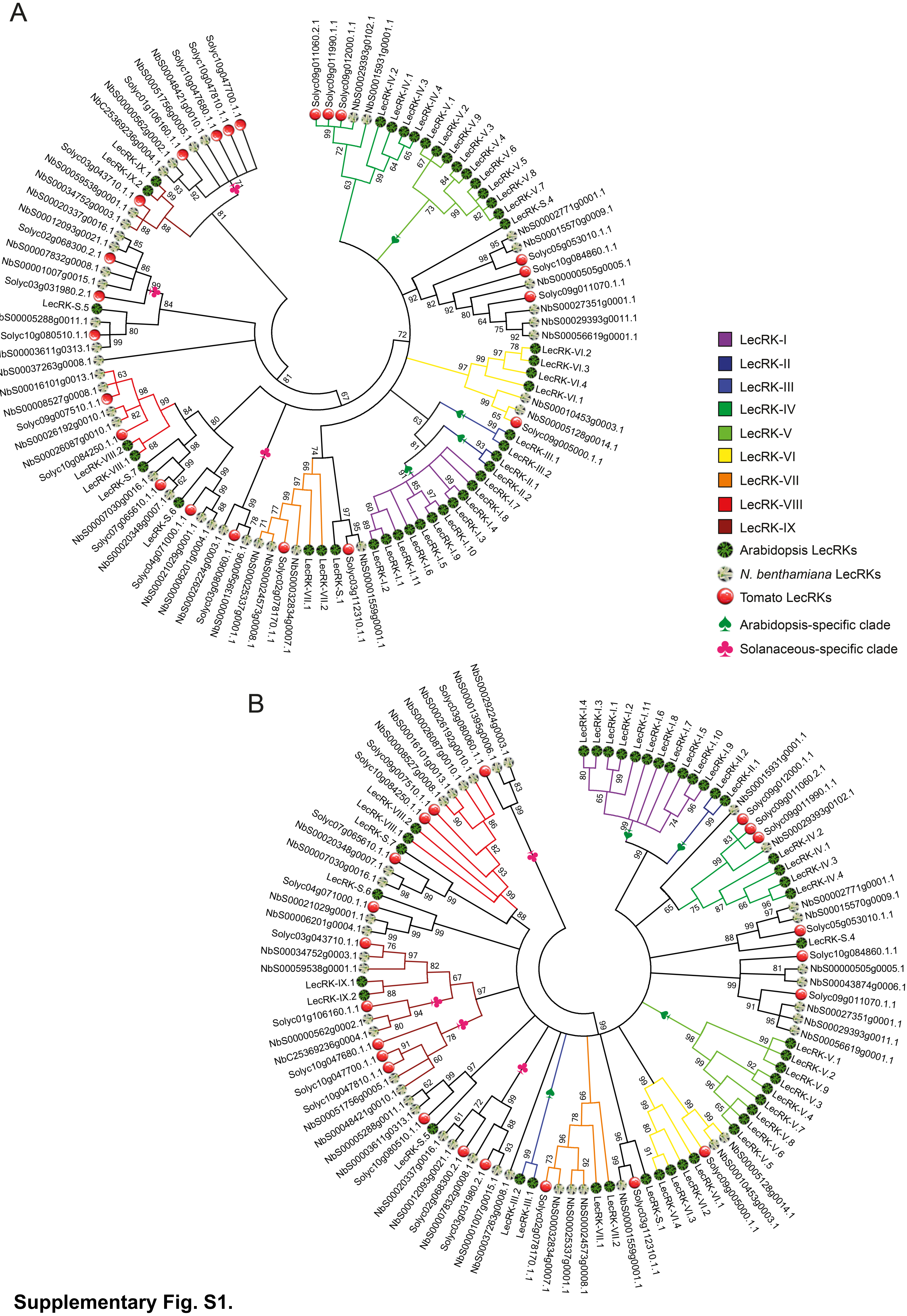

Supplementary Fig. S1.
Neighbor-Joining trees constructed based on the lectin domains (A) and kinase domains (B) of 43 AtLecRKs, 38 NbLecRKs and 22 SlLecRKs.
