# Supplementary material for: L-type lectin receptor kinases in Nicotiana benthamiana and tomato and their role in Phytophthora resistance
Source: J Exp Bot. 2015 Aug 5;66(21):6731–43. doi: 10.1093/jxb/erv379 (PMC4623685; doi:10.1093/jxb/erv379)
Supplement: Supplementary Data [file supp_erv379_Figure_S2___Legend.pdf]

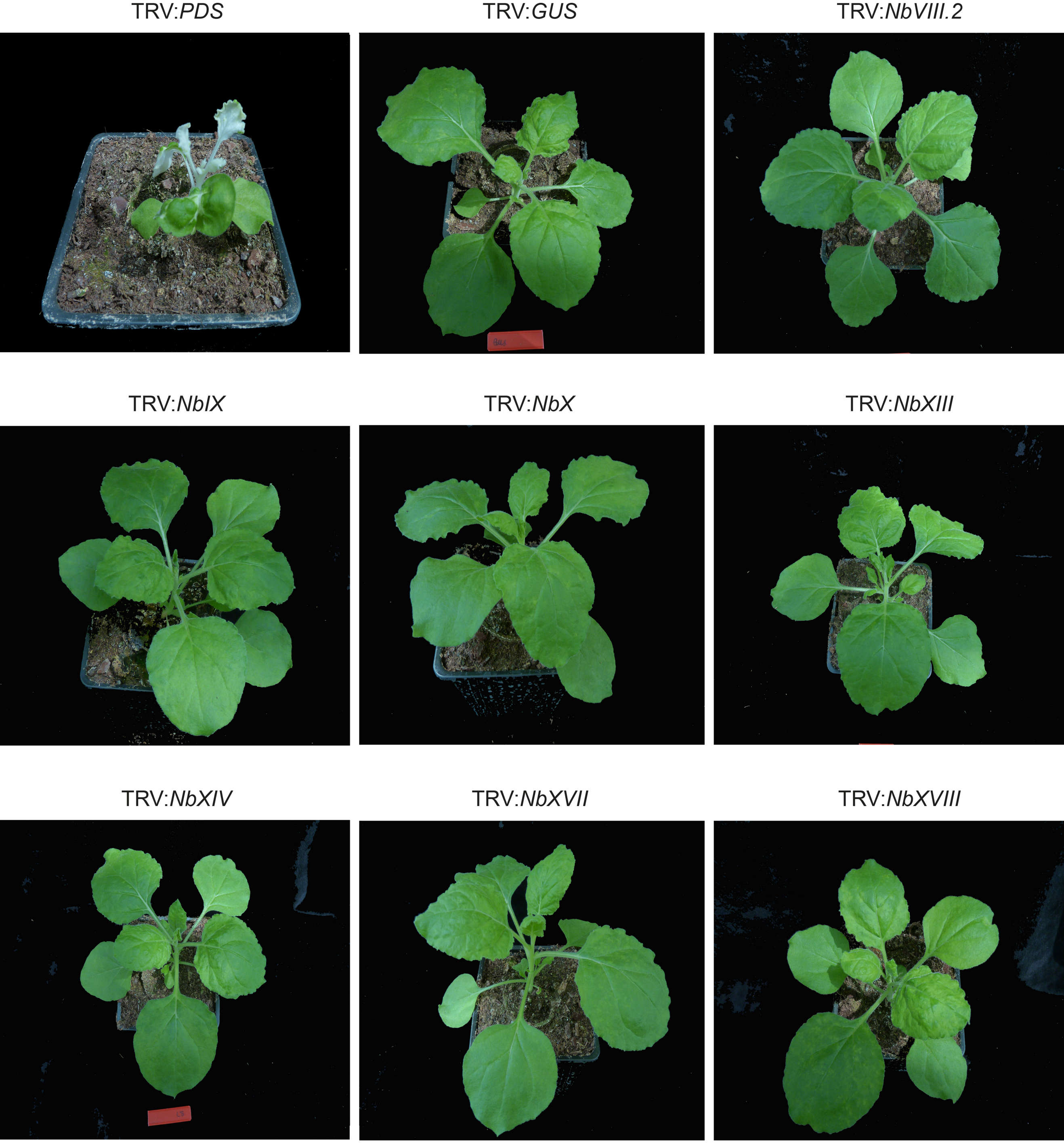

Supplementary Fig. S2.
Morphology of *N. benthamiana* plants treated by TRV:*NbLecRKs*, TRV:*PDS* and TRV:*GUS*.
