# Supplementary material for: L-type lectin receptor kinases in Nicotiana benthamiana and tomato and their role in Phytophthora resistance
Source: J Exp Bot. 2015 Aug 5;66(21):6731–43. doi: 10.1093/jxb/erv379 (PMC4623685; doi:10.1093/jxb/erv379)
Supplement: Supplementary Data [file supp_erv379_Figure_S3___Legend.pdf]

```
NbS00026192g0010.1    1315  CTTATGCCTAATGGGAGTCTTGATAAGGCATTATTTGAATCAAGAATGATTCTACCTTGG
NbS00026087g0010.1    1315  CTAATGCCTAATGGGAGTCTTGATAAGGCATTATTTGAATCAAGAATGATTCTACCTTGG
NbVIII.2                 1  --------------------GAATTCGGCATTATTTGAATCAAGAATGATTCTACCTTGG
NbS00016101g0013.1    1318  TTGATGCCAAATGGTAGTCTTGATAAGGCATTATTTGAGTCAAGAATGGTACTCCCATGG

NbS00026192g0010.1    1375  GTACATAGGAGGAAAATTTTGCTAGGTGTTGCTTCAGCCTTGGCATATTTACATCAAGAA
NbS00026087g0010.1    1375  CTACATAGGAGGAAAATTTTGCTAGGTGTTGCTTCAGCCTTGGCATATTTACATCAAGAA
NbVIII.2                41  CTACATAGGAGGAAAATTTTGCTAGGTGTTGCTTCAGCCTTGGCATATTTACATCAAGAA
NbS00016101g0013.1    1378  CTACATAGGAGGAAAATTTTGTTAGGTGTTGCTTCAGCCTTAGCATATTTACATCAAGAA

NbS00026192g0010.1    1435  TGTGAAAACCAGGTGATTCACAGGGATATAAAGAGTAGTAACATTATGTTGGATGAAGGG
NbS00026087g0010.1    1435  TGTGAAAATCAGGTGATTCATAGGGATATAAAGAGTAGTAACATTATGTTAGATGAAGGG
NbVIII.2               101  TGTGAAAATCAGGTGATTCATAGGGATATAAAGAGTAGTAACATTATGTTAGATGAAGGG
NbS00016101g0013.1    1438  TGTGAAAATCAAGTTATGTTAAGGGATATTAAGACTAGTAATATTATGTTGGATGAAGGG

NbS00026192g0010.1    1495  TTCAATGCAAGATTAGGTGATTTTGGATTAGCAAGACAAGTTGAACATGACAAGTCCCCC
NbS00026087g0010.1    1495  TTCAATGCAAGATTAGGTGATTTTGGATTAGCAAGACAAGTTGAACATGACAAGTCCCCC
NbVIII.2               161  TTCAATGCAAGATTAGGTGATTTTGGATTAGCAAGACAAGTTGAACATGACAAGTCCCCC
NbS00016101g0013.1    1498  TTTAATGCAAGATTAGGTGATTTTGGGTTAGCAAAACAAATTGAACATGACAAATCTCCT

NbS00026192g0010.1    1555  GATGTAACAGTAGCAGCTGGGACAATGGGCTACTTGGCTCCTGAATACTTGTTAACCGGA
NbS00026087g0010.1    1555  GATGCAACGGTAGCAGCCGGGACAATGGGCTACTTGGCTCCTGAATACTTGTTAACCGGA
NbVIII.2               221  GATGCAACGGTAGCAGCCGGGACAATGGGCTACTTGGCTCCTGAATACTTGTTAACCGGA
NbS00016101g0013.1    1558  GATGCGACAGTAGCAGCAGGAACAATGGGATACTTAGCACCTGAATATTTATTAACCGGT

NbS00026192g0010.1    1615  AGAGCAACCGAAAAAACTGATGTTTTTAGCTATGGAGCTGTGGTTCTTGAAGTGGCAAGT
NbS00026087g0010.1    1615  AGAGCAACCGAAAAAACTGATGTTTTTAGCTATGGAGCAGTTGTTCTTGAAGTGGCAAGT
NbVIII.2               281  AGAGCAACCGAAAAAACTGATGTTTTTAGCTATGGAGCAGTTGTTCTTGAAGTGGCAAGT
NbS00016101g0013.1    1618  CGAGCAAGTGAAAAAACTGATGCTTTTAGCTATGGCGCAGTGGTTCTTGAAGTGGCAAGT

NbS00026192g0010.1    1675  GGAAGGAGGCCAATTGAGAGGGAAACAACAAGAGTTGAGAAAGTTGGAGTGAAGAGTAAC
NbS00026087g0010.1    1675  GGAAGGAGGCCAATTGAGAGGGAAACAACAAGAGTTGAGAAAGTTGGAGTGAATAGTAAC
NbVIII.2               341  GGAAGGAGGCCAATTGAGAGGGAAACAACAAGAGTTGAGAAAGTTGGAGTGAATAGTAAC
NbS00016101g0013.1    1678  GGGAGAAGGCCAATTGAGAAAGAAACAAATGGGGTTGGGAAAGTTGGATTAAATAGCAAT

NbS00026192g0010.1    1735  TTAGTTGAATGGGTTTGGGGATTGCATAGAGAAGGGAATTTGCTAATGGCAGCAGATTCA
NbS00026087g0010.1    1735  TTAGTTGAATGGGTTTGGGGATTGCATAGAGAAGGGAATTTGCTAATGGCAGCTGATTCA
NbVIII.2               401  TTAGTTCCCGGG------------------------------------------------
NbS00016101g0013.1    1738  TTGGTGGAATGGGTTTGGGGATTACATAAAGAAGGGAGGTTATTAGCAGCAGCTGATTCA

NbS00026192g0010.1    1855  TGTTCACAACCTGACCCTATGGTTAGACCAACAATGAGAAGTGTGGTCCAAATGCTAGTA
NbS00026087g0010.1    1855  TGTTCACAACCTGACCCTATGGTTAGACCAACAATGAGAAGTGTGGTCCAAATGCTAGTA
NbVIII.2-2               1  ------------GACCCTATGGTTAGACCAACAATGAGAAGTGTGGTCCAAATGCTAGTA
NbS00016101g0013.1    1858  TGTTCACACCCTGACCCTATGGCTAGACCAACAATGAGAGGTGTGGTTCAAATGTTAGTT

NbS00026192g0010.1    1915  GGTGAAGCTGAAGTTCCTATTGTCCCAAGAACTAAGCCTTGTATGAGTTTCAGCACATCA
NbS00026087g0010.1    1915  GGTGAAGCTGAAGTTCCTATTGTCCCAAGAACTAAGCCTTCTATGAGTTTCAGCACATCA
NbVIII.2-2              49  GGTGAAGCTGAAGTACCTATTGTCCCAAGAACTAAGCCTTCTATGAGTTTCAGCACATCA
NbS00016101g0013.1    1918  GGTGAGGCTGAAGTTCCAATTGTCCCTAGAGCTAAGCCAACTATGAGTTTTAGCACATCT
```

NbS00026192g0010.1	1975	CATCTTCTAATGACTTTGCAAGATAGTGTTTCTGATTTGAATGGTATGATCACACTTTCC
NbS00026087g0010.1	1975	CATCTTCTAATGACTTTGCAAGATAGTGTTTCTGATTTGAATGGTATGATCACACTTTCA
NbVIII.2-2	109	CATCTTCTAATGACTTTGCAAGATAGTGTTTCTGATTTGAATGGTATGATCACACTTTCC
NbS00016101g0013.1	1978	CATCTGCTAATGACTTTGCAAGATAGTGTTTCTGACTTGAATGGTATGATCACACTTTCC

NbS00026192g0010.1	2035	TCTTCATCATCTGAAAACAGTTTCACTGGTGTTGCCAATGGGATGGACTTGGTCTAA
NbS00026087g0010.1	2035	ACTTCATCATCTGAAAACAGCTTCAATAGTGCTGGCAATGGCATGGACTTGGTCTAA
NbVIII.2-2	169	ACTTCATCATCTGAAAACAGTTTCACTGGTGTTGCCCATGGGATGA---TGGA----
NbS00016101g0013.1	2038	ACGTCTTCATCTGAAAATAGCTTCAATGGTGGTGGTGTGGGTATGGACCTAGTCTAG

Supplementary Fig. S3. Sequence alignment of NbS00016101g0013.1, NbS00026192g0010.1, NbS00026087g0010.1 and the fragments used for silencing. NbVIII.2 represents the fragment used in this study, while NbVIII.2-2 represents the fragment used by Kanzaki *et al*. (2008).
