# Supplementary material for: L-type lectin receptor kinases in Nicotiana benthamiana and tomato and their role in Phytophthora resistance
Source: J Exp Bot. 2015 Aug 5;66(21):6731–43. doi: 10.1093/jxb/erv379 (PMC4623685; doi:10.1093/jxb/erv379)
Supplement: Supplementary Data [file supp_erv379_Figure_S4___Legend.pdf]

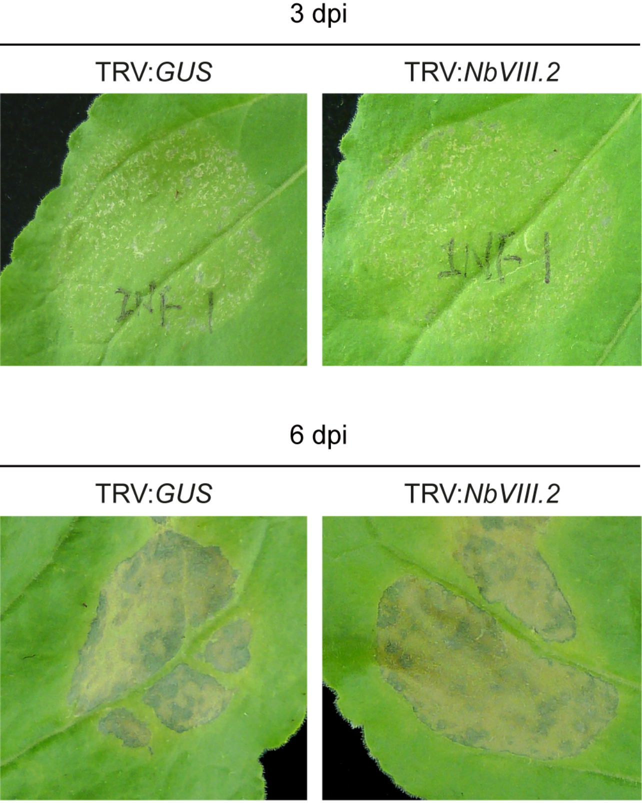


**Supplementary Fig. S4.**
Cell death induced by INF1 on TRV:*GUS*- and TRV:*NbVIII.2*-treated plants. Pictures were taken at three and six days after syringe-infiltration. Each experiment consisted of at least six infiltration sites. This experiment was repeated twice with similar results.
