# Supplementary material for: L-type lectin receptor kinases in Nicotiana benthamiana and tomato and their role in Phytophthora resistance
Source: J Exp Bot. 2015 Aug 5;66(21):6731–43. doi: 10.1093/jxb/erv379 (PMC4623685; doi:10.1093/jxb/erv379)
Supplement: Supplementary Data [file supp_erv379_Supplemental_File_S1.pdf]

Supplementary File S1.

LecRK sequences of *N. benthamiana* and tomato. Highlighted protein sequences were shown to be inconsistent with RNA-seq data. Revised sequences are labelled 'Corrected'. Introns within genomic sequences are underlined.

N. benthamiana LecRK sequences

>NbS00015931g0001.1   Genbank ID: KT225303
MFFKILALIMAAVFGLVTSEDLGFTYNGFNKRANLTIDGIAQLTPRGLLQLTNTSRLQKGHAFY
PTPINFKNLPNGSNFSFSTTFVFAIVPSVLPGHGMAFVIAPVGGLVQTLPSPFLGLFNDNTTG
EATNHIFAVEFDTLQNREFNDIDGNHVGIDINELKSVDSKPAGYYASNSVKLNNMTLASGQP
MQAWVDYNGMAKQINVTLAPMNMAKPNVPLLSISCDLSPILNRTMFIGFSGSTGSVVSTQYV
LGWSFRMNGIAQGLDLARLPKLPRVGPKKPSKLLLIALPLISAVVVVIAFSVLIYYVGRKRKFAE
LLEDWELEYGPHRFKYKDLYIATKGFANKELLGCGGFGRVYRGILPNSALEIAVKRVSHESKQ
GLREFVAEIVSIGRLRHRNLVPFLGYCRRKGELLLVYECMPNGSLDKFLYDKPRCALKWNQR
FRVIKGVASALVYLHEEWEQVVIHRDVKASNVLLDSELNAKLGDFGLARLYDHGSDPLTTHVI
GTVGYLAPEQTRTGKGTTCSDVYAFGAFLLEVACGRRPIDPRVSDENIVLVDYVFSCWAKG
DILEAIDQNLGSDYVKEEVELVLKLGLVCLQTQPTARPSMRQVLLYLEDDLPLPEISLMQTSST
SLTLAGFDHFPLSYPTSADQPLSWPTSVTNSILSNGR

>NbS00015931g0001.1   Genbank ID: KT225303
ATGTTTTTCAAGATCTTGGCCTTGATTATGGCTGCTGTTTTTGGTCTTGTAACCTCTGAAG
ATCTTGGATTTACTTACAATGGCTTCAATAAAAGAGCAAATTTAACTATTGATGGCATAGC
ACAGTTGACACCAAGAGGCTTATTACAGCTAACAAATACTTCTCGGCTGCAGAAAGGCCA
TGCTTTTTACCCTACACCAATAAATTTCAAGAACTTACCAAATGGCTCAAATTTCTCTTTTT
CTACAACTTTTGTCTTTGCTATAGTGCCTTCAGTTTTGCCTGGTCATGGCATGGCTTTTGT
AATTGCACCTGTCGGAGGATTAGTACAAACACTTCCAAGCCCATTTCTAGGCTTATTTAAT
GATAACACTACTGGTGAAGCCACAAATCATATTTTCGCGGTGGAGTTTGATACCCTTCAG
AACAGAGAATTCAATGATATTGATGGTAACCATGTTGGAATTGACATCAACGAATTAAAAT
CTGTTGATTCAAAGCCAGCAGGTTATTATGCTAGCAATAGTGTCAAACTCAATAACATGA
CTCTTGCTAGTGGCCAACCAATGCAAGCTTGGGTGGACTATAATGGTATGGCAAAGCAA
ATTAATGTCACATTGGCTCCAATGAATATGGCAAAACCAAATGTTCCTCTTTTGTCTATAT
CCTGTGATCTTTCACCAATTTTAAATAGAACCATGTTTATTGGCTTCTCTGGATCCACAGG
CTCAGTTGTTTCAACACAATATGTTCTTGGATGGAGCTTTAGAATGAATGGAATAGCTCAA
GGGCTTGATCTTGCTAGGCTTCCTAAGCTTCCTCGAGTTGGACCGAAGAAACCGTCCAA
ACTCTTGTTGATTGCTTTGCCATTGATATCAGCAGTTGTTGTAGTAATAGCCTTCTCTGTG
TTAATTTATTATGTAGGAAGGAAAAGGAAGTTTGCTGAATTGCTTGAAGATTGGGAGCTT
GAATATGGCCCTCATAGGTTCAAGTATAAAGATCTTTATATTGCCACCAAAGGATTTGCAA
ACAAAGAGTTGTTGGGTTGTGGGGGTTTTGGCAGAGTTTATAGAGGCATATTACCTAATT
CTGCTCTTGAGATAGCAGTCAAGAGGGTATCTCATGAATCAAAACAAGGGTTGAGGGAA
TTTGTAGCAGAAATTGTTAGTATTGGTCGACTACGCCATAGAAACTTAGTACCATTTTTAG
GCTATTGTAGGAGAAAAGGAGAGTTGCTTTTGGTTTATGAATGTATGCCTAATGGAAGTC
TTGATAAGTTTCTATATGACAAACCAAGATGTGCTCTCAAGTGGAACCAAAGATTTCGTGT
CATTAAAGGCGTAGCATCAGCGCTAGTCTATCTGCACGAAGAATGGGAACAAGTTGTAAT
TCACAGAGATGTAAAGGCAAGTAATGTATTGTTAGACAGTGAGTTAAATGCCAAGTTGGG
GGATTTTGGCTTAGCAAGATTGTATGATCATGGGAGCGATCCGCTCACTACACACGTAAT
TGGAACTGTAGGATATCTTGCCCCTGAACAAACTAGAACTGGAAAGGGTACAACATGCA
GTGATGTATATGCTTTTGGTGCATTTTTGCTTGAAGTGGCTTGTGGGAGAAGGCCAATAG
ATCCACGAGTATCGGACGAGAACATAGTTTTGGTTGACTATGTATTTTCTTGTTGGGCTA
AAGGTGATATTCTTGAAGCCATTGATCAAAATTTGGGAAGTGATTATGTTAAAGAAGAGG
TTGAGTTGGTATTAAAACTTGGATTAGTTTGTTTACAGACACAACCAACAGCTAGACCAA
GTATGAGGCAAGTGTTGCTATATTTAGAAGATGATCTGCCTTTGCCAGAAATATCACTGA
TGCAAACTTCAAGTACAAGCTTAACCTTAGCAGGGTTTGATCATTTTCCATTGTCATATCC
AACTTCTGCTGATCAGCCACTTTCTTGGCCTACTTCTGTTACAAACTCCATTCTCTCCAAC
GGTCGATGA

>NbS00029393g0102.1 GenBank ID: KT225304
MFFNLVILVAFFLVDFAPTSCEVDGFIYNGFQSRNISLDGIAEFTSTGLLLLTNSNTQDQGHAF
YPNPIHFKNSPNGTAFSFSTTFVFAIRSDYGNLSGHGLVFVITPKRGVEGAFPSHHLGLFNST
NNGNSSNHVVGVEFDTIISKEFNDINDNHVGIDINGLDSVAVQPAGYFDETGLFHNLTLISGQP
MQVWVDYDGYTPHINVTLAPLYVTKPVMPLLVLKYDLSTILDQTMYVGFSSSTGSVPTHHYIL
GWSFKTNGKAEELSQLPNLPRLGRKEKSRFLTTGLPIISLVSLVIATSVVVYYIRRKKKFEELL
DDWELDYRPQRFRYKDLYTATKGFREKELLGVGGFGKVYKGVMPISKLVIAVKKISQESQQG
MKEFVAEIVSIGRIQHRNLVPLLGYCRRKGELLLVYEYMPNGSVDKYLFDQPRFTLDWNQRF
RVIRGVASGLFFLHEECDHVVVHRDIKASNVLLDGELNGRLGDFGLARLYGHGTDPQSTRVV
GTLGYLAPEYARTGRATPSSDVFSFGAFLLEVACGRRPIEPRQDSDDLILVDWVFFCWNRG
NILQVVDPNIGIDFVQGQVELVLKLGLLCSHSEPSFRPTMRQILLFLDGVVALPELSALGISSA
GLTFDHRDGFDDFVKSYPSSLGNAHSRGLSVTDSLLSEGR

>NbS00029393g0102.1 GenBank ID: KT225304
ATGTTTTTCAATCTTGTCATACTAGTGGCTTTCTTCTTAGTTGATTTTGCACCAACTTCTTG
TGAAGTTGATGGATTCATTTACAATGGATTCCAATCAAGGAATATTAGCTTGGATGGCATA
GCTGAATTCACATCAACTGGCCTTTTGCTATTAACAAATTCTAACACACAAGATCAAGGC
CATGCTTTTTATCCAAATCCAATTCATTTCAAGAATTCACCAAATGGTACTGCATTTTCTTT
CTCCACAACTTTTGTGTTTGCCATAAGGTCTGATTATGGAAATTTGAGTGGTCATGGACT
GGTTTTCGTTATCACGCCAAAAAGAGGAGTTGAGGGGGCTTTTCCTAGCCACCATCTTG
GCCTTTTTAACTCAACCAATAATGGAAATAGTTCAAACCATGTTGTTGGAGTTGAATTTGA
TACAATCATAAGCAAGGAATTCAATGACATAAATGACAATCATGTTGGAATTGATATCAAT
GGATTGGATTCTGTAGCAGTTCAACCAGCAGGTTATTTTGATGAAACTGGTTTGTTTCATA
ACTTGACTCTTATTAGTGGCCAGCCAATGCAAGTTTGGGTGGATTATGATGGCTACACTC
CGCATATTAATGTAACATTAGCTCCATTATATGTGACAAAACCAGTTATGCCACTCTTGGT
TTTGAAGTATGATCTTTCAACAATTCTTGATCAGACTATGTATGTTGGTTTTTCATCATCAA
CTGGTTCAGTCCCAACACATCATTATATCTTGGGATGGAGTTTCAAGACAAATGGAAAAG
CTGAAGAACTTTCTCAACTTCCCAATCTTCCTCGTCTCGGGCGCAAAGAGAAATCAAGAT
TTTTAACAACTGGTTTGCCAATAATCTCTTTGGTATCTTTAGTTATAGCAACCTCAGTAGT
GGTGTATTACATAAGAAGGAAGAAGAAATTCGAAGAACTTCTTGATGATTGGGAACTTGA
CTATAGACCACAAAGGTTCAGGTATAAAGATTTGTACACTGCTACTAAGGGATTCAGAGA
AAAGGAACTATTGGGAGTTGGAGGATTTGGTAAAGTTTACAAAGGAGTGATGCCTATTTC
GAAACTTGTGATAGCAGTAAAGAAGATATCTCAAGAATCACAACAAGGAATGAAGGAATT
TGTTGCAGAGATTGTAAGCATCGGTCGTATACAACACAGGAATTTAGTACCACTTTTAGG
ATATTGCAGGAGAAAAGGAGAGTTGCTTTTGGTTTATGAATACATGCCTAATGGAAGTGT
AGACAAGTACTTATTTGATCAACCAAGATTTACCCTCGATTGGAACCAACGGTTCAGAGT
CATTAGAGGCGTTGCATCAGGACTGTTCTTCCTACACGAAGAATGTGACCACGTAGTTGT
TCATCGAGATATTAAGGCTAGTAATGTCTTGTTGGATGGTGAACTAAATGGAAGATTAGG
AGACTTTGGCCTCGCGAGGCTATATGGTCATGGAACCGATCCTCAGTCTACTCGCGTCG
TCGGTACTCTTGGTTACCTTGCACCAGAGTATGCTAGAACTGGTAGAGCAACACCTAGTA
GTGATGTATTTTCTTTTGGTGCTTTTTTGCTTGAAGTTGCCTGTGGTAGGAGGCCGATAG
AGCCAAGACAAGACAGTGATGATCTGATTTTGGTCGATTGGGTGTTCTTTTGCTGGAATA
GAGGTAATATTCTTCAGGTTGTTGATCCAAACATAGGCATTGATTTTGTTCAAGGGCAAG
TGGAGTTGGTCTTAAAGTTAGGCTTGTTGTGCTCTCATTCAGAGCCTTCGTTTAGGCCAA
CCATGCGACAAATCTTGTTGTTCTTGGATGGTGTTGTGGCATTACCAGAGTTATCAGCAC
TCGGCATTTCATCAGCTGGCCTAACATTTGATCATCGTGATGGTTTTGATGATTTTGTCAA
GTCATATCCGTCATCTTTGGGTAATGCACATTCCCGCGGTCTATCAGTAACTGACTCTCT
TCTCTCTGAAGGCCGATGA

>NbS00010453g0003.1
MDKPYCLVIILAFICTILVRAHSFDFLYNGFNKSDIMTDGVAYINPSGALKLTNRSYHVIGHAFH
PKPIPFFNSSTNSTAKNASSFSTNFVFAIVPLKNTPGGFGFAFTLSPSPSFPGAQGDHFLGVL
NSKNDGNATNHIFLVEFDTVNGHNEGVDKDGNHIGININGMRSIASNSADYHINDTSETEQIY
LQGGDRIQAWIDYDGVKKVVNVTVAPISIPKPIQPLISESIDLSPVMKETMYAGFSAATGDKAS
SHYILGWSFRLNGAADPLNPSELPTAPPEVVTSSKNSHLKKALIAAISSMVFLIIVASVIVYIRRR
MVQHEVLEDWELDCPHRFRYRDLYKAAKGFKVSELIGVGGFGAVYKGILPTNGAEVAVKRIA
SNSLQGMREFAAEIESLGRLRHKHLVNLQGWCKTKNDLLLVYDYIPNGSLDSLLYRPKNDIVL
AWDRRFNIIKGIAAGLLYLHEEWEQVVIHRDVKSSNVLIDGEMNGKLGDFGLARLYDHGKNS
HTTNVVGTIGYIAPELSRTGKASTSTDIYAYGVLLLEVASGRPPIIYEPGQGALVLADWVIECL
QLGNILDAVDPKLNSAYVDKEVRMGALVLADWVIECLQLGNILDAVDPRLNSAYVDKEVKMIL

GLGLLCSHPRPEARPTMRQVMRYLNGDESLQISEQLSSVGSGRVDEITSKFLDVFASDTISIS
RRTFSIGQMSSSSLNAGR

>NbS00010453g0003.1 Corrected; GenBank ID: KT225305
MDKPYCLVIILAFICTILVRAHSFDFLYNGFNKSDIMTDGVAYINPSGALKLTNRSYHVIGHAFH
PKPIPFFNSSTNSTAKNASSFSTNFVFAIVPLKNTPGGFGFAFTLSPSPSFPGAQGDHFLGVL
NSKNDGNATNHIFLVEFDTVNGHNEGVDKDGNHIGININGMRSIASNSADYHINDTSETEQIY
LQGGDRIQAWIDYDGVKKVVNVTVAPISIPKPIQPLISESIDLSPVMKETMYAGFSAATGDKAS
SHYILGWSFRLNGAADPLNPSELPTAPPEVVTSSKNSHLKKALIAAISSMVFLIIVASVIVYIRRR
MVQHEVLEDWELDCPHRFRYRDLYKAAKGFKVSELIGVGGFGAVYKGILPTNGAEVAVKRIA
SNSLQGMREFAAEIESLGRLRHKHLVNLQGWCKTKNDLLLVYDYIPNGSLDSLLYRPKNDIVL
AWDRRFNIIKGIAAGLLYLHEEWEQVVIHRDVKSSNVLIDGEMNGKLGDFGLARLYDHGKHS
HTTNVVGTIGYLAPELSRTGKASTSTDIYAYGVLLLEVASGRPPIIYEPGQGALVLADWVIECL
QLGNILDAVDPKLNSAYVDKEVRMVLGLGLLCSHPRPEARPTMRQVMRYLNGDESLQISEQ
LSSVGSGRVDEITSKFLDVFASDTISISRRTFSIGQMSSSSLNAGR

>NbS00010453g0003.1 Corrected; GenBank ID: KT225305
ATGGACAAGCCCTATTGCCTTGTTATTATTCTTGCATTCATTTGCACTATTCTTGTAAGAG
CTCATTCTTTTGATTTTCTTTATAATGGATTCAATAAGTCAGATATCATGACTGATGGAGTT
GCATATATCAATCCTAGTGGTGCACTTAAGCTTACTAATAGATCATACCATGTTATAGGAC
ATGCTTTCCATCCCAAACCTATACCATTTTTCAACTCTAGTACTAATTCCACTGCTAAAAAT
GCTTCTTCATTTAGTACAAACTTTGTCTTCGCCATTGTCCCTCTTAAGAACACTCCAGGTG
GCTTTGGCTTTGCTTTCACCTTATCTCCATCCCCGAGTTTTCCTGGTGCTCAAGGCGATC
ACTTTCTTGGAGTCCTCAATTCAAAGAACGATGGCAATGCTACTAACCACATCTTTTTGGT
CGAATTTGACACTGTGAATGGTCACAATGAAGGTGTGGACAAAGATGGCAACCACATTG
GAATCAACATCAATGGCATGAGGTCTATCGCATCAAACTCAGCCGATTACCATATTAATG
ACACTTCTGAAACAGAACAGATATATCTACAAGGTGGAGACCGGATTCAGGCTTGGATA
GATTACGATGGAGTAAAGAAAGTGGTAAATGTAACTGTGGCTCCAATATCAATTCCAAAG
CCAATTCAACCTCTAATTTCTGAGTCCATAGACTTGTCCCCAGTGATGAAGGAAACTATG
TATGCTGGTTTCTCAGCAGCCACAGGAGATAAAGCAAGCTCTCATTACATCTTGGGTTGG
AGTTTTCGGTTGAATGGAGCTGCTGATCCATTAAATCCCTCTGAGCTACCTACTGCCCCA
CCTGAAGTGGTAACATCCTCCAAGAATTCTCATCTTAAAAAGGCCTTGATCGCAGCAATT
TCCTCCATGGTTTTCCTGATCATTGTGGCATCAGTAATTGTTTATATCCGCAGAAGAATG
GTGCAACATGAAGTGCTAGAGGATTGGGAGCTGGATTGTCCTCACAGGTTCAGATACAG
AGATCTTTACAAGGCAGCCAAGGGATTCAAGGTCAGCGAACTAATTGGAGTTGGAGGCT
TCGGTGCTGTTTACAAAGGTATCTTGCCCACTAATGGAGCTGAGGTTGCAGTGAAGAGG
ATAGCAAGCAATTCTCTTCAGGGAATGAGAGAATTTGCTGCGGAGATTGAAAGCTTAGG
CAGGTTAAGGCACAAACACTTGGTCAACCTTCAAGGCTGGTGTAAGACAAAGAATGATCT
TCTCCTAGTGTATGACTATATCCCAAATGGAAGTCTTGATTCATTACTTTATAGACCAAAA
AATGACATTGTTCTAGCATGGGACCGGAGATTTAACATCATCAAAGGGATTGCTGCAGG
GCTTCTTTACTTGCACGAAGAATGGGAGCAAGTGGTAATACATCGAGACGTGAAGAGCA
GTAACGTCCTTATTGATGGTGAAATGAATGGTAAATTAGGGGATTTTGGGCTTGCAAGAT
TATATGATCATGGCAAGCATTCACACACAACAAATGTTGTAGGAACAATAGGGTACCTTG
CACCCGAATTATCACGAACAGGGAAGGCCTCAACGAGCACAGATATTTATGCGTATGGT
GTACTACTTCTTGAAGTAGCTAGTGGAAGACCACCTATTATCTATGAACCAGGGCAAGGA
GCTCTGGTACTAGCAGATTGGGTGATAGAATGTCTTCAACTAGGTAATATTCTTGATGCA
GTTGATCCTAAGTTGAATTCTGCCTATGTCGATAAAGAGGTGAGAATGGTTTTGGGACTT
GGGCTTCTTTGTTCTCACCCAAGACCAGAAGCTAGGCCAACCATGAGACAAGTAATGAG
GTACCTCAATGGAGATGAATCGCTTCAAATTTCTGAGCAATTGAGCTCTGTTGGATCGGG
CAGGGTTGATGAAATCACATCCAAGTTCTTAGATGTGTTTGCTAGTGATACAATCAGTATA
TCACGACGTACATTTTCCATCGGACAGATGTCATCTAGTTCCCTAAATGCTGGTAGATAG

>NbS00005128g0014.1
MDKPYCLVILVFFYTILVRAHSFGFIYNGFNKSDIMTDGVAYINPSGALKLTNRSYHVIGHAFH
PKPIPIFNSSTNSTAKNASSFSTNFVFAIVPLKNTPGGFGFAFTLSPSPSFPGAQGDHFLGVLN
STNDGNDTNHIFLVEFDTVNGYNEGVDTDGNHIGININGMKSIASQPAYYYVNDTSKTEEVNL
EREESIQAWVDYDGVNKVVNVTVSPKSIPKPIQPLISKSIDLSSVMKETMYAGFSAATGDKAS
SHYILGWSFRLNGAADPLNPSELPTAPPEVVSELIGVGGFGAVYKGILPTNGAEVSELIGVGG
FGAVYKGILPTNGAEVAVKRIASNSLQGMREFAAEIESLGRLRHKHLVNLQGWCKTKNDLLL
VYDYIPNGSLDSLLYRPKNDIVLAWDRRFNIVPNGSLDSLLYRPKNDIVLAWDRRFNIIKGIAA

GLLYLHEEWDQVVIHRDVKSSNVLIDGEMNGKLGDFGLARLYDHGKHSHTTNVVGTIGYLAP
ELSRTGKASTSTDVYAYGVLLLEVASGRPPIIYEPGQGALVLADWVIECLQLGNILDAVDPRL
NSAYVDKEVKMILGLGLLCSHPRPEARPTMRQVMKYLNGDESLQISEQLSSVGSGRVDEITS
KFLEVFASDTINISRHTFSIGQMSSSSLNADRSSISGAFII

>NbS00005128g0014.1 Corrected; GenBank ID: KT225306
MDKPYCLVILVFFYTILVRAHSFGFIYNGFNKSDIMTDGVAYINPSGALKLTNRSYHVIGHAFH
PKPIPIFNSSTNSTAKNASSFSTNFVFAIVPLKNTPGGFGFAFTLSPSPSFPGAQGDHFLGVLN
STNDGNDTNHIFLVEFDTVNGYNEGVDTDGNHIGININGMKSIASQPAYYYVNDTSKTEEVNL
EREESIQAWVDYDGVNKVVNVTVSPKSIPKPIQPLISKSIDLSSVMKETMYAGFSAATGDKAS
SHYILGWSFRLNGAADPLNPSELPTAPPEVVTSSKNSYLKKALIAAFSSTVFLIIVVSVIVHIRR
RMVQHEVLEDWELDCPHRFRYRDLYKATRGFKVSELIGVGGFGAVYKGILPTNGAEVAVKRI
ASNSLQGMREFAAEIESLGRLRHKHLVNLQGWCKTKNDLLLVYDYVPNGSLDSLLYRPKNDI
VLAWDRRFNIIKGIAAGLLYLHEEWDQVVIHRDVKSSNVLIDGEMNGKLGDFGLARLYDHGK
HSHTTNVVGTIGYLAPELSRTGKASTSTDVYAYGVLLLEVASGRPPIIYEPGQGALVLADWVI
ECLQLGNILDAVDPRLNSAYVDKEVKMILGLGLLCSHPRPEARPTMRQVMKYLNGDESLQIS
EQLSSVGSGRVDEITSKFLEVFASDTINISRHTFSIGQMSSSSLNAGR

>NbS00005128g0014.1 Corrected; GenBank ID: KT225306
ATGGACAAGCCCTATTGCCTTGTTATTCTTGTATTCTTTTACACTATTCTTGTAAGAGCTC
ATTCTTTTGGTTTTATTTACAATGGATTCAATAAGTCGGATATCATGACTGATGGAGTTGC
ATATATCAATCCTAGTGGTGCACTTAAGCTTACCAATAGATCATACCATGTTATAGGCCAT
GCTTTCCACCCCAAACCTATACCAATTTTCAACTCTAGTACTAATTCCACTGCTAAAAATG
CTTCTTCATTTAGTACAAACTTCGTCTTCGCCATTGTCCCTCTTAAGAACACTCCAGGTGG
CTTTGGCTTTGCTTTCACCTTATCTCCATCCCCGAGTTTTCCTGGTGCTCAGGGCGATCA
CTTTCTTGGAGTCCTCAATTCAACGAACGATGGCAATGATACTAACCACATCTTTTTGGTT
GAATTTGACACAGTGAATGGCTACAATGAAGGTGTGGACACAGATGGCAATCACATTGG
AATCAACATCAATGGCATGAAGTCTATCGCATCACAACCAGCCTATTACTATGTCAATGA
CACTTCTAAAACAGAGGAGGTAAATCTAGAAAGAGAAGAATCAATTCAGGCTTGGGTAGA
TTATGATGGAGTAAACAAAGTGGTAAATGTAACAGTATCCCCCAAATCAATTCCAAAGCC
AATTCAACCTCTAATCTCTAAGTCCATAGACTTGTCCTCAGTGATGAAGGAAACTATGTAT
GCTGGCTTCTCAGCAGCCACAGGAGATAAAGCAAGCTCGCATTACATCTTGGGTTGGAG
TTTTCGGTTGAATGGAGCTGCTGATCCATTAAATCCCTCTGAGCTACCTACTGCCCCACC
TGAAGTGGTAACATCCTCCAAGAATTCCTATCTTAAAAAGGCCTTGATCGCAGCATTTTC
CTCCACGGTTTTCCTGATCATTGTGGTATCAGTAATTGTTCATATCCGCAGAAGAATGGT
GCAACATGAAGTTCTAGAGGATTGGGAGCTGGATTGTCCTCACAGGTTTAGATACCGAG
ATCTTTACAAGGCAACCAGGGGATTCAAGGTCAGTGAACTAATTGGAGTTGGAGGCTTT
GGTGCTGTTTACAAGGGTATTTTGCCCACTAATGGAGCTGAGGTTGCAGTGAAGAGGAT
AGCGAGCAATTCTCTTCAAGGAATGAGAGAATTTGCAGCGGAGATTGAAAGCTTAGGCA
GGTTAAGGCACAAACACTTGGTCAACCTTCAAGGCTGGTGTAAGACAAAGAATGATCTTC
TCCTAGTGTATGACTATGTCCCAAATGGAAGTCTTGATTCGTTACTTTATAGACCGAAAAA
CGACATTGTTCTAGCATGGGACCGGAGATTTAACATCATTAAAGGGATTGCTGCAGGGC
TTCTTTACTTGCACGAAGAATGGGATCAAGTGGTAATACATCGAGACGTGAAGAGCAGTA
ACGTCCTTATTGATGGTGAAATGAATGGTAAATTAGGGGATTTTGGGCTTGCAAGATTAT
ATGATCATGGCAAGCATTCACACACAACAAATGTTGTAGGAACAATAGGGTACCTTGCAC
CCGAATTATCACGAACAGGGAAGGCCTCAACGAGCACAGATGTTTATGCATATGGGGTA
CTACTACTTGAAGTAGCTAGTGGGAGACCACCTATTATCTATGAACCAGGGCAAGGAGC
TCTGGTTCTAGCAGATTGGGTTATAGAATGTCTTCAACTAGGTAATATTCTTGATGCAGTT
GATCCTAGGTTGAATTCTGCCTATGTCGATAAAGAGGTGAAAATGATTTTGGGACTTGGG
CTTCTTTGTTCTCACCCAAGACCAGAAGCTAGGCCAACCATGAGACAAGTAATGAAGTAC
CTCAATGGAGATGAATCGCTTCAAATTTCTGAGCAGTTGAGCTCTGTTGGGTCGGGCAG
GGTTGATGAAATCACATCCAAGTTCTTGGAAGTGTTTGCTAGTGATACAATCAATATATCA
CGCCATACATTTTCCATCGGACAGATGTCATCTAGTTCCCTAAATGCTGGTAGATAG

>NbS00024573g0008.1
MNQALETLLLYLLITIIFSSVQSVSAIDFVFNGFKPSDLSLFGIATIESGILTLTNDSTFSIGRALH
PPKIVTKAPNSAQVLPFSTSFIFAMAPFKDRLPGHGIVFLFVPQTGIDGTTSSQNLGFLNFTNN
GNPDNHVFGVEFDVFKNQEFNDINDNHVGIDVNSLASVFAHEAGYWPDKYSKFSDDGNLNE
ESFETLKLNNGRNYQVWIDYADFQINVFKEEMYVGFTASTGDLAQVSLFLIKRNRRMKRERE
EMEDWELEYWPHRISYQEIDAATKGFADENVIGIGGNGKVYKGVLAGSSEVAVKRISHESSE

GARQFLAEISSLGRLKHRNLVSLRGWCKKDRHSLILVYDYMENGSLDKRLFECDETNMLSFE
DRIRILKDVASGVLYLHEGWEAKVLHRDIKASNVLLDKDMNARLGDFGLARMHDHGQVANTT
RVVGTVGYLAPEFVKTGRASTQTDVFGYGVLVLEVMCGRRPIEEDGKPPLLDWLWELMRR
GECDVWKEAYRGRRRAPFIGLAVGTNETRQID

>NbS00024573g0008.1 Corrected; GenBank ID: KT225307
MNQALETLLLYLLITIIFSSVQSVSAIDFVFNGFKPSDLSLFGIATIESGILTLTNDSTFSIGRALH
PPKIVTKAPNSAQVLPFSTSFIFAMAPFKDRLPGHGIVFLFVPQTGIDGTTSSQNLGFLNFTNN
GNPDNHVFGVEFDVFKNQEFNDINDNHVGIDVNSLASVFAHEAGYWPDKYSKFSDDGNLNE
ESFETLKLNNGRNYQVWIDYADFQINVTTAPVGMKRPKQPLLDFPLNLSQVFKEEMYVGFTA
STGDLAQGHKILAWSFSNSNFRISDALITQGLPSFELPKDPVHRSKGFIAGMTVSLLFLVVVTV
VVSLFLIKRNRRMKREREEMEDWELEYWPHRISYQEIDAATKGFADENVIGIGGNGKVYKGV
LTGSLEVAVKRISHESSEGARQFLAEISSLGRLKHRNLVSLRGWCKKDRHSLILVYDYMENG
SLDKRLFECDETNMLSFEDRIRILKDVASGVLYLHEGWEAKVLHRDIKASNVLLDKDMNARLG
DFGLARMHDHGQVANTTRVVGTVGYLAPEFVKTGRASTQTDVFGYGVLVLEVMCGRRPIEE
DGKPPLLDWLWELMRRGELINAFDRRLRTSQDFNEEEALRVLQLGMICASMDPKARPTMRQ
VVKFFERNSEADESEAEDMDVYLLETLRSNTMLSNFSLSLSHGSHPTFEEIREGLSSSMSIS
WTNSLVDGR

>NbS00024573g0008.1 Corrected; GenBank ID: KT225307
ATGAACCAAGCCCTAGAAACTCTTCTTCTCTACCTGCTGATAACCATAATATTTTCTAGTG
TTCAATCAGTTTCAGCTATTGATTTTGTGTTTAATGGCTTTAAACCATCAGATTTATCACTG
TTTGGGATTGCTACAATTGAATCTGGAATACTTACTCTTACAAATGACTCAACTTTCTCAA
TTGGCAGGGCTCTACACCCTCCAAAGATTGTCACAAAAGCACCAAATTCAGCACAAGTTC
TTCCCTTTTCAACATCTTTCATTTTTGCAATGGCTCCTTTTAAGGATAGGCTACCAGGACA
TGGCATAGTTTTCTTGTTTGTGCCACAAACAGGTATCGATGGTACTACTTCTTCACAAAAT
TTAGGCTTTTTGAACTTCACAAATAATGGGAATCCTGATAATCATGTTTTTGGGGTTGAAT
TTGATGTGTTCAAGAATCAAGAATTCAATGACATAAATGACAACCATGTTGGAATTGATGT
TAATTCTCTTGCATCAGTGTTTGCTCATGAAGCAGGCTATTGGCCTGATAAGTACAGCAA
GTTTAGCGATGATGGTAACTTAAATGAAGAGTCTTTCGAGACTTTAAAGTTGAATAATGGA
AGAAATTACCAAGTTTGGATTGACTATGCAGATTTTCAGATTAATGTGACTACGGCACCA
GTTGGTATGAAAAGGCCTAAGCAACCTTTGTTGGATTTTCCCCTCAATCTTTCTCAGGTTT
TTAAGGAAGAGATGTATGTGGGGTTCACTGCATCCACTGGAGATCTTGCTCAAGGTCAC
AAAATTTTAGCTTGGAGTTTTAGTAACTCGAATTTTCGGATAAGTGATGCTTTAATCACAC
AGGGTTTGCCTTCATTTGAGCTGCCTAAAGATCCAGTTCATCGATCAAAAGGGTTCATTG
CAGGTATGACAGTGTCACTCTTGTTTCTTGTTGTGGTCACTGTTGTAGTTTCATTGTTTCT
GATTAAGAGAAATAGGAGAATGAAAAGGGAAAGAGAGGAAATGGAAGATTGGGAATTGG
AATATTGGCCACATAGGATAAGTTATCAGGAAATTGATGCTGCCACAAAGGGTTTTGCTG
ATGAAAATGTGATTGGAATTGGAGGTAATGGGAAGGTATATAAAGGTGTTTTGACTGGGA
GTTTAGAGGTTGCAGTAAAGCGCATTTCTCATGAAAGCAGTGAAGGGGCAAGACAATTC
TTGGCTGAGATTTCAAGTCTCGGTAGGCTAAAGCACAGAAATTTGGTGTCACTAAGAGG
CTGGTGCAAGAAAGACCGACACAGCTTGATTTTGGTTTATGATTATATGGAAAATGGGAG
TTTGGATAAAAGGCTGTTTGAATGTGATGAGACGAACATGTTGAGTTTTGAAGATAGAAT
TAGGATTTTGAAAGATGTGGCATCAGGAGTTCTATACTTGCATGAGGGATGGGAGGCAA
AAGTGTTACATAGGGACATTAAGGCTAGCAATGTTTTACTTGACAAGGACATGAATGCAA
GACTAGGTGATTTTGGTCTAGCCAGAATGCATGATCATGGTCAAGTGGCTAACACGACT
CGAGTTGTTGGCACAGTAGGTTACCTGGCACCAGAGTTTGTCAAGACTGGTCGTGCCTC
TACACAAACTGATGTGTTTGGATATGGAGTTTTAGTTTTGGAGGTGATGTGTGGAAGGAG
GCCTATAGAGGAGGATGGCAAGCCCCCTTTATTGGATTGGCTGTGGGAACTAATGAGAC
GAGGCGAATTGATTAATGCCTTTGACCGTCGATTAAGGACGAGTCAAGATTTCAATGAAG
AGGAAGCATTAAGAGTATTGCAATTAGGCATGATATGCGCGAGCATGGACCCCAAAGCT
AGGCCAACTATGAGACAAGTAGTGAAATTCTTTGAAAGGAACAGTGAGGCTGATGAATCT
GAAGCTGAGGATATGGATGTTTACCTTCTGGAGACTTTGAGATCCAATACCATGTTGTCC
AATTTTTCCTTGAGTTTGAGCCATGGTTCACACCCAACATTTGAAGAAATTAGAGAAGGTT
TGTCTTCTTCCATGTCCATTTCTTGGACAAATTCTTTGGTGGATGGTAGGTGA

>NbS00025337g0001.1 GenBank ID: KT225308
MNLKTLLLYLLITIFSCIQSVSAIDFVFNGFKSSDISLFGIATIESRILTLTNDSTFSIGRALHPSKIV
TKAPNSSQVLPFSTSFIFAMAPFKDRLPGHGIVFLFVPQTGIDGTTSSQNLGFLNFTNNGNPD
NHVFGVEFDVFKNQEFNDINDNHVGIDVNSLASVFDHEAGYWPDKYNKFSDDGSLNEESFE

TLKLNNGRNYQVWIDYADFQINVTMAPVGMKRPKQPLLDFPLNLSQVFKEEMYVGFTASTG
DLAQGHKILAWSFSNSNFRISDALITQGLPSFELPKDPVHRSKGFIAGMTVSLLFLVVVTVVVS
LFLIKRNRRMKREREEMEDWELEYWPHRISYQEIDAATKGFADENVIGIGGNGKVYKGVLTG
SLEVAVKRISHESSEGARQFLAEISSLGRLKHRNLVSLRGWCKKDRRSLILVYDYMENGSLD
KRLFECDETNMLSFEDRIRILKDVASGVLYLHEGWEAKVLHRDIKASNVLLDKDMNARLGDF
GLARMHDHGQVANTTRVVGTVGYLAPEFVKTGRASTQTDVFGYGVLVLEVMCGRRPIEED
GKPPLLDWLWELMRRGELINAFDRRLRTSQDFNEEEALRVLQLGMICASLDPKGRPTMRQV
VKFFERNSEADESEAEHMDVYLLETLRSNTMLSNFSLSLSHGSHPTFEEIREGLSSSMSISW
TNSLVDGR

>NbS00025337g0001.1 GenBank ID: KT225308
ATGAACCTAAAAACTCTTCTTCTCTACCTGCTGATAACCATATTTAGTTGTATTCAATCAGT
TTCAGCTATTGATTTTGTCTTCAATGGCTTTAAATCATCAGATATATCACTGTTTGGGATT
GCTACAATTGAATCTCGAATACTTACTCTTACCAATGACTCAACTTTCTCAATTGGCAGAG
CTCTACACCCTTCCAAGATTGTCACGAAAGCACCAAATTCATCCCAAGTTCTTCCCTTTTC
AACATCTTTCATTTTTGCAATGGCTCCTTTTAAGGACAGGTTACCAGGACATGGCATAGTT
TTCTTGTTTGTGCCACAAACAGGTATTGATGGTACTACTTCTTCACAAAATTTAGGTTTTTT
GAACTTCACAAATAATGGGAATCCTGATAATCATGTTTTTGGGGTTGAGTTTGATGTTTTC
AAGAATCAAGAATTCAATGACATAAATGACAACCATGTTGGAATTGATGTCAATTCTCTTG
CATCAGTGTTTGATCATGAAGCAGGCTATTGGCCTGATAAGTACAATAAGTTTAGTGATG
ATGGTAGTTTAAATGAAGAGTCTTTCGAGACTTTAAAGTTGAATAATGGAAGAAATTACCA
AGTTTGGATTGACTATGCAGATTTTCAGATTAATGTGACTATGGCACCAGTTGGTATGAA
AAGGCCTAAGCAACCTTTATTGGATTTTCCCCTCAATCTTTCTCAGGTTTTTAAGGAAGAG
ATGTATGTGGGGTTCACTGCATCCACTGGAGATCTTGCTCAAGGTCACAAAATTTTAGCT
TGGAGTTTTAGTAACTCGAATTTTCGGATAAGTGATGCTTTAATCACACAGGGTTTGCCTT
CATTTGAGCTGCCTAAAGATCCAGTTCATCGATCAAAAGGGTTCATTGCAGGTATGACAG
TGTCACTCTTGTTTCTTGTTGTGGTCACTGTTGTAGTTTCATTGTTTCTGATTAAGAGAAA
TAGGAGAATGAAAAGGGAAAGAGAGGAAATGGAAGATTGGGAATTGGAATATTGGCCAC
ATAGGATAAGTTATCAGGAAATTGATGCTGCCACAAAGGGTTTTGCTGATGAAAATGTGA
TTGGAATTGGAGGTAATGGGAAGGTATATAAAGGTGTTTTGACTGGGAGTTTAGAGGTTG
CAGTAAAGCGCATTTCTCATGAAAGCAGTGAAGGGGCAAGACAATTCTTGGCTGAGATTT
CAAGTCTTGGTAGGCTAAAGCATAGAAATTTGGTGTCATTAAGAGGCTGGTGCAAGAAA
GACCGACGCAGCCTGATTTTGGTTTATGATTATATGGAAAATGGGAGCTTGGATAAAAGA
CTATTTGAATGTGATGAGACAAACATGTTGAGTTTTGAAGACAGAATTAGGATTTTGAAAG
ATGTGGCATCAGGGGTTCTATATTTGCATGAGGGGTGGGAGGCAAAAGTGTTACATAGG
GACATTAAGGCTAGCAATGTTTTACTTGACAAGGACATGAATGCAAGACTAGGTGATTTT
GGTCTAGCCAGAATGCATGATCATGGTCAAGTGGCTAACACGACTCGAGTTGTTGGCAC
AGTAGGTTACCTGGCACCAGAGTTTGTCAAGACTGGTCGTGCCTCTACACAAACTGATG
TGTTTGGATATGGAGTTTTAGTTTTGGAGGTGATGTGTGGAAGGAGGCCTATAGAGGAG
GATGGCAAGCCCCCTTTATTGGATTGGCTGTGGGAACTAATGAGACGAGGCGAATTGAT
TAATGCCTTTGACCGTCGATTAAGGACGAGTCAAGATTTCAATGAAGAGGAAGCATTAAG
AGTATTGCAATTAGGCATGATATGCGCGAGCCTAGACCCCAAAGGTAGGCCAACCATGA
GACAAGTAGTGAAATTCTTTGAAAGGAACAGTGAGGCTGATGAATCTGAAGCTGAGCATA
TGGATGTTTACCTTCTGGAGACACTGAGATCTAATACCATGTTGTCCAATTTTTCCTTGAG
TTTGAGCCATGGTTCACACCCAACATTTGAAGAAATTAGAGAAGGTTTGTCTTCTTCCAT
GTCCATTTCTTGGACAAATTCTTTGGTGGATGGTAGGTGA

>NbS00032834g0007.1
MKQYLKTLLLYLLITILSSIQSASATDFVFNSFKPSDISAYGDATIESGILSLTLDVAYFSDGRAL
HPSKIVTKAPNSSQVLPFSTSFIFAMAPYRDRLPGHGMVFLFVPHTTGMSSSSSQNLGFLNF
TNNGNPDNHVFGVEFDVFRNQEFNDINNNHVGIDVNSLESVFVHEAGYWPDKYNKYNDNG
SLNEEYFETLKLNNGKNYQVWIDYADFHINVTMAPVGMKRPKQPLLDFPLNLSQVFKDEMYV
GFTASTGMLAQGHKILAWSFKFNDINNNHVGIDVNSLESVFVHEAGYWPDKYNKYNDNGSL
NEEYFETLKLNNGKNYQVWIDYADFHINVTMAPVGMKRPKQPLLDFPLNLSQVFKDEMYVG
FTASTGMLAQGHKILAWSFSNSNFSISDALITQGLPSFELPKDLIYRSKGFIVGMTMSLFFLVV
VTSVASLFLIKRNRRLKREKEDYLSRNDAATKGFADEIVIGVGGNGKVYKGVLAGGSEVAVK
RMSHDNCEGERQFLSEISSLGRLKHRNLVALRGWCKKGGGSLIVVYDYMENGSLDKKLFEC
DKGNMLSFEDRIKILKDVASGVLYLHEGWEAKVLHRDIKASNVLLDKEMHARIGDFGLARMH
DHGQVAKETRVVGTVGYLAPEFAKTGRASTQTDLFSYGVLILEVMCGRRPIEEGKPPLVDWL

WELMKRGELSNAFDNQLKSNKGFNEEEALRVLQLGMICASLDPRTRPTARQVVKFFERNSD
GAESEAEDMDAYLLESMRHNTMLTNYCSSMPISWTNSLVEGR

>NbS00032834g0007.1 Corrected; GenBank ID: KT225309
MKQYLKTLLLYLLITILSSIQSASATDFVFNSFKPSDISAYGDATIESGILSLTLDVAYFSDGRAL
HPSKIVTKAPNSSQVLPFSTSFIFAMAPYRDRLPGHGMVFLFVPHTTGMSSSSSQNLGFLNF
TNNGNPDNHVFGVEFDVFRNQEFNDINNNHVGIDVNSLESVFVHEAGYWPDKYNKYNDNG
SLNEEYFETLKLNNGKNYQVWIDYADFHINVTMAPVGMKRPKQPLLDFPLNLSQVFKDEMYV
GFTASTGMLAQGHKILAWSFSNSNFSISDALITQGLPSFELPKDLIYRSKGFIVGMTMSLFFLV
VVTSVASLFLIKRNRRLKREKEGIEDWELEYWPHRITYQEIDAATKGFADEIVIGVGGNGKVY
KGVLAGGSEVAVKRMSHDNCEGERQFLSEISSLGRLKHRNLVALRGWCKKGGGSLIVVYDY
MENGSLDKKLFECDKGNMLSFEDRIKILKDVASGVLYLHEGWEAKVLHRDIKASNVLLDKEM
HARIGDFGLARMHDHGQVAKETRVVGTVGYLAPEFAKTGRASTQTDLFSYGVLILEVMCGR
RPIEEGKPPLVDWLWELMKRGELSNAFDNQLKSNKGFNEEEALRVLQLGMICASLDPRTRP
TARQVVKFFERNSDGAESEAEDMDAYLLESMRHNTMLTNYCSSMPISWTNSLVEGR

>NbS00032834g0007.1 Corrected; GenBank ID: KT225309
ATGAAGCAATACCTAAAAACTCTTCTTCTCTACCTGCTAATAACCATACTTTCTAGTATTCA
ATCAGCTTCAGCAACTGATTTTGTCTTCAATAGCTTTAAACCATCAGATATATCAGCATAT
GGAGATGCTACAATTGAATCTGGAATACTTAGTCTTACCCTTGATGTAGCTTACTTTTCAG
ATGGTAGAGCTCTACACCCTTCAAAAATTGTCACTAAAGCACCCAATTCATCTCAAGTTCT
TCCCTTTTCAACATCTTTCATTTTTGCTATGGCGCCTTATAGGGACAGGCTACCAGGACA
TGGCATGGTTTTCTTGTTTGTGCCACACACCACAGGTATGTCGAGTTCTTCTTCACAGAA
TTTAGGTTTCTTGAACTTCACAAATAATGGGAATCCCGATAACCATGTCTTTGGGGTTGA
GTTTGATGTTTTCAGGAATCAAGAATTCAATGACATAAATAACAACCATGTTGGAATTGAT
GTCAATTCTCTTGAATCAGTGTTTGTTCATGAAGCAGGCTATTGGCCTGATAAGTACAATA
AGTATAATGATAATGGTAGCCTAAATGAAGAATATTTCGAGACTTTAAAGTTGAATAATGG
AAAAAACTACCAAGTTTGGATTGACTATGCAGATTTCCACATTAATGTTACTATGGCACCA
GTTGGTATGAAAAGGCCTAAGCAACCTTTGTTGGATTTCCCTCTGAATCTTTCTCAAGTTT
TTAAGGATGAGATGTATGTGGGGTTCACAGCCTCCACTGGAATGCTTGCTCAAGGTCAC
AAGATTTTAGCTTGGAGTTTTAGTAACTCTAATTTTTCGATTAGTGATGCTTTGATCACAC
AAGGTTTGCCGGTTAGAATTATGATTCCGACTAAACACAATAGCCTTCATATCGAATAATT
TGGAATAACAACATGAACGGAAGCATACCTGAATCCATATGTGGAAATCCTTAAGCCTCC
TTTTGATGATTGTTTGAAAGCTCCTTGATCCTTTCTTCAAGATTGTACTTCACAAATAATG
GGAATCCCGATAACCATGTCTTTGGGGTTGAGTTTGATGTTTTCAGGAATCAAGAATTCA
ATGACATAAATAACAACCATGTTGGAATTGATGTCAATTCTCTTGAATCAGTGTTTGTTCA
TGAAGCAGGCTATTGGCCTGATAAGTACAATAAGTATAATGATAATGGTAGCCTAAATGA
AGAATATTTCGAGACTTTAAAGTTGAATAATGGAAAAAACTACCAAGTTTGGATTGACTAT
GCAGATTTCCACATTAATGTTACTATGGCACCAGTTGGTATGAAAAGGCCTAAGCAACCT
TTGTTGGATTTCCCTCTGAATCTTTCTCAAGTTTTTAAGGATGAGATGTATGTTGGGTTCA
CAGCCTCCACTGGAATGCTTGCTCAAGGTCACAAGATTTTAGCTTGGAGTTTTAGTAACT
CTAATTTTTCGATTAGTGATGCTTTGATCACACAAGGTTTGCCTTCATTTGAGCTTCCTAA
AGATCTAATTTATCGATCGAAGGGGTTTATTGTAGGTATGACAATGTCACTTTTCTTTCTT
GTTGTGGTCACTTCTGTAGCTTCATTATTTCTGATTAAGAGAAATAGGAGACTAAAAAGG
GAAAAAGAGGTATAGAAGCTTGGGAATTGGAATATTGGCCACATAGGATTACTTATCAAG
AAATGATGCTGCAACAAAGGGTTTTGCTGATGAAATTGTGATTGGAGTTGGAGGTAATGG
GAAGGTTTATAAAGGGGTTTTGGCTGGGGGTTCAGAGGTTGCAGTAAAGCGCATGTCTC
ATGACAACTGTGAAGGGGAAAGGCAATTCTTGAGTGAGATTTCAAGTCTTGGTAGGCTAA
AGCACAGAAATTTGGTGGCATTAAGAGGCTGGTGCAAGAAAGGTGGAGGCAGCCTGATT
GTGGTTTATGATTATATGGAAAATGGGAGCTTGGATAAAAAGCTGTTTGAATGTGATAAG
GGAAACATGTTGAGTTTTGAAGACAGAATTAAGATTTTGAAAGATGTGGCATCAGGGGTT
TTATACTTGCATGAGGGGTGGGAGGCAAAAGTGTTGCATAGGGACATTAAGGCTAGCAA
TGTTTTACTTGACAAGGAAATGCATGCAAGAATTGGTGATTTTGGTCTAGCAAGAATGCA
TGATCATGGTCAAGTGGCTAAGGAGACTCGAGTTGTTGGCACAGTTGGTTACCTGGCGC
CAGAGTTTGCCAAGACAGGCCGTGCCTCCACGCAAACTGATTTGTTCAGTTATGGAGTT
CTAATATTGGAGGTGATGTGTGGAAGGAGGCCAATAGAGGAAGGCAAGCCACCTTTAGT
GGATTGGCTGTGGGAACTAATGAAACGAGGCGAATTGAGTAATGCCTTTGATAATCAATT
AAAGAGTAATAAAGGATTTAATGAAGAGGAGGCCTTAAGGGTATTGCAATTAGGCATGAT
ATGCGCGAGCCTAGACCCCAGAACTAGGCCAACCGCGAGACAAGTGGTGAAATTCTTTG
AAAGGAACTCTGATGGAGCTGAATCTGAAGCTGAAGATATGGATGCTTACCTTCTGGAAA

GTATGAGACATAATACCATGTTGACCAATTATTGTTCTTCCATGCCTATTTCTTGGACAAA
TTCTCTAGTGGAGGGTAGGTGA

>NbS00026087g0010.1
MKSIPTYSNILVWFILLFCFCCSNKSAFAITEFDFGTLTLSNLKLLGDAHLGDNNSVQLTRDLA
VPNSGAGKALYSEPVRFRQPDLDFPASFSTFFSFSVTNLNPSSIGGGLAFVLTPNDESIGDA
GGYIGILDSKGTQSGTISVEFDTLMDVEFNDINGNHVGLDLNSMVSTQVSDLDSIGVDLKSGD
IVNSWIEYSGSTAELNVFVSYSNLKPKEPFLSVVLDIAEYVNDFMFVGFSGSTQGSTEIHSIEW
WSFSSSFEASPKSAAAAAPPPPTASLMNPAADSVTSWPPSMAPSESNSSASIMQDKSNGK
CHNNFCKQGPGAVVGVVTASAFFLAFATLVLIWLYTKKFKKVKNSEFLASDVIKMPKEFSYKE
LKLATKAFDLTRIIGHGAFGTVYKGILSDNGSIVAVKRCSHSGQGKAEFLSELSIIGTLRHRNLV
RLQGWCHEKGEILLVYDLMPNGSLDKALFESRMILPWLHRRKILLGVASALAYLHQECENQVI
HRDIKSSNIMLDEGTSHLLMTLQDSVSDLNGMITLSTSSSENSFNSAGNGMDLV

>NbS00026087g0010.1 Corrected; GenBank ID: KT225310
MKSIPTYSNILVWFILLFCFCCSNKSAFAITEFDFGTLTLSNLKLLGDAHLGDNNSVQLTRDLA
VPNSGAGKALYSEPVRFRQPDLDFPASFSTFFSFSVTNLNPSSIGGGLAFVLTPNDESIGDA
GGYIGILDSKGTQSGTISVEFDTLMDVEFNDINGNHVGLDLNSMVSTQVSDLDSIGVDLKSGD
IVNSWIEYSGSTAELNVFVSYSNLKPKEPFLSVVLDIAEYVNDFMFVGFSGSTQGSTEIHSIEW
WSFSSSFEASPKSAAAAAPPPPTASLMNPAADSVTSWPPSMAPSESNSSASIMQDKSNGK
CHNNFCKQGPGAVVGVVTASAFFLAFATLVLIWLYTKKFKKVKNSEFLASDVIKMPKEFSYKE
LKLATKAFDLTRIIGHGAFGTVYKGILSDNGSIVAVKRCSHSGQGKAEFLSELSIIGTLRHRNLV
RLQGWCHEKGEILLVYDLMPNGSLDKALFESRMILPWLHRRKILLGVASALAYLHQECENQVI
HRDIKSSNIMLDEGFNARLGDFGLARQVEHDKSPDATVAAGTMGYLAPEYLLTGRATEKTDV
FSYGAVVLEVASGRRPIERETTRVEKVGVNSNLVEWVWGLHREGNLLMAADSRLCGEFDE
GEMRRVLMVGLACSQPDPMVRPTMRSVVQMLVGEAEVPIVPRTKPSMSFSTSHLLMTLQD
SVSDLNGMITLSTSSSENSFNSAGNGMDLV

>NbS00026087g0010.1 Corrected; GenBank ID: KT225310
ATGAAGTCAATTCCTACATATAGCAACATTCTTGTATGGTTCATACTATTGTTCTGTTTTTG
CTGTAGTAATAAGTCAGCATTTGCTATCACTGAGTTTGATTTTGGAACATTAACACTGAGT
AATTTGAAGCTTCTTGGAGATGCACATTTGGGTGACAACAACAGTGTTCAGTTAACACGT
GACCTAGCCGTGCCAAATTCCGGCGCCGGAAAAGCTTTATATTCCGAACCAGTAAGATT
CCGGCAGCCGGATCTTGATTTTCCGGCGAGTTTCTCTACATTCTTTTCATTTTCAGTGAC
TAATTTGAACCCGTCGTCTATTGGTGGTGGTCTTGCTTTTGTACTCACGCCTAATGATGA
GTCAATAGGTGATGCTGGTGGGTATATTGGAATCTTGGATTCTAAAGGCACACAAAGTG
GTACAATTTCAGTTGAATTTGACACCCTTATGGATGTTGAGTTTAATGATATTAATGGAAA
TCATGTTGGCTTGGATCTGAATTCAATGGTTTCAACTCAAGTTAGTGATTTGGATTCTATT
GGTGTTGATCTCAAGAGTGGTGACATAGTCAATTCTTGGATTGAATATTCTGGTTCTACT
GCAGAGTTGAATGTGTTTGTATCATACTCTAATTTAAAACCAAAGGAACCATTTTTGTCTG
TTGTTTTGGATATTGCTGAATATGTAAATGATTTCATGTTTGTTGGGTTTTCTGGTTCAACT
CAAGGGAGTACTGAGATTCATAGTATTGAGTGGTGGAGTTTTAGTTCATCATTTGAAGCA
AGTCCTAAGTCGGCGGCCGCAGCTGCGCCACCACCGCCAACGGCTAGTTTAATGAATC
CCGCGGCGGACTCCGTCACGTCGTGGCCGCCTTCTATGGCTCCTTCAGAGTCTAATAGT
AGTGCAAGTATAATGCAAGATAAGAGCAATGGGAAATGTCATAACAATTTTTGTAAACAA
GGTCCTGGAGCTGTTGTTGGGGTAGTAACTGCTAGTGCATTTTTTCTTGCATTTGCTACA
TTAGTACTTATTTGGTTATACACCAAAAAGTTCAAGAAAGTGAAAAATTCTGAATTTTTGG
CATCTGATGTTATCAAAATGCCTAAAGAGTTTAGCTATAAAGAGCTTAAATTGGCTACAAA
AGCCTTTGATTTGACGAGGATTATAGGCCACGGTGCATTTGGGACAGTTTACAAGGGCA
TTTTATCGGACAATGGTAGCATTGTAGCAGTCAAGAGATGTAGTCATAGCGGACAAGGG
AAGGCGGAGTTCTTATCTGAATTATCTATAATTGGAACACTTAGGCACAGAAATCTTGTTA
GACTTCAAGGTTGGTGCCATGAGAAAGGTGAAATTTTGTTAGTCTATGATCTAATGCCTA
ATGGGAGTCTTGATAAGGCATTATTTGAATCAAGAATGATTCTACCTTGGCTACATAGGA
GGAAAATTTTGCTAGGTGTTGCTTCAGCCTTGGCATATTTACATCAAGAATGTGAAAATCA
GGTGATTCATAGGGATATAAAGAGTAGTAACATTATGTTAGATGAAGGGTTCAATGCAAG
ATTAGGTGATTTTGGATTAGCAAGACAAGTTGAACATGACAAGTCCCCCGATGCAACGGT
AGCAGCCGGGACAATGGGCTACTTGGCTCCTGAATACTTGTTAACCGGAAGAGCAACCG
AAAAAACTGATGTTTTTAGCTATGGAGCAGTTGTTCTTGAAGTGGCAAGTGGAAGGAGGC
CAATTGAGAGGGAAACAACAAGAGTTGAGAAAGTTGGAGTGAATAGTAACTTAGTTGAAT
GGGTTTGGGGATTGCATAGAGAAGGGAATTTGCTAATGGCAGCTGATTCAAGACTATGT

GGTGAGTTTGATGAAGGAGAAATGAGAAGGGTACTAATGGTTGGGTTAGCTTGTTCACA
ACCTGACCCTATGGTTAGACCAACAATGAGAAGTGTGGTCCAAATGCTAGTAGGTGAAG
CTGAAGTTCCTATTGTCCCAAGAACTAAGCCTTCTATGAGTTTCAGCACATCACATCTTCT
AATGACTTTGCAAGATAGTGTTTCTGATTTGAATGGTATGATCACACTTTCAACTTCATCA
TCTGAAAACAGCTTCAATAGTGCTGGCAATGGCATGGACTTGGTCTAA

>NbS00026192g0010.1
MKLIPAYSNILVWFILLFCFCSSNKSIFAITEFDFGTLTLSNLKLLGDAHLGDNNSVQLTRDLAV
PNSGSGKALYSKPVRFRQPGLDFPASFSTFFSFSVTNLNPSSIGGGLAFVLTPNDESVGDAG
GYMGILDSKGTQSGTMSVEFDTLMDVEFKDINGNHVGLDLNSMVSTQVGDLGSIGIDLKSGD
IVNSWIEYSGSTAELNVFVSYSNIKPKEPFLSVVLDIAEYVNDFMFVGFSGSTQGSTEIHSIEW
WSFSSSFDGSPKSAATAAPPPPTASLMNPTADSVKSRPPSMAPSESNSSASIMQDKSSGKC
HNNFCKQGPGAVVGVVTASAFFLAFATLVLIWLYTKKFKKVKNSEFLASDVIKMPKEFSYKEL
KLATKAFDSTRIIGHGAFGTVYKGILPDNGGIVAVKRCSHNGQGKAEFLSELSIIGTLRHRNLV
RLQGWCHEIGEILLVYDLMPNGSLDKALFESRMILPWVHRRKILLGVASALAYLHQECENQVI
HRDIKSSNIMLDEGTSHLLMTLQDSVSDLNGMITLSSSSSENSFTGVANGMDLVSSSLLLRHV
YFMEKTHGPACS

>NbS00026192g0010.1 Corrected; GenBank ID: KT225311
MKLIPAYSNILVWFILLFCFCSSNKSIFAITEFDFGTLTLSNLKLLGDAHLGDNNSVQLTRDLAV
PNSGSGKALYSKPVRFRQPGLDFPASFSTFFSFSVTNLNPSSIGGGLAFVLTPNDESVGDAG
GYMGILDSKGTQSGTMSVEFDTLMDVEFKDINGNHVGLDLNSMVSTQVGDLGSIGIDLKSGD
IVNSWIEYSGSTAELNVFVSYSNIKPKEPFLSVVLDIAEYVNDFMFVGFSGSTQGSTEIHSIEW
WSFSSSFDGSPKSAATAAPPPPTASLMNPTADSVKSRPPSMAPSESNSSASIMQDKSSGKC
HNNFCKQGPGAVVGVVTASAFFLAFATLVLIWLYTKKFKKVKNSEFLASDVIKMPKEFSYKEL
KLAPKAFDSTRIIGHGAFGPVSKGILPDNGGIVAVKRCSHNGQGKAEFLSELSIIGTLRHRNLV
RLQGWCHEIGEILLVYDLMPNGSLDKALFESRMILPWVHRRKILLGVASALAYLHQECENQVI
HRDIKSSNIMLDEGFNARLGDFGLARQVEHDKSPDVTVAAGTMGYLAPEYLLTGRATEKTDV
FSYGAVVLEVASGRRPIERETTRVEKVGVKSNLVEWVWGLHREGNLLMAADSRLYDEFDEG
EMRRVLMVGLACSQPDPMVRPTMRSVVQMLVGEAEVPIVPRTKPCMSFSTSHLLMTLQDS
VSDLNGMITLSSSSSENSFTGVANGMDLV

>NbS00026192g0010.1 Corrected; GenBank ID: KT225311
ATGAAGTTAATTCCTGCATATAGCAACATTCTTGTATGGTTCATACTATTGTTCTGTTTTTG
CAGTAGTAATAAATCAATATTTGCTATAACTGAGTTTGACTTTGGAACATTAACTCTGAGT
AATTTGAAGCTTCTTGGAGATGCTCATTTGGGTGACAACAACAGTGTTCAGTTAACACGT
GATCTTGCCGTGCCAAATTCCGGTTCCGGAAAAGCTTTATATTCAAAGCCAGTAAGATTC
CGGCAGCCGGGTCTTGATTTTCCGGCGAGTTTCTCTACATTCTTTTCATTTTCAGTGACT
AATTTGAACCCATCATCTATCGGTGGCGGCCTTGCTTTTGTACTCACTCCTAATGATGAG
TCAGTAGGTGATGCTGGTGGGTATATGGGAATCTTGGATTCAAAAGGCACACAAAGTGG
TACGATGTCAGTTGAATTTGACACCCTTATGGATGTTGAGTTTAAAGATATTAATGGAAAT
CATGTTGGTTTGGATCTGAATTCAATGGTTTCAACTCAAGTTGGTGATTTAGGTTCTATTG
GTATTGATCTCAAGAGTGGTGATATAGTCAATTCTTGGATTGAATATTCTGGTTCTACTGC
AGAGTTGAATGTGTTTGTATCATACTCTAATATAAAACCAAAGGAACCATTTTTGTCTGTT
GTTTTGGATATTGCTGAATATGTAAATGATTTCATGTTTGTTGGGTTTTCTGGTTCAACTC
AAGGGAGTACTGAGATTCATAGTATTGAGTGGTGGAGTTTTAGTTCATCATTTGATGGAA
GTCCTAAGTCGGCCGCCACGGCTGCGCCACCACCGCCAACAGCTAGCTTGATGAACCC
AACGGCAGATTCAGTCAAGTCGCGGCCACCTTCTATGGCTCCTTCAGAATCTAATAGTAG
TGCAAGTATAATGCAAGATAAGAGCAGTGGGAAATGTCATAACAATTTTTGTAAACAAGG
TCCTGGAGCTGTTGTTGGGGTGGTAACTGCTAGTGCATTTTTTCTTGCATTTGCTACATT
AGTACTTATTTGGTTATACACCAAAAAGTTCAAGAAAGTGAAAAATTCTGAATTTTTGGCA
TCTGATGTTATCAAAATGCCTAAAGAGTTTAGCTATAAAGAGCTTAAATTGGCTCCAAAAG
CTTTTGATTCGACGAGGATTATAGGCCACGGTGCATTTGGGCCAGTTTCCAAGGGCATTT
TACCGGACAATGGTGGCATTGTGGCAGTGAAGAGATGTAGTCATAATGGACAAGGGAAA
GCGGAGTTCTTATCAGAATTATCTATAATTGGAACACTTAGGCACAGAAATCTTGTTAGAC
TTCAAGGATGGTGCCATGAGATAGGTGAAATTTTGTTAGTCTATGATCTTATGCCTAATG
GGAGTCTTGATAAGGCATTATTTGAATCAAGAATGATTCTACCTTGGGTACATAGGAGGA
AAATTTTGCTAGGTGTTGCTTCAGCCTTGGCATATTTACATCAAGAATGTGAAAACCAGG
TGATTCACAGGGATATAAAGAGTAGTAACATTATGTTGGATGAAGGGTTCAATGCAAGAT
TAGGTGATTTTGGATTAGCAAGACAAGTTGAACATGACAAGTCCCCCGATGTAACAGTAG

CAGCTGGGACAATGGGCTACTTGGCTCCTGAATACTTGTTAACCGGAAGAGCAACCGAA
AAAACTGATGTTTTTAGCTATGGAGCTGTGGTTCTTGAAGTGGCAAGTGGAAGGAGGCC
AATTGAGAGGGAAACAACAAGAGTTGAGAAAGTTGGAGTGAAGAGTAACTTAGTTGAAT
GGGTTTGGGGATTGCATAGAGAAGGGAATTTGCTAATGGCAGCAGATTCAAGACTTTAT
GATGAGTTTGATGAAGGAGAAATGAGAAGGGTACTAATGGTTGGGTTAGCTTGTTCACAA
CCTGACCCTATGGTTAGACCAACAATGAGAAGTGTGGTCCAAATGCTAGTAGGTGAAGC
TGAAGTTCCTATTGTCCCAAGAACTAAGCCTTGTATGAGTTTCAGCACATCACATCTTCTA
ATGACTTTGCAAGATAGTGTTTCTGATTTGAATGGTATGATCACACTTTCCTCTTCATCAT
CTGAAAACAGTTTCACTGGTGTTGCCAATGGGATGGACTTGGTCTAA

>NbS00008527g0008.1
MEIYPLLIVFCVLASNGFGVLSTTEFDFGILALNSLKLLGDAHMGNNKIKLTRELAVPNSGTGK
VLYSKPVRFRQPGLDFPASFSTFFSFSVTNLNPSSIGGGLAFVITPEDDLVGDSGGYLGIMDA
KGTQNGNFAIEFDTLMDVEFKDINGNHVGLDLNSMVSTQVGDLDSIGVDLKSGDLVNSWVE
YSGSNKKLNIFVSYSNLKPKEPFLSVTIDLSEYVNDFMFVGFCGSTQGSTEIHSIEWWSFSSS
FDVNPKSPAAALPPPPPTASLMNPTANSVTSPPPSMAPVQSNGTVNSEQKSSSSKCHNSFC
KQGAGAVVGVVTAGAFFLAFATLVLVWLYSNKFKNVKNSENLGSDIIKMPKEFSYKELKIATK
GFDSTRIIGHGAFGTVYKGILSETGDIVAMKRDGAFGTVYKGILSETGDIVAVKRCSHNGQGK
AEFLSELSIIGTLRHRNLVRLQGWCHEKGEILLVYDLMPNGSLDKALFESRIVLPWLHRRKILL
GVASALAYLHQECENQDSVSDLDGMITLSTSSSENSFNCGGVGMDLV

>NbS00008527g0008.1 Corrected; GenBank ID: KT225312
MEIYPLLIVFCVLASNGFGVLSTTEFDFGILALNSLKLLGDAHMGNNKIKLTRELAVPNSGTGK
VLYSKPVRFRQPGLDFPASFSTFFSFSVTNLNPSSIGGGLAFVITPEDDLVGDSGGYLGIMDA
KGTQNGNFAIEFDTLMDVEFKDINGNHVGLDLNSMVSTQVGDLDSIGVDLKSGDLVNSWVE
YSGLTKKLNIFVSYSNLKPKEPFLSGTIDLSEYVNDFMFVGFCGSTQGSTEIHSIEWWSFSSS
FDVNPKSPAAALPPPPPTASLMNPTANSVTSPPPSMAPVQSNGTVNSEQKSSSSKCHNSFC
KQGAGAVVGVVTAGAFFLAFATLVLVWLYSNKFKNVKNSENLGSDIIKMPKEFSYKELKIATK
GFDSTRIIGHGAFGTVYKGILSETGDIVAMKRCSHNGQGKAEFLSELSIIGTLRHRNLVRLQG
WCHEKGEILLVYDLMPNGSLDKALFESRMVLPWLHRRKILLGVASALAYLHQECENQVIHRDI
KTSNIMLDEGFNARLGDFGLAKQIEHDKSPDATVAAGTMGYLAPEYLLTGRASEKTDVFSYG
AVVLEVASGRRPIEKETNGVGKVGLNSNLVEWVWGLHKEGRLLAAADSRLNSEFEEQEMR
RVLLVGLACSHTDPMARPTMRGVVQMLVGEAEVPIVPRAKPTMSFSTSHLLMTLQDSVSDL
DGMITLSTSSSENSFNCGGVGMDLV

>NbS00008527g0008.1 Corrected; GenBank ID: KT225312
ATGGAGATTTACCCATTACTTATAGTGTTCTGTGTCTTGGCTAGTAATGGGTTCGGTGTA
CTGTCTACTACTGAGTTTGATTTTGGTATTTTAGCTTTGAACAGTTTAAAGCTATTAGGAG
ATGCTCATATGGGTAATAACAAAATCAAATTGACACGTGAGCTCGCCGTTCCAAATTCCG
GTACCGGAAAAGTTTTATATTCTAAACCAGTAAGATTCCGGCAGCCGGGTCTTGATTTTC
CGGCGAGTTTCTCTACGTTTTTCTCATTTTCTGTTACAAATCTGAACCCTTCATCGATCGG
CGGTGGTTTAGCTTTTGTCATCACGCCGGAAGATGACTTGGTTGGAGACTCCGGTGGTT
ATTTGGGAATTATGGATGCAAAAGGGACCCAAAATGGTAATTTTGCTATTGAATTTGATAC
ACTTATGGATGTTGAATTTAAAGATATTAATGGGAATCATGTGGGGTTGGATTTAAATTCA
ATGGTTTCAACGCAAGTTGGTGATCTTGATTCTATTGGTGTTGATCTAAAGAGTGGTGAT
TTAGTAAATTCATGGGTTGAATATTCAGGTTTAACAAAAAAGCTGAATATTTTTGTTTCATA
TTCTAATTTAAAGCCCAAAGAACCATTTTTATCAGGTACTATTGATCTTTCTGAGTACGTA
AATGATTTTATGTTTGTGGGGTTTTGTGGTTCAACACAAGGGAGTACTGAGATTCATAGT
ATTGAATGGTGGAGTTTCAGTTCATCATTTGATGTAAATCCAAAATCTCCAGCGGCGGCG
CTGCCTCCGCCGCCCCCAACCGCTAGTTTGATGAATCCCACGGCGAATTCTGTTACATC
GCCGCCGCCTTCGATGGCTCCTGTTCAGTCTAATGGTACTGTGAATTCAGAGCAAAAAA
GTAGTAGTAGCAAATGCCATAACAGTTTTTGTAAACAAGGTGCTGGAGCTGTTGTTGGAG
TTGTAACTGCTGGCGCATTTTTCTTAGCTTTTGCTACATTAGTACTTGTTTGGTTGTATTC
CAACAAATTCAAGAATGTGAAGAATTCTGAAAATTTGGGGTCTGATATAATCAAAATGCCT
AAGGAATTCAGCTATAAGGAGCTCAAAATCGCGACGAAAGGTTTTGATTCGACGAGGAT
AATCGGGCATGGTGCATTTGGAACAGTTTATAAGGGGATTTTATCTGAGACTGGTGACAT
TGTAGCAATGAAAAGGTGTAGTCATAACGGACAAGGGAAAGCTGAGTTTTTATCTGAATT
ATCAATAATTGGAACACTTAGGCATAGAAATCTTGTTAGACTTCAAGGATGGTGTCATGA
GAAAGGTGAAATTTTGTTAGTTTATGATTTGATGCCAAATGGTAGTCTTGATAAGGCATTA
TTTGAGTCAAGAATGGTACTCCCATGGCTACATAGGAGGAAAATTTTGTTAGGTGTTGCT

TCAGCCTTAGCATATTTACATCAAGAATGTGAAAATCAAGTTATTCATAGGGATATTAAGA
CTAGTAATATTATGTTGGATGAAGGGTTTAATGCAAGATTAGGTGATTTTGGATTAGCAAA
ACAAATTGAACATGACAAATCTCCTGATGCAACAGTAGCAGCAGGAACAATGGGATACTT
AGCACCGGAATATTTGTTAACCGGTCGAGCAAGTGAAAAAACTGATGTTTTTAGCTATGG
GGCAGTGGTTCTTGAAGTGGCAAGTGGGAGAAGGCCAATAGAGAAAGAAACAAATGGG
GTTGGGAAAGTTGGATTGAATAGTAATTTGGTGGAATGGGTGTGGGGATTGCATAAAGA
AGGGAGATTATTAGCAGCAGCTGATTCAAGATTGAATAGTGAGTTTGAGGAACAAGAAAT
GAGAAGGGTTTTGTTAGTTGGTTTGGCTTGTTCACACACTGACCCTATGGCTAGACCAAC
AATGAGAGGTGTTGTTCAAATGTTAGTTGGTGAGGCTGAAGTTCCAATTGTTCCTAGAGC
TAAGCCAACTATGAGTTTTAGCACATCTCATCTGCTAATGACATTGCAGGACAGTGTTTCT
GACTTGGATGGTATGATCACACTTTCAACTTCTTCATCTGAAAATAGCTTCAATTGTGGTG
GTGTGGGTATGGACCTAGTCTAG

> NbS00016101g0013.1
MEIYPLLIVLFCFLASNGFSVLSTTEFDFGTLALNSLKLLGDAHMGGNKIKLTRELAVPNSGAG
KVLYSKPVRFRQPGLDFPASFSTFFSFSVTNLNPSSIGGGLAFVITPDDESVGDAGGYLGIMD
AKGTQNGNFAIEFDTLMDVEFKDINGNHVGLDLNSMVSTQVGDLDSVGVDLKSGDLVNSWV
EYSGSNKKLNIFVSYSNLKPKEPFLSVTIDLSEYVNDFMFVGFCGSTQGSTEIHSIEWWSFSS
SFDVNPKSPAAALPPPPPTASLMNPTADSVTSLPPSTAPLQSNGTVNSEQKSSSSKCHNSF
CKQGAGAVVGVVTAGAFFLAFATLVLVWLYSKKFKNVENSENLGSDIIKMPKEFSYKELKIAT
KGFDSTRIIGHGAFGTVYKGILSETGDIVAVKRCSHNGQGKAEFLSELSIIGTLRHRNLVRLQG
WCHEKGEILLVYDLMPNGSLDKALFESRMVLPWLHRRKILLGVASALAYLHQECENQVMLR
DIKTSNIMLDEGFNARLDSVSDLNGMITLSTSSSENSFNGGGVGMDLV

>NbS00016101g0013.1 Corrected; GenBank ID: KT225313
MEIYPLLIVLFCFLASNGFSVLSTTEFDFGTLALNSLKLLGDAHMGGNKIKLTRELAVPNSGAG
KVLYSKPVRFRQPGLDFPASFSTFFSFSVTNLNPSSIGGGLAFVITPDDESVGDAGGYLGIMD
AKGTQNGNFAIEFDTLMDVEFKDINGNHVGLDLNSMVSTQVGDLDSVGVDLKSGDLVNSWV
EYSGSNKKLNIFVSYSNLKPKEPFLSVTIDLSEYVNDFMFVGFCGSTQGSTEIHSIEWWSFSS
SFDVNPKSPAAALPPPPPTASLMNPTADSVTSLPPSTAPLQSNGTVNSEQKSSSSKCHNSF
CKQGAGAVVGVVTAGAFFLAFATLVLVWLYSKKFKNVENSENLGSDIIKMPKEFSYKELKIAT
KGFDSTRIIGHGAFGTVYKGILSETGDIVAVKRCSHNGQGKAEFLSELSIIGTLRHRNLVRLQG
WCHEKGEILLVYDLMPNGSLDKALFESRMVLPWLHRRKILLGVASALAYLHQECENQVMLR
DIKTSNIMLDEGFNARLGDFGLAKQIEHDKSPDATVAAGTMGYLAPEYLLTGRASEKTDAFSY
GAVVLEVASGRRPIEKETNGVGKVGLNSNLVEWVWGLHKEGRLLAAADSRLNGEFEEQEM
RRVLLVGLACSHPDPMARPTMRGVVQMLVGEAEVPIVPRAKPTMSFSTSHLLMTLQDSVSD
LNGMITLSTSSSENSFNGGGVGMDLV

>NbS00016101g0013.1 Corrected; GenBank ID: KT225313
ATGGAGATTTACCCATTACTGATAGTATTATTTTGTTTCTTGGCTAGTAATGGGTTCAGTG
TACTGTCTACTACTGAGTTTGATTTTGGTACTTTAGCTTTGAACAGTTTAAAGCTATTAGG
AGATGCTCATATGGGTGGCAACAAAATCAAATTGACACGTGAGCTCGCCGTGCCAAATT
CCGGTGCCGGAAAAGTTTTATATTCTAAACCAGTAAGATTCCGGCAGCCAGGTCTTGATT
TTCCGGCGAGTTTCTCTACGTTTTTCTCATTTTCTGTTACAAATCTGAACCCTTCATCGAT
CGGCGGGGGTTTAGCTTTTGTCATCACGCCGGATGATGAATCAGTAGGTGATGCCGGC
GGCTATTTGGGAATTATGGATGCAAAAGGGACCCAAAATGGTAATTTTGCAATTGAATTT
GATACACTTATGGATGTTGAATTTAAAGATATTAATGGGAATCATGTGGGGTTAGATTTAA
ATTCAATGGTTTCAACTCAAGTTGGTGATCTTGATTCTGTTGGTGTTGATCTCAAGAGTG
GTGATTTGGTAAATTCATGGGTTGAATATTCAGGTTCTAACAAAAAGCTGAATATTTTTGT
TTCATATTCTAATTTAAAGCCCAAAGAACCATTTTTATCAGTTACTATTGATCTTTCTGAGT
ATGTAAATGATTTTATGTTTGTGGGGTTTTGTGGTTCAACACAAGGGAGTACTGAGATTC
ATAGTATTGAATGGTGGAGTTTCAGTTCATCATTTGATGTAAATCCAAAATCTCCGGCGG
CGGCGCTGCCTCCTCCGCCGCCAACGGCTAGTTTGATGAATCCCACGGCAGATTCTGTT
ACATCGCTGCCGCCTTCGACGGCTCCATTGCAGTCTAATGGTACTGTGAATTCAGAGCA
AAAAAGTAGTAGTAGCAAATGCCATAACAGTTTTTGTAAACAAGGTGCTGGGGCTGTTGT
TGGTGTTGTAACTGCTGGAGCATTTTTCTTAGCTTTTGCTACATTAGTACTTGTTTGGTTG
TATTCGAAAAAATTCAAGAATGTGGAGAATTCTGAAAATTTGGGGTCTGATATTATCAAAA
TGCCTAAGGAATTCAGCTATAAGGAGCTTAAAATTGCGACGAAAGGTTTTGATTCGACGA
GGATTATAGGGCATGGTGCATTTGGGACAGTTTATAAGGGGATTTTGTCTGAGACTGGT
GATATTGTAGCAGTGAAAAGGTGTAGTCATAATGGACAAGGTAAAGCTGAGTTTTTATCT

GAATTATCAATAATTGGAACACTTAGGCATAGAAATCTTGTTAGACTTCAAGGATGGTGTC
ATGAGAAAGGTGAAATTTTGTTAGTTTATGATTTGATGCCAAATGGTAGTCTTGATAAGGC
ATTATTTGAGTCAAGAATGGTACTCCCATGGCTACATAGGAGGAAAATTTTGTTAGGTGT
TGCTTCAGCCTTAGCATATTTACATCAAGAATGTGAAAATCAAGTTATGTTAAGGGATATT
AAGACTAGTAATATTATGTTGGATGAAGGGTTTAATGCAAGATTAGGTGATTTTGGGTTA
GCAAAACAAATTGAACATGACAAATCTCCTGATGCGACAGTAGCAGCAGGAACAATGGG
ATACTTAGCACCTGAATATTTATTAACCGGTCGAGCAAGTGAAAAAACTGATGCTTTTAGC
TATGGCGCAGTGGTTCTTGAAGTGGCAAGTGGGAGAAGGCCAATTGAGAAAGAAACAAA
TGGGGTTGGGAAAGTTGGATTAAATAGCAATTTGGTGGAATGGGTTTGGGGATTACATAA
AGAAGGGAGGTTATTAGCAGCAGCTGATTCAAGATTGAATGGTGAGTTTGAGGAACAAG
AAATGAGAAGGGTTTTGTTAGTTGGTTTGGCTTGTTCACACCCTGACCCTATGGCTAGAC
CAACAATGAGAGGTGTGGTTCAAATGTTAGTTGGTGAGGCTGAAGTTCCAATTGTCCCTA
GAGCTAAGCCAACTATGAGTTTTAGCACATCTCATCTGCTAATGACTTTGCAAGATAGTG
TTTCTGACTTGAATGGTATGATCACACTTTCCACGTCTTCATCTGAAAATAGCTTCAATGG
TGGTGGTGTGGGTATGGACCTAGTCTAG

>NbS00034752g0003.1 GenBank ID: KT225314
MNSISLVIIFFLIPFVHSIYFKIPRFGPDVTDILYEGDAVASVGEIEFNKFTYLCRVAHAIYKEKVP
IWDPDSTKLADFFTHFSFTIDTLNRSLYGHGIAFFLAQVGFHIPPNSDGGFLGLFNTTTSDSAQ
NQIVVVEFDSFSNPEWDPPFEHVGINKNSIASSVTAPWNTLVILRKWRRRKMKRNPETISLTS
FNDDLEKGTGPKKFSYEELDTSTNHFSEERKLGEGGFGEVYKGYLIDLDIAIAVKKISRGSKQ
GKKEYITEVKVISRLRHRNIVQLIGWCHDQEWEQCVIHRDIKSSNVMLDSSFNVKLGDFGLAR
LMDHELGPQTTGLAGTLGYLAPEYIKTGRASKESDVYSFGIVAQEIATGRKSVDPGTGKSDA
VLVEYVWELYKGQLLSAVDEKLNLDFDAKQVERLMATRLWCAHPESNLRPSIRQAIHVLNFE
ASLPNIPIKMPVPVYDLPSSGEPGVSSGEPTITYTSIDVGR

>NbS00034752g0003.1 GenBank ID: KT225314
ATGAACTCCATTTCCCTCGTTATTATCTTCTTTCTAATTCCGTTTGTACATTCAATCTACTT
TAAAATACCACGTTTTGGCCCGGACGTGACTGATATACTCTATGAAGGAGATGCAGTTGC
TTCTGTAGGAGAGATTGAGTTTAACAAATTTACTTATCTTTGTCGAGTTGCACATGCTATC
TATAAAGAGAAAGTACCAATTTGGGATCCTGACTCTACTAAACTTGCTGATTTCTTCACAC
ATTTTTCGTTCACTATCGATACTCTAAATCGTTCTTTGTATGGTCATGGCATTGCATTTTTC
CTCGCTCAGGTTGGTTTTCATATCCCACCTAACTCAGATGGTGGTTTTCTTGGACTGTTC
AATACTACCACCAGTGATTCAGCTCAAAACCAAATTGTTGTTGTTGAGTTTGACTCGTTTT
CCAACCCCGAATGGGATCCTCCATTCGAACATGTCGGTATAAACAAGAATTCTATTGCTT
CATCAGTGACAGCTCCTTGGAATGTTAGTTTACATAGTGGAAATCCTATCGATGCATGGA
TTACTTACAATTCTACGACAAAAAATCTGAGTGTCTTTTGGAACTATGGAAAAGGCTCCAA
TTCCAGCATATCTTATATAATAAACCTTAGAGAGGTTTTGCCTTCGTGGATAACGATAGGA
TTCTCTGCTGCAACAGGTACAAATGTTGAACGACATACACATGAATCGTGGGAGTTCAGC
TCGAGTCTTGATATGACAGACTTAGGGGGAAATGGTCGCAGAAAGATTGGATTGATTGC
AGGGTTAACAGCATTAGGGGGAGTTTTATTTGTGGGCGCGATTTAGACTTTAGTAATATT
GAGGAAATGGAGACGACGAAAGATGAAGAGAAATCCAGAGACAATAAGCTTAACATCTT
TCAATGATGATCTTGAAAAGGGAACAGGACCGAAAAAGTTTTCTTACGAAGAGCTGGATA
CGTCAACAAATCACTTCTCAGAGGAACGAAAGTTGGGCGAAGGAGGATTTGGAGAAGTT
TACAAAGGTTACCTCATAGATCTTGATATTGCGATTGCGGTGAAGAAGATCTCGAGAGGG
TCTAAGCAGGGGAAAAAGGAATACATAACCGAGGTGAAGGTTATAAGTCGGTTAAGACA
TAGAAATATTGTGCAGCTAATTGGTTGGTGTCATGACCAAGGTAAGTTCTTACTTGTTTAC
GAATTCATGCCAAATCGTAGCTTAGATTGTCACTTATTTGGTAAGAGCAATCCTCTTAGTT
GGAGTACGAGGTATAAGATATCGCTCGGATTAGCATCTGCCTTGCTCTATCTACACGAAG
AATGGGAGCAGTGTGTGATCCATAGAGACATCAAATCGAGTAATGTAATGCTCGATTCAA
GTTTTAATGTCAAGCTCGGTGATTTTGGCTTAGCTAGACTAATGGACCATGAATTAGGTC
CTCAGACTACAGGGTTGGCTGGAACTTTAGGTTATTTGGCTCCTGAATACATAAAAACAG
GCCGAGCAAGTAAAGAGTCAGACGTATACAGCTTCGGAATAGTTGCACAAGAAATTGCA
ACCGGAAGAAAATCAGTTGATCCGGGGACGGGGAAATCTGATGCAGTGTTAGTGGAGTA
TGTTTGGGAGCTTTATAAAGGACAACTTCTTTCTGCTGTTGATGAGAAATTAAACCTGGAT
TTTGACGCGAAACAAGTAGAGCGATTGATGGCTACCAGGTTATGGTGTGCTCATCCGGA
GAGCAATCTGAGGCCATCTATAAGACAAGCAATTCATGTTCTGAATTTTGAGGCATCATT
GCCTAATATACCAATTAAGATGCCAGTTCCTGTGTATGATTTACCTAGTTCTGGTGAACCA
GGAGTTAGCTCGGGGGAGCCTACAATTACTTACACGAGCATTGATGTAGGTCGTTAA

>NbS00059538g0001.1
MIRFVSSKINPLSLFITFFLIPFVNSVYFKIPRFGPDVTDILYEGDAVASIGDIEFNKVTYLCRVA
HAIYREKVPIWDPDSTKLADFSTHFSFTIDTLNRSLYGHGIAFFLAPVGFRIPPNSDGGFLGLF
NTTTSDSAQNQIVVVEFDSFSNPEWDPPYEHVGINKNSIASSMTAPWNVSLHSGDPIDAWIT
YNSTTKNLSVFWNYGKGSNSSISYVINLKEVLPSWITIGFSAATGTNVERHTLESWEFSSSLDI
TELGGNGRRKIGLIAGLTALGGVLFVSTIFALVIFRKWRRQKMKKNPETISLTSFNDDLEKGTG
PKKFSYEELDTSTNHFSEERKLGEGGFGEVYKGYLIDLDIAVAVKRISRGSKQGKKEYITEVK
VISRLRHRNLVQLIGWCHDQGKFLLVYEFMPNGSLDCHLFGKRNPLSWSTRYKILLGLASALL
YLHEEWEQCVIHRDIKSSNVMLDSSFNVKLGDFGLARLMDHELGPQTTGLAGTLGYLAPEYI
KTGRASKESDVYSFGVVALEIATGRKSVDPGTRKSNVGLVEYVWDLYGKGQLLSAVDEKLN
LVFDAKQVELLMITGLWCAHPESNLRPSIRQAIHVLNFEASLPNIPIKMPVPVYYLPSSGEPGT
IILKILNSLSRELRFGIDAA

>NbS00059538g0001.1 Corrected; GenBank ID: KT225315
MIRFVSSKINPLSLFITFFLIPFVNSVYFKIPRFGPDVTDILYEGDAVASIGDIEFNKVTYLCRVA
HAIYREKVPIWDPDSTKLADFSTHFSFTIDTLNRSLYGHGIAFFLAPVGFRIPPNSDGGFLGLF
NTTTSDSAQNQIVVVEFDSFSNPEWDPPYEHVGINKNSIASSMTAPWNVSLHSGDPIDAWIT
YNSTTKNLSVFWNYGKGSNSSISYVINLKEVLPSWITIGFSAATGTNVERHTLESWEFSSSLDI
TELGGNGRRKIGLIAGLTALGGVLFVSTIFALVIFRKWRRQKMKKNPETISLTSFNDDLEKGTG
PKKFSYEELDTSTNHFSEERKLGEGGFGEVYKGYLIDLDIAVAVKRISRGSKQGKKEYITEVK
VISRLRHRNLVQLIGWCHDQGKFLLVYEFMPNGSLDCHLFGKRNPLSWSTRYKILLGLASALL
YLHEEWEQCVIHRDIKSSNVMLDSSFNVKLGDFGLARLMDHELGPQTTGLAGTLGYLAPEYI
KTGRASKESDVYSFGVVALEIATGRKSVDPGTRKSNVGLVEYVWDLYGKGQLLSAVDEKLN
LVFDAKQVELLMITGLWCAHPESNLRPSIRQAIHVLNFEASLPNIPIKMPVPVYYLPSSGEPGV
SSGDPTMTYTSIDVGR

>NbS00059538g0001.1 Corrected; GenBank ID: KT225315
ATGATACGATTTGTGTCTTCTAAAATAAACCCTCTTTCCCTCTTTATTACCTTCTTTCTAAT
TCCGTTTGTAAATTCAGTCTACTTTAAAATACCACGTTTTGGCCCGGACGTGACTGACAT
ACTCTATGAAGGAGATGCAGTTGCTTCTATAGGAGACATTGAGTTTAACAAAGTCACTTA
TCTTTGTCGCGTTGCACATGCTATATATAGAGAGAAAGTACCAATTTGGGATCCGGACTC
TACTAAACTTGCTGATTTCTCCACACATTTTTCGTTCACTATCGATACCTTAAATCGTTCTT
TGTATGGTCATGGCATTGCATTTTTCCTCGCTCCGGTTGGTTTTCGTATTCCACCTAACTC
AGATGGTGGTTTTCTTGGACTGTTCAATACTACCACCAGTGATTCAGCTCAGAACCAAAT
TGTTGTTGTTGAGTTTGACTCGTTTTCCAACCCCGAATGGGATCCTCCATACGAACATGT
TGGTATAAACAAAAATTCTATTGCTTCATCTATGACAGCTCCATGGAATGTTAGTTTACAT
AGTGGAGATCCTATTGATGCATGGATTACTTACAATTCTACGACAAAAAATCTAAGTGTCT
TTTGGAACTATGGAAAAGGCTCCAATTCCAGCATATCTTATGTAATAAACCTTAAAGAGGT
TTTGCCTTCGTGGATAACGATAGGATTCTCTGCTGCAACAGGTACAAATGTTGAAAGACA
TACACTTGAATCGTGGGAGTTCAGCTCGAGTCTTGATATAACAGAGTTAGGGGGAAATG
GTCGCAGAAAGATTGGATTGATTGCAGGGTTAACAGCATTAGGGGGAGTTTTGTTTGTG
AGCACGATTTTTGCTTTAGTAATATTTAGGAAATGGAGACGACAAAAGATGAAGAAAAAT
CCAGAGACAATTAGCTTAACATCTTTCAATGATGATCTTGAAAAGGGAACAGGACCGAAA
AAGTTTTCTTACGAAGAGCTGGATACTTCAACAAATCACTTCTCAGAGGAACGAAAGTTA
GGCGAAGGAGGATTTGGAGAAGTTTACAAAGGTTACCTCATCGATCTTGATATCGCGGT
TGCTGTCAAGAGGATCTCGAGAGGGTCTAAGCAAGGGAAAAAGGAATACATAACCGAAG
TGAAGGTTATTAGTCGGTTAAGACATAGAAATCTTGTGCAGCTAATTGGTTGGTGTCATG
ACCAAGGTAAGTTCTTACTTGTATATGAATTCATGCCAAATGGTAGCTTAGATTGCCACTT
ATTTGGTAAAAGGAATCCTCTTAGTTGGAGTACGAGGTATAAGATATTACTCGGATTAGC
ATCTGCTTTGCTCTATCTACACGAAGAATGGGAGCAGTGTGTGATCCACAGAGACATCAA
ATCGAGTAATGTAATGCTAGATTCAAGTTTTAATGTCAAGCTCGGTGATTTTGGCTTAGCT
AGACTAATGGACCATGAATTAGGTCCTCAGACTACAGGTTTGGCTGGAACTTTAGGTTAT
TTGGCTCCTGAATACATAAAAACAGGCCGAGCAAGTAAAGAGTCAGACGTATACAGCTTC
GGAGTAGTTGCACTAGAAATTGCAACCGGAAGAAAATCAGTTGATCCGGGTACGCGGAA
ATCTAATGTAGGGTTAGTGGAGTATGTTTGGGACCTTTATGGTAAAGGACAACTTCTTTC
TGCTGTTGATGAGAAATTAAACCTGGTTTTTGACGCAAAACAAGTAGAGCTATTGATGATT
ACCGGGTTATGGTGTGCTCATCCGGAGAGCAATCTGAGGCCATCTATAAGACAAGCAAT
TCATGTTCTGAATTTTGAAGCATCATTGCCTAATATACCAATAAAGATGCCAGTTCCTGTG
TATTATTTACCTAGTTCTGGTGAACCAGGAGTTAGCTCGGGGGATCCTACAATGACTTAC

ACAAGCATTGATGTGGGCCGTTAA

>NbC25369236g0004.1 GenBank ID: KT225316
MEKARGELADFTTHFSFVIDSNGNNSFADGLAFFLAPVGSSIPIGSAGNGLGLAKAELEVAHP
LSHLLLSSSIHFSIPGTHCIYMNNITPGKKNDAWISYNASSHTLKVVFTGFSNNKYNKDSLSLLI
DLRNYLPENVTFGFSASTGQLFQKKNNVKSWDFNSSLSLEHAEQPSANPPIQVLNPNDPPIT
QVQDPKFRPPREVSTRKKGNKGLVVGSSIGLPILLFVLITASCFLWKKKRKRNDKDHVFIDLS
MDNEFQKDRGPKKFSYGELARATNNFAEGQKLGEGGFGEVYEGLLKECKSYVAVKKVSSG
SKQGIKEYASEVKIISRLRHRNLVQLIGWCHEKGQLHLVYELMPNGKLR

>NbC25369236g0004.1 GenBank ID: KT225316
ATGGAAAAAGCTAGAGGAGAATTGGCAGATTTTACTACACATTTCTCCTTTGTTATTGATT
CAAATGGTAACAATAGCTTTGCTGATGGACTCGCCTTTTTCTTGGCTCCTGTGGGTTCCA
GTATCCCAATTGGTTCAGCTGGCAACGGCCTTGGTCTTGCCAAGGCCGAGTTAGAAGTA
GCTCATCCGCTGAGCCATTTGTTGCTATCGAGTTCGATACATTTCTCAATACCTGGGACC
CACTGTATTTACATGTTGGTATCAATTTAAATTCTATGAAATCTGCGGTTACTCTAAGTAT
GGAGGAATAACATTACACCAGGGAAGAAAAATGATGCTTGGATTAGTTACAATGCTAGTT
CCCATACTCTTAAAGTTGTTTTCACTGGATTTTCAAATAACAAATATAACAAGGACAGCCT
TAGTTTATTGATTGATCTGAGGAATTATTTGCCTGAGAATGTTACTTTTGGCTTCTCAGCA
TCAACAGGACAATTGTTTCAGAAAAAAAACAATGTCAAGTCTTGGGATTTCAATTCAAGTT
TGAGTTTAGAACATGCTGAACAGCCTAGTGCAAATCCTCCCATTCAAGTTCTAAATCCAA
ATGATCCTCCTATTACTCAAGTTCAAGATCCAAAGTTTCGTCCTCCTCGAGAAGTCAGCA
CCAGGAAAAAAGGAAACAAGGGACTAGTAGTTGGATCAAGCATAGGTTTACCTATTCTGC
TTTTCGTATTGATTACTGCCAGCTGCTTTTTATGGAAGAAAAAGAGGAAAAGGAATGATAA
AGACCATGTTTTTATTGATCTCAGTATGGACAATGAATTTCAGAAGGATAGAGGCCCTAA
GAAGTTTTCATATGGTGAATTAGCTCGTGCAACGAACAACTTTGCTGAGGGACAGAAGCT
TGGAGAAGGTGGATTTGGCGAAGTTTATGAAGGTTTATTGAAGGAATGTAAATCATATGT
TGCTGTTAAGAAAGTATCAAGCGGTTCTAAACAAGGAATAAAGGAGTATGCATCAGAAGT
GAAGATCATAAGCCGATTAAGACATAGAAATTTGGTTCAACTTATTGGTTGGTGTCACGA
GAAAGGGCAGTTGCATCTTGTTTATGAATTGATGCCTAATGGAAAGCTTAGATAA

>NbS00000562g0002.1 GenBank ID: KT225317
MVFSKILNILLFLILFLIPSFYSLNFNLSSINPSDANRSINVTGDAYISKQGIQVTPDERNVALGR
KTGRANFIEPLQLWNKATGELSDFTTHFSFVIDSNGNDSFADGLAFFLAPVGSSIPIGSAGNG
LGLAKAELEGSSSPEPFVAIEFDTFLNTWDPQYLHVGINVNSMESAVAQVWRNDITLGKKND
AWICYDASSHTLKVVFTGFSNNKYNKDSLSLLIDLRNYLPENVTFGFSASTGQLFQKNNVKS
WDFNSSMSFDPNKVPEHAEPPSANPPITQVTPPSNQEISTRKKGNKGLVVGSSIGLPILVIVLI
TGSCFLWKKKSKRNDKDHVFIDLSMDNEFQKGTGPKKFSYGELARATNNFAEVQKLGEGGF
GEVYEGLLKECKSYVAVKRVSSGSKQGIKEYASEVMIISRLRHRNLVQLIGWCHEKGKLHLV
YELMPNGSLDKHLFKEKSLLVWEIRWKIVQGLASALLYLHEEWEQCVVHRDVKASNVMLDS
NFNAKLGDFGLARLVDHEKGSQTTMLAGTVGYMAPECVMNGKASKESDVYSFGIVALEIAS
GRKSIDIKAAEDQVRLVEWIWNLYGIGKLVEATDPRLNKIYNEQQMERLMVLGLWCAHPDNK
LRPSIRQAIHVLNSEVQLPILPSRMPVATYSPPPLNMFSPPFSHTYETKTVGSEQEQTMTYTY
NTKSSSVYTSSAASSTKSLL

>NbS00000562g0002.1 GenBank ID: KT225317
ATGGTTTTCAGCAAAATTTTGAACATCTTGCTATTCTTAATACTTTTTCTTATCCCTTCTTT
CTATTCATTGAACTTTAACCTCTCCAGCATCAATCCTTCTGATGCTAATCGTTCTATCAAT
GTCACTGGAGACGCCTATATATCGAAACAAGGCATCCAAGTCACCCCTGATGAACGTAA
CGTGGCATTAGGTAGAAAAACTGGTCGAGCTAATTTTATTGAGCCGCTGCAATTATGGAA
TAAGGCTACAGGAGAATTGTCAGATTTTACTACGCATTTCTCCTTTGTTATTGATTCCAAT
GGTAACGATAGCTTTGCTGATGGACTCGCCTTTTTCTTGGCTCCTGTCGGTTCCAGTATC
CCTATTGGTTCAGCTGGTAACGGCCTTGGTCTTGCCAAGGCCGAGTTAGAAGGCAGCTC
ATCCCCTGAGCCATTTGTTGCTATCGAGTTCGATACATTTCTCAATACCTGGGACCCACA
GTATTTACATGTTGGTATCAATGTAAATTCTATGGAATCTGCTGTTGCTCAAGTATGGAGG
AATGACATTACGCTGGGGAAGAAAAATGATGCTTGGATTTGTTACGATGCTAGTTCACAT
ACTCTTAAAGTTGTTTTCACTGGATTTTCAAACAATAAATATAACAAGGATAGCCTTAGTTT
ATTGATTGATCTGAGGAATTATTTGCCGGAGAATGTTACTTTTGGCTTCTCAGCATCAACT
GGACAATTGTTTCAGAAAAACAATGTCAAGTCTTGGGATTTCAATTCAAGTATGAGTTTTG

ATCCAAACAAAGTTCCTGAACATGCTGAACCGCCGAGTGCAAATCCTCCTATTACTCAAG
TTACTCCTCCTTCTAACCAAGAAATCAGCACCAGAAAAAAAGGAAATAAGGGACTAGTCG
TTGGATCGAGCATAGGTTTGCCTATTCTGGTTATCGTATTGATTACTGGTAGCTGCTTTTT
ATGGAAGAAAAAGAGTAAAAGGAATGATAAAGACCATGTTTTTATTGATCTCAGTATGGA
CAATGAATTTCAAAAGGGTACTGGCCCTAAGAAGTTTTCCTATGGTGAATTAGCTCGTGC
AACGAACAATTTTGCTGAGGTACAGAAGCTTGGAGAAGGTGGATTTGGCGAAGTTTATG
AAGGTTTATTGAAGGAATGTAAATCATATGTTGCTGTTAAGAGAGTATCAAGCGGTTCTAA
ACAAGGGATAAAGGAGTATGCATCAGAAGTGATGATCATCAGCCGATTAAGACACAGAA
ATTTGGTTCAACTTATCGGTTGGTGCCATGAGAAAGGGAAGCTGCATCTTGTTTATGAAT
TGATGCCTAATGGAAGCTTAGATAAACATCTTTTCAAAGAGAAGTCATTGTTGGTATGGG
AAATCAGGTGGAAAATTGTTCAAGGATTGGCCTCGGCTTTGCTCTATTTACACGAAGAGT
GGGAACAATGTGTAGTCCATAGGGACGTAAAAGCAAGTAACGTCATGTTGGATTCAAACT
TCAACGCTAAATTAGGCGATTTTGGTTTGGCTAGACTCGTTGATCATGAAAAAGGATCTC
AAACAACAATGCTGGCGGGGACTGTAGGGTATATGGCGCCTGAATGTGTTATGAATGGA
AAAGCTAGTAAGGAATCAGATGTGTACAGTTTTGGAATAGTGGCATTGGAAATAGCAAGT
GGAAGAAAATCAATAGATATTAAAGCAGCAGAAGATCAAGTGAGATTGGTGGAATGGATT
TGGAATCTCTACGGAATTGGAAAGCTAGTTGAAGCAACTGATCCAAGATTGAATAAGATA
TATAATGAGCAACAAATGGAGCGTTTAATGGTTCTCGGTCTCTGGTGTGCTCATCCAGAC
AACAAACTCAGGCCATCAATAAGGCAAGCAATCCATGTTCTTAATTCTGAAGTTCAGCTA
CCTATTCTTCCCTCAAGAATGCCTGTTGCGACGTATTCTCCCCCTCCATTGAATATGTTTT
CGCCTCCATTTTCACATACTTATGAAACTAAAACAGTGGGGAGTGAGCAGGAACAGACTA
TGACTTACACTTATAACACTAAGTCCTCAAGTGTTTATACATCCTCAGCTGCTTCGTCAAC
AAAATCACTTTTGTAA

>NbS00048421g0010.1
MVGFASISALKYYLVFLLIIIPFVTSLSFNFDSFNPNGQNVTYEEDAFSANGVIQLTKNQLDRG
SGVSIGRATYFKTLHLWDKALTDFITHFSFSINSQGRTAYGDGLAFFLAPAGSRIPENTTIGGS
LGLTIDTQQLNTSNNHFVAVEFDTFKNWYDPQGDHVGIDINSMQSVVNLTWFSSIPNGNRTD
AWISYNSTSKNLTVVFTGFRANSTVTVQQSLSHNLDLREYLPERVTFGFTGATGGLFALQSIS
SWNFTSSLEINDNITDPGVALPMPKPEETPSKSKLGLVIGLVSGGCVLVAVSVLALFVFWRKR
KLREDEDEDDDDSIDGSMADEFERSTGPKKFLYSELARCTNNFAQEEKLGEGGFGGVYKGY
LRESNSYVAVKRVSRGSRQGVKEFASEVRIISRLRHRHLVQLIGWCHEKRELLLVYEFMPNG
SLDFHLFKGKSHLTWPIRYKIAQGLASALLYLHEEWEQCVVHRDIKSSNIMLDSNFNAKLGDF
GLARLVDHEKGSQTTVLADSNCRPSIRQAIHVLNFEAPLPTLPPNMPVPTYCSPSKHLSSASF
SSSYDTNGSRITEIQNSVTRDYTDSSNNTAASATSSPSASLLYSR

>NbS00048421g0010.1 Corrected; GenBank ID: KT225318
MVGFASISALKYYLVFLLIIIPFVTSLSFNFDSFNPNGQNVTYEEDAFSANGVIQLTKNQLDRG
SGVSIGRATYFKTLHLWDKALTDFITHFSFSINSQGRTAYGDGLAFFLAPAGSRIPENTTIGGS
LGLTIDTQQLNTSNNHFVAVEFDTFKNWYDPQGDHVGIDINSMQSVVNLTWFSSIPNGNRTD
AWISYNSTSKNLTVVFTGFRANSTVTVQQSLSHNLDLREYLPERVTFGFTGATGGLFALQSIS
SWNFTSSLEINDNITDPGVALPMPKPEETPSKSKLGLVIGLVSGGCVLVAVSVLALFVFWRKR
KLREDEDEDDDDSIDGSMADEFERSTGPKKFLYSELARCTNNFAQEEKLGEGGFGGVYKGY
LRESNSYVAVKRVSRGSRQGVKEFASEVRIISRLRHRHLVQLIGWCHEKRELLLVYEFMPNG
SLDFHLFKGKSHLTWPIRYKIAQGLASALLYLHEEWEQCVVHRDIKSSNIMLDSNFNAKLGDF
GLARLVDHEKGSQTTVLAGTMGYMAPECATTGKASKETDVYSFGIVALEIACGRRPIDRKAE
SDRQVNIVEWVWSLYGMGNLNEAADHKLSLEFNEMEMKHLLIVGLWCAHPDSNCRPSIRQA
IHVLNFEAPLPTLPPNMPVPTYCSPSKHLSSASFSSSYDTNGSRITEIQNSVTRDYTDSSNNT
AASATSSPSASLLYSR

>NbS00048421g0010.1 Corrected; GenBank ID: KT225318
ATGGTTGGTTTTGCCTCTATATCTGCTCTAAAATACTACCTTGTTTTTCTTCTCATCATAAT
CCCATTTGTTACTTCACTATCCTTCAATTTTGATAGTTTTAATCCCAATGGTCAGAATGTAA
CATATGAGGAAGATGCTTTTTCAGCAAATGGAGTAATTCAACTCACCAAAAACCAGCTTG
ATCGTGGTTCAGGAGTCAGCATAGGTCGAGCCACATATTTCAAGACACTGCATCTTTGG
GACAAGGCTTCTGGAAATCTCACAGATTTTATTACTCATTTCTCCTTTAGCATCAATTCAC
AGGGCAGAACAGCTTATGGTGACGGTCTTGCCTTCTTCCTCGCGCCTGCAGGTTCAAGA
ATTCCTGAAAACACAACCATAGGTGGCAGCCTTGGCCTTACAATTGATACTCAGCAACTT
AATACATCGAATAATCATTTTGTTGCAGTGGAGTTTGACACCTTTAAGAATTGGTATGATC
CACAGGGTGATCATGTAGGTATCGATATCAACTCTATGCAATCTGTTGTTAATTTGACCT

GGTTTAGTAGCATTCCAAATGGTAATAGAACTGACGCCTGGATTAGCTATAACTCAACCT
CGAAAAATCTTACTGTTGTCTTCACTGGTTTTAGAGCTAATTCCACTGTCACTGTCCAGCA
AAGCCTATCTCACAATCTTGATCTGAGGGAATATTTGCCAGAACGGGTCACTTTTGGCTT
CACAGGTGCAACAGGAGGTCTCTTTGCGTTACAAAGCATCTCCTCTTGGAATTTTACGTC
TTCTTTAGAAATTAATGACAACATAACAGATCCAGGGGTAGCCTTACCAATGCCAAAGCC
AGAGGAGACTCCAAGCAAAAGTAAGCTAGGACTTGTGATTGGATTAGTATCTGGTGGTT
GTGTTTTGGTTGCAGTATCTGTTTTGGCGTTGTTTGTATTTTGGAGAAAGAGGAAGTTGA
GAGAAGATGAAGATGAAGATGATGATGATAGCATTGATGGTTCCATGGCTGATGAATTTG
AAAGAAGTACAGGACCGAAGAAGTTCCTCTACAGTGAGTTGGCTAGATGTACAAATAACT
TTGCGCAGGAAGAGAAGCTTGGGGAGGGTGGATTCGGGGGTGTTTATAAAGGATATCTC
AGGGAATCCAATTCCTATGTTGCTGTTAAAAGGGTTTCAAGGGGGTCAAGGCAAGGAGT
AAAAGAGTTCGCATCAGAAGTGAGGATCATTAGCCGGTTAAGACATAGACATTTGGTGCA
ACTCATTGGTTGGTGCCATGAGAAAAGAGAACTTCTACTTGTTTATGAGTTTATGCCTAAT
GGAAGCTTAGATTTCCATCTTTTCAAAGGAAAAAGCCATTTGACATGGCCAATAAGATAC
AAGATTGCTCAAGGCTTGGCCTCAGCGTTGTTATATCTACACGAAGAATGGGAACAATGT
GTGGTGCATAGGGACATAAAGTCGAGCAATATTATGTTGGATTCCAATTTCAATGCCAAA
CTTGGGGATTTTGGTTTAGCTAGACTAGTTGACCATGAAAAGGGATCCCAAACAACAGTT
TTGGCAGGTACAATGGGCTACATGGCCCCCGAATGTGCTACCACTGGGAAAGCTAGCAA
GGAAACAGATGTCTATAGCTTTGGTATCGTCGCCTTAGAAATAGCTTGTGGAAGAAGACC
TATTGACCGTAAAGCTGAATCAGATCGTCAAGTAAACATTGTTGAGTGGGTTTGGAGCCT
TTATGGGATGGGAAATCTTAACGAAGCAGCTGATCATAAACTCTCACTAGAGTTCAATGA
AATGGAGATGAAGCACTTGTTAATTGTTGGATTATGGTGTGCTCATCCAGATAGCAATTG
TAGGCCTTCTATTAGGCAAGCAATTCATGTCCTTAATTTTGAAGCTCCATTGCCCACCCT
CCCTCCAAACATGCCTGTTCCAACATATTGCAGTCCATCAAAACATTTATCAAGTGCTTCA
TTTTCATCATCATATGACACCAACGGATCTCGGATTACTGAAATACAGAACTCAGTAACCA
GAGACTACACTGATTCTTCAAATAACACAGCAGCTTCCGCTACATCTTCACCTTCAGCAT
CACTTCTATACTCACGTTGA

>NbS00051756g0005.1
MVVFASLSALKYHVFLLIIIPFATSLSFNFDSFRPSDQNITYERDAYPANGAIQLTTDLINRDINA
TIGRATYSKLLHLWDKAVTDFSTHFSFSINSQGRARYGDGLAFFLAPVGSRIPENTTRGGSLG
LTSNTRRLNTSSNHFVAVEFDTYQNVQYDPKGDHVGIDINSMQSVVNVTWFSSIPSGRRTYA
WISYNSTSKNLTVVFTGFKLQGNTTVTVLQSLSHNLDLREYLPEWVTFGFTGGAGIVFALQSI
YSWNFTSSLEINDNITIPGVVLPSPKPEHAQSKNKLGLVVGLVSGGCVLVFVCVLMLFAYWR
KRKLREDEDNDIFDGSMTHEFERSTGPKKFLYKELARCTKSFAHEKKLGEGGFGGVYKGFL
RESNSYVAVKRISRESKQGIKEYASEVRIISRLRHRHLVQLIGWCHEKRELLLVYEFMPNGSL
DFHLFKGKIQLTWPIRYKIAQGLASALLYLHEEWEQCVVHRDIKSSNIMLDSNFNAKLGDFGL
ARLVDHEKGSQTTVLADSKCRPSIRQAIHVLNFEAPLPTLPPNMPVPTYCSPSQLLPTSSLSS
SYETNGHEIIVAQIQYSVNRDNTDSSNNTTASAASSSSASMLYP

>NbS00051756g0005.1 Corrected; GenBank ID: KT225319
MVVFASLSALKYHVFLLIIIPFATSLSFNFDSFRPSDQNITYERDAYPANGAIQLTTDLINRDINA
TIGRATYSKLLHLWDKAVTDFSTHFSFSINSQGRARYGDGLAFFLAPVGSRIPENTTRGGSLG
LTSNTRRLNTSSNHFVAVEFDTYQNVQYDPKGDHVGIDINSMQSVVNVTWFSSIPSGRRTYA
WISYNSTSKNLTVVFTGFKLQGNTTVTVLQSLSHNLDLREYLPEWVTFGFTGGAGIVFALQSI
YSWNFTSSLEINDNITIPGVVLPSPKPEHAQSKNKLGLVVGLVSGGCVLVFVCVLMLFAYWR
KRKLREDEDNDIFDGSMTHEFERSTGPKKFLYKELARCTKSFAHEKKLGEGGFGGVYKGFL
RESNSYVAVKRISRESKQGIKEYASEVRIISRLRHRHLVQLIGWCHEKRELLLVYEFMPNGSL
DFHLFKGKIQLTWPIRYKIAQGLASALLYLHEEWEQCVVHRDIKSSNIMLDSNFNAKLGDFGL
ARLVDHEKGSQTTVLAGTRGYMAPECFITGKASKETDVYSFGVVALEIACGRKAIDPKAEEH
QENLVDWVWSLYGMGNLNEAADPKLSSEFNEMEMKHLLIVGLWCAHPDSKCRPSIRQAIHV
LNFEAPLPTLPPNMPVPTYCSPSQLLPTSSLSSSYETNGHEIIVAQIQYSVNRDNTDSSNNTT
ASAASSSSASMLYPR

>NbS00051756g0005.1 Corrected; GenBank ID: KT225319
ATGGTTGTTTTTGCCTCTTTATCTGCTCTCAAATACCATGTTTTTCTTCTTATCATAATCCC
CTTTGCGACTTCGCTGTCCTTCAATTTTGATAGTTTTAGACCCAGCGATCAAAATATAACA
TACGAGAGAGATGCTTATCCAGCAAATGGTGCAATTCAACTTACCACAGACCTTATCAAT
CGTGATATAAACGCAACTATAGGTCGAGCCACATATTCCAAGCTTCTGCATCTTTGGGAC
AAGGCCTCAGGAAATGTCACAGATTTCAGTACTCACTTCTCCTTTAGCATCAATTCACAA

GGCAGAGCAAGATATGGTGATGGTCTTGCCTTCTTCCTCGCACCTGTAGGTTCAAGAATT
CCTGAGAACACAACCAGAGGTGGCAGTCTTGGCCTTACAAGTAATACTCGACGACTGAA
TACATCGAGCAATCATTTTGTAGCTGTGGAGTTTGATACGTATCAGAACGTCCAGTATGA
CCCAAAGGGTGATCATGTAGGTATTGATATCAACTCTATGCAATCTGTTGTTAATGTGAC
CTGGTTTAGTAGCATTCCGAGTGGTAGGAGAACTTATGCCTGGATTAGCTATAACTCGAC
TTCAAAAAATCTTACTGTTGTCTTCACTGGTTTCAAACTTCAAGGAAATACCACTGTCACG
GTCCTGCAAAGCCTATCTCATAATCTTGATCTGAGAGAATACTTGCCTGAATGGGTCACT
TTTGGCTTCACAGGTGGAGCTGGAATTGTCTTTGCATTACAAAGTATCTATTCTTGGAACT
TTACTTCTTCATTAGAAATTAACGACAACATAACAATTCCAGGAGTAGTCTTACCAAGCCC
CAAGCCAGAGCATGCACAGAGTAAAAACAAGTTAGGACTTGTGGTTGGATTGGTTTCTG
GTGGTTGTGTTTTGGTTTTTGTATGTGTTTTGATGTTGTTTGCATATTGGAGAAAGAGGAA
GCTGAGAGAAGATGAAGACAATGACATCTTTGATGGATCCATGACTCATGAATTTGAAAG
AAGCACAGGACCAAAGAAGTTCCTTTACAAAGAGTTGGCTAGATGTACAAAAAGCTTTGC
GCATGAAAAGAAGCTCGGGGAGGGTGGGTTTGGAGGTGTTTATAAAGGATTTCTCAGGG
AATCCAATTCCTATGTTGCTGTTAAGAGGATTTCAAGGGAGTCAAAGCAAGGAATAAAAG
AGTATGCATCAGAAGTGAGGATCATCAGCCGGCTAAGACATAGACATCTGGTGCAACTC
ATTGGCTGGTGTCATGAGAAAAGAGAACTACTACTTGTCTATGAGTTTATGCCTAACGGA
AGCCTAGATTTCCATCTTTTCAAGGGAAAAATCCAGTTGACATGGCCAATAAGATACAAG
ATTGCTCAAGGCTTGGCCTCAGCATTGCTATATCTACACGAAGAATGGGAACAATGTGTG
GTGCATAGGGACATAAAGTCAAGCAATATTATGTTGGATTCCAATTTCAATGCAAAACTTG
GGGATTTTGGTTTAGCTAGACTAGTTGACCATGAAAAGGGATCCCAAACAACAGTTTTGG
CAGGTACAAGGGGCTACATGGCCCCGGAATGTTTCATCACTGGCAAAGCGAGCAAGGA
AACAGATGTCTATAGCTTTGGTGTTGTCGCGTTAGAAATAGCTTGTGGAAGGAAAGCTAT
TGATCCTAAAGCTGAAGAACATCAAGAAAACCTTGTTGATTGGGTTTGGAGCCTTTACGG
GATGGGAAATCTTAATGAAGCAGCTGATCCTAAACTCTCATCAGAGTTCAATGAAATGGA
GATGAAGCACTTGTTAATTGTTGGCTTATGGTGTGCTCATCCAGATAGCAAATGCAGGCC
TTCTATTAGGCAAGCAATTCACGTCCTTAATTTTGAAGCTCCATTGCCCACCCTCCCTCC
AAACATGCCTGTGCCAACATATTGCAGTCCATCACAACTACTACCAACTTCTTCATTGTCA
TCATCGTATGAAACCAATGGTCATGAGATCATTGTCGCCCAGATCCAGTATTCAGTGAAC
AGAGACAACACTGATTCTTCGAACAACACAACAGCTTCTGCAGCGTCTTCATCTTCAGCT
TCAATGTTGTACCCACGTTGA

>NbS00037263g0008.1
YSNGRNLTCWGSVTAANETLNLTPDQPQNNSNKVGRVLFSQSLPIWPASFSTIFTIRILTNQS
ISGDGMAFLIAQDDKPSPPDSYGSFIGILDPSTQGGALHQLAVEFDTYRNEGEIDGDHFAIVTT
NMESPVAVKSLNAIGIDLKSGRNITIKIDYDGWAKVLDISAAYAGQTPVKFLSQEIIMQETVPQ
NAYVGFSASTAYFSELHQVLNWNFTLYELPERSLKYGPDPEKEKIALLVAIPVVVVSLAVTVS
FLIVARKDRKERLQRKEDIEMLTRTAANAPQMFTYRRLSNATKNFSKDNLLGTGGFGSVYKG
VFSDPPTTIAVKRTNATSNQDGILAEICTIGRLRHKNLVQLHGWCHDREKLLLIYEYMPNGSL
DKYIGKIFLDWDTRFKILSGLASALVYLHEECGNPIVHRDVKPNNVMLDSEYNAHLGDFGLAR
LLQNENFVTTMVAGTPGYLAPEVSYTGRATPESDVYSFGMVVLEVVCGRRSKGIMEENSLV
ECVWSSYEKGALLECMDQKLDGNFDNAEAMRCLITGLACLHPDSILRPKMRKVVQVFMNPD
EPLMKLPESRPSVVCVSWNSCTCSTMTIAEDYAPGGVNMEIIPDEVTVSYEYAYETKK

>NbS00037263g0008.1 Corrected; GenBank ID: KT225320
MLRLLIILVLLNQFSVLFVESYNFSFSSFDVGSYSNGRNLTCWGSVTAANETLNLTPDQPQNN
SNKVGRVLFSQSLPIWPASFSTIFTIRILTNQSISGDGMAFLIAQDDKPSPPDSYGSFIGILDPS
TQGGALHQLAVEFDTYRNEGEIDGDHFAIVTTNMESPVAVKSLNAIGIDLKSGRNITIKIDYDG
WAKVLDISAAYAGQTPVKFLSQEIIMQETVPQNAYVGFSASTAYFSELHQVLNWNFTLYELPE
RSLKYGPDPEKEKIALLVAIPVVVVSLAVTVSFLIVARKDRKERLQRKEDIEMLTRTAANAPQM
FTYRRLSNATKNFSKDNLLGTGGFGSVYKGVFSDPPTTIAVKRTNATSNQDGILAEICTIGRL
RHKNLVQLHGWCHDREKLLLIYEYMPNGSLDKYIGKIFLDWDTRFKILSGLASALVYLHEECG
NPIVHRDVKPNNVMLDSEYNAHLGDFGLARLLQNENFVTTMVAGTPGYLAPEVSYTGRATP
ESDVYSFGMVVLEVVCGRRSKGIMEENSLVECVWSSYEKGALLECMDQKLDGNFDNAEAM
RCLITGLACLHPDSILRPKMRKVVQVFMNPDEPLMKLPESRPSVVCVSWNSCTCSTMTIAED
YAPGGVNMEIIPDEVTVSYEYAYETKK

>NbS00037263g0008.1 Corrected; GenBank ID: KT225320
ATGTTAAGGCTTCTAATTATTCTAGTCTTGCTTAATCAATTCTCAGTTCTATTTGTTGAATC
CTACAACTTTTCTTTTTCTTCATTTGATGTTGGTAGTTACAGTAACGGCAGAAACTTAACA

TGCTGGGGATCTGTCACTGCTGCTAATGAAACGCTTAACCTCACACCTGATCAGCCGCA
AAACAATTCTAACAAAGTTGGAAGAGTGTTGTTCAGTCAATCCTTACCTATATGGCCTGCT
TCTTTTTCCACCATATTCACTATAAGGATTTTGACAAATCAATCCATTTCTGGTGATGGAA
TGGCATTCCTCATTGCTCAAGACGATAAGCCTTCCCCACCGGATAGTTATGGCTCGTTTA
TTGGAATTCTTGATCCATCAACTCAAGGTCAGCTCCTAGTATATAAATTGGACTTAAAGTT
GGTTCTTTTTCAATTCTTGATCCATCACTTCTCAGACTTTCTTCTTTTAATATGCAGGGGG
CGCGCTTCATCAGCTTGCTGTGGAGTTTGATACATATAGAAATGAAGGCGAAATTGATGG
AGATCATTTTGCTATTGTGACTACAAATATGGAGAGTCCTGTGGCTGTTAAAAGCTTAAAT
GCTATTGGGATTGATCTAAAGAGTGGAAGAAATATCACAATCAAGATTGACTATGATGGA
TGGGCTAAAGTTCTTGATATTTCTGCAGCATATGCAGGACAAACTCCAGTGAAATTTCTA
AGCCAAGAAATCATCATGCAAGAAACAGTTCCACAAAATGCTTATGTTGGATTTTCAGCTT
CCACTGCTTATTTCTCAGAGTTACATCAAGTTCTCAACTGGAATTTCACATTGTATGAATT
ACCAGAAAGATCTCTAAAGTATGGCCCTGATCCAGAAAAGGAAAAGATAGCTCTACTGGT
TGCTATTCCTGTGGTAGTTGTCTCGTTAGCTGTGACTGTATCGTTTCTCATTGTAGCTCGT
AAGGATAGAAAAGAAAGACTTCAGAGGAAAGAGGACATTGAAATGCTAACAAGAACTGC
AGCCAATGCTCCTCAAATGTTTACTTACCGGAGACTTTCCAACGCTACTAAAAACTTCAG
CAAAGATAACCTATTGGGAACTGGAGGTTTTGGGAGCGTTTACAAAGGAGTGTTTTCTGA
TCCTCCAACAACTATAGCTGTTAAACGAACCAACGCAACATCTAATCAAGGTTTGTTTTCC
ATTAAGTTCCCCTAAATATAAGTCGATAGGCACTATTACTAAAATTTCAAATTACCTAGTAA
TATTTATAATTAAGCTGTTATGGTTTTTATACTAGGTGAGACGGAATTTTGGCTGAAATAT
GTACAATTGGGCGCCTAAGGCACAAAAACTTAGTGCAGTTACATGGTTGGTGCCATGAC
CGCGAGAAACTCCTATTAATATATGAGTATATGCCTAATGGAAGTCTTGATAAATACATTG
GCAAAATCTTTCTTGATTGGGATACCAGATTCAAGATTTTATCAGGATTGGCATCAGCACT
AGTGTATCTTCATGAAGAATGTGGAAATCCTATAGTACATCGAGACGTTAAGCCAAACAA
TGTGATGTTAGACTCTGAGTATAATGCTCACTTGGGCGATTTTGGGCTAGCAAGATTACT
CCAAAATGAGAACTTTGTTACAACAATGGTGGCTGGCACTCCAGGATACTTAGCCCCGG
AAGTTAGCTACACGGGGAGGGCTACCCCCGAATCTGATGTCTACAGCTTTGGTATGGTT
GTTTTAGAAGTGGTTTGTGGACGACGATCAAAAGGGATCATGGAAGAAAATAGCTTGGT
GGAATGTGTATGGAGTTCATATGAGAAAGGTGCATTATTGGAATGTATGGACCAAAAACT
TGACGGAAATTTTGATAATGCGGAAGCTATGAGGTGTTTGATCACTGGATTAGCATGTTT
ACACCCTGACAGTATTCTCCGGCCTAAAATGAGAAAAGTGGTGCAAGTGTTCATGAATCC
TGATGAGCCATTGATGAAATTGCCCGAGTCTCGGCCTAGTGTTGTTTGTGTGTCATGGAA
TTCTTGTACGTGTTCAACGATGACAATAGCAGAAGACTATGCCCCTGGTGGCGTTAACAT
GGAGATCATTCCCGATGAGGTGACAGTCTCATATGAATATGCTTATGAAACAAAGAAATA
G

>NbS00001559g0001.1 GenBank ID: KT225321
MLPILFILINLTIFQSISALDFLFNSFTTNSTPVLHLIDDARLEPPVIRLTNDSNQFSRGRAFYPSP
IPIRSTSNSTSISSSFSTQFIFSILPDDPSSPGFGLAFVLSASTSPPNALSSQYFGLFTNATVHT
VAPLLAVEFDTGRNPEFNDPDRNHVGIDLNRIESVVTQTAGYYNSGTDSFVPLNMRSGQNIH
VWIEFNGPEFEIDVTIAPAGMSRPAKTMLSYKSPIIANYTSADMFVGFSASKTQWIEAQRLLA
WSFSDSGVARDINTTNLPVFQLENSTSSSLSSGAIAGIVIGCVVAVLGCLCVFYFFWWKKRG
KEEEDVIEDWELEYWPHRFSYEELNLATKGFSKDELLGAGGFGKVYKGILSNNTEVAVKCVN
HDSRQGIREFMAEISTIGRLQHKNLVQMRGWCRKGNELMIVYDYMPNGSLNKWIFDKPEKV
MNWLDRRKVLTDVAEGLNYLHHGWEQVVVHRDIKSSNVLLDCEMRGRLGDFGLAKLYTHG
GVPNTTRVVGTLGYLAPEVVTRATPTAASDVYSFGVVVLEVACGRRPIDAGFMVEEEEVLID
WVRQKYREGRLCEAADDRIRGQYSEDEMEAMLKLGLTCCHPDPLRRPTMREVVAVLLGEN
VEATQNELLAELTPTVSSMKDRSNSASDEGDKYLSQESELLSAV

>NbS00001559g0001.1 GenBank ID: KT225321
ATGCTACCAATACTGTTCATTCTCATCAATCTTACAATTTTCCAATCTATTTCTGCTCTCGA
TTTTCTCTTCAATTCCTTCACTACCAACTCAACCCCAGTCCTACACCTCATCGACGATGCT
CGCCTTGAGCCACCAGTGATCCGCCTCACCAACGACTCCAACCAATTTTCCCGTGGCCG
TGCTTTTTACCCATCTCCAATTCCTATCAGATCAACTTCCAATTCTACTTCCATTTCCTCCT
CTTTCTCTACCCAATTCATCTTCTCTATTCTCCCCGATGATCCCTCTAGTCCTGGTTTCGG
CCTTGCTTTTGTTCTTTCAGCTTCCACTTCCCCACCCAACGCTCTCTCAAGTCAATACTTT
GGACTTTTCACTAACGCCACCGTTCATACCGTTGCCCCACTTCTCGCCGTCGAGTTTGAT
ACGGGTCGTAACCCGGAATTCAATGATCCGGATAGAAACCATGTCGGGATTGATCTTAA
CAGAATCGAATCTGTTGTTACTCAAACGGCTGGATATTATAATTCTTCTGGTAATGGTACT
GATTCTTTCGTGCCTCTAAATATGCGAAGTGGTCAAAATATTCATGTTTGGATCGAGTTCA

ATGGGCCTGAATTCGAGATTGATGTTACTATAGCTCCAGCTGGTATGTCACGGCCTGCTA
AGACTATGTTAAGTTATAAAAGCCCCATAATTGCAAATTACACATCTGCTGATATGTTTGT
CGGGTTCTCTGCTTCCAAGACTCAATGGATTGAGGCACAAAGACTTTTAGCTTGGAGTTT
TAGTGATTCTGGAGTTGCAAGGGATATTAATACTACGAATTTGCCTGTTTTTCAGCTGGA
GAATTCGACGTCTTCTTCGTTATCTTCCGGTGCTATTGCTGGAATTGTAATTGGTTGTGTA
GTGGCAGTGTTAGGTTGTTTGTGTGTATTTTATTTTTTTTGGTGGAAGAAAAGGGGTAAG
GAGGAAGAGGATGTAATTGAAGATTGGGAACTTGAGTATTGGCCTCATAGATTTTCATAC
GAAGAGCTTAACCTAGCTACAAAAGGGTTCTCTAAGGATGAGCTACTTGGGGCTGGTGG
ATTTGGTAAAGTATATAAGGGAATACTATCTAATAACACAGAAGTGGCAGTGAAATGTGT
GAACCATGACTCAAGACAAGGAATAAGAGAATTCATGGCTGAGATATCGACTATCGGAA
GGCTTCAACACAAGAATTTAGTACAAATGAGAGGGTGGTGTAGGAAGGGAAATGAACTT
ATGATTGTGTATGATTATATGCCTAATGGAAGTCTGAATAAATGGATATTTGATAAGCCAG
AGAAGGTTATGAATTGGCTAGACAGGAGGAAGGTCCTAACTGATGTTGCTGAGGGTTTA
AACTATTTACATCATGGTTGGGAACAAGTGGTTGTACATAGGGATATTAAATCTAGCAAT
GTCTTGTTAGATTGTGAAATGAGAGGGAGACTGGGAGATTTCGGGCTAGCAAAGTTATA
CACTCACGGTGGTGTACCAAATACAACTAGGGTAGTAGGTACATTAGGGTACTTGGCAC
CTGAAGTTGTGACAAGGGCTACACCAACTGCAGCTAGTGATGTTTATAGTTTTGGGGTG
GTGGTGTTGGAAGTGGCATGCGGGCGTAGGCCGATTGACGCAGGGTTTATGGTGGAGG
AAGAGGAAGTGTTGATTGATTGGGTTAGACAAAAGTACAGGGAAGGGAGATTGTGTGAG
GCAGCAGATGATAGGATTAGGGGGCAGTATTCGGAGGACGAAATGGAAGCTATGTTGAA
ACTAGGCCTAACTTGTTGTCACCCGGATCCTCTCCGACGACCTACTATGAGAGAGGTGG
TTGCTGTATTGCTTGGTGAGAATGTGGAGGCAACACAAAATGAACTGCTAGCTGAATTAA
CACCCACTGTCAGCAGCATGAAAGATAGAAGCAATAGTGCAAGTGACGAAGGAGATAAA
TACTTGTCGCAAGAATCGGAACTACTATCGGCAGTGTAG

>NbS00015570g0009.1
MSRTVFSEHEFVYNGFNGIAASYLSVNGIAEIEKTGALRLTNETSRTVGHAFYKSAIKFKNFPN
RKVSSFSTAFAFGIVPEYAKLGGHGFAFTISRSKDMKGALPSQYLGLLNSSDIGNFSNHLFAV
EFDTVQDFEFGDISDNHVGIDINNLKSNASCGKTIQAWIDYDSNRNVLNVTLSLSSVKPNYSIL
SFPVDLSPILEEFMYVGFSASTGLLASSHYIFGWSFKMNGKAQSLDLDLLPNLPGPKKDQTTL
IVATSLSAVVFLAFGVVLALYIIWKIKNMDEIEPWELEIGPHRFSYKELKKATRDFRDKELLGFG
GFGRVYKGTLPKTSTEAAVKRINHDAKQGLQEFVSEIATIGRLRHRNLVQLIGWCRRKGDLLL
VYDFMPNGSLDKYIYDEPRIILTWDQRFKIIKGVASGLLYLHEEWEQTVIHRDIKAGNVLLDAE
MNGRLGDFGLAKLYEHGANPSTTRVVGTLGYLAPELTKTGKPTTSSDVFAFGALLLEVACGR
RPIEAKALPEELILVDWVWDKWKEGAILEVVDPRLNSKYDEMEAVVVLKLGLMCSNNTPSKR
PSMRLVVRYLEGEVALPEMLAAPDEYEGKKGGASGMGFEDFEHSYPSSSYFEKVSTWSSV
YDGEGDIDIEANAITPLTDSGREDSR

>NbS00015570g0009.1 Corrected; GenBank ID: KT225322
MAKNTSLIFFLVFLILMSRTVFSEHEFVYNGFNGIAASYLSVNGIAEIEKTGALRLTNETSRTVG
HAFYKSAIKFKNFPNRKVSSFSTAFAFGIVPEYAKLGGHGFAFTISRSKDMKGALPSQYLGLL
NSSDIGNFSNHLFAVEFDTVQDFEFGDISDNHVGIDINNLKSNASVNASYFSEGNSTKQKLFL
QCGKTIQAWIDYDSNRNVLNVTLSLSSVKPNYSILSFPVDLSPILEEFMYVGFSASTGLLASSH
YIFGWSFKMNGKAQSLDLDLLPNLPGPKKDQTTLIVATSLSAVVFLAFGVVLALYIIWKIKNMD
EIEPWELEIGPHRFSYKELKKATRDFRDKELLGFGGFGRVYKGTLPKTSTEAAVKRINHDAKQ
GLQEFVSEIATIGRLRHRNLVQLIGWCRRKGDLLLVYDFMPNGSLDKYIYDEPRIILTWDQRF
KIIKGVASGLLYLHEEWEQTVIHRDIKAGNVLLDAEMNGRLGDFGLAKLYEHGANPSTTRVVG
TLGYLAPELTKTGKPTTSSDVFAFGALLLEVACGRRPIEAKALPEELILVDWVWDKWKEGAIL
EVVDPRLNSKYDEMEAVVVLKLGLMCSNNTPSKRPSMRLVVRYLEGEVALPEMLAAPDEYE
GKKGGASGMGFEDFEHSYPSSSYFEKVSTWSSVYDGEGDIDIEANAITPLTDSGREDSR

>NbS00015570g0009.1 Corrected; GenBank ID: KT225322
ATGGCTAAAAATACTAGTCTAATATTTTTCTTGGTATTTCTTATTTTGATGTCAAGAACAGT
TTTTTCTGAACATGAATTTGTTTACAACGGTTTTAATGGTATTGCAGCAAGTTATTTAAGTG
TAAATGGTATAGCTGAGATTGAAAAAACTGGTGCTCTAAGGCTAACAAATGAAACATCAA
GAACAGTTGGTCATGCCTTTTACAAAAGTGCAATCAAATTCAAGAATTTCCCAAATAGGAA
AGTTTCTTCATTTTCTACTGCTTTTGCTTTTGGGATTGTTCCAGAATATGCTAAATTAGGT
GGCCATGGTTTTGCTTTCACAATTTCAAGGTCTAAAGATATGAAAGGTGCTCTTCCTAGT
CAGTATTTAGGTCTTTTAAATTCTAGTGATATTGGTAATTTTTCAAATCATTTATTTGCTGT
TGAATTTGATACTGTTCAAGATTTTGAGTTTGGTGATATTAGTGATAATCATGTTGGTATT

GATATCAATAATTTAAAGTCTAATGCTTCAGTTAATGCTAGCTATTTTTCTGAAGGAAATTC
TACTAAGCAAAAGCTTTTTCTTCAGTGTGGTAAGACAATTCAGGCTTGGATTGACTATGAT
TCAAATAGAAATGTACTGAATGTTACACTCTCACTATCTTCAGTAAAACCAAATTATTCAAT
TTTGTCATTTCCAGTTGACCTTTCACCAATTCTTGAGGAGTTTATGTATGTTGGATTTTCT
GCTTCTACTGGTTTGCTTGCTAGTTCACATTATATATTTGGTTGGAGTTTCAAAATGAATG
GAAAAGCTCAATCTTTGGATCTTGATTTATTGCCTAATTTACCTGGACCTAAGAAGGATCA
AACAACCTTAATTGTAGCGACGTCATTATCTGCTGTCGTCTTTCTTGCATTTGGTGTTGTT
TTGGCCTTGTACATCATTTGGAAAATCAAGAACATGGATGAGATTGAGCCTTGGGAACTT
GAAATAGGTCCACATAGGTTTTCGTATAAGGAGTTAAAGAAAGCTACAAGGGATTTTAGA
GATAAGGAGTTACTTGGATTTGGTGGATTTGGTCGAGTTTATAAAGGAACGTTGCCTAAG
ACTAGTACCGAGGCTGCTGTTAAGCGAATAAATCATGATGCAAAACAAGGTCTCCAAGAA
TTTGTGTCTGAAATCGCTACTATTGGTCGCCTTAGACATAGGAATTTGGTTCAGCTTATAG
GGTGGTGTCGACGAAAAGGTGACTTGTTACTTGTTTATGACTTTATGCCTAATGGAAGCT
TAGACAAGTACATTTATGATGAGCCAAGAATCATTTTAACGTGGGATCAGCGATTCAAGA
TTATCAAAGGTGTGGCTTCGGGGCTGTTGTATTTACATGAAGAATGGGAACAAACTGTAA
TCCATAGAGATATCAAAGCAGGGAATGTGTTATTAGATGCGGAAATGAATGGACGACTTG
GAGATTTTGGACTTGCCAAGTTATATGAGCACGGGGCAAATCCTAGCACAACACGAGTA
GTGGGCACGTTGGGTTACTTAGCTCCAGAATTAACCAAGACTGGAAAGCCTACAACAAG
CTCGGATGTTTTTGCCTTTGGTGCATTGCTGCTAGAAGTTGCTTGTGGAAGGAGGCCTAT
CGAGGCAAAAGCATTGCCCGAGGAGTTAATTTTAGTTGATTGGGTTTGGGATAAATGGAA
GGAAGGTGCCATTCTTGAAGTGGTTGATCCGAGGTTAAACAGCAAGTACGATGAGATGG
AGGCTGTGGTGGTGCTTAAATTAGGACTGATGTGTTCAAATAATACACCATCTAAGAGAC
CTAGCATGAGGTTAGTGGTGAGATACTTGGAGGGGGAAGTAGCTCTGCCGGAGATGCT
GGCAGCGCCCGATGAATATGAAGGGAAAAAAGGCGGCGCTAGTGGTATGGGGTTTGAG
GACTTTGAACATTCATATCCATCCTCGTCTTATTTTGAGAAAGTAAGTACCTGGTCATCGG
TTTATGATGGTGAAGGAGATATTGACATTGAAGCTAATGCGATAACGCCGTTAACGGATT
CTGGCAGAGAGGACAGCAGATAG

>NbS00002771g0001.1
MAKDIRLIFFLVFFILISRKVFSDQEFVYNGFNGIAASYLSVNGIAEIEKTGALRLTNETSRAVGH
AFYKSAIKFKNFPNRKVSSFSTAFAFGIVPEYAKLGGHGFAFTISRSKDMKGALPSQYLGLLN
SSDIGNFSNHLFAVEFDTVQDFEFGDISDNHVGIDINNLKSNASVNASCFSEGNSTKQKLFLQ
CGKTIQAWIDYDSNRNVLNVTLSLSSVKPNYSILSFPVDLSPILEEFMYVGFSASTATSLSAVV
FLAFGVILAFYIIWKIKNIDVIEPWELEIGPHRFGYKELKKSTRGFRDRELLGFGGFGRVYKGTL
PKTNMVVAVKRINHDAKQGLQEFVSEIATIGRLRHRNLVQLIGWCRQKGDLLLVYDFMPNGS
LDKYIYDEPRVILTWDQRFKIIKGVASGLLYLHEEWEQTVIHRDIKAGNVLLDAEMNGRLGDF
GLAKLYEHGANPSTTRVVGTLGYLAPELTKTGKPTTSSDVFAFGALLLEVTCGRRPIEAKALP
EELILVDWVWDKWKEGAILEVVDPKLNGEYDDMEAVVVLKLGLMCSNNTPSKRPSMRLVVR
YLEGEMVLPETLAAPDEYDGKKGGASGMGFEDFVHSYPSSSYFEKVSTWSPAYDGEGDIDI
EADPIKPLTDSGREDSR

>NbS00002771g0001.1 Corrected; GenBank ID: KT225323
MAKDIRLIFFLVFFILISRKVFSDQEFVYNGFNGIAASYLSVNGIAEIEKTGALRLTNETSRAVGH
AFYKSAIKFKNFPNRKVSSFSTAFAFGIVPEYAKLGGHGFAFTISRSKDMKGALPSQYLGLLN
SSDIGNFSNHLFAVEFDTVQDFEFGDISDNHVGIDINNLKSNASVNASCFSEGNSTKQKLFLQ
CGKTIQAWIDYDSNRNVLNVTLSLSSVKPNYSILSFPVDLSPILEEFMYVGFSASTGLLASSHY
IFGWSFKMNGKAQSLDLDLLPNLPGPKKNQTTLIAATSLSAVVFLAFGVILAFYIIWKIKNIDVIE
PWELEIGPHRFGYKELKKSTRGFRDRELLGFGGFGRVYKGTLPKTNMVVAVKRINHDAKQG
LQEFVSEIATIGRLRHRNLVQLIGWCRQKGDLLLVYDFMPNGSLDKYIYDEPRVILTWDQRFKI
IKGVASGLLYLHEEWEQTVIHRDIKAGNVLLDAEMNGRLGDFGLAKLYEHGANPSTTRVVGT
LGYLAPELTKTGKPTTSSDVFAFGALLLEVTCGRRPIEAKALPEELILVDWVWDKWKEGAILE
VVDPKLNGEYDDMEAVVVLKLGLMCSNNTPSKRPSMRLVVRYLEGEMVLPETLAAPDEYDG
KKGGASGMGFEDFVHSYPSSSYFEKVSTWSPAYDGEGDIDIEADPIKPLTDSGREDSR

>NbS00002771g0001.1 Corrected; GenBank ID: KT225323
ATGGCTAAAGATATTAGACTAATATTTTTCTTGGTATTTTTTATTTTGATATCAAGAAAAGT
GTTTTCTGATCAAGAATTTGTTTACAATGGTTTTAATGGTATTGCAGCTAGTTATTTAAGTG
TAAATGGTATAGCTGAGATAGAAAAAACTGGTGCTCTAAGGCTAACAAATGAAACATCAA
GAGCAGTTGGTCATGCTTTTTACAAAAGTGCAATCAAATTCAAGAATTTCCCAAATAGGAA
GGTTTCTTCATTTTCTACTGCTTTTGCTTTTGGGATTGTTCCAGAATATGCTAAATTAGGT

GGCCATGGTTTTGCTTTCACAATTTCAAGGTCTAAAGATATGAAAGGTGCTCTTCCTAGT
CAGTATTTAGGTCTTTTAAATTCAAGTGATATTGGTAATTTTTCAAATCATTTATTTGCTGT
AGAATTTGATACTGTTCAAGATTTTGAGTTTGGTGATATTAGTGATAATCATGTTGGTATT
GATATCAATAATTTAAAGTCTAATGCTTCAGTTAATGCTAGTTGCTTTTCTGAAGGAAATT
CTACTAAGCAGAAGCTTTTTCTTCAGTGTGGCAAGACAATTCAGGCTTGGATTGACTATG
ATTCAAATAGAAATGTACTGAATGTTACACTCTCGCTATCTTCAGTAAAACCAAATTATTC
AATTTTGTCATTTCCAGTTGACCTTTCACCAATTCTTGAGGAGTTTATGTATGTTGGATTTT
CTGCTTCTACTGGTTTGCTTGCTAGTTCACATTATATATTTGGTTGGAGTTTCAAAATGAA
TGGAAAAGCTCAATCTTTGGATCTTGATTTATTGCCTAATTTACCTGGACCTAAGAAGAAT
CAAACAACCTTAATTGCAGCGACGTCTTTATCTGCTGTCGTCTTTCTTGCATTTGGTGTTA
TTTTGGCGTTTTACATCATTTGGAAAATCAAGAACATAGATGTGATTGAGCCGTGGGAAC
TTGAAATAGGTCCACATAGGTTTGGATACAAGGAGTTAAAGAAATCTACAAGGGGTTTTA
GGGATAGGGAGTTACTTGGATTTGGTGGATTTGGTCGAGTTTATAAAGGAACGTTGCCTA
AGACTAATATGGTTGTTGCTGTTAAGCGAATAAATCATGATGCAAAACAAGGTCTCCAAG
AATTTGTGTCTGAAATCGCCACTATTGGTCGCCTTAGACATAGGAATTTGGTTCAGCTCA
TTGGATGGTGTCGACAAAAAGGTGACTTGTTACTTGTTTATGACTTTATGCCTAATGGAA
GCTTAGACAAGTATATCTATGATGAACCAAGAGTCATTTTAACGTGGGATCAGCGATTCA
AGATTATCAAAGGTGTGGCTTCGGGGCTGCTGTATTTACATGAAGAATGGGAACAAACT
GTAATCCATAGAGATATCAAAGCAGGGAATGTGTTATTAGATGCAGAAATGAATGGACGA
CTTGGAGATTTTGGACTTGCCAAGTTATATGAACATGGAGCAAATCCTAGCACGACAAGA
GTAGTGGGCACGTTGGGTTACTTGGCTCCTGAATTAACCAAGACCGGAAAGCCTACAAC
AAGCTCGGATGTTTTTGCCTTTGGTGCATTGCTGCTAGAAGTTACTTGTGGAAGGAGGC
CAATCGAGGCAAAAGCATTACCCGAGGAGTTAATTTTAGTGGATTGGGTTTGGGATAAAT
GGAAGGAAGGTGCCATTCTTGAAGTGGTTGATCCGAAACTAAATGGTGAGTACGATGAT
ATGGAGGCTGTGGTGGTGCTTAAATTAGGACTAATGTGTTCAAATAATACACCATCTAAG
AGACCTAGCATGAGGCTAGTGGTAAGATACTTGGAAGGGGAGATGGTGCTGCCGGAGA
CGCTTGCAGCGCCCGATGAATATGATGGGAAAAAAGGCGGCGCTAGTGGTATGGGATTT
GAGGACTTTGTACATTCATATCCATCCTCGTCTTATTTCGAGAAAGTAAGCACGTGGTCA
CCGGCTTATGATGGTGAAGGAGATATTGACATTGAAGCGGATCCGATAAAGCCGTTAAC
GGATTCTGGCAGAGAGGACAGCAGGTAG

>NbS00043874g0006.1 GenBank ID: KT225324
MYVGFSASTGLLASSHYILGWSFKLNGEAKFLDLDSLPSLPGVKKKHAGLIIVISNAAAVFALS
SILVAIYLFCRFKNADVIEPWELEIGPHRYSYQELKQATRGFKDSELLGRGGFGKVYKGILNSI
TQIAVKRISHESKQGWCRRRGDLLLVYDFMPNGSLDNFLFDQPKMVLTWEQRFKIIKGVASG
LLYLHEGYEQVVVHRDVKASNVLLDGELNGKLGDFGLARLYEHGSNPCTTRVVGTLGYLAP
ELPRTGRATTSSDVFAFGALLLEVVCGRRPIDPKAEPEEFVLVDLVWDKLREGKILEVVDYRL
KGEFNENEVLMLLKLGLMCSNNQPLARPSIRQVVRYLEGEILMPEAPTEQNADDRELEFNEN
ERWNSLGSANIIKSSSFTSLSNGDEDDNFASFSTSPLPLLYSGEAPRY

>NbS00043874g0006.1 GenBank ID: KT225324
ATGTATGTTGGCTTCTCTGCTTCTACTGGTTTGCTTGCCAGTTCACATTACATTTTAGGTT
GGAGTTTTAAGTTAAATGGAGAAGCTAAATTCTTAGACTTGGATTCTTTGCCATCACTTCC
TGGAGTCAAGAAGAAACACGCTGGCCTAATTATAGTCATTTCGAATGCTGCAGCTGTTTT
CGCGTTAAGCTCAATCTTGGTTGCTATTTATTTATTCTGCAGATTCAAGAATGCTGATGTG
ATTGAGCCATGGGAGCTTGAGATTGGTCCTCACAGATATTCATATCAAGAACTTAAGCAA
GCTACTAGAGGTTTTAAGGACAGTGAACTACTCGGACGTGGCGGATTTGGTAAAGTTTA
CAAGGGTATTTTAAATTCAATAACACAAATTGCTGTGAAGCGCATTTCGCATGAATCTAAA
CAAGGTTTGCAGGAATTTGTGTCTGAAATTGATTGGAAGACTCCGTCATAGGAATTTGGT
TCAATTGTTAGGGTGGTGTAGGCGTCGTGGTGACTTGTTGCTTGTGTATGATTTTATGCC
TAATGGAAGTTTGGATAATTTCTTGTTTGATCAGCCGAAAATGGTATTAACTTGGGAACAA
AGGTTCAAGATAATCAAAGGGGTTGCTTCTGGTTTATTGTACTTACATGAAGGCTATGAA
CAAGTTGTGGTACATCGCGATGTTAAGGCTAGTAATGTGCTACTAGATGGGGAATTGAAC
GGCAAGCTTGGGGATTTTGGACTTGCAAGATTATATGAGCACGGATCAAATCCCTGCAC
AACTAGAGTGGTAGGTACATTGGGGTACCTTGCACCAGAATTGCCAAGAACAGGACGGG
CCACAACAAGTTCCGATGTGTTTGCCTTTGGTGCTTTGTTACTCGAGGTAGTCTGTGGAC
GTAGGCCAATTGATCCCAAGGCAGAACCCGAGGAGTTTGTACTGGTGGACTTGGTGTGG
GATAAATTAAGAGAAGGGAAAATTCTTGAAGTGGTGGACTATAGATTGAAAGGTGAATTC
AATGAGAATGAGGTTTTGATGTTGTTAAAATTAGGACTAATGTGCTCAAATAACCAGCCAT
TGGCACGACCGAGCATAAGGCAAGTGGTAAGATACTTGGAAGGTGAAATTCTGATGCCT

GAGGCTCCAACAGAGCAAAATGCTGATGATCGAGAATTGGAGTTCAACGAAAATGAACG
TTGGAATTCGTTAGGATCAGCTAATATTATCAAGTCAAGTTCATTTACAAGTTTGTCTAAT
GGAGATGAAGATGATAATTTTGCTTCTTTTTCTACTTCACCACTTCCACTTCTATATAGTG
GTGAAGCACCTAGATACTGA

>NbS00000505g0005.1
MTQVDYWVMPSSLFHFKNSSSNETAFSFSTCFAFAIVPEYPKLRCHGLVFTVSHSKDFSTAL
PSQYLGLLNASDVGGHGLAFTVSHSKDFSTALPSQYLGLLNASDVGNFSNHIFAVEFDTVRD
FEFGDINDNHVGVNINSIQSNKSAAAAYYNDELVKQDLNLKSGKVILAWVEYDSVKKLINVTLS
PTSLKPKIPLFSYHLDLSPILKETTYVGFSASTGLLASSHYIFGWTFKLNGEVKFLDLDSLPSLP
GAKKKHTESSQLLQLFKNADVIEPWELEIGPHRYSYQELKQATKGFKDSELLGRGGFGKVYN
GILRNSKTQIAVKRRMNLKKACRDFVSEIVSIGRLRHRNLVQLLGWCRRRGDLLLVYDFMPN
GSLDSFLFDQPKMVLTWEQRFKIIKGVASALLYLHEGYEQVVVHRDVKASNVLLDGELNGKL
GDFGLARLYEHGSNPCTTRVMGTLGYLAPELPRSGRATTSSDVFAFGALLLEVVCGRRSIEP
KPLARPSIRQVIRYLEGEILMPEAPTEQNADDGELRFDENERWNSLRSTNIIKSSSFTSLANG
DEDDTFASFSTSPLPLLYSGEVRRY

>NbS00000505g0005.1 Corrected; GenBank ID: KT225325
MTQVDYWVMPSSLFHFKNSSSNETAFSFSTCFAFAIVPEYPKLRCHGLVFTVSHSKDFSTAL
PSQYLGLLNASDVGNFSNHIFAVEFDTVRDFEFGDINDNHVGVNINSIQSNKSAAAAYYNDEL
VKQDLNLKSGKVILAWVEYDSVKKLINVTLSPTSLKPKIPLFSYHLDLSPILKETTYVGFSASTG
LLASSHYIFGWTFKLNGEVKFLDLDSLPSLPGAKKKHTESSQLLQLFKNADVIEPWELEIGPH
RYSYQELKQATKGFKDSELLGRGGFGKVYNGILRNSKTQIAVKRRMNLKKACRDFVSEIVSI
GRLRHRNLVQLLGWCRRRGDLLLVYDFMPNGSLDSFLFDQPKMVLTWEQRFKIIKGVASAL
LYLHEGYEQVVVHRDVKASNVLLDGELNGKLGDFGLARLYEHGSNPCTTRVMGTLGYLAPE
LPRSGRATTSSDVFAFGALLLEVVCGRRSIEPKPLARPSIRQVIRYLEGEILMPEAPTEQNAD
DGELRFDENERWNSLRSTNIIKSSSFTSLANGDEDDTFASFSTSPLPLLYSGEVRRY

>NbS00000505g0005.1 Corrected; GenBank ID: KT225325
ATGACACAAGTAGACTATTGGGTCATGCCTTCTTCACTTTTCCACTTCAAGAACTCCAGTA
GTAATGAAACTGCCTTCTCTTTCTCAACATGTTTTGCTTTCGCCATCGTCCCCGAGTATCC
AAAACTCAGGTGCCATGGCTTAGTATTCACTGTTTCTCACTCAAAAGATTTCAGTACAGCT
CTACCAAGTCAGTATTTAGGTCTGCTTAATGCAAGTGATGTTGGTAATTTTTCTAACCACA
TTTTTGCTGTTGAATTCGACACGGTACGAGATTTTGAGTTCGGAGATATTAATGATAATCA
TGTTGGTGTCAACATCAACAGCATACAGTCCAATAAGTCTGCTGCAGCTGCTTACTACAA
TGATGAGTTAGTGAAACAAGATTTGAATCTCAAAAGTGGAAAGGTTATTCTAGCTTGGGT
AGAATATGATTCAGTCAAAAAAATTAATTAATGTTACTCTTTCACCAACTTCTTTAAAACCCA
AAATTCCTCTTTTTTCTTATCATTTAGACCTCTCTCCAATTCTTAAAGAAACTACATATGTT
GGTTTCTCTGCTTCTACTGGTTTGCTTGCAAGTTCACATTATATTTTCGGTTGGACTTTTA
AGTTAAATGGAGAAGTTAAATTTTTAGACTTGGATTCTTTGCCATCACTTCCTGGAGCCAA
GAAGAAACACACTGAATCATCTCAATTGCTGCAGCTGTTTTCGCGATAAGCTTAATCTTG
GTTGCTATTTATTTAATATGGAGATTCAAGAATGCTGATGTGATTGAGCCTTGGGAGCTT
GAGATTGGTCCTCATAGATATTCATACCAAGAACTTAAGCAAGCTACTAAAGGTTTTAAG
GACAGTGAACTACTCGGACGTGGCGGATTTGGTAAAGTTTACAATGGTATTTTAAGAAAT
TCAAAAACACAAATTGCAGTGAAGCGTCGCATGAATCTAAAAAAGGCTTGCAGGGATTTT
GTGTCTGAAATTGTCAGCATTGGAAGACTCCGTCATAGGAATTTGGTTCAACTGTTAGGG
TGGTGTAGGCGTCGAGGTGACTTGTTACTTGTGTATGATTTTATGCCTAATGGAAGCTTG
GATAGTTTCTTGTTTGATCAACCCAAAATGGTATTAACTTGGGAACAAAGGTTCAAGATAA
TCAAAGGGGTTGCTTCTGCTTTATTGTATTTACATGAAGGCTATGAACAAGTTGTGGTAC
ATCGAGACGTTAAGGCTAGTAATGTGCTACTAGATGGGGAACTGAACGGCAAGCTTGGG
GATTTTGGACTAGCAAGATTATATGAGCACGGATCAAATCCGTGCACGACTAGGGTGAT
GGGCACCTTGGGGTACCTTGCACCAGAATTGCCAAGATCAGGGCGGGCCACAACGAGT
TCTGATGTGTTTGCCTTCGGTGCTTTGTTACTCGAGGTGGTATGTGGACGTAGGTCAATT
GAACCCAAGGTAGAATGGAGTTTGTACTAGTGGATTTGGTACGGGACAAATTAAGAGAA
GGGAATGTTCATGATTACTCCTCACCCGGGGTTGGGTGAGGCTTGGTAGGGCTATCCGT
ACACGATTTGTGCATTGGTTTGTGTCTCCTTTAGTAGTTATTTATGATAGTTACTGTCTGA
TATATTTGCATGCTATTTTATTTATGGTTCTTTTGTTTAGATAATTATTTACGTTATTTCTGT
TGGAGTTACTAATGCTCTTTTGTTTTTGTTTTTGTTTTTGTTTTTGTTTTTGTTTTTCCTTTC
CTCCACTGAGCCGAGGGTCGATTGGAAACAGCCTCTCTGCCCTTTCGGGTAGGGGTAA

GGCCCGTCTACATATTACCCTCTCAAACCCCTGGGACTATACTGGATAGTTATTGTTGTT
GTTGTTGGAATGTTCATGATGTGGTGGACTATAGATTGAAAGGTGAGTTCAATGAAAATG
AGGTTTTGGTGGTGTTAAAATTAGGACTAATGTGCTCAAATAGCCAGCCATTGGCGCGAC
CGAGCATAAGGCAAGTGATAAGATACTTGGAAGGTGAAATTCTGATGCCTGAGGCTCCA
ACAGAGCAAAATGCTGATGATGGAGAATTGCGGTTCGACGAAAATGAACGTTGGAATTC
TTTAAGATCAACTAATATTATCAAGTCAAGTTCATTTACAAGTTTGGCTAATGGAGATGAA
GATGATACTTTTGCTTCTTTTTCTACTTCACCACTTCCACTTCTATATAGTGGTGAAGTAC
GTAGATACTGA

>NbS00056619g0001.1
MGHAFYSSPFQFKNSTTATAFSFSTCFALSIVPEYPKLGGHGLAFTISQSNDLSTALPSQYLG
LLNATDVGNFSNHIFAVEFDTVQDFEFRDINDNHVGVNINSLRSNMSVAAAYFDDDLVKQDL
NLKCGKVILAWVEYDSVKNLVNVTLSKSSKKPKLPLLSYHIDLSPFLKENMYVGFSASTGLLA
SSHYIFGWSFKLNGEAQFLDLDSLPLLPGPKKKHTGLILAISIISVIFTLISILVGIYLIKKFKNADVI
ESWELEVGPHRYSYQELKQATRGFKDSELLGYGGFGKVYKGVSQNSKMQIAVKRISHESKQ
GLREFVSEISSIGRLRHRNLVQLVGWCRRRGDLLLVYDFMPNGSLDNFLFDKPRMVLSWEQ
RFKIIKGVASGLLYLHEGYEQVVVHRDVKASNVLLDGELNAKLGDFGLARLYEHGSNPCTTR
VVGTLGYLAPELPRTGRATEKSDVFAFGALLLEVVCGRRPIESKAGPEELVLVDMVWNKMR
EGKILDVIDKRLKGEFNEREVVIVLKLGLMCSNNMREGKTLDVIDKRLKGEFNEREVVMVLKL
GLMCSNNEALARPSMRQVMSYLEGEIEMPDAPMAPGVYNGGFGFDENELHSLASSRGHTY
LANGDVDGTFVSISTAPMSCLFTDEVPR

>NbS00056619g0001.1 Corrected; GenBank ID: KT225326
MFQTPLEMVSLCLLLLCSLISSSIQLDGFTYTRFNQPNNNMTLSGVAKISQNGFIQLTNETSRL
MGHAFYSSPFQFKNSATGTAFSFSTCFALSIVPEYPKLGGHGLAFTISQSNDLSTALPSQYLG
LLNATDVGNFSNHIFAVEFDTVQDFEFGDINDNHVGVNINSLRSDISAAAAYFDDDLVKQDLN
LKCGKVILAWVEYDSVKNLVNVTLSRSSLKPKLPLLSYHVDLSPFLKGNMYVGFSASTGLLAS
SHYIFGWSFKLNGEAKFLDLDSLPLLPGPKKKHTGLILAISTIAVVFTLISILVGIYLIKKFKNADVI
ESWELEVGPHRYSYQELKQATRGFKDSELLGFGGFGKVYKGVLQNSKMEIAVKRISHESKQ
GLREFVSEISSIGRLRHRNLVQLVGWCRRRGDLLLVYDFMPNGSLDNFLFDKPRMVLSWEQ
RFKIIKGVASGLLYLHEGYEQVVVHRDVKASNVLLDGELNAKLGDFGLARLYEHGSNPCTTR
VVGTLGYLAPELPRTGRATEKSDVFAFGALLLEVVCGRRPIESKAGPEELVLVDMVWNKMR
EGKILDVIDKRLKGEFNEREVVIVLKLGLMCSNNEALARPSMRQVMSYLEGEIEMPDAPMAP
GVYNGGFGFDENELHSLASSRGHTYLANGDVDGTFVSISTAPMSCLFTDEVPR

>NbS00056619g0001.1 Corrected; GenBank ID: KT225326
ATGTTTCAAACCCCATTAGAAATGGTCAGTTTATGTCTTCTACTACTCTGCTCTCTCATTT
CCTCCTCTATACAGCTTGATGGCTTTACCTACACAAGATTCAATCAACCAAACAATAACAT
GACCTTAAGTGGAGTTGCAAAAATAAGCCAAAATGGATTTATTCAGTTAACCAATGAAAC
AAGTAGACTTATGGGTCATGCTTTCTATTCTTCACCTTTTCAGTTCAAGAACTCAGCTACA
GGCACTGCTTTTTCTTTTTCAACATGTTTTGCTCTTTCTATAGTACCTGAATATCCAAAACT
CGGTGGTCATGGACTTGCTTTTACCATTTCTCAATCCAATGATTTAAGCACAGCTCTACC
AAGTCAGTATTTAGGTTTACTCAATGCTACTGATGTTGGTAATTTCTCTAATCATATATTTG
CTGTTGAATTTGATACAGTACAAGATTTTGAGTTTGGAGATATTAATGATAATCATGTTGG
TGTTAACATTAATAGTTTAAGGTCTGATATCTCTGCTGCAGCTGCTTATTTTGATGATGAT
TTGGTAAAACAAGATCTGAATCTTAAATGTGGTAAGGTAATATTGGCATGGGTAGAATAT
GATTCAGTTAAAAACTTGGTTAATGTTACTCTTTCAAGATCTTCTTTAAAACCCAAGTTGC
CACTTTTATCTTATCATGTAGATCTCTCTCCATTTCTCAAAGGAAATATGTATGTTGGTTTT
TCTGCTTCAACTGGTTTGCTTGCTAGTTCTCATTATATTTTTGGTTGGAGTTTTAAGTTGA
ATGGTGAAGCCAAATTTCTAGACTTGGATTCACTTCCTTTATTGCCTGGTCCCAAGAAGA
AGCACACTGGCTTAATTTTAGCTATCTCAACTATAGCTGTCGTTTTTACATTGATTTCTATC
TTAGTTGGTATTTATTTGATCAAGAAATTCAAGAATGCTGATGTTATAGAGTCTTGGGAGC
TTGAGGTTGGTCCTCATAGATATTCTTATCAAGAACTTAAGCAAGCTACTAGAGGTTTTAA
GGATAGTGAGCTTCTTGGTTTTGGTGGATTTGGTAAAGTGTACAAGGGTGTTTTACAAAA
TTCAAAGATGGAAATCGCTGTGAAGCGTATTTCGCATGAATCTAAACAGGGTTTGCGCGA
GTTTGTGTCTGAAATTTCTAGCATTGGAAGACTTCGTCATAGGAATTTGGTTCAATTAGTA
GGATGGTGCAGACGTCGTGGTGATTTGTTACTTGTCTATGATTTTATGCCTAATGGGAGC
TTGGACAATTTCTTGTTTGATAAACCTAGAATGGTATTGTCTTGGGAGCAGAGGTTCAAG
ATCATCAAAGGGGTTGCTTCAGGTTTACTATACTTACATGAAGGTTATGAACAAGTTGTG
GTGCATCGAGACGTTAAGGCTAGTAATGTGTTGCTAGATGGTGAGTTAAATGCCAAGCTT

GGAGATTTTGGACTAGCAAGATTATATGAGCACGGATCAAACCCGTGCACGACTAGGGT
AGTAGGCACATTGGGGTACCTTGCACCAGAATTGCCAAGAACGGGAAGGGCTACAGAAA
AATCTGATGTCTTTGCCTTTGGTGCATTGTTGCTTGAGGTGGTGTGTGGGCGTAGGCCG
ATTGAGTCAAAGGCAGGGCCCGAGGAGTTAGTCTTAGTGGATATGGTATGGAACAAAAT
GAGAGAAGGGAAAATTCTTGATGTTATCGACAAGAGATTGAAAGGTGAGTTCAATGAACG
TGAAGTTGTGATTGTATTGAAATTGGGACTAATGTGTTCAAACAATGAAGCGTTGGCGCG
GCCTAGCATGAGACAAGTGATGAGTTATTTGGAAGGTGAAATTGAAATGCCAGATGCTC
CAATGGCTCCTGGTGTTTATAATGGAGGATTTGGATTCGACGAAAATGAACTGCATTCTT
TAGCATCTTCAAGAGGACACACATATTTGGCTAATGGAGATGTAGATGGTACATTTGTTT
CTATTTCTACTGCACCAATGTCATGTTTATTTACTGATGAAGTACCTAGGTAG

>NbS00027351g0001.1
MGHAFHSSPFQFKNSTTGTAFSFSTCFALAIVPEYPELVGHGLAFTISQSNDLSTTLPTLPSQ
YLGLLNATDVGNFSNHIFAVEFDTVQDFEFGNINDNIVGVKINSLRSNMSVAAAYFDDDLVKQ
DLKMS

>NbS00027351g0001.1 Corrected; GenBank ID: KT225327
MVSLCLLLLCSLISPSIQLDGFIHTRFNQPNNNMTLSGVAEINQNGFIQLTNETSRLMGHAFYS
SPFQFKNSTTATAFSFSTCFALSIVPEYPKLGGHGLAFTISQSNDLSTALPSQYLGLLNATDV
GNFSNHIFAVEFDTVQDFEFRDINDNHVGVNINSLRSNMSVAAAYFDDDLVKQDLNLKCGKVI
LAWVEYDSVKNLVNVTLSKSSKKPKLPLLSYHIDLSPFLKENMYVGFSASTGLLASSHYIFGW
SFKLNGEAQFLDLDSLPLLPGPKKKHTGLILAISIISVIFTLISILVGIYLIKKFKNSDVIESWELEV
GPHRYSYQELKQATRGFKDSELLGYGGFGKVYKGVSQNSKMQIAVKRISHESKQGLREFVS
EISSIGRLRHRNLVQLVGWCRRRGDLLLVYDFMPNGSLDNFLFDKPRMVLSWEQRFKIIKGV
ASGLLYLHEGYEQVVVHRDVKASNVLLDGELNAKLGDFGLARLYEHGSNPCTTRVVGTLGY
LAPELPRTGRATEKSDVFAFGALLLEVVCGRRPIESKAGPEELVLVDMVWNKMREGKILDVID
KRLKGEFNEREVVIVLKLGLMCSNNEALARPSMRQVMSYLEGEIEMPDAPMAPGVYNGGFG
FDENELHSLASSRGHTYLANGDVDGTFVSISTAPMSCLFTDEVPR

>NbS00027351g0001.1 Corrected; GenBank ID: KT225327
ATGGTCAGTTTATGTCTTCTACTACTCTGCTCTCTCATTTCCCCCTCTATACAGCTTGATG
GCTTCATCCATACAAGATTCAATCAACCAAACAACAACATGACCTTAAGTGGAGTTGCAG
AAATAAACCAAAATGGATTTATTCAATTAACCAATGAAACAAGTAGACTTATGGGTCATGC
TTTCTATTCTTCACCTTTTCAGTTCAAGAACTCAACTACAGCCACTGCTTTTTCTTTTTCAA
CATGTTTTGCTCTTTCTATAGTGCCTGAATATCCAAAACTTGGTGGTCATGGACTTGCTTT
TACTATTTCTCAATCAAATGATTTAAGCACAGCTCTTCCTAGTCAGTATTTAGGTTTACTCA
ATGCTACTGATGTTGGTAATTTCTCTAATCATATATTTGCTGTTGAATTTGATACAGTACAA
GATTTTGAGTTTAGAGATATTAATGATAATCATGTTGGTGTCAACATCAATAGTTTAAGGT
CTAATATGTCTGTTGCAGCTGCTTACTTTGATGATGATTTGGTAAAACAAGATCTGAATCT
TAAATGTGGGAAGGTAATATTGGCATGGGTAGAATATGATTCAGTTAAAAACTTGGTTAAT
GTTACTCTTTCAAAATCTTCTAAGAAACCCAAGTTGCCACTTTTATCTTATCATATAGATCT
CTCTCCATTTCTCAAAGAAAATATGTATGTTGGTTTCTCTGCTTCAACTGGTTTGCTTGCT
AGTTCTCATTATATTTTTGGTTGGAGTTTTAAGTTGAATGGTGAAGCCCAATTTCTAGACT
TGGATTCACTTCCATTACTACCTGGTCCCAAGAAGAAGCACACTGGCCTAATTTTAGCTA
TCTCAATCATATCTGTTATTTTTACTTTGATTTCTATCTTAGTTGGTATTTATTTGATCAAGA
AATTCAAGAATTCTGATGTTATAGAGTCTTGGGAGCTTGAGGTTGGTCCTCATAGATATT
CTTATCAAGAACTTAAGCAAGCTACTAGAGGTTTTAAGGATAGTGAGCTTCTTGGTTATG
GTGGATTTGGTAAAGTTTATAAGGGTGTTTCACAAAATTCGAAGATGCAAATCGCTGTGA
AGCGTATTTCGCATGAATCTAAACAGGGTTTGCGCGAATTTGTGTCTGAAATTTCTAGCA
TTGGAAGACTTCGTCATAGGAATTTGGTTCAATTGGTAGGATGGTGTAGACGTCGTGGT
GATTTGTTACTTGTCTATGATTTTATGCCTAATGGGAGTTTAGACAACTTCTTGTTTGATAA
ACCTAGAATGGTATTGTCTTGGGAGCAGAGGTTCAAGATCATCAAAGGGGTCGCTTCGG
GTTTGCTATACTTACATGAAGGTTATGAACAAGTTGTGGTGCATCGAGACGTTAAGGCTA
GTAACGTGTTGCTAGATGGTGAGTTAAATGCCAAGCTTGGAGATTTTGGACTAGCAAGAT
TATATGAGCACGGATCAAACCCGTGCACGACTAGGGTAGTAGGCACATTGGGGTACCTT
GCACCAGAATTGCCAAGAACGGGAAGGGCTACAGAAAAATCTGATGTCTTTGCCTTTGG
TGCATTGTTGCTTGAGGTGGTGTGTGGGCGTAGGCCGATTGAGTCAAAGGCAGGGCCC
GAGGAGTTAGTCTTAGTGGATATGGTATGGAACAAAATGAGAGAAGGGAAAATTCTTGAT
GTTATCGACAAGAGATTGAAAGGTGAGTTCAATGAACGTGAAGTTGTGATTGTATTGAAA
TTGGGACTAATGTGTTCAAACAATGAAGCGTTGGCGCGGCCTAGCATGAGACAAGTGAT

GAGTTATTTGGAAGGTGAAATTGAAATGCCAGATGCTCCAATGGCTCCTGGTGTTTATAA
TGGAGGATTTGGATTCGACGAAAATGAACTGCATTCTTTAGCATCTTCAAGAGGACACAC
ATATTTGGCTAATGGAGATGTAGATGGTACATTTGTTTCTATTTCTACTGCACCAATGTCA
TGTTTATTTACTGATGAAGTACCTAGGTAG

>NbS00029393g0011.1
MFQTPLEMVSLCLLLLCSLISSSIQLDGFTYTRFNQPNNNMTLSGVAKISQNGFIQLTNETSRL
MGHAFYSSPFQFKNSATGTAFSFSTCFALSIVPEYPKLGGHGLAFTISQSNDLSTALPSQYLG
LLNATDVGNFSNHIFAVEFDTVQDFEFGDINDNLQDFEFGDINDNHVGVNINSLRSDISAAAA
YFDDDLVKQDLNLKCGKVILAWVEYDSVKNLVNVTLSRSSLKPKLPLLSYHVDLSPFLKGNM
YVGFSASTGLLASSHYIFGWSFKLNGEAKFLDLDSLPLLPGPKKKHTGLILAISTIAVVFTLISIL
VGIYLIKKFKNADVIESWELEVGPHRYSYQELKQATRGFKDSELLGFGGFGKVYKGVLQNSK
MEIAVKRISHESKQGLREFVSEISSIGRLRHRNLVQLVGWCRRRGDLLLVYDFMPNGSLDNF
LFDKPRMVLSWEQRFKIIKGVASGLLYLHEGYEQVVVHRDVKASNVLLDGELNAKLGDFGLA
RLYEHGSNPCTTRVVGTLGYLAPELPRTGRATEKSDVFAFGALLLEVVCGRRPIESKAGPEE
LVLVDMVWNKMREGKTLDVIDKRLKGEFNEREVVMVLKLGLMCSNNEALARPSMRQVMSY
LEGEIEMPDAPMAPGVYNGGFGFDENELHSLASSRGHTYLANGDVD

>NbS00029393g0011.1 Corrected; GenBank ID: KT225328
MFQTPLEMVSLCLLLLCSLISSSIQLDGFTYTRFNQPNNNMTLSGVAKISQNGFIQLTNETSRL
MGHAFYSSPFQFKNSATGTAFSFSTCFALSIVPEYPKLGGHGLAFTISQSNDLSTALPSQYLG
LLNATDVGNFSNHIFAVEFDTVQDFEFGDINDNHVGVNINSLRSDISAAAAYFDDDLVKQDLN
LKCGKVILAWVEYDSVKNLVNVTLSRSSLKPKLPLLSYHVDLSPFLKGNMYVGFSASTGLLAS
SHYIFGWSFKLNGEAKFLDLDSLPLLPGPKKKHTGLILAISTIAVVFTLISILVGIYLIKKFKNADVI
ESWELEVGPHRYSYQELKQATRGFKDSELLGFGGFGKVYKGVLQNSKMEIAVKRISHESKQ
GLREFVSEISSIGRLRHRNLVQLVGWCRRRGDLLLVYDFMPNGSLDNFLFDKPRMVLSWEQ
RFKIIKGVASGLLYLHEGYEQVVVHRDVKASNVLLDGELNAKLGDFGLARLYEHGSNPCTTR
VVGTLGYLAPELPRTGRATEKSDVFAFGALLLEVVCGRRPIESKAGPEELVLVDMVWNKMR
EGKTLDVIDKRLKGEFNEREVVMVLKLGLMCSNNEALARPSMRQVMSYLEGEIEMPDAPMA
PGVYNGGFGFDENELHSLASSRGHTYLANGDVDGTFVSISTAPMSCLFTDEVPR

>NbS00029393g0011.1 Corrected; GenBank ID: KT225328
ATGTTTCAAACCCCATTAGAAATGGTCAGTTTATGTCTTCTACTACTCTGCTCTCTCATTT
CCTCCTCTATACAGCTTGATGGCTTTACCTACACAAGATTCAATCAACCAAACAATAACAT
GACCTTAAGTGGAGTTGCAAAAATAAGCCAAAATGGATTTATTCAGTTAACCAATGAAAC
AAGTAGACTTATGGGTCATGCTTTCTATTCTTCACCTTTTCAGTTCAAGAACTCAGCTACA
GGCACTGCTTTTTCTTTTTCAACATGTTTTGCTCTTTCTATAGTACCTGAATATCCAAAACT
CGGTGGTCATGGACTTGCTTTTACCATTTCTCAATCCAATGATTTAAGCACAGCTCTACC
AAGTCAGTATTTAGGTTTACTCAATGCTACTGATGTTGGTAATTTCTCTAATCATATATTTG
CTGTTGAATTTGATACAGTACAAGATTTTGAGTTTGGAGATATTAATGATAATCATGTTGG
TGTTAACATTAATAGTTTAAGGTCTGATATCTCTGCTGCAGCTGCTTATTTTGATGATGAT
TTGGTAAAACAAGATCTGAATCTTAAATGTGGTAAGGTAATATTGGCATGGGTAGAATAT
GATTCAGTTAAAAACTTGGTTAATGTTACTCTTTCAAGATCTTCTTTAAAACCCAAGTTGC
CACTTTTATCTTATCATGTAGATCTCTCTCCATTTCTCAAAGGAAATATGTATGTTGGTTTT
TCTGCTTCAACTGGTTTGCTTGCTAGTTCTCATTATATTTTTGGTTGGAGTTTTAAGTTGA
ATGGTGAAGCCAAATTTCTAGACTTGGATTCACTTCCTTTATTGCCTGGTCCCAAGAAGA
AGCACACTGGCTTAATTTTAGCTATCTCAACTATAGCTGTCGTTTTTACATTGATTTCTATC
TTAGTTGGTATTTATTTGATCAAGAAATTCAAGAATGCTGATGTTATAGAGTCTTGGGAGC
TTGAGGTTGGTCCTCATAGATATTCTTATCAAGAACTTAAGCAAGCTACTAGAGGTTTTAA
GGATAGTGAGCTTCTTGGTTTTGGTGGATTTGGTAAAGTGTACAAGGGTGTTTTACAAAA
TTCAAAGATGGAAATCGCTGTGAAGCGTATTTCGCATGAATCTAAACAGGGTTTGCGCGA
GTTTGTGTCTGAAATTTCTAGCATTGGAAGACTTCGTCATAGGAATTTGGTTCAATTAGTA
GGATGGTGCAGACGTCGTGGTGATTTGTTACTTGTCTATGATTTTATGCCTAATGGGAGC
TTGGACAATTTCTTGTTTGATAAACCTAGAATGGTATTGTCTTGGGAGCAGAGGTTCAAG
ATCATCAAAGGGGTTGCTTCAGGTTTACTATACTTACATGAAGGTTATGAACAAGTTGTG
GTGCATCGAGACGTTAAGGCTAGTAATGTGTTGCTAGATGGTGAGTTAAATGCCAAGCTT
GGAGATTTTGGACTAGCAAGATTATATGAGCACGGATCAAACCCGTGCACCACAAGGGT
AGTAGGCACATTGGGTTACCTTGCACCAGAATTGCCAAGAACAGGACGGGCTACAGAAA
AGTCTGATGTCTTTGCCTTTGGGGCATTGTTGCTTGAGGTGGTTTGTGGGCGTAGGCCG
ATTGAATCAAAGGCAGGGCCCGAGGAGTTAGTCTTAGTTGATATGGTATGGAACAAAAT

GCGAGAAGGGAAAACTCTTGATGTTATAGACAAGAGATTGAAAGGCGAGTTCAACGAAC
GTGAGGTTGTGATGGTATTGAAATTGGGGCTGATGTGTTCAAACAACGAAGCGTTGGCG
CGGCCTAGCATGAGACAAGTGATGAGTTATTTGGAAGGTGAAATTGAAATGCCAGATGC
TCCAATGGCTCCTGGTGTTTATAATGGAGGATTTGGATTCGACGAAAATGAACTGCATTC
TTTAGCATCTTCAAGAGGACACACATATTTGGCTAATGGAGATGTAGATGGTACATTTGTT
TCTATTTCTACTGCACCAATGTCATGTTTATTTACTGATGAAGTACCTAGGTAG

>NbS00003611g0313.1
MQRNTKLKIRKWKRLGSYILIQVKTVDCLNFSYANFPKQNENDFITTNYSFIGLNAAMQVTGD
ARGTTITNLSGRIWYHQPFKLWNRKKNITASFNSTFVINIAPQSNPWGEGLAFILTKESGLNSI
PDNSDGQWLGIVNASTNGSLSNNIFAVEFDTRKSYFEDLDDNHVGIDINSIYSINQASLTDRG
VNLSRAIDVIASVQYDGESKILEVYAFMSNQTGGFNARIPIIVMPLDLSYLSEDVFVGFSASTGI
YTQLNCIKAWNFTSIQIGNDNDGSLLWLWILIPIISGLVMFLGGVFIYFTWRRKKKRMQVIDQN
EKIEILIRSSATAPQRFQLKDLKRGTGNFDPRNILGRGGCGVVFKGLLADKDVAVKRFFKDSS
QGAKDLIAEVTTIGNLHHKNLVKLFGWCYESNELLVVYEFMPNGSLDKLIFCEEKGEIREGLS
LSWEKRHVIVCGIAQALDYLHNGCAKRVLHRDIKASNIMLDSELNARLGDFGLARTVQVSGM
THHSTKEIAGTLGYMAPESFLIGRATVETDVFAFGVLILEVVCGRKPGNQNVENIYSNSIIEYV
WDLYKSEKITDAIDVQLDREFDEGQAECVLMLGLACCHPNPYERPSMKTALQILTGESVLPTI
PTEKPAFVWPARAPSSNDAAGDSFMEGQLTPITVLSGR

>NbS00003611g0313.1 Corrected; GenBank ID: KT225329
MFYLGKISYLTSLLYLIFFIFTLFLEVFLVIRVDCLNFSYANFPKQNENDFITTNYSFIGLNAAMQ
VTGDARGTTITNLSGRIWYHQPFKLWNRKKNITASFNSTFVINIAPQSNPWGEGLAFILTKES
GLNSIPDNSDGQWLGIVNASTNGSLSNNIFAVEFDTRKSYFEDLDDNHVGIDINSIYSINQASL
TDRGVNLSRAIDVIASVQYDGESKILEVYAFMSNQTGGFNARIPIIVMPLDLSYLSEDVFVGFS
ASTGIYTQLNCIKAWNFTSIQIGNDNDGSLLWLWILIPIISGLVMFLGGVFIYFTWRRKKKRMQ
VIDQNEKIEILIRSSATAPQRFQLKDLKRGTGNFDPRNILGRGGCGVVFKGLLADKDVAVKRF
FKDSSQGAKDLIAEVTTIGNLHHKNLVKLFGWCYESNELLVVYEFMPNGSLDKLIFCEEKGEI
REGLSLSWEKRHVIVCGIAQALDYLHNGCAKRVLHRDIKASNIMLDSELNARLGDFGLARTV
QVSGMTHHSTKEIAGTLGYMAPESFLIGRATVETDVFAFGVLILEVVCGRKPGNQNVENIYSN
SIIEYVWDLYKSEKITDAIDVQLDREFDEGQAECVLMLGLACCHPNPYERPSMKTALQILTGE
SVLPTIPTEKPAFVWPARAPSSNDAAGDSFMEGQLTPITVLSGR

>NbS00003611g0313.1 Corrected; GenBank ID: KT225329
ATGTTTTATCTAGGCAAGATTAGTTATTTAACCTCCCTTCTTTATCTAATTTTCTTCATATTC
ACACTTTTCTTGGAAGTTTTTTTGGTTATTAGGGTGGATTGCCTGAATTTCAGCTACGCAA
ATTTCCCCAAGCAAAACGAAAATGATTTCATCACTACTAATTATTCTTTCATAGGATTGAAT
GCTGCTATGCAAGTAACAGGCGATGCTCGTGGAACTACTATCACCAACTTATCTGGAAG
AATATGGTATCATCAGCCTTTCAAGTTATGGAATCGAAAGAAGAACATCACAGCATCGTT
TAATTCCACGTTTGTGATTAATATCGCTCCTCAGAGTAATCCATGGGGCGAAGGATTAGC
TTTCATATTGACAAAGGAAAGTGGTCTTAATTCTATCCCAGATAATAGTGATGGACAATGG
CTTGGAATTGTGAATGCAAGTACAAATGGATCTTTATCAAACAACATCTTTGCAGTTGAAT
TTGACACAAGGAAAAGTTACTTTGAAGATCTTGATGATAATCATGTTGGCATTGACATAAA
CAGCATCTACTCTATCAACCAAGCTTCTTTAACTGACCGTGGTGTTAATCTTTCACGAGCT
ATTGATGTTATAGCAAGTGTTCAGTATGATGGAGAATCAAAGATCTTGGAGGTTTATGCA
TTCATGAGTAATCAGACTGGGGGCTTTAACGCGAGAATTCCCATCATTGTCATGCCTCTT
GATCTTTCCTATCTTTCCGAGGATGTTTTCGTGGGATTTTCAGCTTCAACTGGGATCTATA
CTCAGCTGAACTGCATAAAAGCATGGAATTTTACAAGCATACAAATTGGAAATGATAATG
ATGGTAGCCTATTATGGTTGTGGATCTTGATACCAATCATATCGGGTCTGGTTATGTTTCT
TGGCGGGGTTTTTATATATTTCACCTGGAGGAGGAAAAAGAAGAGGATGCAAGTGATAG
ATCAAAATGAGAAAATAGAGATACTGATTCGGAGTTCAGCTACTGCACCACAGAGATTCC
AGCTTAAGGACTTGAAACGAGGGACCGGGAACTTTGATCCCAGGAACATTCTTGGAAGA
GGAGGATGTGGAGTAGTGTTCAAAGGGTTGTTAGCTGATAAAGATGTAGCTGTGAAGAG
ATTCTTTAAAGATTCAAGTCAAGGGGCAAAAGATCTCATAGCAGAAGTCACAACCATTGG
CAATCTCCACCACAAAAACCTTGTCAAACTGTTTGGATGGTGCTATGAAAGCAATGAACT
ACTTGTGGTGTATGAGTTCATGCCAAATGGGAGCTTAGACAAGTTGATATTTTGTGAAGA
AAAAGGTGAAATCAGAGAAGGGTTGAGCCTGAGTTGGGAAAAAAGGCATGTCATTGTCT
GTGGCATAGCTCAAGCACTGGATTATCTGCATAATGGGTGCGCGAAAAGAGTACTGCAT
AGGGACATAAAAGCCAGCAACATCATGCTTGACTCAGAACTCAATGCTCGGTTGGGAGA
TTTTGGATTGGCTAGGACAGTTCAAGTGAGTGGAATGACTCACCATTCGACGAAAGAGAT
AGCTGGAACTCTTGGCTATATGGCTCCAGAAAGTTTCCTTATAGGCCGAGCTACAGTTGA
GACAGATGTGTTTGCATTTGGAGTGCTCATTCTTGAAGTTGTCTGTGGTCGAAAACCTGG
AAACCAAAATGTGGAGAATATCTACAGCAACAGTATCATTGAATATGTGTGGGATCTCTA
TAAGAGTGAAAAGATCACTGATGCCATAGACGTTCAATTGGATAGAGAATTTGATGAAGG
GCAAGCTGAATGTGTTTTAATGTTAGGATTGGCTTGCTGCCATCCGAATCCATATGAAAG
GCCAAGCATGAAAACTGCTTTACAGATTCTAACAGGGGAATCAGTTTTACCAACTATTCC
TACTGAGAAACCTGCATTTGTGTGGCCAGCTAGAGCTCCATCGTCGAATGACGCTGCAG
GTGACTCTTTCATGGAAGGCCAACTTACACCAATTACAGTTCTCAGTGGCAGATGA

>NbS00005288g0011.1
MRVDCLNFSYANFPKQNKHDFITTNYSFIGLNAAMQVTGDARGTTITNLPGRIWYRQPFKLW
NRKKNITASFNSNPWGEGLAFILTEKSGLHSIPDKSNGQWLGIVNASTNGSLSNNIFAVEFDT
RKSYFEDLDDNHVGIDINSIYSINQASLTNRGVNLSRAIDVIARVQYDGDSKILNVYAFMSNQI
GGFNASNPIISMPLDLSYLPEDVFVGFSASTGIYTQLNCIKAWNFTSTEIGNDNDDTNNICSGY
VSWWGFYIFHLEEEKEEDATTAPQRFQLKDLKRGFDPRNILGRGGCGVVFKGLLADKEVAV
KRFFRDSSQGAKDLIAEVTTIGNLHHKNLVKLLGWCYESKELLVVYEFKPNGSLDKLIFCEEK
GEIRGVEPELGKKTVLHRDIKASNIMLDSELNARLGDFGLARTVQVSGMTHHSKKEIAGTLGY
MAPESFLIGRATVETDVFAFGVLILEVACGQKPGNQNEENIYSNRIIEYVWDLYKSERIIGAIDV
RLDRDFDEGQAECV

>NbS00005288g0011.1 Corrected; GenBank ID: KT225330
MFYLIFFIFTLFLEGFIVMRVDCLNFSYANFPKQNKHDFITTNYSFIGLNAAMQVTGDARGTTIT
NLPGRIWYRQPFKLWNRKKNITASFNSNPWGEGLAFILTEKSGLHSIPDKSNGQWLGIVNAS
TNGSLSNNIFAVEFDTRKSYFEDLDDNHVGIDINSIYSINQASLTNRGVNLSRAIDVIARVQYD
GDSKILNVYAFMSNQIGGFNASNPIISMPLDLSYLPEDVFVGFSASTGIYTQLNCIKAWNFTST
EIGNDNDDTNNICSGYVSWWGFYIFHLEEEKEEDATTAPQRFQLKDLKRGSGNFDPRNILGR
GGCGVVFKGLLADKEVAVKRFFRDSSQGAKDLIAEVTTIGNLHHKNLVKLLGWCYESKELLV
VYEFKPNGSLDKLIFCEEKGEIRGVEPELGKKTVLHRDIKASNIMLDSELNARLGDFGLARTV
QVSGMTHHSKKEIAGTLGYMAPESFLIGRATVETDVFAFGVLILEVACGQKPGNQNEENIYS
NRIIEYVWDLYKSERIIGAIDVRLDRDFDEGQAECV

>NbS00005288g0011.1 Corrected; GenBank ID: KT225330
ATGTTTTATCTAATTTTCTTCATATTCACACTTTTCTTAGAAGGTTTTATTGTTATGAGGGT
GGATTGCCTGAATTTCAGCTACGCAAATTTCCCCAAGCAAAATAAACATGATTTCATCACT
ACTAATTATTCCTTCATAGGATTGAACGCTGCTATGCAAGTAACAGGCGATGCTCGTGGA
ACTACTATCACAAACTTACCTGGAAGAATATGGTATCGTCAGCCTTTCAAGTTATGGAATC
GAAAGAAGAACATCACAGCATCGTTTAACGTTTGTGATTAATATCACTCCTCAGAGTAATC
CGTGGGGAGAAGGATTAGCTTTCATATTGACAGAGAAAAGTGGTCTTCATTCCATCCCAG
ATAAAAGTAATGGACAATGGCTTGGAATTGTGAATGCAAGTACAAATGGATCTTTATCAAA
CAACATTTTTGCAGTTGAATTTGACACAAGGAAAAGTTACTTTGAAGATCTTGATGATAAT
CATGTTGGCATTGACATAAACAGCATCTATTCTATCAATCAAGCTTCTTTAACTAACCGTG
GTGTTAATCTTTCACGAGCTATTGATGTTATAGCAAGAGTTCAGTATGATGGAGACTCAA
AGATCTTGAATGTTTATGCATTCATGAGTAATCAGATCGGGGGCTTTAACGCGAGTAATC
CCATCATTTCCATGCCTCTTGATCTTTCCTATCTTCCCGAGGATGTTTTCGTGGGATTTTC
AGCTTCAACTGGGATATATACTCAGCTGAACTGCATAAAGGCATGGAATTTTACAAGCAC
AGAAATTGGTAATGATAATGATGATACCAATAATATCTGTTCTGGTTATGTTTCTTGGTGG
GGTTTCTATATCTTTCACCTGGAGGAGGAAAAAGAAGAGGATGCAAGTGATAGATCAAAA
TGAGAAAATAGAGATACTGATTCGGAGTTCAGCTACTGCACCACAGAGATTCCAGCTAAA
GGACTTGAAACGAGGGAGCGGGAACTTTGATCCCAGAAACATTCTTGGAAGAGGAGGG
TGTGGAGTAGTGTTCAAAGGGTTGTTAGCTGATAAAGAGGTAGCTGTGAAGAGATTCTTT
AGAGACTCAAGTCAAGGGGCAAAAGATCTCATAGCAGAAGTCACGACCATTGGCAATCT
CCACCACAAAAACCTTGTCAAATTGTTAGGATGGTGCTATGAAAGCAAAGAACTCCTTGT
GGTGTATGAGTTCAAGCCAAATGGAAGCTTAGACAAGTTGATATTTTGTGAAGAAAAAGG
CGAAATCAGAGGGGTTGAGCCTGAGTTGGGAAAAAAGACGTCATTGTCTGTGGCATAGC
TCAAGCACTGGATTATCTGCATAATGGGTGCGCGAAAAGAGTACTTCATAGGGACATAAA
AGCCAGCAATATCATGCTTGACTCAGAACTCAATGCTCGGTTGGGAGATTTTGGATTGGC
TAGGACAGTTCAAGTGAGTGGAATGACTCACCATTCGAAGAAAGAGATAGCTGGAACTC
TAGGCTATATGGCTCCAGAGAGTTTCCTTATAGGCCGAGCCACTGTTGAGACAGATGTG
TTTGCATTTGGAGTGCTCATTCTTGAAGTTGCCTGTGGTCAAAAACCTGGAAACCAAAAT
GAGGAGAACATCTACAGCAACAGAATCATTGAATATGTGTGGGATCTCTATAAGAGTGAA
AGGATCATTGGTGCCATAGATGTTCGATTGGATAGAGATTTTGATGAAGGGCAAGCTGAA
TGTGTGTAA

>NbS00007832g0008.1 GenBank ID: KT225331
MRLLTPKLNTIFIFFCCLHSISQAKLKKFEKQYGNFDHTYIPFLEIKEPAQISNQALQVTPDTAN
SHYNMFNNSGRILLKQSFKLWEGDTSSKDSRVASFNSSFLVNIYRLDNKTAAEGLTFLITPDL
DLPPNSQGQYLGLTNATTDGKFINRILAVELDTFQQDFDIDDNHIGIDIHSIKSIRSESLAKHGIE
LAPIGARFYNIWVQYDGIKKVLDVYIAEQDEKNGATPPRPKDPILSHDLDLRDFVNQESYFGF
SASTGNFNQLNCVLRWNFTVEYFQEKNQSLTLGLGVGVPLFVLFLILVVYLGYYCYKKRVAR
SESNIVGALKSLPGMPRDFEFKVLKNATDNFDEKNKLGEGGFGVVYKGYLVGENLEIAVKWF
SRESMKGQDDFLAELTIINRLRHKHLVKLLGWSHKHGKLILVFEYMPNGSLDKHLFSGPDKK
PLSWQVRYKIVSGVASALHYLHDEFEQKVVHRDLKASNIMLDTNFNARLGDFGLARALDHEK
TSYAESEGVLGTMGYIAPECFHTGKATQHSDVYAFGAVLLELVCGQRPGTKVNGFQLFADW
VWYLHRDGRILETVDTSLGDDYVAEEAKRLLLLALACSHPIASERPKTQTIVQIISGSVPAPEV
PPFKPAFVWPSMVPMETDIDSSLVDTISITTPQFSSGNNSVEYRSK

>NbS00007832g0008.1 GenBank ID: KT225331
ATGAGATTATTGACACCAAAACTCAACACCATCTTCATCTTTTTCTGTTGTCTTCATTCCAT
ATCTCAAGCCAAGCTGAAAAAATTCGAAAAACAATATGGAAATTTTGACCATACATATATT
CCATTCTTGGAAATTAAAGAACCAGCACAAATCAGCAACCAAGCTTTACAAGTAACTCCT

GATACAGCCAACTCTCATTACAACATGTTTAATAATTCAGGTAGAATTCTCTTGAAACAAT
CCTTCAAATTATGGGAAGGTGACACGTCATCAAAAGATAGTAGAGTTGCATCTTTCAACT
CTTCATTTCTTGTGAATATTTACAGGCTAGATAATAAAACTGCAGCTGAAGGTTTAACTTT
CTTGATTACTCCTGATTTGGATTTGCCACCAAATAGCCAAGGGCAGTATTTAGGGTTGAC
AAATGCTACTACTGATGGGAAATTTATTAACAGAATTCTCGCGGTTGAGCTCGATACTTTT
CAGCAAGATTTTGACATTGATGATAACCATATTGGCATTGATATTCATAGTATTAAATCTAT
AAGGTCTGAATCTTTAGCCAAACATGGGATTGAGCTTGCTCCAATTGGTGCAAGATTTTA
CAATATTTGGGTACAATATGATGGTATCAAGAAAGTTCTTGATGTGTACATAGCAGAACAA
GATGAGAAAAATGGCGCAACCCCACCTAGGCCAAAGGATCCAATATTATCACATGATCTT
GATTTAAGGGATTTTGTGAATCAAGAATCATACTTTGGATTTTCTGCTTCAACAGGGAATT
TTAATCAATTGAATTGTGTGTTAAGGTGGAATTTTACAGTTGAGTATTTTCAAGAAAAAAAT
CAATCTTTAACTCTTGGTTTAGGTGTTGGGGTTCCATTATTTGTTCTATTCTTGATTTTGGT
AGTATATTTAGGGTATTATTGCTACAAAAAGAGGGTTGCTAGGTCAGAGTCAAATATAGTT
GGTGCACTTAAGAGTTTGCCTGGAATGCCTAGAGATTTTGAGTTTAAGGTATTGAAGAAC
GCTACTGATAATTTTGATGAAAAGAATAAGTTGGGTGAAGGGGGATTTGGAGTTGTATAC
AAAGGGTATTTAGTTGGTGAAAATTTGGAAATTGCAGTGAAATGGTTTTCAAGGGAAAGT
ATGAAAGGTCAGGATGATTTCTTGGCTGAACTTACCATTATCAACCGTCTCAGGCATAAA
CATCTTGTCAAATTGCTTGGTAAGATTTTTTTTTTACTGTACTTTTACTTTTTGTATTAAGG
AAAATTTGTTATATATAAGAGTTGTTGCTGTGTGACCAGAAAATCTCGAGTTGAAGCATGA
GTTGGAATCTTTTAACAAGGTGCATGAATTTCATCTGTTCTTAAAATAAGTCTTTACTACTA
TGGAGAAAAATTTAAGAGCTTTCATGGCAGCGTCAAAAATGGTTTGCGCATAGATTTGAC
AACAGTTTGAAAGTGAACAATATTTTTTTCAACGAAGTAGGAGTTTGGACATGATTTTTGA
AAAAAAGTAATATTTGAAACTAAGTTGAAAAACGGTGAAATTTGAAATTATATTTGGACAT
GCATTTTACTTGAAAAAAAAGTTGTATATTTTTTAGCGGAGAAAATAAAATTCTTAAAATTT
GAAAAAGGGGTTTTTTTGCAAATTTTTCATAAACTTGCAAAAATTTACAGACAAATACATTT
TTGAAAAAAAAATTAACAATAAAAAAAAGTTATAGACAAACGGGACCTAAATCTTGCTTCT
AATATTTCCTGAGGGTTTGGTACCAGAGATAATTAAGGGATAATTAATTTCAAGATTAAAT
TTAAGATAATTTATCTTATGTTTGGTTGGAACAAAATCACTGTATAAATAATTTCGAAATTA
GTTATCTCAGAATTATAATATTATTCTTATTCCTATAGAAGGGCGGGATAACTGAACTCGA
AACAATTAATTTTGAGATAATTTATTTCTCAACTAAACGACCCATATATGATGTTGCACTG
GTTGAACGGTTCTTCTTGCCGTTTTTAAATTTTGGGTCCGCCTATAACGCGTGGGGATAA
CTTTGAATGTTAAAAGTTGGTCAATAGTGGATAGGATTTAGGAGTAATTGAGAGAACAATT
ATAAAAGCAAAAATATTGAATTATCTTTCTTTCGAGATGAAATAAAGAATCCTATGGAAAA
AGACTTCATGGATGTGATCGGAGAAAATGGTCCAAATCTTTAAAACGAATAAAATATATGT
AAAGCCAAACCTTTGTAGGGTATGATCACTCGAATGCAAGAACCACTTTTGCTAGCCTAA
ATAATAACAATAATGCCCTCTTGGAGCAAAAATCCAAAATTTAATAGTTAAGTTCTTGGCT
GGAAGACAGAAGAGCACTGATTCATTCCCTTTAGTATCTATTTTTGCTTTCTCTACCAACA
CTTTCAGACATAGTCCCACAGAATTTAAATTTTATGAATTTTAAATTTACAACTAAACTTAG
AGTTCATTATTTAGTTGTTAGGTTTGCAATTAAATATTTAATAATTTTTTTAGTACAAATATA
AAATTTAAGCAAAAAGTTGCTGGGTTCGTCTGAACCCGTAGATAACACCCTCTCTCCGCC
ACTGCTTGTAGTTCTCAAGTTTGAGCTTTGAAAATGGAGTAATCCGTTTTCATAAATTAGT
CCTACACGGCACGAATTTGAATTAATTAGATCAGTAGATTCCGGATATTATTTGATTCTTA
TGTACGATCTTTTGTTCTTGTGCTCAAGCTTAAAATGTGCCCTAGATTAGTCTTTAAACAG
TGAAATCAGCAATGAGATCATTAGTGTCTTGAATCATGGTTTTGAACAGGAATTTTCTTTT
TTGTGGAATCCTTCAATACTAGCTTGTCCTCATTCTTATCTTTAAGTCTTCTCGATTTTATC
TTGTGTTTATAAGGACTTTATTTAAAGTGTGTACATATAAGCAATGAGGTTCAAACTATAA
AATTCAAATCCAAAATTTGCTTCTGACCATAGTCAAAGCAATTTTCTTGTGCTATACTTTCT
CAGTAAGAATCAAGGAAGCTTCATTGGAAAATCAACCTTCATTCAAATGTCAATTATTGTG
ACAAATTTGGTATTATCTAAAAGTTAATATCAACATCATAGCCACTGAATAGAACAATTTG
CTTCAAAATGCAATCAACTCCAATTACTGCTAAGAGGTGACCCTACAACAATACATCATAA
TTGAGACCTGTTAAAACTAGGTCCTACAAAATAGATGATTGGAGCACTGCTTACCCAACT
TTTTTTTTGCCATGTAAAAAGTTTGATATCCTTAGCATTTTATAGATCATTATTGTTGATTG
GATAAATTGATATACCATTATTATTGTATCAAGGAATTAACCTATATACACTAACCGTATTA
AGAATTTTACATTATCAATGTGCTTTGATCTATTGTAGCAGATAAATTTACTTCAAAAGTGA
AACTGTTGTATCAGTTTGAAGCTAACCAGGATTTTCTTTAAGGGGGTTCAATTAAAAATTT
AAAAGAGATTGTAGTTAGTGAGAATTGAACCAGTGACCTCACAAAAGTTTTAAACTTCCTT
CACCACTAAACTATGCTTTACGGTTATGATAAGTTGATTCAGAATATAACATATACAGATT
AAACAGAACTTGCCTTGTATGTACAATATAATTTTTCGGCGAAGAGGTTTCGAGTGAACT
CCTTCGGCCCCCCTAAATCCGCCCATGGCTGCAGCTATTATCATAGTATAAAAAATGAAG
GAGAACCTCGGCGTAACAAGTAAAGTTGTTATCATGTGACCAGGAGGTCACGGGTTCGA

TTACGGGGTAAGGCTACGTACGATAGATCATTATGGTCCAGCCCTTCCTTGGACCCTGT
GCATAGCGGGAGCTTAGTGCACCGGACTGCCCTTTTTTGTCAGAAACATTTAACTATTGG
CCTTTCTAGCAGACTTATTGACTTTCAAGCAAATTAGTGTGCAACTACAAGCTGATGAGA
ACAGCAATACAGACATAAGCATCTTACAATCATATATTATTGTGACAGGATGGAGCCACA
AGCATGGAAAGCTAATACTTGTATTTGAATACATGCCAAATGGTAGCCTAGACAAACACC
TTTTCTCAGGTCCAGATAAAAAGCCACTCAGCTGGCAAGTCAGGTACAAGATTGTATCGG
GCGTCGCCTCAGCCCTGCACTATCTCCACGATGAGTTTGAGCAGAAAGTGGTCCATCGT
GATCTTAAGGCGAGCAACATCATGCTCGACACCAACTTCAATGCACGCCTCGGTGATTTT
GGCCTGGCACGAGCACTTGACCACGAGAAGACCTCGTATGCTGAGTCCGAGGGAGTGC
TAGGCACGATGGGGTACATTGCACCAGAGTGTTTTCACACTGGAAAAGCCACTCAACAT
TCTGATGTCTATGCATTTGGGGCAGTATTGTTGGAATTAGTATGCGGCCAAAGGCCTGG
AACGAAAGTTAATGGCTTTCAACTATTCGCCGATTGGGTTTGGTACTTGCATCGCGACGG
TAGAATCCTCGAAACTGTTGACACGAGTCTCGGGGATGATTATGTAGCTGAAGAAGCAA
AGAGATTGTTACTTCTTGCTTTGGCATGCTCTCATCCAATTGCTAGTGAAAGGCCTAAAA
CACAGACGATAGTTCAAATTATATCAGGGTCAGTACCAGCACCAGAAGTTCCACCATTTA
AACCAGCATTTGTGTGGCCTTCTATGGTGCCAATGGAAACTGATATAGACTCGAGTCTTG
TCGATACGATATCCATTACAACTCCTCAGTTCAGTTCAGGAAACAACAGCGTTGAGTATC
GAAGCAAGTAG

>NbS00001007g0015.1 GenBank ID: KT225332
MGLLTAKLNTIFIFLCCLQSISQAKLKKFDKQYGPFDETYYDIFQVVPPATISNQALQVTSDYA
KNLMILFNNAGRILFEQSFKLWDDSSKRVASFNSSFLVNIYRVKNETATEGLTFLISPDLELPT
NSLTNGNTDGRSANKVIAVELDTFKQEFDPDDNHIGIDIHSVISVKVESLTPHGIELAGIGARFY
NIWVQYDGIKKVLDVYIVEQTEVNGSTPPRPENPILTHDVDLRDVVNQESYFGFSASTGNFS
QLNCVLRWNLTVLGFICSILDFVTTSGYYYHKKKAARSESITLGALKSLPGMPRDFEFKVLKK
ATNNFDEKNKLGEGGFGVVYKGYLVGENLEIAVKWFSRESIKGQDDFLAELTLSTVFGINIFK
LLGWSHKHGKLLLIYKYMPNGSLDKHLFSGPDKKPLSWQIRYKIISGKVIHRDLKANNIMLDSK
SSKYRPRKE

>NbS00001007g0015.1 GenBank ID: KT225332
ATGGGATTGTTGACAGCAAAACTCAACACCATCTTCATCTTTCTCTGCTGTCTTCAGTCCA
TATCTCAAGCCAAGCTAAAGAAATTCGATAAGCAATATGGACCCTTTGACGAAACATACT
ATGACATATTTCAAGTAGTACCTCCTGCAACTATCAGTAACCAGGCTCTCCAAGTAACTT
CTGATTATGCCAAAAATTTAATGATCCTGTTCAATAATGCAGGAAGAATCCTCTTTGAACA
ATCCTTCAAATTGTGGGATGATAGTAGCAAAAGAGTGGCATCTTTCAACTCTTCATTCCTT
GTAAACATTTACAGGGTAAAAAATGAAACTGCAACTGAAGGTTTAACTTTCTTGATTTCTC
CTGATTTAGAGCTGCCAACCAACAGTCACTGACAGCATTTAGGCTTGACAAATGGTAATA
CTGATGGTAGATCTGCCAATAAAGTAATCGCAGTCGAGCTTGATACATTCAAGCAAGAGT
TTGACCCTGATGATAACCATATTGGGATTGATATTCATAGTGTTATTTCTGTAAAGGTCGA
ATCTTTAACCCCTCATGGAATTGAGCTTGCTGGCATTGGTGCAAGATTTTACAATATTTGG
GTACAATATGACGGTATCAAGAAAGTTCTTGATGTGTACATAGTAGAGCAAACTGAGGTA
AATGGCTCAACCCCACCTAGGCCAGAGAATCCAATATTAACACATGATGTTGATTTAAGA
GATGTTGTGAATCAAGAATCATACTTTGGATTCTCTGCTTCAACAGGGAATTTTTCCCAAT
TGAATTGTGTGTTAAGGTGGAACTTGACAGTTGAGTACTTTCAAGAAAAAAATCAATACTT
AACCATTGGTTTAGGTGTTGGGGTTCATTTGCTCTATTCTTGATTTTGTTACTACTTCTGG
GTACTATTACCACAAGAAAAAGGCTGCTAGGTCAGAGTCAATTACACTAGGTGCACTTAA
GAGTTTGCCTGGAATGCCTAGAGATTTTGAGTTTAAGGTATTGAAGAAAGCTACTAATAA
TTTTGATGAAAAGAATAAGCTAGGTGAAGGGGGATTTGGAGTGGTATACAAAGGGTATTT
AGTTGGTGAAAATTTGGAAATTGCAGTGAAGTGGTTTTCAAGGGAAAGTATCAAAGGTCA
AGATGATTTCTTGGCTGAACTTACTTTATCAACCGTCTTCGGCATAAACATCTTCAAATTG
CTTGGGTAAGTATTTTTCACTCTACTTTTACTTTTCGTATTAAGGAAAACTTGGCACAACAG
GTGTTGCTGTCTGACTAGAAAATCACAGGTTTAAGCAAAAACAGCAACATGCAGAAACTT
GAGTTCATGATCTTTTTTTAAAAACCGTGTTTTCCGGGCATCGCTTTGTGAACCCGACTA
GCTATACCCACCGGGTCCCACTGTTTCAAGGCGTGCACCACGGTTAGTTCCACGGGGC
CCTGAAGACGAGGCTGGTTCAGGGTTGAGTTCATGAGTTGAATTATTGCTTTGCTACACT
CTATTCAGTGAGAACCCATTTCTCCAGCTTCTCTCTGATCAGTTTCTCTGCCTGTTTGAGT
CCAGTGGTGTTTTCTTGCATCCTCAGGTCCTCTTTCACTATCATTAGTTGTTGTCTGCAGC
TCTTAATCTTGTACACTACTCCTGATTATTGAGTTGTATTGAGATTTTTGATTTCCTGCTTG
ACCTTCTTCAGTTTGCCTCAAATATTTTGCATGTGGCCTCTCTTGTTTATATTGGTCGATC

CTTTCTCCACACACTGCTGAAACTCTGAATGCTCCACAATGAAGTTAAAAAACCTGAATG
GTCTATAACTTTTGACATTCCATGTGATTAATTTCATCGTATATTGGAAGGTTGTTGTAGG
TAACCTGCCTTATTTTCTAGTAGATTACTAGTTTCACCTTTTATGAATAGTTACATGTAATT
ATTATTTAGCTGACTTGATAGTGTAAAAGTAAATTTACTCAATCAGTGTATATAAGTTAAAC
TCTCGTATCATTCAGAGAAAAATCACATCTATGACATTACTCTTACTAATAGCATCGCATC
AAAACAACAACAACTATCCAGTATAGTCCCGTAAAGTGAGGTCTGGGGAGAGTAATATGT
AGACGAGCCTTACCCCTATCCGAAAGGGCAGAGAGGCTGTTTCCAATCGACAATCGGTT
CAGTGAAGGAAAGGAAAAACAAAAACAAAAACAAAGACAAAAGAGCATTAGTAACTCCAG
CAGAAATAGCGTAAATAATTATTTAAACATAATAACCATAAATAAAACACTTATAGTATTAT
CTGCCTTTGTAGAAAACTTTTTTATTTGTTTTCTACCATATTATTGTGTTAACTTCAAGCTG
ATTAGGAGAGCAATAGAGACACATATCTTACAATACATATTATTGTGACAGGATGGAGCC
ACAAGCATGGAAAGCTACTACTTATATACAAGTACATGCCAAATGGTAGCCTAGACAAAC
ACCTTTTCTCAGGTCCTGATAAAAAGCCACTCAGTTGGCAAATCAGATACAAGATTATTTC
GGGCGTCGCTTCAGCCCTGCACTATCTACACGATAAGTTTTAGCAGAAAGTGATCCATC
GCGATCTTAAGGCGAACAACATCATGCTCGACTCCAAGAGCTCCAAATATCGCCCAAGA
AAGGAATAA

>NbS00012093g0021.1
MGLLTAKLLTIFIFLSCLQLKIQAQKLKTFNKEYPSFDETFFNIFEVEKPATISNGALQVTPDSAS
DDFELNNNSGRILLKQPFKLWDGDISNNKSRVASFNSSFLVNIYRPKNETAAEGLAFLISPDLE
KPLNSQGQYLGLTNATTDGNSTNKVVAVELDTFKQNFDPDGNHIGIDIHSVRSVKYESLSSH
GIELAPIGARFYNIWVQYDGIKKILDVYIAEQAEKDGSTPPRPNNPILTYNLDLKNHVNQESYF
GFAASTGSNYQLNCVMRWNLTVEYFPGKKHPWLKVVLGVGIPVVVLLLLGAAYLGYIYHRRR
VDRSQSNILGALKSLPGTPQEYKFKALKKATNNFDEKNKLGQGGYGVVYASLVEGVGIPVVV
LLLLGAAYLGYIYHRKRVDRSQSNILGALKSLPGTPQEYKFKALKKATNNFDEKNKLGQGGY
GVVYRGFLAAEDKDIAVKWFSRESIKGEDDFLAELTIINRLRHKHLVKLLGWCHKNGKLLLVY
EYMPNGSLDMHLFSGPDKQPLSWHVRYKIMQGVASALHYLHNEYEQRVVHRDLKASNIMLD
SQFNARLGDFGLARALDNERTSYAEAEGVLGTMGYIAPECFHTGKATSQSDVYAFGAVLLE
VVCGQRPGTKVNGFQFFVDWVWYLHRDGRILEAVDQRLGDDYVAEEAKKLLLLGLACSHPI
ASERPKTQTIVQIISGSAPAPDVPPFKPAFVWPSMMPMDIDSSVMDTTSITTSHFNSGWSLD
YQSRETPTYADHPNSLV

>NbS00012093g0021.1 Corrected; GenBank ID: KT225333
MGLLTAKLLTIFIFLSCLQLKIQAQKLKTFNKEYPSFDETFFNIFEVEKPATISNGALQVTPDSAS
DDFELNNNSGRILLKQPFKLWDGDISNNKSRVASFNSSFLVNIYRPKNETAAEGLAFLISPDLE
KPLNSQSQYLGLTNATSDGNSTNKVIAVELDTFKQNFDPDDNHIGIDIHSIRSVKYESLSSHGI
ELAPIGARFYNVWVQYDGIKKILDVYIVEQAEKNGSTPPRPNNPILTYNLDLKDHVNQESYFG
FSASTGSNYQLNCVLRWNLTVEYFPDKKHPWLKVVLGVGIPVVVLLLLGAAYLGYIYHRKRV
DRSQSNILGALKSLPGTPQEYKFKALKKATNNFDEKNKLGQGGYGVVYRGFLAAEDKDIAVK
WFSRESIKGEDDFLAELTIINRLRHKHLVKLLGWCHKNGKLLLVYEYMPNGSLDMHLFSGPD
KQPLSWHVRYKIMQGVASALHYLHNEYEQRVVHRDLKASNIMLDSQFNARLGDFGLARALD
NERTSYAEAEGVLGTMGYIAPECFHTGKATSQSDVYAFGAVLLEVVCGQRPGTKVNGFQFF
VDWVWYLHRDGRILEAVDQRLGDDYVAEEAKKLLLLGLACSHPIASERPKTQTIVQIISGSAP
APDVPPFKPAFVWPSMMPMDIDSSVMDTTSITTSHFNSGWSLDYQSRETPTYADHPNSLV

>NbS00012093g0021.1 Corrected; GenBank ID: KT225333
ATGGGATTGTTGACAGCAAAATTACTCACCATTTTCATTTTTCTCAGCTGTCTTCAACTCA
AGATTCAAGCCCAGAAACTGAAAACGTTCAACAAAGAATATCCATCTTTTGATGAAACTTT
CTTTAACATTTTTGAGGTTGAAAAACCTGCAACAATCAGTAACGGTGCTCTTCAGGTGAC
TCCTGACTCTGCATCTGATGATTTTGAACTCAATAATAACTCGGGAAGAATCCTCTTGAAA
CAGCCATTTAAACTCTGGGATGGTGATATTTCAAACAATAAAAGCAGGGTTGCATCCTTC
AACTCTTCTTTTCTTGTAAATATTTATAGGCCAAAGAATGAAACAGCTGCTGAAGGTTTAG
CATTCTTGATTTCTCCAGATTTAGAGAAGCCACTCAATAGCCAAGGGCAGTATTTGGGTT
TGACAAATGCTACTACTGATGGAAATTCCACCAATAAAGTTGTTGCTGTTGAGCTTGATA
CTTTCAAGCAAAACTTTGACCCTGATGGCAACCATATTGGTATTGATATTCACAGTGTTAG
ATCTGTAAAGTATGAATCTTTGTCATCTCATGGGATTGAGCTTGCTCCTATTGGTGCAAG
ATTCTATAATATTTGGGTACAATATGATGGAATCAAGAAAATTCTTGATGTGTATATTGCA
GAACAAGCTGAAAAAGATGGCTCTACACCACCTAGGCCAAACAACCCTATATTAACATAT
AATCTTGATTTAAAAAACCATGTGAATCAAGAATCATATTTTGGATTTGCTGCATCAACTG
GGAGTAATTACCAACTCAACTGTGTGATGAGGTGGAACTTAACAGTTGAGTACTTTCCAG

GTAAAAAGCATCCATGGTTGAAGGTTGTTTTAGGGGTTGGGATTCCAGTGGTGGTGTTAT
TACTACTTGGGGCAGCATATTTAGGGTATATTTATCACAGGAGAAGAGTGGATAGGTCAC
AGTCAAACATATTGGGTGCACTAAAGAGTTTGCCTGGAACCCCACAAGAGTATAAGTTCA
AGGCTTTGAAAAAAGCTACCAACAATTTTGATGAAAAGAATAAGCTTGGGCAAGGGGGAT
ATGGGGTGGTTTACAGAGGATTTTTAGCTGCTGAGGATAAAGATATTGCAGTCAAGTGGT
TTTCAAGGGAAAGTATCAAAGGTGAAGATGATTTCTTGGCTGAACTTACCATCATTAATC
GTCTCAGGCATAAACATCTTGTCAAATTGCTTGGTAAGTCTTTTTATTTTTGTTTGAATGTA
GATATTGGAATGTTTCTTACCATGAATTTCATCTGTTTGAAAAGAGTAAAGAGTTATGTTA
TGCATGTTTTTAGTATGATCTTGGTAGTAGGCTCAGCATGACAACATATTAGGTTTAAAAG
CAGTTAGGAAAATGGTACTAATAATTGTCCATCGTATAAGAGAGTACAAGAAAATGAGCT
TTTGATGGCAACTACAAAGATTGTGTTCGCGCACAGCTTTTAGCTAAACCTTTGAGTCAC
AATGAGGCACATCTCCCAGTATTTCCATTGAAGTGATAAATTTGTTACCTTTCTCGGGGA
AAAAAAATAGTCCGGTGCACTAAATACTCATTATGTGTGGGGTCCGGGGAAGGCCAGAC
CACAAGGGTTTATTATTCACAAACTTACCCTGTATTTTTACAAGAGGTTGTTTCCACGCCT
TAAACTCATGATCTCCCGATCACATGACAACAGTTTTACCATTTACGCCAAAACTCCCCTT
TCCTTTCTTTAAAAAGAAAATCACTGATATAAATGTTGTTCACAAGGTCGGAGTTGTATGA
AGGGGTTTAATTGAATCCTGTTTGCTGGAGAAGTAAAATGTGCAAATATGATAAAATTGAT
TCTTTGTGTATAAAGAAAGTTTACAGTGTAGCAGCAAGGTGGTTCAAAAATGTATTTAGGT
CGTAGATTTGAATCCACTGTGATAATCTTGCATTTTCTTAAATTTAGGATTCTATCTCTGC
CCCTACCATAATTACGTAATAGAGACGGGGACAAAGGAAAGGGGCCTTGTTTTATTGAC
GTTTAGCATTTAGTAATCAGATACAAAATTCTGGTATGTTCTTGATTCCCAACGAAAAAGA
GAATTCATTAACAAGGACTCTACTGAGAGAATGGTTCCAAAAGTTATTGAGATAATTTAAA
AGCAAAGAGTGGTGGGGCATCAACTTTAATGGTAAATTTGGTACTAATAGACTGATTAAC
CGCGTGGCAAGAATCCACTGGGCACATGCCTTATCTTTTTATGCTTTTCCTTTTCCAGTG
ACTTTCCACTCAGATATCTGTGAGCTTTCTTTTATTCGACTAAGTTGCGAAATGTTGATGT
TGGACTTGTCTTTCTAGTCTGGCATTTAGCTTGCTAGGAATTCTATAAACATTTTTAGACA
TTTAATGTGAGGAGTGAGAAAACTTTTTAAAGATATATACGTACGGATTATTTTGTAAAAA
CAAATTGAATTCTTTCTTGATTACATAACTGACACTTATTTTAAACTAAAATAAAAAAGTAA
ATTGGACACTCATTGTGAACCGAAGGGAGTACTAAATTGGATTTTCTGAGAAGCAGATTT
GTCGGTTTGAAGAATCAAAGCCTTCTTGAACCCTTGTAATTTAACTTGGTCCTCCTTTTGT
CTTTATTCTCTTTCCTTTATTGGAATTCTCGAGTCATCCCAAACGATTTGTCAAAGCTTCT
CCTTGAGGCTCCGGTTATTAGCGTAGCCGAAATTTAATAGCCCGATTAATTAGCCTCGTG
CTGGGGTTGCTCTGATGGTAATCAACCCCACTTCTAACCAAGAGGTTGTTAGTTCGAGTC
TCTCCAAGATCAAGGTGGGAAGTTCTTGGAGGGAAGGATGCTGGGGTCTATTTGGAAAC
AGCCTCTCTACCCAAGGTAAGGTCTGTGTACACAGTACCCTCCCCAGACCCCACTAAGT
GGGATTATACTGGGTTATTGTTGTTGTTGTTGTGCTGGATTGGATTCTTCCTATCAAGAG
CTTTCCTTAAGAATGGCGTGAAGCCTAGACTTCCGGTTAAAGATGGAAGAAACATATTCA
TCTCTTAATAGTTGATTCCTGCAATATGATAAGTGATAGTTTATCCTATATAAAATCATGGA
AGTAGGAAAAACTAATGGAATGACCTTAAATATTTTGACCAAGAACCAACTTGCTGCTAT
GTCCCCTCCAATTTAGAACATTGAATCTCAGCAGCCATTCGTCAAACCATTCTTTTATGCC
ATATATGTCAATATTTTGAGGAAGCTTCCTTCTTTGTCCTAACATACCTTTCAATGCATTGT
CAATGATTGAGACAAGTTTTTGAATGTTAAATAAAGTGATGATAATTATATCATCAACTTC
GTATTTTAGTATACAATCATCTTCAAACACCTAATCTTTTGTGTCAATCTACTCTTGTCATC
TGACCCTACAAAGAGATAAAGTTCATGACTTTCTTTTATAAACTACAGAATAAAGTTGACC
GTCTGCAATTCTTTCCTCTATTTGATGAACTTATACATCTTTGTTACTGAATCACTGGATAA
TTTTTATTGATAAAAGACAATCTTACTGACATAAGCATCTTATAATTGCATGTTATGGTAAC
AGGATGGTGCCACAAGAATGGAAAACTACTGCTTGTATACGAATACATGCCTAATGGCA
GCCTAGATATGCACCTTTTTTCGGGGCCAGATAAGCAGCCCCTGAGCTGGCATGTCCGC
TACAAGATTATGCAAGGTGTTGCCTCAGCATTGCACTATCTGCACAATGAGTATGAGCAG
AGGGTGGTCCATCGCGATCTAAAGGCAAGTAACATAATGCTCGACTCTCAGTTCAATGC
ACGCCTTGGGGATTTTGGCCTTGCACGAGCCCTTGACAACGAAAGGACCTCGTATGCTG
AGGCCGAGGGAGTGCTTGGCACAATGGGATACATAGCACCAGAGTGTTTCCACACTGG
GAAAGCCACATCACAATCTGATGTTTACGCATTTGGGGCCGTGTTGTTGGAAGTTGTATG
CGGCCAAAGACCTGGAACCAAGGTTAATGGCTTTCAATTCTTTGTTGATTGGGTTTGGTA
CTTGCATCGGGATGGTCGTATCCTAGAAGCTGTTGATCAAAGGCTTGGAGATGACTATG
TTGCTGAAGAAGCAAAGAAACTTCTGCTGTTAGGTTTGGCTTGCTCACATCCAATTGCAA
GTGAGAGACCTAAAACACAGACAATAGTTCAGATTATATCAGGGTCAGCACCAGCACCA
GATGTTCCACCATTTAAGCCAGCATTTGTATGGCCATCTATGATGCCAATGGACATAGAT
TCTAGTGTTATGGACACAACATCTATTACAACTTCTCACTTCAATTCAGGATGGAGTCTTG
ACTATCAAAGCAGGGAGACCCCAACATATGCAGACCACCCCAACTCATTGGTGTAG

>NbS00020337g0016.1 GenBank ID: KT225334
MGLLTAKKLTIFVLLCCLQLKIQAQKLKTFNREYLSFDETFFNIFEVEKPATISNGALQVTPDSA
SEDFELNNNSGRILLKQPFKLWDGDISNNKSRVASFNSSFLVNIYRPKNETAAEGLAFLISPDL
EKPLNSQSQYLGLTNATSDGNSTNKVIAVELDTFKQNFDPDDNHIGIDIHSIRSVKYESLSSHG
IELAPIGARFYNVWVQYDGIKKILDVYIVEQAEKNGSTPPRPNNPILTYNLDLKDHVNQESYFG
FSASTGSNYQLNCVLRWNLTVEYFPDKKHPWLKVVLGVGIPVVVLLLLGAAYLGYIYHRKRV
DRSQSNILGALKSLPGTPQEYKFKALKKATNNFDEKNKLGQGGYGVVYRGFLAAEDKDIAVK
WFSRESIKGEDDFLAELTIINRLRHKHLVKLLGWCHKNGKLLLVYEYMPNGSLDMHLFSGPD
KQPLSWHVRYKIVQGVASALHYLHNEYEQRVVHRDLKASNIMLDSKFNARLGDFGLARALD
NERTSYAEAEGVLGTMGYIAPECFHTGKATSQSDVYAFGAVLLEVVCGQRPGTKVNGFQFF
VDWVWYLHRDGRILEAVDQRLGDDYVAEEAKKLLLLGLACSHPIASERPKTQTIVQIISGSAP
TPEVPPFKPAFVWPSMMPTDIDSSVMDTTSITTSHFNSGWSLDYQSRETPTYADHPNSLV

>NbS00020337g0016.1 GenBank ID: KT225334
ATGGGATTATTGACCGCAAAAAAACTCACCATTTTCGTCTTATTGTGCTGTCTTCAACTCA
AAATTCAAGCCCAGAAACTGAAAACTTTCAACAGAGAGTATCTATCTTTTGATGAAACCTT
CTTTAACATATTTGAGGTTGAAAAGCCTGCAACAATCAGCAACGGTGCTCTTCAGGTGAC
TCCTGACTCTGCATCTGAAGATTTCGAACTCAATAATAACTCAGGAAGAATCCTCTTGAAA
CAGCCATTTAAACTCTGGGATGGTGATATTTCAAACAATAAAAGCAGGGTTGCATCTTTC
AACTCTTCTTTTCTTGTAAATATTTATAGGCCAAAGAATGAAACAGCTGCTGAAGGTTTAG
CATTCTTGATTTCTCCAGATTTGGAGAAGCCACTCAATAGCCAAAGCCAATATTTGGGTTT
GACAAATGCTACTAGTGATGGAAATTCCACAAATAAAGTTATTGCTGTTGAGCTTGATACT
TTTAAGCAGAACTTTGACCCTGATGATAACCATATTGGTATTGATATTCACAGTATTAGAT
CTGTAAAGTATGAATCTTTATCATCCCATGGGATTGAGCTTGCTCCTATTGGTGCAAGATT
CTATAATGTTTGGGTACAATATGATGGTATCAAGAAAATTCTTGATGTGTATATTGTAGAA
CAAGCTGAAAAAAATGGCTCTACACCACCTAGGCCAAATAACCCCATCTTAACATATAAT
CTTGATTTAAAAGACCATGTGAATCAAGAATCATATTTTGGATTTTCTGCATCAACTGGGA
GTAATTACCAACTCAACTGTGTGCTGAGGTGGAACTTAACAGTTGAGTATTTTCCAGATA
AAAAGCATCCTTGGTTGAAGGTTGTATTAGGGGTTGGAATTCCAGTGGTGGTGTTATTGC
TACTTGGGGCAGCATATTTAGGGTATATTTATCACAGGAAAAGAGTTGATAGGTCACAGT
CAAACATATTGGGTGCCCTAAAGAGTTTGCCTGGAACCCCACAAGAGTATAAGTTCAAG
GCTCTGAAAAAAGCTACTAACAATTTTGATGAAAAGAATAAGCTTGGGCAAGGTGGATAT
GGAGTGGTTTATAGAGGATTTTTAGCTGCTGAGGATAAAGATATTGCAGTCAAGTGGTTT
TCAAGGGAAAGTATCAAAGGTGAAGATGATTTCTTGGCTGAACTTACTATCATTAATCGT
CTCAGGCATAAACATCTTGTCAAATTACTTGGTAAGTCTTTTCATTTTTGTTTGAATGTAGA
TATTGGAATGTTTCTTACCATGAATTTCATCTGTTTGAAAAGAGTAAAGAGTTATGTTATG
CATGTTTCTAGTATGATCTTGGTAGTAGGTTCAGCGTGACAACATATTAGGTTTAAAAGCA
GTTAGGAAAATGGTACTAATAGTTGTCCATCGTATAAGATAATACAAGAGAATGATCTTTT
GATGGCAACTGCAAAGATTGCGTTCGCACACAGCTTTTAGCTAACCTTTGAGAGTCACAA
TGAGGACACATCTCCCAGTATTTCCATTGAAGTGATATAAATTTGTTACCTTTCTCGGAAA
AAAAAATAGTCTGGTGCACTAAACTCCCACTATGCGTTGGGTCCGGGGAAGGTCAGACC
ACAAGGGTTTATTGTGCGCAACCTTACCCTGCATTTTTACAAGAGGTTATTTCCATGGCT
CATACCCATGACCTTTTGGTCATATAACAACGGTTTTACCAATTACGCCATACTCTCCTTT
ATCTTTCTTTAAAAAAATTACTGGGTCGGAATTGTATGAAGGGGTTTAATTGAATCCTCTT
TGTTGGAGAAGTAAAGTGTGCAAATATGATAAAATTGATTCTTTTTATATAAAAAAAAGTTT
ACAGTATAGCAGGAAGTTGGTTCAAAAATATATTTAGGTCATGGGTTTGAATCCACTGTG
ATAATCTTGCATTTTCTTAAATTTAGAATTCTTTCTCTGCCCCTTCCATAATTAGGTGATAT
AGACCGGGAGACGTGCTGCATTGGATTCACTAAAGTGAGAAACGCTTCCTATCAAGAGT
TTTCCTAAAGGATGACTTGAAGCCTAGACTTCCGGTTAAAGATGGAGGAATCATATTCAT
CACTTAATAGTTGATTCCAGCAATATGATAAGTGATAGTTTATGCCATATAACATCATGGA
AGTAGGAAAAACTAATGGTGACCAAGAACCAACTTGCTGCAATGTCCCATCCAATTTTGA
ACATTGAATCTCCTCATATGATTTTCTTGAAAGAGAACAAAAAGAAGATGATCATCTTTGG
CTGACCGTGGAATATGCCCTAAGCAGCCATTCGTTAAACCATTTTTATGCCATATTTATAT
GTCAATATTTTGAGGAAGCTTCCTTCTTTGTCCTAACATACCTTTCAATGCATTGTCAATG
ATTGAGACAAGTTTTTGAATGTTAAATTAAATGATGATAATTACATCCACTGAAAAGGACA
TCAACTTCGTATTTTAGTATACAGTCATCTTCAAACACGTATTCTTTTGTGTCATCTACTCT
TGTCATCTGACCCTACAAAGAGATAAAGTTCGTGACTTCCTTTTTATAAACTACAAAATGA
AGTTGACCATCTGGAATTTGTTCTCTGTTTGATAAACTTATACATCTTGTTACTGAATCACT

GAATAATTTTTATTGATAAAAAAAACAATCTTACTGACATAAGCATCTTATAATTGCATGTT
ATGGTAACAGGATGGTGCCACAAGAATGGAAAACTACTGCTTGTATATGAATACATGCCT
AATGGCAGCCTAGATATGCACCTCTTTTCGGGCCCAGATAAGCAGCCGCTTAGCTGGCA
TGTCCGCTACAAAATTGTGCAAGGTGTCGCCTCAGCATTGCACTATCTGCACAACGAGTA
CGAGCAGAGGGTGGTGCATCGCGATCTAAAGGCAAGTAACATAATGCTCGACTCCAAGT
TCAATGCGCGCCTTGGGGATTTTGGCCTTGCACGTGCCCTTGACAACGAAAGGACCTCA
TATGCTGAGGCTGAGGGAGTGCTTGGCACTATGGGATACATAGCACCAGAGTGTTTCCA
CACCGGTAAAGCCACATCGCAATCTGATGTTTACGCATTTGGAGCCGTGCTGTTGGAAG
TTGTATGTGGCCAAAGACCTGGAACCAAGGTTAATGGCTTTCAATTCTTTGTTGATTGGG
TATGGTACTTGCATCGCGACGGCCGTATCCTAGAAGCTGTTGATCAAAGGCTTGGAGAT
GACTATGTTGCTGAAGAAGCAAAGAAACTTCTGCTATTAGGTTTGGCTTGCTCACATCCA
ATAGCAAGTGAGAGGCCTAAAACACAGACAATAGTTCAAATTATATCAGGGTCAGCACCA
ACACCAGAAGTTCCGCCATTTAAGCCGGCGTTTGTATGGCCATCTATGATGCCGACGGA
CATAGACTCTAGTGTTATGGACACAACATCTATTACAACTTCTCACTTCAATTCAGGATGG
AGTCTTGACTATCAAAGCAGGGAGACCCCAACATATGCAGATCACCCCAACTCATTGGT
GTAG

>NbS00006201g0004.1
MHFSLQKLLTVTLFLILTINSPSLSSPFFPLNNVTLYGDASFTTNSIYLTQQRNCSSNSPSISAI
GRAFYVYPIRFLDSLTNNTASFLCTFSFTILPTTPSCPFGDGFAFLISSDVDSLTNSNGYMGLP
NRDSDMGDSFLAVEFDTNDNHIGIGVKEVKFLASADVNLKSGKELTAWIEYKDSEKMMRVWI
GYEMQIRPFNPVLATKIDISNQLKEFMRTGFTAKGSAVYSINRWRFRTFGLLSSQTSWDHQS
DEGDCLMCFPEEIGEHFSAPHHSKSLLKLIFVYGGLAAVVTLVVGILAIVSVFVLRRKKRDGSR
GENEGQMCRFQGNRVPQRLSLSEIKSATEGFNNERIIGEGGSAVVYEGDIPSKGTVAIKRFV
QGTRLGPSHIPFNTEFASMVGCLRHKNLIQFQGWCCERNELVLVYEFMPNGSLNKILHEHSH
FAKFLTWERRLNIVLGVASALVYLHEECENQIIHRDVKTCNIMLDAEFNACVFA

>NbS00006201g0004.1 Corrected; GenBank ID: KT225335
MHFSLQKLLTVTLFLILTINSPSLSSPFFPLNNVTLYGDASFTTNSIYLTQQRNCSSNSPSISAI
GRAFYVYPIRFLDSLTNNTASFLCTFSFTILPTTPSCPFGDGFAFLISSDVDSLTNSNGYMGLP
NRDSDMGDSFLAVEFDTNDNHIGIGVKEVKFLASADVDLKSGKELTAWIEYKDSEKMMRVWI
GYEMQIRPFNPVLATKIDISNQLKEFMRTGFTAKGSAVYSINRWRFRTFGLLSSQTSWDHQS
DEGDCLMCFPEEIGEHFSAPHHSKSLLKLIFVYGGLAAVVTLVVGILAIVSVFVLRRKKRDGSR
GENEGQMCRFQGNRVPQRLSLSEIKSATEGFNNERIIGEGGSAVVYEGDIPSKGTVAIKRFV
QGTRLGPSHIPFNTEFASMVGCLRHKNLIQFQGWCCERNELVLVYEFMPNGSLNKILHEHSH
FAKFLTWERRLNIVLGVASALVYLHEECENQIIHRDVKTCNIMLDAEFNAKLGDFGLAEVFDN
SRTRDATVPAGTMGYLAPEYVFSGVPTVKTDVYSFGVVVLEVASGRKPIDECGGLTTDWVW
DLWEKGRITEAADPKLKGRFLKNEMDRVLVVGLSCVHPNQEKRPRMREVSRMLKDEAPLPI
LPPKKPSVRIQSVLPEGCEEIMNCAGKMEDSPWATPRTHFSRN

>NbS00006201g0004.1 Corrected; GenBank ID: KT225335
ATGCATTTTTCTTTGCAAAAACTCTTAACAGTCACTCTATTTCTCATCTTAACCATCAATTC
ACCTTCCCTTTCATCTCCTTTCTTCCCTCTTAACAATGTAACTCTTTACGGTGACGCTTCT
TTCACCACCAACTCCATTTACCTCACTCAACAGCGCAACTGTTCATCAAATTCACCTTCCA
TTTCTGCCATTGGTAGAGCTTTCTATGTTTACCCCATTCGTTTTCTTGATTCTTTAACAAAT
AACACTGCTTCTTTCTTATGCACTTTCTCTTTTACTATTCTCCCTACTACCCCTTCTTGCCC
TTTTGGTGATGGCTTTGCCTTTTTGATCAGTTCTGATGTTGATTCTTTGACCAACTCTAAT
GGTTACATGGGTCTTCCCAATCGAGATTCTGACATGGGAGATTCATTCTTGGCCGTGGA
ATTTGATACAAACGATAATCATATTGGTATTGGTGTTAAAGAAGTTAAGTTTTTGGCTTCT
GCTGATGTTGATTTGAAAAGTGGGAAAGAATTGACGGCTTGGATTGAGTATAAAGATTCT
GAAAAAATGATGAGAGTTTGGATTGGTTATGAAATGCAAATTAGGCCTTTTAATCCTGTTC
TCGCTACTAAAATTGACATTTCCAATCAGTTGAAGGAGTTTATGAGAACTGGTTTCACTGC
AAAAGGCTCTGCAGTTTACAGCATTAATCGTTGGCGATTCAGAACGTTCGGATTGCTTTC
GTCTCAAACGTCTTGGGATCATCAATCCGATGAAGGAGATTGCTTGATGTGTTTTCCTGA
GGAAATTGGTGAGCATTTTTCTGCCCCTCATCATAGTAAAAGTTTACTGAAATTGATTTTT
GTATATGGTGGCTTAGCTGCAGTTGTTACACTTGTTGTTGGTATTCTGGCTATTGTGTCT
GTTTTTGTGTTAAGGAGAAAAAAAAGGGATGGTAGTAGAGGGGAAAATGAAGGTCAAAT
GTGTAGATTCCAAGGAAATAGAGTGCCTCAAAGATTGTCATTATCTGAAATAAAATCAGC
CACAGAAGGATTTAATAATGAAAGGATAATTGGTGAAGGAGGATCTGCTGTTGTATATGA
AGGAGATATTCCTTCTAAGGGAACTGTTGCTATTAAGAGATTTGTTCAAGGGACTAGATT

AGGTCCTTCACATATTCCATTTAACACTGAATTTGCTTCTATGGTTGGCTGCTTAAGACAC
AAGAATTTGATTCAGTTCCAAGGATGGTGTTGTGAGAGGAATGAGTTGGTTTTAGTTTAT
GAATTCATGCCTAATGGTAGCCTTAACAAAATCCTACACGAACACTCGCATTTTGCTAAG
TTTCTGACATGGGAGCGAAGGCTGAATATAGTTCTTGGCGTTGCGTCTGCTCTGGTGTAT
TTGCATGAAGAGTGTGAGAATCAAATAATTCATAGAGATGTGAAGACTTGTAATATAATGC
TTGATGCTGAGTTCAATGCTAAGCTTGGGGATTTCGGTTTAGCTGAAGTGTTTGATAATT
CCAGGACAAGGGATGCTACTGTACCAGCTGGAACAATGGGATATTTAGCTCCTGAATAC
GTCTTTTCTGGCGTTCCAACTGTCAAAACGGATGTGTATAGCTTTGGTGTTGTGGTACTA
GAAGTAGCATCGGGGAGAAAGCCTATCGATGAATGTGGGGGTTTGACTACTGATTGGGT
GTGGGATTTGTGGGAAAAGGGGAGGATAACTGAGGCTGCTGATCCGAAACTAAAGGGA
CGGTTTCTGAAGAACGAGATGGATAGGGTGCTCGTCGTGGGACTTTCTTGTGTGCACCC
TAATCAGGAGAAGAGGCCGAGAATGAGGGAAGTTTCCCGTATGCTTAAAGATGAAGCTC
CCCTGCCCATTTTGCCTCCAAAGAAACCAAGTGTGAGAATTCAATCTGTTTTACCAGAGG
GATGTGAAGAAATCATGAATTGTGCTGGAAAAATGGAGGATAGCCCATGGGCAACTCCA
AGAACTCATTTTAGCAGGAACTAG

>NbS00021029g0001.1
MHFSLQKLLTVTLFLVLTINSPSLSSPFSPLNNVTVYGDASFTTNSICLTQQRNCSSNSPSISAI
GRAFYVYPIRFLDSLTNNTASFLCTFSFTILPTTPSCPFGDGFAFLISSDVDSLSNSNGYMGLP
KQDSDMGDSFLAVEFDTNDNHIGIGVKEVKFLVSADVNLKSGKELTAWIEYKDSEKMMRVWI
GYEMQIRPFNPVLATKIDISNQLKEFMRIGFTAKGSVVYSINRWRFRTFGLLSSQTSWDHQS
DEGDCLMCFPEEIGEHSSAPHHGKSLLKLIFVYGGLAAVVTLVVGVLAIVSVFVLRRKKRDSS
RGDNEGQMCRLQGNRVPQRLSLSEIKSATEGFNNERIIGEGGSAVVYEGDIPSKGTVAIKRF
VQGTRLGPSHIPFNTEFA

>NbS00021029g0001.1 Corrected; GenBank ID: KT225336
MHFSLQKLLTVTLFLVLTINSPSLSSPFSPLNNVTVYGDASFTTNSICLTQQRNCSSNSPSISAI
GRAFYVYPIRFLDSLTNNTASFLCTFSFTILPTTPSCPFGDGFAFLITSDVDSLSNSNGYMGLP
KQDSDMGDSFLAVEFDTNDNHIGIGVKEVKFLVSADVNLKSGKELTAWIEYKDSEKMMRVWI
GYEMQIRPFNPVLATKIDISNQLKEFMRIGFTAKGSVVYSINRWRFRTFGLLSSQTSWDHQS
DEGDCLMCFPEEIGEHSSAPHHGKSLLKLIFVYGGLAAVVTLVVGVLAIVSVFVLRRKKRDSS
RGDNEGQMCRLQGNRVPQRLSLSEIKSATEGFNNERIIGEGGSAVVYEGDIPSKGAVAIKRF
VQGTRLGPSRIPFNTEFASMVGCLRHKNLIQLQGWCCERNELVLVYEFMPNGSLNKILHEHS
HFAKFLTWERRLNIVLGIASALVYLHEECENQIIHRDVKTCNIMLDAEFNAKLGDFGLAEVFDN
SRTRDATVPAGTMGYLAPEYVFSGVPTVKTDVYSFGVVVLEVASGRKPIDECGGLITDWVW
DLWEKGRITEAADPKLNGRFQKNEMDRVLVVGLSCVHPNEEKRPKMREVARMLKEEAPLPI
LPPKKPTVRIQSVLPEGCEEIMNCAGKLEDSPWATPRTHFSRN

>NbS00021029g0001.1 Corrected; GenBank ID: KT225336
ATGCATTTTTCTTTGCAAAAACTCTTAACAGTCACTCTGTTTCTCGTATTAACCATCAATTC
ACCTTCCCTTTCATCTCCTTTCTCCCCTCTTAACAATGTAACTGTTTACGGCGACGCTTCT
TTCACCACCAACTCCATTTGCCTCACTCAACAACGCAACTGTTCATCAAATTCACCTTCCA
TTTCTGCCATTGGTAGAGCTTTCTATGTTTACCCAATTCGTTTTCTTGATTCTTTAACAAAT
AACACTGCTTCTTTCTTATGCACTTTCTCTTTTACTATTCTCCCTACTACCCCTTCTTGCCC
TTTTGGTGATGGCTTTGCCTTTTTGATCACTTCTGATGTTGATTCCTTGTCCAACTCTAAT
GGTTACATGGGTCTTCCAAAGCAAGATTCTGATATGGGAGATTCATTCTTGGCCGTGGAA
TTTGATACAAACGATAATCATATAGGTATTGGTGTTAAAGAAGTTAAGTTTTTGGTTTCTG
CTGATGTTAATTTGAAAAGTGGGAAAGAATTGACGGCTTGGATTGAGTATAAAGATTCTG
AAAAAATGATGAGAGTTTGGATTGGTTATGAAATGCAAATTAGGCCTTTTAATCCTGTTCT
CGCTACTAAAATAGACATTTCCAATCAGTTGAAGGAGTTTATGAGAATTGGTTTCACTGCA
AAAGGCTCTGTAGTTTACAGCATTAATCGTTGGCGATTCAGAACTTTCGGATTGCTTTCC
TCTCAAACGTCTTGGGATCATCAATCTGACGAAGGAGATTGCTTGATGTGTTTTCCTGAG
GAAATAGGTGAGCATTCTTCTGCCCCGCATCATGGTAAAAGTTTACTGAAATTGATTTTTG
TATATGGTGGCTTAGCTGCAGTTGTTACACTTGTTGTTGGTGTTCTGGCTATTGTGTCTG
TTTTTGTGTTAAGGAGAAAAAAAAGGGATAGTAGTAGAGGGGATAATGAAGGCCAAATGT
GTAGATTACAAGGAAATCGAGTGCCTCAAAGATTGTCATTATCTGAAATAAAATCAGCCA
CAGAAGGATTTAATAATGAAAGGATAATTGGTGAAGGAGGATCTGCTGTTGTATATGAAG
GAGATATTCCTTCTAAAGGAGCTGTTGCAATTAAGAGATTTGTTCAAGGGACTAGATTAG
GTCCTTCACGTATTCCATTTAATACTGAATTTGCTTCAATGGTTGGTTGCTTAAGACACAA
GAATCTGATTCAGCTCCAAGGATGGTGTTGTGAGAGGAATGAGTTGGTTTTAGTTTATGA

ATTCATGCCTAATGGTAGCCTTAACAAAATCCTACACGAGCACTCGCATTTTGCTAAGTTT
TTGACGTGGGAGCGAAGGCTGAATATAGTTCTTGGCATTGCGTCTGCTCTGGTGTATTT
GCATGAGGAATGTGAGAATCAGATAATTCATAGAGATGTGAAGACTTGTAATATAATGCT
TGATGCTGAGTTCAATGCTAAGCTTGGGGATTTCGGTTTAGCTGAAGTGTTTGATAATTC
TAGGACAAGGGATGCTACTGTACCAGCTGGAACAATGGGATATTTAGCTCCTGAATACG
TCTTTTCTGGCGTTCCAACTGTTAAAACGGATGTGTATAGCTTTGGTGTTGTGGTACTAG
AAGTAGCATCAGGGAGAAAGCCTATCGATGAATGTGGGGGTTTGATTACTGATTGGGTG
TGGGATTTGTGGGAAAAGGGGAGGATAACTGAGGCTGCTGATCCAAAACTAAATGGACG
CTTTCAGAAGAACGAGATGGATAGGGTGCTCGTCGTAGGACTTTCTTGTGTGCACCCTA
ATGAGGAGAAGAGGCCGAAAATGAGGGAAGTTGCCCGTATGCTTAAAGAAGAAGCTCCA
CTGCCCATTTTGCCTCCAAAGAAACCAACTGTGAGAATTCAATCTGTTTTACCAGAGGGA
TGTGAAGAAATCATGAATTGTGCTGGAAAATTGGAGGATAGCCCATGGGCAACTCCAAG
AACTCATTTTAGCAGGAATTAG

>NbS00007030g0016.1
MSKLFSCSIFFSFLYCALLVSAQNCSFDLESFTLRNFTLLGDSYLRNGVVGLTRELQVPSSSS
GSLIYNNPISFFDPESKKIVSFSTRFAFSVNNINPSSFGVTPFHSLIKNLKRLFAFSVNNINPSSF
GDGLAFFLSPDNQTLGSPGGFLGLVNSSQLTKNKFVAVEFDTRQDLHFNDPDDNHVGLDID
SLISIKTANVKLAGVDLKLITSWIDYKSEEKQLLVFLSYSSSKSKKPLLTVDIDLSHYLKEFMYV
GFAASTEGSTELHCIENWSFQTFGFSPMRPRHNPHDVSDNSVLVKPPTIQEDSDNKHHKRL
GLGLGIAGPAFFCAVLVAFGWISLQKWRGLNTEKNLTAELVTGPRQFSYKELRSATRGFHSS
RIIGNGAFGTVYKAFFMDSSSIAAVKRSKHSHESKTEFGAELSIIACLRHKNLVQLQGWCIEKG
ELLLVYDFMPNGSLDKVLYQESENGNPLKWPYRYNIAVGLASVLTYLHQECEQQVIHRDIKA
SNIMLDVSCII

>NbS00007030g0016.1 Corrected; GenBank ID: KT225337
MSKLFSCSIFFSFLYCALLVSAQNCSFDLESFTLRNFTLLGDSYLRNGVVGLTRELQVPSSSS
GSLIYNNPISFFDQESKKTVSFSTRFAFSVNNINPSSFGDGLAFFLSPDNQTLGSPGGFLGLV
NSSQLTKNKFVAVEFDTRQDLHFNDPDDNHVGLDIDSLISIKTANVKLAGVDLKLITSWIDYKS
EEKQLLVFLSYSSSKSKKPLLTVDIDLSHYLKEFMYVGFAASTEGSTELHCIENWSFQTFGFS
PMRPRHNPHDVSDNSVLVKPPTIQEDSDNKHHKRLGLGLGIAGPAFFCAVLVAFGWISLQK
WRGLNTEKNLTAELVTGPRQFSYKELRSATRGFHSSRIIGNGAFGTVYKAFFMDSSSIAAVK
RSKHSHESKTEFGAELSIIACLRHKNLVQLQGWCIEKGELLLVYDFMPNGSLDKVLYQESEN
GNPLKWPYRYNIAVGLASVLTYLHQECEQQVIHRDIKASNIMLDVSYNARLGDFGLARLMDH
DKSPVSTLTAGTMGYLAPEYLQYGKATERTDVFSYGVVILEVACGRRPIEGEGSGHEMVNLV
DWVWRLYSEGRIIEAADKRLNGDFKEEEMRKLLLVGLSCANPDSMERPCMRRVFQILNNEA
EPIFVPKVKPMLTFSTSLPFSINDIFSDDEDNEAAPEPEFEIRVD

>NbS00007030g0016.1 Corrected; GenBank ID: KT225337
ATGAGTAAGCTTTTTTCTTGCTCCATTTTCTTCAGCTTCTTGTACTGTGCACTATTGGTAT
CAGCACAAAATTGCAGCTTTGATTTGGAATCTTTTACACTGAGAAATTTCACACTTCTTGG
AGATTCTTATCTCAGAAATGGGGTTGTGGGTCTAACAAGAGAGCTTCAAGTTCCATCTTC
AAGCTCTGGTTCTCTCATTTACAATAACCCCATTTCATTCTTTGATCAAGAATCTAAAAAG
ACTGTTTCTTTTTCCACAAGATTTGCTTTTTCAGTCAATAACATCAATCCATCTTCATTTGG
AGATGGTTTGGCCTTTTTCCTTTCACCTGATAATCAGACTTTAGGCAGTCCAGGTGGTTT
CTTGGGTCTGGTTAATTCATCACAATTAACCAAGAACAAGTTTGTTGCTGTTGAGTTTGAC
ACTAGGCAAGATTTACACTTTAATGATCCTGATGATAACCATGTAGGTCTTGATATTGATA
GCCTTATCTCAATCAAGACTGCTAATGTAAAGTTGGCTGGTGTTGATCTCAAAAGTGGGA
ATTTGATTACTTCTTGGATTGATTACAAGAGTGAGGAGAAGCAGTTGTTAGTGTTCTTGA
GTTACTCAAGTTCCAAGTCTAAAAAACCACTCTTGACTGTGGATATTGACCTGTCTCATTA
TCTAAAGGAGTTTATGTATGTGGGGTTTGCTGCTTCTACTGAGGGAAGCACTGAATTGCA
TTGTATTGAGAATTGGAGTTTTCAAACTTTTGGATTTAGTCCTATGAGGCCAAGGCATAAT
CCCCATGATGTGTCTGATAATTCTGTGTTAGTGAAACCCCCTACTATTCAGGAAGATTCT
GACAATAAACATCATAAGAGGTTAGGTTTGGGTCTTGGAATTGCTGGTCCAGCTTTCTTT
TGTGCTGTTCTTGTAGCTTTTGGTTGGATTTCTTTACAGAAATGGAGGGGCCTTAACACA
GAGAAGAATTTGACAGCTGAGCTTGTTACTGGACCAAGGCAGTTTAGCTACAAGGAACT
GAGGTCAGCTACAAGAGGATTTCATTCAAGTAGGATCATAGGCAACGGCGCTTTTGGTA
CTGTTTACAAGGCCTTTTTCATGGATTCGAGCTCTATCGCTGCAGTGAAAAGATCTAAGC
ACAGCCACGAAAGTAAAACTGAGTTCGGAGCTGAACTGTCGATCATAGCATGTTTAAGG
CACAAGAATTTAGTTCAGTTGCAAGGGTGGTGCATTGAGAAGGGAGAATTACTTCTTGTC

TATGACTTTATGCCAAATGGGAGTCTTGATAAGGTACTATACCAAGAATCTGAGAATGGG
AATCCACTGAAATGGCCTTATAGGTATAATATAGCAGTTGGTTTGGCCTCTGTGCTGACT
TATTTGCATCAAGAATGTGAGCAGCAAGTGATTCACAGAGATATTAAAGCAAGCAATATT
ATGCTTGATGTCAGCTACAACGCGAGGCTTGGCGATTTTGGGTTGGCAAGACTTATGGA
TCATGACAAGAGTCCAGTCTCGACTCTTACAGCTGGAACAATGGGATACCTTGCTCCTGA
GTACCTTCAATATGGAAAGGCAACCGAGAGAACTGATGTTTTCAGCTATGGCGTGGTTAT
ACTAGAAGTGGCTTGTGGGAGGAGGCCAATAGAGGGAGAAGGGAGTGGTCATGAAATG
GTTAATTTGGTTGATTGGGTTTGGAGGCTTTACTCTGAAGGTAGGATAATTGAAGCAGCA
GACAAAAGGCTAAATGGTGACTTCAAAGAGGAAGAGATGAGAAAGCTTCTACTTGTCGG
TTTAAGCTGTGCAAATCCTGATAGTATGGAAAGACCTTGTATGAGAAGAGTATTTCAGAT
CCTCAACAATGAAGCTGAACCTATCTTTGTTCCGAAAGTGAAACCGATGCTAACTTTCTC
CACTAGTCTGCCTTTCAGTATTAATGACATTTTCTCAGACGACGAAGACAATGAGGCAGC
ACCAGAACCTGAGTTTGAAATCAGAGTAGATTGA

>NbS00020348g0007.1
MSKLFSCSIFFSFLYCALLVSSQNCSFDLQSFTLRNFTLLGDSYLRNGVVGLTRELQVPSSSS
GSLIYNNPISFFDPESKKIVSFSTRFAFSVNNINPNPVSFFDPESKKIVSFSTRFAFSVNNINPS
SFGDGLAFFLSPDNQTLGSPGGFLGLVNSSQLTKNKFVAVEFDTWQDLHFNDPDDNHVGLD
IDSLISIKTANVKLAGVDLKSRNLITSWIDYKSEEKQLFVFLSYSSSKPKKPLLTVDIDLSDYLKE
FMYVGFAASTEGSTEMHCIENWSFQTFGFSPMRPRHNPHNVSDNSVLVKPPTIQEDSDNKH
NKRLGLGLGIAGPAFFCAVLVAFGWISFKKWRGLHTVKNLTAELVTGPRQFSYKELRSATRG
FHSSRIIGNGAFGTVYKAFFMDSSSIAAVKRSKHSHESKTEFGAELSIIACLRHKNLVQLQGW
CIEKGELLLVYDFMPNGSLDKVLYQESENGNPLKWPYRYNIAVGLASVLTYLHQECEQQVIH
RDIKASNIMLDVSYNARLEMRKLLLVGLSCANPDSMERPCMRRVFQILNNEAELIFVPKVKPT
LTFSTSLPFSINDIFSDDEDNEAAPEPEFEIRVD

>NbS00020348g0007.1 Corrected; GenBank ID: KT225338
MSKLFSCSIFFSFLYCALLVSAQNCSFDLESFTLRNFTLLGDSYLRNGVVGLTRELQVPSSSS
GSLIYNNPVSFFDPESKKIVSFSTRFAFSVNNINPSSFGDGLAFFLSPDNQTLGSPGGFLGLV
NSSQLTKNKFVAVEFDTWQDLHFNDPDDNHVGLDIDSLISIKTANVKLAGVDLKSRNLITSWI
DYKSEEKQLFVFLSYSSSKPKKPLLTVDIDLSDYLKEFMYVGFAASTEGSTEMHCIENWSFQT
FGFSPMRPRHNPHNVSDNSVLVKPPTIQEDSDNKHNKRLGLGLGIAGPAFFCAVLVAFGWIS
LQKWRGLNTEKNLTAELVTGPRQFSYKELRSATRGFHSSRIIGNGAFGTVYKAFFMDSSSIA
AVKRSKHSHESKTEFGAELSIIACLRHKNLVQLQGWCIEKGELLLVYDFMPNGSLDKVLYQES
ENGNPLKWPYRYNIAVGLASVLTYLHQECEQQVIHRDIKASNIMLDVSYNARLGDFGLARLM
DHDKSPVSTLTAGTMGYLAPEYLQYGKATERTDVFSYGVVILEVACGRRPIEGEGSGHEMV
NLVDWVWRLYSEGRIIEAADKRLNGDFKEEEMRKLLLVGLSCANPDSMERPCMRRVFQILN
NEAELIFVPKVKPTLTFSTSLPFSINDIFSDDEDNEAAPEPEFEIRVD

>NbS00020348g0007.1 Corrected; GenBank ID: KT225338
ATGAGTAAGCTTTTTTCTTGCTCCATTTTCTTCAGCTTCTTGTACTGTGCACTATTGGTAT
CAGCACAAAATTGCAGCTTTGATTTGGAATCTTTTACACTGAGAAATTTCACACTTCTTGG
AGATTCTTATCTCAGAAATGGGGTTGTGGGTCTAACAAGAGAGCTTCAAGTTCCATCTTC
AAGCTCTGGTTCTCTCATTTACAACAACCCCGTTTCATTCTTTGATCCAGAATCTAAAAAG
ATAGTTTCTTTTTCCACAAGATTTGCGTTTTCAGTCAATAACATCAATCCATCTTCATTTGG
AGATGGTTTGGCTTTTTTCCTTTCACCTGATAATCAGACTTTAGGTAGTCCAGGTGGTTTC
TTGGGTCTGGTTAATTCATCCCAATTGACTAAGAACAAGTTTGTTGCTGTTGAGTTCGAC
ACTTGGCAAGATTTGCACTTTAATGATCCTGATGATAACCATGTAGGTCTTGATATAGATA
GCCTTATCTCAATCAAGACTGCTAATGTAAAGTTGGCTGGTGTTGATCTCAAAAGTAGGA
ATTTGATTACTTCTTGGATTGATTACAAGAGTGAGGAAAAGCAGTTGTTTGTGTTCTTGAG
TTACTCAAGTTCCAAGCCTAAAAAACCACTCTTGACTGTGGATATTGACCTGTCTGATTAT
CTAAAGGAGTTTATGTACGTGGGGTTTGCTGCTTCTACTGAGGGAAGCACTGAAATGCAT
TGTATTGAGAATTGGAGTTTTCAAACTTTTGGATTTAGTCCTATGAGGCCAAGGCATAATC
CCCATAATGTGTCTGATAATTCTGTGTTAGTGAAACCCCCTACTATTCAGGAAGATTCTGA
CAATAAACATAATAAGAGGTTAGGTTTGGGTCTTGGAATTGCTGGTCCAGCTTTCTTTTGT
GCTGTTCTTGTAGCTTTTGGTTGGATTTCTTTACAGAAATGGAGGGGCCTTAACACAGAG
AAGAATTTGACAGCTGAGCTTGTTACTGGACCAAGGCAGTTTAGCTACAAGGAACTGAG
GTCAGCTACAAGAGGATTTCATTCAAGTAGGATCATAGGCAACGGCGCTTTTGGTACTGT
TTACAAGGCCTTTTTCATGGATTCGAGCTCTATCGCTGCAGTGAAAAGATCTAAGCACAG
CCACGAAAGTAAAACTGAGTTCGGAGCTGAACTGTCGATCATAGCATGTTTAAGGCACAA

GAATTTAGTTCAGTTGCAAGGGTGGTGCATTGAGAAGGGAGAATTACTTCTTGTCTATGA
CTTTATGCCTAATGGGAGTCTTGATAAGGTACTATACCAAGAATCTGAGAATGGGAATCC
ACTGAAATGGCCTTATAGATATAATATAGCAGTTGGTTTGGCCTCTGTTCTGACTTATTTG
CATCAAGAATGTGAGCAGCAAGTGATTCACAGAGATATTAAAGCAAGCAATATTATGCTT
GATGTCAGCTACAACGCGAGGCTTGGCGATTTTGGGTTGGCAAGACTTATGGATCATGA
CAAGAGTCCAGTCTCGACTCTTACAGCTGGAACAATGGGATACCTTGCTCCTGAGTACC
TTCAATATGGAAAGGCAACCGAGAGAACTGATGTTTTCAGCTATGGCGTGGTTATACTAG
AAGTGGCTTGTGGGAGGAGGCCAATAGAGGGAGAAGGGAGTGGTCATGAAATGGTTAA
TTTGGTTGATTGGGTTTGGAGGCTTTACTCTGAAGGTAGGATAATTGAAGCAGCAGACAA
AAGGCTAAATGGTGACTTCAAAGAGGAAGAGATGAGAAAGCTGCTACTTGTCGGTTTAA
GCTGTGCAAATCCTGATAGTATGGAAAGACCTTGTATGAGAAGAGTATTTCAGATCCTCA
ACAATGAAGCTGAGCTTATCTTTGTTCCAAAAGTGAAACCGACGCTAACTTTCTCCACTA
GTCTGCCTTTCAGTATTAATGACATTTTCTCAGATGACGAAGACAATGAGGCAGCACCAG
AACCTGAGTTTGAAATCAGAGTAGATTGA

>NbS00029224g0003.1 GenBank ID: KT225339
MDYYYWYFFLFAVLVNLVASAPTGPVINVTKHISFQDFSSKNPRLKQDLTLLGSAIVSDEKST
VQIPDPEQEGDLKHLAGRAIYSSPIRFFDPLSQTPASFETTFSFQFQVLKSNSSNWTSDLGES
VGGSGLTFIIVPDELTVGRSGPWLGMLNDLCDDDYKSVAIEFDTRQNPEFGDPNDNHLGINL
GSIVSTAAINASDVGVQLNDGSIHRVWISYDGQRRFIDIRLAPDGRGFPSKSVYSGFLDLSPY
LNEYMFVGFSAATGNLAQIHNVLSWNFTSVSQASLRIPSTETCQNKIMLQNNTQAETAHRKT
PNSFFIFLAVVILLVIVLVNLYFSSYKRDDNSSETIILPEKKQRPRPPNKARRFTIAEISVATRNF
SELQILGSDEKSVTYKATMLNGCNVVVKRFLTQFFNTHGFDKRRFHKEIKAISSIRHPNLVPV
RGWCYDNRETIVVYDYIPNGSLDKWLFGVGVLPWTRRFKVVKDLADSLVYLHSKQLAHKNV
KSSSAFLDVSFRAVMGDFGFVLTSAGSTRFESMVSQTADVFEFGVVVLETVAGRSRKSNPG
ERDLLDLAWAMHEVQQKETLVDRRMGAVVNLEQAIRVLDIGLLCTLNENKGRPTIEEVVEFL
NMEKPIPELPPGRPVCLFPYNSTTGLCSGYACTAF

>NbS00029224g0003.1 GenBank ID: KT225339
ATGGATTACTATTACTGGTACTTTTTCCTGTTTGCCGTACTTGTTAATTTAGTTGCATCAG
CTCCAACCGGACCTGTAATTAACGTGACTAAGCATATTTCGTTCCAAGATTTCAGTTCAAA
AAATCCAAGATTGAAGCAAGATCTTACACTTCTTGGCAGTGCTATCGTCTCAGATGAAAA
ATCAACAGTTCAAATTCCTGACCCTGAACAGGAAGGTGATCTTAAGCATTTAGCAGGGCG
AGCTATATACTCTTCACCTATTCGTTTCTTCGATCCTCTGTCTCAAACACCAGCTTCATTT
GAAACAACCTTCTCATTTCAGTTCCAAGTATTGAAGTCCAATTCGAGCAACTGGACATCA
GATCTTGGCGAATCTGTTGGTGGTAGCGGCCTTACTTTTATCATAGTCCCTGATGAATTG
ACAGTTGGCCGCTCTGGTCCGTGGCTTGGCATGCTCAACGATCTATGCGACGACGATTA
TAAGTCAGTAGCTATTGAGTTTGATACACGGCAGAATCCTGAATTTGGTGATCCAAATGA
CAATCACTTGGGCATCAATTTAGGAAGCATAGTTTCAACTGCAGCAATAAATGCTTCTGA
CGTTGGAGTCCAACTGAATGACGGATCAATTCACAGGGTTTGGATATCTTACGATGGGC
AGAGGCGATTTATCGACATCCGTCTTGCACCTGATGGCAGAGGATTTCCTTCTAAATCAG
TCTACTCTGGTTTTCTTGACCTCTCGCCTTACTTAAATGAGTACATGTTCGTAGGATTTTC
AGCTGCAACTGGTAACCTTGCACAAATTCACAATGTCCTGTCCTGGAATTTCACTTCAGT
TAGTCAAGCTTCCCTTAGAATTCCTTCAACAGAGACATGTCAGAATAAAATCATGCTTCAA
AATAATACCCAAGCTGAAACTGCACATCGGAAAACGCCTAATAGTTTCTTTATTTTCCTTG
CTGTTGTCATTCTTCTAGTAATTGTTCTTGTTAATCTCTATTTTAGTAGCTACAAGCGAGAT
GACAATTCCAGTGAAACAATCATTCTGCCCGAGAAAAAGCAAAGACCAAGGCCACCAAA
TAAAGCACGTCGCTTTACAATAGCCGAAATCTCAGTCGCAACAAGGAATTTCAGTGAGTT
ACAAATATTGGGCAGTGATGAGAAGAGCGTTACTTATAAGGCTACGATGCTGAACGGGT
GCAATGTTGTTGTAAAACGATTTTTAACTCAGTTTTTCAATACCCATGGGTTTGACAAGCG
AAGATTTCACAAGGAAATTAAGGCCATCAGCAGTATTCGCCACCCGAATTTGGTCCCCGT
TAGAGGATGGTGCTATGACAACCGAGAAACTATAGTTGTTTACGATTACATCCCAAACGG
TAGCCTAGACAAATGGTTGTTTGGTGTCGGTGTATTACCTTGGACGAGGCGTTTTAAGGT
TGTTAAAGATTTAGCAGACTCTCTTGTTTACCTACACTCGAAGCAACTTGCTCACAAGAAC
GTGAAAAGTAGCAGTGCGTTTCTTGACGTGAGCTTCAGAGCAGTGATGGGTGATTTTGG
ATTTGTGCTCACTTCGGCTGGGTCAACTCGGTTTGAGTCCATGGTGAGTCAAACAGCAG
ACGTGTTCGAGTTTGGAGTTGTTGTGCTAGAAACTGTTGCGGGTCGGAGCAGGAAGTCC
AACCCGGGAGAACGTGACTTGTTGGATCTTGCATGGGCCATGCATGAGGTACAACAGAA
GGAAACGTTAGTGGATCGAAGAATGGGCGCGGTTGTAAACCTGGAGCAGGCGATTCGG
GTATTGGATATCGGGTTGCTATGTACATTGAACGAGAACAAAGGAAGACCTACAATAGAA

GAAGTGGTAGAGTTCCTGAATATGGAGAAACCAATTCCTGAGCTGCCACCGGGTCGACC
CGTTTGTTTGTTCCCGTATAACAGCACCACAGGTCTGTGCAGTGGGTACGCCTGCACTG
CATTCTAA

>NbS00001395g0006.1 GenBank ID: KT225340
MDYYYWFFFLFAVLINLVAAAPTGPVINVTKHIAFQDFSSKNPRLKQDLTLLGSAIVSDEKSTV
QIPDPEQEGDLKHLAGRAIYASPIRFFDPLSQTPASFETTFSFQFQVLKSNSSNGTSDLGKYV
GGSGFTFIIVPDELTLGRSGPWLGMLNDLCDDDYKSVAIEFDTRRNTEFGDPNDNHLGINLG
SIVSTAAINASDVGVQLNDGSIHRVWISYNGQRRFIDIRLAPDGRGYPSKSVYSGFLDLSPYL
NEYMFVGFSAATGNHTQIHNVLSWNFTSVCQASLRIPSTETCQNQIMLQNNTQAETTHRKTP
NSFFIFLAVVILLVIVLINLYFSSYKRNDNSSETIILPEKKQRPRPPNKARRFTIAEISVATRNFSE
LQILGSDEKSITYKATMLNGCNVVVKRFLTQFFNTHGFDKQRFHKEIKAISSIRYPNLVPLRGW
CYDNQETIVVYDYIPNGSLDKWLFGVGVLPWTRRFKVVKDLADSLVYLHSKQLAHKNVKSSS
VFLDVSFRAVVGDFGFVLTSAGSTRFESMVSQTADVFEFGVVVLETVAGRSRKSNPGERDL
LDLAWAMHEVQQKETLVDRRMGAVVNLEQAIRVLDIGLLCTLNENKGRPTMEEVVEFLNME
NPIPELPSGRPVCLFPYNSTTGLCSGYACTAFK

>NbS00001395g0006.1 GenBank ID: KT225340
ATGGATTACTATTACTGGTTCTTTTTCCTGTTTGCCGTACTTATTAATTTAGTTGCAGCAG
CTCCAACAGGACCTGTTATTAACGTGACTAAGCATATTGCTTTCCAAGATTTTAGTTCCAA
AAATCCCAGATTGAAGCAAGATCTTACACTACTTGGCAGTGCTATCGTCTCAGATGAAAA
ATCAACAGTTCAAATTCCTGACCCTGAACAGGAAGGTGACCTTAAGCATTTAGCAGGACG
AGCTATTTATGCTTCACCTATCCGTTTCTTTGATCCTCTATCTCAAACACCAGCTTCATTT
GAAACAACCTTCTCATTTCAATTTCAAGTACTGAAGTCCAATTCCAGCAACGGGACATCA
GATCTTGGAAAATATGTTGGTGGTAGTGGCTTTACTTTTATTATTGTCCCTGATGAATTAA
CCCTTGGCCGTTCTGGTCCGTGGCTTGGCATGCTCAACGATCTATGCGACGACGATTAC
AAATCAGTAGCTATTGAGTTTGATACGCGCCGGAATACTGAATTTGGTGATCCAAATGAC
AATCACTTGGGCATCAATTTAGGAAGCATAGTTTCAACTGCAGCAATTAATGCTTCGGAC
GTTGGAGTCCAACTCAATGACGGCTCAATTCACAGAGTTTGGATATCATACAATGGGCAG
AGGCGATTTATCGACATCCGTCTTGCACCTGATGGCAGAGGATATCCTTCTAAGTCAGTC
TACTCTGGTTTTCTTGACCTCTCGCCTTACTTGAATGAGTACATGTTTGTAGGATTTTCAG
CTGCAACTGGTAACCATACGCAAATCCACAATGTCCTGTCCTGGAATTTCACTTCAGTTT
GTCAAGCTTCCCTTAGAATTCCTTCAACAGAGACATGTCAAAATCAAATCATGCTTCAAAA
TAATACACAAGCTGAAACTACACATCGGAAAACGCCTAATAGTTTCTTTATTTTCCTTGCT
GTTGTCATTCTTCTAGTAATTGTTCTTATTAATCTCTATTTTAGTAGCTACAAGCGAAATGA
CAATTCCAGTGAAACAATCATTCTGCCCGAGAAAAAGCAAAGACCAAGGCCACCAAATAA
AGCACGTCGCTTTACAATAGCCGAAATCTCAGTNCAATCATTCTGCCCGAGAAAAAGCAA
AGACCAAGGCCACCAAATAAAGCACGTCGCTTTACAATAGCCGAAATCTCAGTAGCAACT
AGGAATTTCAGTGAGTTACAAATATTGGGCAGTGACGAGAAGAGCATTACGTATAAGGC
CACGATGCTGAACGGGTGTAACGTGGTTGTAAAACGATTTTTAACTCAGTTTTTCAACAC
TCATGGATTTGACAAGCAAAGATTTCACAAGGAAATTAAGGCTATTAGCAGTATTCGCTA
CCCAAATTTGGTCCCCCTTAGAGGATGGTGCTATGACAACCAAGAAACTATAGTTGTTTA
CGATTACATCCCAAACGGTAGCCTAGACAAATGGTTGTTTGGTGTCGGCGTCTTACCTTG
GACGAGGCGTTTTAAGGTTGTTAAAGATTTAGCGGACTCTCTTGTCTACCTTCACTCAAA
GCAACTTGCTCACAAGAACGTGAAAAGTAGTAGTGTGTTTCTTGACGTGAGCTTCAGAGC
AGTAGTGGGTGATTTTGGATTTGTGCTTACTTCAGCTGGGTCAACTCGGTTTGAGTCCAT
GGTGAGTCAAACAGCAGACGTGTTCGAGTTTGGAGTTGTTGTCCTAGAAACTGTTGCGG
GTCGGAGCAGGAAGTCTAACCCGGGAGAACGTGACTTGTTGGATCTTGCATGGGCCAT
GCATGAGGTACAACAGAAGGAAACGTTAGTGGATCGAAGAATGGGCGCGGTTGTGAAC
CTGGAGCAGGCGATTCGGGTATTGGATATCGGGTTGCTATGTACGTTGAACGAGAACAA
AGGAAGGCCTACAATGGAAGAAGTGGTGGAGTTCCTGAATATGGAGAATCCAATTCCTG
AGTTGCCATCGGGTCGACCCGTTTGTTTGTTCCCCTACAACAGCACCACAGGTTTGTGC
AGTGGGTACGCCTGCACTGCATTCAAATGA

S. lycopersicum LecRK sequences

>Solyc09g012000.1.1 GenBank ID: KT232153
MLPMLLNFVILVSLLFAPTSGEVDGFIYNGFKPSDVSLDGIANITPNGLLLLTDSKTQDQGHAF
YPNPIHFKNSPYGDAFSFSTTFVFAIRSDYRPLDGHGLVFVIAPQRGIRKALANHYLGLFNSR
NNGDKSNHVVGVEFDTIYSADFGDMNDNHVGIDINGLRSVANHTAGYFDDHTGLFNNFTLVS
GMAMQVWVDYDGITKLINVTVAPLHMEKPVTPLLSLKYDLSPILNQIMYVGFSSSTGSVPTHH
YILGWSFKANGKAQELSQLPSLPRLGRKGASRFVTIGLPVISLVTIVVATLVVVCYVRRKKYEE
VLEDWEREYRPQRFNYKDLYTATKGFREKELLGAGGFGKVYKGVMPVTKHEIAVKKISHES
RQGMKEFVSEIVSIGRMQHRNVVPLLGYCRRKGELLLVYEFMSNGSLDKYLYDQPRLTLDW
NQRFRVIQGVASGLFFLHEECDRVVVHRDIKASNVLLDGELNGRLGDFGLARLYGHGTDPQ
STRVVGTLGYLAPEHTRTGRATPSSDVFSFGAFLLEVACGRRPIEPRHDGDDLILVDWVFSC
WNRGNILEAVDPNLGNDFVPGQVELVLSLGLFCSHSEPSFRPTMRQNVLFLEGVVALPELSA
LGVSYADLTFDHRGGFDDFIKSYPASSVNAHSPASSVAESLLSDGR

>Solyc09g012000.1.1 GenBank ID: KT232153
ATGTTACCAATGTTGTTAAATTTTGTCATACTAGTGTCTCTCTTGTTTGCACCAACTTCTG
GTGAAGTTGATGGATTCATTTACAATGGATTCAAACCAAGTGATGTTAGTTTAGATGGCAT
TGCTAATATTACACCCAATGGTCTTTTGTTGTTAACAGATTCCAAAACACAAGATCAAGGC
CATGCTTTTTATCCAAATCCAATTCATTTCAAGAATTCACCTTATGGTGATGCATTTTCATT
CTCTACAACCTTTGTGTTCGCGATAAGGTCTGATTATAGACCATTGGATGGTCATGGACT
AGTTTTTGTTATCGCGCCACAGAGAGGAATTCGAAAGGCTTTGGCAAACCACTATCTTGG
TCTTTTTAACTCAAGAAATAATGGGGATAAATCAAACCATGTTGTTGGAGTTGAGTTTGAT
ACAATATATAGCGCGGATTTTGGTGATATGAATGACAATCATGTTGGAATTGATATAAATG
GATTAAGGTCTGTAGCAAATCACACAGCAGGTTATTTTGATGATCATACTGGTTTGTTTAA
TAACTTTACTCTGGTTAGTGGTATGGCAATGCAAGTTTGGGTGGATTATGATGGTATAAC
TAAGCTAATCAATGTGACAGTAGCTCCATTACATATGGAAAAACCAGTTACTCCACTTTTG
TCTTTGAAATATGATCTTTCGCCGATTCTTAATCAGATTATGTATGTTGGTTTTTCATCATC
AACTGGTTCAGTCCCAACACATCATTATATCTTAGGATGGAGCTTTAAGGCAAATGGGAA
AGCTCAAGAACTTTCTCAACTTCCTAGTCTTCCTCGTCTTGGGCGTAAAGGGGCATCAAG
ATTTGTAACGATTGGGTTACCAGTGATCTCTTTGGTTACAATTGTTGTAGCAACCTTGGTG
GTGGTTTGTTATGTTAGAAGGAAGAAGTATGAAGAAGTTCTTGAAGATTGGGAACGTGAG
TATAGACCGCAAAGGTTCAATTATAAGGATTTGTACACTGCTACTAAAGGGTTCAGAGAA
AAGGAGCTGTTGGGAGCTGGTGGATTTGGTAAAGTTTATAAAGGAGTGATGCCTGTAAC
CAAACATGAGATAGCTGTAAAGAAGATATCTCATGAATCAAGACAAGGGATGAAGGAATT
TGTTTCGGAGATTGTAAGTATAGGCCGTATGCAACATAGGAATGTAGTACCACTTTTGGG
CTATTGTAGGCGAAAAGGGGAATTACTTTTGGTTTATGAGTTCATGTCTAATGGAAGTCT
AGATAAGTATTTATACGACCAACCAAGACTCACCCTCGATTGGAACCAAAGATTCAGAGT
CATTCAAGGTGTAGCATCAGGACTATTTTTCCTACATGAAGAATGTGACCGTGTAGTGGT
TCATAGGGATATTAAGGCTAGTAATGTGTTGTTGGATGGTGAACTAAATGGCAGGCTAGG
AGACTTTGGACTCGCGAGGCTATATGGTCATGGGACTGATCCTCAATCAACTCGTGTCG
TTGGTACTCTTGGTTATCTTGCACCAGAGCATACTAGAACTGGCAGGGCAACACCTAGTA
GTGATGTATTTTCCTTTGGGGCTTTTCTGCTTGAAGTTGCGTGTGGTAGGAGGCCGATAG
AGCCAAGACATGACGGTGATGATCTGATTTTAGTTGATTGGGTGTTTTCGTGCTGGAATA
GAGGTAATATTCTCGAGGCTGTTGATCCAAACTTAGGCAATGATTTTGTTCCAGGGCAAG
TTGAGTTGGTCTTAAGTCTAGGCTTGTTCTGTTCTCATTCAGAGCCCTCATTTAGGCCAA
CCATGCGACAAAACGTGTTGTTCTTGGAAGGTGTGGTGGCCTTACCAGAGTTATCAGCT
CTCGGTGTTTCATATGCTGACCTAACATTTGACCATCGCGGAGGTTTTGATGATTTTATCA
AGTCGTATCCAGCTTCTTCTGTTAATGCACATTCCCCCGCTTCATCTGTAGCAGAATCGC
TCCTTTCTGATGGCCGATGA

>Solyc09g011990.1.1 GenBank ID: KT232154
MLFNLVILVSLFLVGIAPVSCEVDHAFIYNGFQSQNLSLDGIAEFTPDGLLLLTDSETQDQGHA
FYPNPIHFKNSTNGTVVSFSTTFVFAIRSDYGILSGHGLVFVIAPQRRIQGALAYHYLGLFNST
NNGDRSNRVVGVELDTIYSEEFGDINANHVGIDINGLRSVAVETAGYFDNTGLFHNLTLISGQ
PMQVWVDYDGSTKQINVTVAPLHVEKPVSPLLSLKTDLSPILDQTMYVGFSSSTGSVPTYHYI
LGWSFKTNGKAQELSQLPNLPRLGRKGTSRFVKIGLPLLSLVLLVVATSMVVYYVRRKKYEE
LLEDWEREYRPQRFMYKDLYTATKGFREKELLGAGGFGKVYKGVMPITNLEIAVKKISHESR
QGMKEFVSEIVIIGRLQHRNVVPLLGYCRRKGELLLVYEYMSGGSLDKYLYNKPRFTLDWSQ
RFRVIRGVASGLFFLHEECDHVVVHRDIKASNVLLDGELNGRLGDFGLARLYSHGTDPQSTR

VVGTLGYLAPEHTRTGRSTPSSDVFSFGAFLLEVACGRRPIEPRQDDDDLILVDWVFSCWN
RGNILEAVDLNVGIDFVPGEVEMVLKLGLLCSHSKPSYRPTMRQVLLFLDGVVALPELSSLGI
SAAGLTFDHHGGFDDFVTSYSSSINAHSRSPSVTDSFLSGGR

>Solyc09g011990.1.1 GenBank ID: KT232154
ATGTTGTTCAATCTTGTCATACTAGTTTCTCTTTTCTTAGTTGGTATTGCACCAGTTTCTTG
TGAAGTTGATCATGCATTCATTTACAATGGATTCCAATCACAAAATCTTAGCTTGGATGGT
ATAGCAGAGTTTACGCCCGATGGTCTTTTGCTGTTAACAGATTCTGAAACTCAAGATCAG
GGCCATGCTTTCTATCCGAATCCAATTCATTTCAAGAATTCAACTAATGGTACTGTAGTTT
CATTCTCTACAACATTTGTGTTCGCCATAAGGTCTGATTATGGAATTTTGAGTGGTCATGG
ACTGGTTTTTGTTATCGCGCCACAGAGAAGAATTCAAGGAGCTTTAGCATACCACTATCT
AGGACTTTTTAACTCAACTAATAATGGGGATAGATCAAACCGTGTAGTTGGAGTTGAGCT
CGATACAATCTATAGCGAGGAATTTGGTGATATCAATGCCAATCATGTTGGAATTGATATA
AATGGATTAAGGTCTGTAGCAGTTGAAACAGCAGGTTATTTTGATAATACTGGCTTGTTTC
ATAATTTGACTTTGATAAGTGGCCAGCCTATGCAAGTTTGGGTGGATTATGATGGCAGTA
CTAAGCAAATTAATGTAACAGTAGCTCCATTACATGTGGAAAAACCAGTTAGTCCACTTTT
GTCTTTGAAAACTGATCTTTCGCCGATTCTTGATCAGACCATGTATGTTGGTTTTTCATCA
TCAACTGGTTCTGTCCCAACATATCATTATATCTTGGGATGGAGCTTCAAGACAAATGGG
AAGGCTCAAGAACTTTCTCAACTTCCTAATCTTCCTCGTCTTGGACGCAAAGGGACATCA
AGATTTGTAAAGATTGGTTTGCCATTACTCTCTTTGGTTTTACTTGTTGTAGCAACCTCGA
TGGTGGTTTACTATGTTAGAAGGAAGAAGTATGAAGAACTTCTTGAAGATTGGGAACGCG
AGTACAGACCACAAAGGTTTATGTACAAAGATTTATACACTGCCACTAAAGGATTCAGAG
AAAAGGAGCTGTTGGGAGCTGGTGGATTTGGTAAAGTTTACAAAGGAGTAATGCCTATC
ACCAATCTTGAGATAGCTGTAAAGAAGATATCTCATGAATCAAGACAAGGGATGAAGGAA
TTTGTTTCGGAGATTGTGATCATAGGTCGTCTACAACACAGGAATGTAGTACCACTTTTG
GGTTATTGCAGGCGGAAAGGAGAGTTGCTTTTGGTTTATGAATACATGTCTGGTGGAAGT
CTAGACAAATATTTATACAACAAACCAAGATTCACCCTTGATTGGAGCCAAAGATTCAGA
GTTATTAGAGGTGTAGCATCAGGACTATTTTTCCTACACGAAGAATGTGACCATGTAGTG
GTTCATAGGGACATTAAGGCTAGCAATGTCTTGTTGGATGGTGAACTAAATGGTAGGCTA
GGAGACTTTGGACTTGCGAGGCTATATAGTCATGGTACCGATCCTCAGTCTACTCGTGT
CGTTGGTACACTTGGTTACCTTGCACCAGAGCATACTAGAACTGGCAGGTCAACACCTA
GTAGTGATGTATTTTCCTTTGGTGCTTTTCTGCTTGAAGTTGCCTGTGGTAGGAGGCCTA
TAGAGCCAAGACAAGACGATGATGATCTGATTTTAGTCGATTGGGTTTTCTCATGCTGGA
ATAGGGGTAATATTCTTGAGGCTGTTGATCTAAACGTAGGGATCGATTTTGTTCCAGGGG
AAGTGGAGATGGTCTTAAAGCTAGGCTTGCTCTGCTCTCATTCAAAGCCCTCGTATAGGC
CAACCATGCGACAAGTCTTGTTGTTCTTAGATGGTGTTGTGGCCTTACCAGAGTTATCAT
CACTCGGTATTTCAGCAGCCGGCCTAACATTTGATCATCACGGAGGTTTTGATGATTTTG
TCACGTCCTATTCATCTTCGATTAATGCACATTCCCGTTCTCCATCTGTAACAGACTCCTT
TCTCTCTGGTGGGCGATGA

>Solyc09g011060.2.1 GenBank ID: KT232155
MFFNLFILVALFLLDFSPASCEVDAFIFNGFQSANLSLDGIAQFKSNDLLLLTNSGTQNQGHAF
YPNPIHFKNSSNGTVFSFSTTFVFAIRSDYGNLSGHGLAFVIAPHKGLQGSLANHYLGLFNSS
NNGNTSNHVVGVELDTIYSEDFGDINDNHVGIDINGLRSVAIHTAGYFDDTDLFHNLTLISGQE
MQVWIDYDGRTKQMDVTVAQSHMEKPVRPLLALKYDLSSILDQTMYVGFSSSTGSVPTHHY
ILGWSFKTNGKAQELSQLPKLPRLGTKGTSRFVTIGLPIISLVSLVAAVLAVVYYVRKKKYEEIH
EDWEREYRLQRFKYKELYIATKGFREKELLGAGGFGKVYKGVMPTTKLEIAVKKISHESRQG
MKEFVSEIVSIGRMQHRNVVPLLGYCRRKGELILIYEYMSNGSLDKYLYDQPRYTLDWNQRF
RVIRGVASGLFFLHEECDHVVVHRDVKASNVLLDGELNGRLGDFGLARLYGHGTDPQSTRV
VGTLGYLAPEHTRTGRATPSSDVFSFGAFLLEVVCGRRPIQPRQDGDDLILVDWVFSCWNR
GNILDAADPNIGIDFVPGQVELVLKLGLFCSHSEPSCRPTMRQILLFLDGVVALPELSELGVSS
ANLTFEHRGGFDDFVKSYPSSLGYAYSGSPSVTDSFLSGGNVGDHIALLKVYNSWKETNFS
TQWCYDNYIQVRSMKRARDIRDQLEGLLERVEIKLTSNVNDLEAIKKCITSGFFPHTARLQKA
GSYRTVKHPQTVNIHPSSGLSQELPRWVVYHEQVLTSKEYMQQVTELKPEWLVEIAPHYYQ
LKDVEDSSSKKMPRGIGHAP

>Solyc09g011060.2.1 GenBank ID: KT232155
ATGTTCTTCAATCTTTTCATACTAGTAGCTCTCTTCTTACTTGATTTTTCACCTGCTTCTTG
TGAAGTTGATGCATTCATTTTCAATGGATTTCAATCAGCTAATCTTAGCTTGGATGGCATA
GCACAGTTCAAATCTAATGATCTTTTGTTGTTAACAAATTCTGGAACACAAAATCAGGGCC

ATGCTTTTTATCCAAATCCAATTCATTTCAAGAATTCATCAAATGGTACTGTATTTTCTTTC
TCCACAACTTTTGTGTTCGCGATAAGGTCTGATTATGGAAATTTGAGTGGTCATGGACTA
GCTTTCGTCATCGCGCCACACAAAGGACTTCAGGGGTCTTTGGCAAATCACTATCTTGGT
CTTTTTAACTCTAGTAATAATGGGAATACATCAAACCATGTCGTTGGAGTTGAGCTCGATA
CAATCTATAGCGAGGATTTTGGTGATATCAATGACAATCATGTTGGAATTGATATAAATGG
ATTGAGGTCTGTAGCAATTCACACAGCAGGTTATTTTGATGATACTGATTTGTTTCATAAC
TTGACTTTAATTAGTGGCCAGGAAATGCAAGTTTGGATCGATTACGATGGGAGGACTAAG
CAAATGGATGTAACAGTAGCTCAATCACATATGGAAAAACCAGTTAGACCACTCTTGGCT
TTGAAATATGATCTTTCGTCGATTCTTGATCAGACTATGTATGTTGGTTTTTCATCGTCAA
CAGGTTCAGTCCCAACGCATCATTATATCTTGGGATGGAGCTTCAAGACAAATGGGAAA
GCGCAAGAACTCTCGCAACTTCCTAAGCTTCCTCGTCTTGGGACTAAAGGGACATCAAG
ATTTGTAACGATTGGTTTGCCTATAATCTCATTGGTTTCACTTGTTGCAGCTGTCTTAGCG
GTGGTTTATTATGTAAGAAAGAAGAAGTATGAAGAAATTCATGAAGATTGGGAACGCGAG
TATAGACTACAAAGGTTTAAGTATAAAGAATTGTACATTGCTACTAAGGGATTCAGAGAAA
AAGAGCTATTGGGAGCTGGAGGATTTGGTAAAGTTTATAAAGGAGTGATGCCTACCACC
AAACTTGAGATAGCTGTAAAGAAGATATCTCATGAATCGAGACAAGGGATGAAGGAATTT
GTTTCGGAGATTGTAAGCATTGGTCGTATGCAACACAGGAATGTAGTACCACTTTTGGGT
TATTGCAGGCGAAAAGGGGAGTTGATTTTGATTTACGAATACATGTCAAATGGAAGTCTA
GACAAGTATTTATACGATCAGCCAAGATACACCCTCGATTGGAACCAAAGATTCAGAGTC
ATTAGAGGTGTAGCTTCGGGACTATTTTTCCTACACGAAGAATGTGATCACGTAGTGGTT
CATAGAGATGTTAAGGCCAGTAATGTCTTGTTAGATGGTGAACTAAATGGCAGGTTAGGA
GATTTTGGACTCGCTAGGCTGTATGGTCATGGGACCGATCCTCAGTCCACACGGGTTGT
TGGTACTCTTGGTTACCTTGCACCAGAGCATACTAGAACTGGCAGGGCAACACCTAGTA
GTGATGTTTTTTCATTTGGGGCATTTCTGCTTGAAGTTGTCTGTGGTAGGAGGCCGATAC
AGCCAAGACAAGACGGTGATGATCTGATTTTGGTCGATTGGGTGTTCTCGTGTTGGAATA
GGGGTAATATTCTTGATGCTGCTGATCCGAACATAGGCATTGATTTTGTTCCAGGGCAAG
TGGAGTTGGTCTTAAAGTTAGGCTTGTTCTGTTCTCATTCAGAGCCCTCGTGTAGGCCAA
CCATGCGACAAATCTTGTTGTTTTTGGATGGTGTTGTGGCCTTACCAGAGTTATCAGAAC
TCGGTGTTTCATCAGCTAACCTAACATTTGAACATCGCGGAGGTTTTGATGATTTTGTCAA
GTCGTATCCATCTTCTTTGGGCTATGCATATTCCGGTTCTCCATCTGTAACAGACTCCTTT
CTCTCTGGTGGCCGGTGATTACTAATGTATTGAACCTGGTAAACATAATCTTGTTTTGGT
GGATTATGTACTGATAAATTAGATTTAATCTACACATTATAGAACAGTTTCATAACATTAAA
ATGATTCAGGCTTTGATAGTACAGTTTACAATGGAACAATACACAGAGTTAAGTGATTTCC
TGTACTTATTTTTCTTTCATTTGTTATGTTTTCACCTATCTAATCAATAACCGTTTATGACTG
ATCACTCGCCATTAACTCGGTTTTTGTGTCATAATCATTATATAGAAGAGATGGCCGAGT
GATTTAAGGCGTAGCATTGGTTAATAGGAATCAAGTTATTCATTGGAATCAATTCATTGTG
ATGAAGGGCTCTATATTTGCCTTCCTCAGATGTTGAATGTTTAATGAGTTCATGACTTTAT
ATGTTGACAATGACAAACTGCAAACCACAAAGCACTCTTTGACTGTTATCACCTAGTTTTC
TATCTCATTCCGATGGGGGATTTGCCTAAGCTATCTTACATATCCCTCTTCTTCAGAAGCT
TGTCAGTCTACTAGTCCTACTTTTTTACTAGCCAAGTACGTTGAAATTCTTTTTGATATGG
GTGGCAGTGTGATTAGGTCTCAGGACCTCTGTCTGCTCTGATATCATGTTGAAGTGTATG
ACAATCTTAATTATCTAAAAGTTTAAGTTGTTAGAGAGAACACATTTTTATTAATTAATTGT
ATTCTCAGCTGACATCGCCCTAACCACAGTGCTGTTATATACATCCACACACATGAAGTG
CACCTATATGCAAAATTTCTACTGTTAATTAGAGGTTTTAATGATTCATTGTAAAATCATTT
TGCAAGAGGACCCTGAAAAGAAGTGGCAAAAATATCAAATTTAGATGCCAAAGCTTAGTA
ATATTTTGTTGAATTTGCTGTGAAGGGAAATATTTTGGTAGGAAAATTTTGGTGGTATAGA
ACCCGCTACATTGAATTTTGACTTCATGGACCCTCCACCATCAGAAGCTTTACTTAAAGC
ACTCGAACTTCTCTTTGCCCTCGGTGCCCTTAATAAGCTTGGAGAGTTAACAACAATAGG
TAGAAGAATGGCAGAGTTTCCTCTAGATCCCATGCTGTCCAAGACGATCGTTGCCTCTGA
CAAATACAAGTGTTCTGATGAAATTATAAGTATTGCTGCAATGCTTTCTGTGGGGAATTCA
ATCTTTTATCGTCCAAAGGACAAACAAGTCCACGCTGACAATGCACGTTTGAATTTTCATA
CAGGCAACGTTGGAGATCATATCGCACTGTTAAAGGTTTATAATTCCTGGAAGGAGACAA
ACTTCTCAACACAGTGGTGCTACGACAATTACATCCAGGTCCGAAGCATGAAGAGAGCT
AGGGATATCCGAGATCAATTGGAAGGCCTGCTGGAGAGAGTAGAAATTAAGTTAACTTC
AAATGTTAATGACTTAGAAGCTATAAAGAAGTGTATAACATCAGGATTTTTCCCACACACG
GCGAGACTACAAAAGGCTGGATCTTATCGAACTGTTAAACATCCTCAAACAGTTAACATT
CACCCCAGCTCAGGTTTATCTCAGGAGCTTCCTAGATGGGTTGTGTACCATGAGCAGGT
CTTGACGAGTAAGGAGTATATGCAACAGGTTACTGAACTGAAACCAGAATGGCTTGTAGA
AATAGCTCCTCACTACTATCAGTTGAAAGATGTTGAAGACTCTAGCTCAAAGAAAATGCC
TCGCGGAATTGGACATGCACCTTAG

>Solyc09g005000.1.1 GenBank ID: KT232156
MDNPYCLVIILALIFCPILVRAQSFDFAYNGFNNSNIIRDGVAIINSSGALKLTNRSYNVIGHAFH
PNPVPIFNSSTKNVTSFSTYFVFAIVPLEKTSGGFGFAFTLSPSPGFPGAQGDHFLGVVNIMN
DGNDTNHIFMVEFDTVNGHNEGVDTDGNHIGVNRNGMSPTASKSADYYVNDSAVAEQVNL
QSGESIQAWIDYDGMSKVVNVTISPITVPKPGRPLISEVVDLSTVLNETMYAGFSAATGDKAS
SHYILGWSFRLNGAADPLELVKLPVAPPEVVTSSKNSHLKRALIGTFCSVVILLLGAFYIYTRM
RQHEVLEDWELDCPHRFRYRHLYKATKGFQESELIGVGGFGAVYKGVLPTNGAEVAVKKIS
SNSLQGMREFAAEIESLGRLRHKHLINLQGWCKNKNDLLLVYDYVPNGSLDSLLYRQKRDIV
LTWEQRFNIIKGIAAGLLYLHEEWEQVVIHRDVKSSNVLIDGEMNGRLGDFGLARLYDHGKN
SHTTNVVGTIGYIAPELTRTGKASTSTDVYAYGVLLLEVASGRPPIVYEPGKGALVLADWVIEC
LQLGNILDAVDQRLNSVYVDKEVQMIFRLGLVCSHPRPEARPTMRQVMKYLNGDESLPVFE
QQLSCIGSDRVDEITAKFLEVFSTDTINISRRSLSVGQISSSSLHSGR

>Solyc09g005000.1.1 GenBank ID: KT232156
ATGGACAATCCCTATTGCCTAGTTATTATTCTTGCATTGATCTTTTGCCCTATTCTTGTGA
GAGCTCAATCTTTTGATTTTGCTTACAATGGATTCAATAACTCAAATATCATTAGAGACGG
AGTAGCAATAATCAATTCAAGTGGTGCACTTAAGCTCACTAATAGATCATATAATGTTATA
GGTCATGCTTTCCACCCCAATCCTGTACCAATTTTCAACTCCAGTACTAAAAATGTTACTT
CATTTAGTACATACTTCGTCTTCGCCATCGTCCCTCTTGAGAAAACCTCTGGTGGCTTTG
GCTTTGCTTTCACCTTATCTCCATCCCCGGGTTTTCCGGGTGCTCAGGGTGATCATTTTC
TTGGAGTCGTCAACATTATGAACGATGGCAATGATACTAACCACATCTTCATGGTTGAAT
TTGACACTGTGAATGGTCACAATGAAGGTGTAGACACAGATGGAAATCACATTGGGGTC
AACAGAAATGGCATGAGCCCTACAGCGTCAAAATCAGCTGACTACTATGTTAATGATTCT
GCTGTAGCGGAGCAGGTAAATCTACAAAGTGGAGAATCAATTCAAGCTTGGATTGATTAT
GATGGAATGAGCAAAGTGGTGAATGTAACTATATCTCCCATAACAGTTCCAAAGCCAGGT
AGGCCTCTGATCAGTGAGGTCGTAGATTTATCTACTGTGTTGAACGAAACTATGTATGCT
GGTTTCTCAGCAGCCACGGGAGACAAAGCGAGCTCTCATTACATCTTGGGTTGGAGTTT
TCGGTTGAATGGAGCTGCTGATCCTTTAGAGCTTGTAAAGCTACCTGTTGCCCCACCTGA
AGTGGTAACATCATCCAAGAATTCCCATCTCAAAAGGGCCTTGATCGGAACATTTTGCTC
CGTCGTTATCCTGCTCCTTGGGGCATTTTACATCTATACAAGGATGAGGCAACATGAAGT
TCTAGAGGACTGGGAGCTGGATTGTCCTCACAGGTTCCGATATAGACATCTTTACAAGG
CAACTAAGGGATTCCAGGAGAGTGAACTAATTGGAGTTGGTGGATTTGGTGCTGTTTACA
AGGGTGTTTTGCCTACTAATGGAGCAGAAGTTGCAGTGAAGAAGATATCGAGCAATTCTC
TTCAAGGAATGAGAGAATTTGCAGCGGAGATCGAAAGCTTAGGCAGGTTAAGGCACAAA
CACTTGATCAACCTTCAAGGCTGGTGCAAGAACAAAAATGATCTTCTCCTAGTGTATGAC
TATGTCCCAAATGGAAGTCTTGATTCATTACTTTACAGACAGAAGAGGGACATTGTTTTAA
CATGGGAACAGAGATTTAACATCATCAAAGGGATTGCTGCAGGGCTTCTTTACTTGCATG
AAGAATGGGAACAAGTGGTAATACATCGCGATGTGAAAAGCAGTAACGTCCTTATTGATG
GGGAAATGAATGGCCGGTTAGGGGATTTTGGGTTAGCAAGATTATACGACCATGGCAAG
AATTCACACACACAACAAATGTTGTTGGCACAATAGGGTACATTGCACCAGAATTGACACGG
ACAGGGAAGGCCTCAACGAGCACGGATGTGTATGCATATGGTGTACTACTTCTTGAAGT
AGCTAGTGGAAGACCTCCTATTGTCTATGAACCAGGGAAAGGAGCTTTAGTACTAGCTG
ATTGGGTGATTGAATGTTTACAACTAGGTAATATTCTTGATGCAGTTGATCAAAGGTTGAA
TTCAGTGTATGTAGATAAAGAGGTTCAAATGATTTTCAGACTTGGGCTTGTTTGTTCTCAC
CCAAGGCCAGAAGCTAGGCCAACAATGAGACAAGTAATGAAGTACCTCAATGGAGATGA
ATCACTTCCAGTTTTTGAGCAACAGTTGAGTTGTATTGGATCTGATAGGGTTGATGAAAT
CACAGCCAAGTTCTTGGAAGTGTTTTCTACTGATACAATCAATATATCACGTCGTTCGCTA
TCCGTCGGACAGATATCATCTAGTTCACTCCATTCTGGTCGATGA

>Solyc02g078170.1.1
MNQFYPKILLSYLLLALFSCNQSVAETDFVFNGFKQSDVSIFGNVTIESRILTLSNDSTFSIGRA
LYPSKIVTKEANSSKVLPFSTSFIFAMAPYRDRLPGHGIVFLFVPHTGIDGTTSAQNLGFLNFT
NNGNPDNHVFGVEFDVFKNQEFNDINENHVGIDVNSLASEFAHEAGYWPDEKIKYNSDGSL
NEESLETLKLNNGRNYQVWIDYVDFNISVTMAPVGMKRPKQPLLDFHLNLSQVFEEEMYVG
FTAATGELAQSHKILAWSFSNSNFSIGDGLITQGLPSFELPEDPVYRSKGFIAGMTVSLLFLVV
VIAVASWFLIKRNRRMKKEREDMEDWELEYWPHRITYQEIDAATKGFADENVIGIGGNGKVY
KGVLAGGSEVAVKRISHQNSEGARQFLAEISSLGRLKQRNLVSLRGWCKKDRGSMFVVYDY
MENGSLDKRLFESNDRNMLSFEDRIRILKDVASGVLYLHEGWEAKVLHRDIKASNVLLDRDM
NARLGDFGLARMHDHSQVASTTRVVGTVGYLAPEFAKTGRASTQTDVFGYGVLIMEVMCG

RRPIEEEGKPPLVDWLWELMSRGELINAFDSRLRTNQDFNEEEALRVLQLGMICASLDAKAR
PSMRQVVKFFDRNNDIDEYETEDMDAYLLESLRSNTMLSNFSLSLSHGSHPTFDEIREGASF
D

>Solyc02g078170.1.1 Corrected; GenBank ID: KT232157
MNQFYPKILLSYLLLALFSCNQSVAETDFVFNGFKQSDVSIFGNVTIESRILTLSNDSTFSIGRA
LYPSKIVTKEANSSKVLPFSTSFIFAMAPYRDRLPGHGIVFLFVPHTGIDGTTSAQNLGFLNFT
NNGNPDNHVFGVEFDVFKNQEFNDINENHVGIDVNSLASEFAHEAGYWPDEKIKYNSDGSL
NEESLETLKLNNGRNYQVWIDYVDFNISVTMAPVGMKRPKQPLLDFHLNLSQVFEEEMYVG
FTAATGELAQSHKILAWSFSNSNFSIGDGLITQGLPSFELPEDPVYRSKGFIAGMTVSLLFLVV
VIAVASWFLIKRNRRMKKEREDMEDWELEYWPHRITYQEIDAATKGFADENVIGIGGNGKVY
KGVLAGGSEVAVKRISHQNSEGARQFLAEISSLGRLKQRNLVSLRGWCKKDRGSMFVVYDY
MENGSLDKRLFESNDRNMLSFEDRIRILKDVASGVLYLHEGWEAKVLHRDIKASNVLLDRDM
NARLGDFGLARMHDHSQVASTTRVVGTVGYLAPEFAKTGRASTQTDVFGYGVLIMEVMCG
RRPIEEEGKPPLVDWLWELMSRGELINAFDSRLRTNQDFNEEEALRVLQLGMICASLDAKAR
PSMRQVVKFFDRNNDIDEYETEDMDAYLLESLRSNTMLSNFSLSLSHGSHPTFDEIREGLSS
SMSISWTNSLVDGR

>Solyc02g078170.1.1 Corrected; GenBank ID: KT232157
ATGAACCAATTTTATCCAAAAATTCTTCTTTCTTATCTGTTATTAGCCCTATTTTCTTGTAA
CCAATCAGTTGCAGAAACTGATTTTGTCTTCAATGGCTTCAAACAATCAGATGTGTCAATA
TTTGGGAATGTTACAATTGAATCAAGAATTCTTACTCTTTCAAATGACTCAACTTTTTCAAT
TGGTAGAGCTCTGTACCCTTCAAAGATTGTTACAAAAGAAGCTAATTCATCTAAAGTTCTT
CCCTTTTCAACATCTTTCATTTTTGCAATGGCTCCTTACAGAGACAGGCTACCGGGACAT
GGTATAGTTTTCTTGTTTGTGCCACACACAGGTATTGATGGTACTACTTCTGCACAGAATT
TAGGTTTCTTGAATTTTACAAATAATGGGAATCCTGATAATCATGTGTTTGGGGTTGAGTT
TGATGTTTTCAAGAATCAAGAGTTTAATGATATAAATGAGAATCATGTTGGAATTGATGTT
AATTCTCTTGCATCTGAGTTTGCTCATGAAGCAGGATATTGGCCTGATGAGAAAATTAAG
TATAATAGTGATGGTAGCCTAAATGAGGAGTCTTTAGAGACTTTGAAGTTGAACAATGGA
AGAAATTATCAAGTTTGGATTGATTATGTTGATTTCAACATTAGTGTAACTATGGCACCAG
TTGGTATGAAAAGGCCTAAGCAGCCTTTGTTGGATTTTCACCTAAATCTTTCCCAGGTTTT
TGAGGAAGAGATGTATGTGGGGTTTACAGCAGCCACTGGAGAGCTTGCTCAAAGTCACA
AGATTTTAGCTTGGAGCTTTAGTAACTCGAATTTTTCGATAGGTGATGGTTTGATCACACA
AGGGCTGCCTTCATTTGAGCTTCCTGAGGATCCAGTTTATCGATCGAAGGGATTCATTGC
AGGTATGACAGTGTCACTCTTGTTTCTTGTTGTGGTCATTGCTGTAGCTTCATGGTTTTTG
ATTAAGAGAAACAGGAGAATGAAAAAGGAAAGAGAGGATATGGAAGATTGGGAATTGGA
ATATTGGCCACATAGGATTACTTATCAAGAAATTGATGCTGCAACAAAGGGTTTTGCTGA
TGAAAATGTGATTGGAATTGGAGGTAATGGGAAGGTTTATAAAGGGGTTTTGGCTGGGG
GTTCGGAGGTTGCAGTAAAGCGCATTTCTCACCAAAACAGTGAAGGAGCAAGGCAATTC
TTGGCTGAGATTTCGAGTCTTGGTAGGCTAAAGCAAAGAAATTTGGTGTCATTAAGAGGG
TGGTGCAAGAAAGATAGAGGCAGTATGTTTGTGGTTTATGATTATATGGAAAATGGGAGC
TTGGATAAAAGACTGTTTGAATCTAATGACAGAAACATGCTTAGTTTTGAAGACAGAATTA
GGATTTTGAAAGACGTGGCATCAGGGGTGCTATACTTGCACGAGGGATGGGAGGCAAA
AGTGTTGCACAGGGACATTAAGGCTAGCAATGTTTTACTTGACAGGGACATGAATGCAA
GGCTAGGTGATTTTGGTCTAGCTAGAATGCATGATCATAGTCAAGTGGCTAGCACGACT
CGAGTTGTTGGCACGGTGGGATACTTGGCACCAGAGTTTGCCAAGACTGGCCGTGCCT
CCACACAAACCGATGTGTTTGGATATGGAGTACTAATTATGGAGGTGATGTGTGGAAGG
AGGCCTATAGAGGAGGAAGGCAAGCCGCCTTTAGTGGATTGGCTGTGGGAACTAATGA
GCCGAGGCGAATTGATTAACGCCTTTGATAGTCGATTAAGGACTAACCAAGATTTCAATG
AAGAGGAAGCTTTAAGGGTGTTGCAATTAGGCATGATATGCGCGAGCCTAGACGCCAAA
GCTAGGCCAAGCATGAGACAAGTGGTGAAATTCTTTGACAGGAATAATGATATTGATGAA
TATGAAACGGAGGACATGGATGCTTACCTTCTAGAGAGTTTGAGATCTAATACCATGTTG
TCCAATTTTTCGTTGAGTTTAAGCCATGGTTCACATCCAACATTTGATGAAATTAGAGAAG
GTTTGTCTTCTTCCATGTCAATTTCTTGGACAAATTCACTGGTGGATGGTAGATGA

>Solyc10g084250.1.1 GenBank ID: KT232158
MTSNLEKKIRDFLVWFILLLCFCSISSTFGKTDFDFENLTLSSLKLLGDAHLSNNIVKLTRDLAV
PNSGAGKVLYSKPVRFQQPGFDFPASFSTFFTFSVTNLNPSSIGGGLAFVLTPGDELVGDAG
GYMGILDAKGTQIGTIAVEFDTLMDVEFKDINGNHVGLDLNSMISTEVGNLDSIDIDLKSGDLV
NCWIDYSGSNGEMDIFVSYTNLKPKESFLSVNINLAEYVNDFMFVGFSASTQGSTEIHNIVW

WSFSSSFDATPNSGAVVAEPPPPANSLMNPTADSVPLPPPSMAPSESNSSKGVLQEKSSRK
CHSNFCRQGPGAVVGVVTAGAFFLAFATLVLIWLYSKRFKRVKNSEIVASDIIKMPKEFSYKE
LKLATKGFIPTRIIGRGAFGTVYKGILSDTGGIVAVKRCSHNGQGKAEFLSELSIIGTLRHRNLV
RLQGWCHEKGEILLVYDLMPNGSLDKVLFESRMVLSWSHRRKILIGVASALAYLHQECENQV
IHRDIKSSNIMLDEGFNARLGDFGLARQVEHDKSPDATVAAGTMGYLAPEYLLTGRATEKTD
VFSYGAVVLEVATGRRPIERETTKVEKVRVNSNLVEWVWGVHREGNLLNAADSRLCGEFDE
QEMRRVLMIGLACSHPDPTARPTMRSVVQMLEGESDIPIVPRTRPSMSFSTSHLLMSLQDSV
SDLNGLITLSPSSSESSFTGGHNGNCNGMELV

>Solyc10g084250.1.1 GenBank ID: KT232158
ATGACTTCAAATCTTGAAAAAAAAATCAGGGACTTTCTCGTATGGTTCATCTTATTGCTCT
GTTTCTGTAGTATTAGTTCAACATTTGGTAAAACTGATTTCGATTTTGAAAATTTAACATTG
AGTAGTTTAAAGCTTCTTGGTGATGCACACTTGAGTAACAACATTGTAAAGTTGACACGT
GATCTTGCTGTACCAAATTCCGGTGCCGGAAAAGTTCTGTATTCAAAACCAGTAAGATTC
CAGCAACCGGGGTTTGATTTTCCGGCGAGTTTCTCGACATTCTTTACATTTTCAGTTACTA
ATTTGAACCCATCGTCGATTGGTGGCGGTCTTGCTTTTGTGCTTACGCCGGGTGATGAG
TTAGTAGGTGATGCTGGTGGGTATATGGGAATCTTGGATGCTAAAGGGACACAAATTGG
TACAATTGCTGTGGAATTTGACACCCTTATGGATGTTGAATTCAAAGATATTAATGGAAAT
CATGTTGGTTTGGATCTAAATTCAATGATTTCAACTGAAGTTGGTAATTTGGATTCCATTG
ATATTGATCTCAAGAGTGGTGATTTGGTTAATTGTTGGATTGACTATTCTGGTTCTAATGG
AGAGATGGATATATTTGTGTCATATACTAATCTCAAGCCAAAAGAATCATTTTTATCAGTT
AATATCAATCTTGCTGAGTATGTAAATGATTTCATGTTTGTGGGGTTTTCTGCTTCAACTC
AAGGGAGTACTGAGATTCATAATATTGTGTGGTGGAGTTTCAGTTCATCATTTGATGCAA
CTCCTAATTCGGGGGCGGTGGTGGCTGAGCCACCACCGCCAGCGAATAGTTTGATGAA
CCCAACTGCAGATTCTGTCCCGTTGCCACCGCCTTCTATGGCTCCATCAGAGTCTAATAG
TAGCAAAGGTGTATTGCAAGAAAAGAGTAGTAGGAAATGTCATAGCAATTTTTGTAGACA
AGGACCTGGAGCTGTTGTTGGTGTGGTTACTGCTGGTGCATTTTTTCTTGCATTTGCTAC
ATTGGTACTTATTTGGTTATACTCCAAAAGATTCAAGAGAGTGAAAAATTCTGAGATTGTG
GCATCTGATATCATCAAAATGCCTAAGGAGTTCAGCTATAAAGAGCTTAAATTGGCCACG
AAAGGTTTCATTCCAACGAGAATTATAGGCCGTGGTGCATTTGGGACTGTTTACAAGGGC
ATTTTATCGGATACCGGAGGCATTGTGGCAGTCAAGAGATGTAGTCATAATGGACAAGG
GAAAGCAGAGTTCTTATCTGAATTATCTATAATTGGAACCCTTAGGCACAGAAATCTTGTG
AGGCTTCAAGGATGGTGTCATGAGAAAGGTGAAATTTTGTTGGTTTATGATCTTATGCCT
AATGGGAGTCTTGATAAGGTGCTATTTGAATCAAGAATGGTTCTTTCATGGTCGCATAGG
CGAAAAATCTTGATAGGTGTTGCTTCTGCCTTGGCATATTTGCATCAAGAATGCGAAAAC
CAGGTGATTCATAGGGACATTAAGAGTAGTAACATTATGTTGGATGAAGGGTTCAATGCA
AGATTAGGAGATTTTGGATTAGCAAGACAAGTAGAGCATGACAAGTCCCCCGATGCAAC
GGTAGCAGCCGGGACAATGGGCTACTTGGCTCCAGAGTACTTGTTAACCGGTAGAGCAA
CCGAAAAAACTGATGTTTTCAGCTATGGAGCAGTGGTTCTTGAAGTGGCTACTGGAAGG
AGGCCAATTGAGAGGGAAACAACTAAAGTTGAGAAAGTTAGAGTGAATAGCAACTTGGTT
GAATGGGTATGGGGGGTGCATAGAGAAGGGAATTTACTAAATGCAGCTGATTCAAGACT
CTGTGGTGAGTTTGATGAACAAGAAATGAGAAGGGTTCTAATGATTGGGCTAGCTTGTTC
ACATCCCGACCCTACGGCTAGACCAACAATGAGAAGTGTAGTCCAAATGCTGGAAGGTG
AATCTGATATCCCAATTGTCCCAAGAACTAGACCTTCTATGAGTTTTAGCACATCACATCT
TCTAATGAGTTTGCAAGATAGTGTCTCTGACTTGAATGGGTTGATCACACTTTCCCCTTCA
TCATCCGAAAGTAGCTTCACCGGGGGTCACAATGGCAATTGCAATGGCATGGAGTTGGT
CTAA

>Solyc09g007510.1.1
MDVEFKDINGNHVGLDLNSMISTQVGDLDSIGVDLKSGDLVNSWIDYFGSTKKLNLYVSYSN
LKPKEPFLSVTIDISEYVNDFMFVGFSGSTQGSTEIHSIEYWSFTSSFDTNPKSPPAAVAEQP
PPPPTASLMNPTADVTTSVTSPPPEQQQIAPAEANGTANAVQKNSSKCHSSFCKQGAGAVV
GVVTAGAFFLALATLVLIWLYSKKFKHVKASETMLPSDIIKMPKEFSYKELKIATKGFDSTRIIG
HGAFGTVYKGILSETGDIVAVKRCSHNGQGKAEFFSELSIIGTLRHRNLVRLQGWCHEKGEIL
LVYDLMPNGSLDKALFESRMILSWQHRQKILLGVASALAYLHQECENQVIHRDIKSSNIMLDE
GFNAKLGDFGLARQIEHDKSPDATVAAGTMGYLAPEYLLTGRATEKTDVFSYGAVVLEVASG
RRPIDKETTTSGVGLNSNLVEWVWGLHKDGSLLSAVDSKLNLEYDEKEMTRVLLVGLACSH
PDPIARPTMRGVVQMLVGEAEVPIVPRAKPSMSFSTSHLLMTLQDSVSDLNGMITLSTSSSE
NSFNGGGVVGGHDGMDLV

>Solyc09g007510.1.1 Corrected; GenBank ID: KT232159
MEIYSLLVVLLCFFAALAAATEFDSGTLTLSSLKLLGDAPFLAVPNSGAGKVLYSKPVKFRQP
GIDFPASFSTFFSFSVSNLNPSSIGGGLAFVITPDDESIGDSGGYLGILDAEGGQNGNFGVEF
DTLMDVEFKDINGNHVGLDLNSMISTQVGDLDSIGVDLKSGDLVNSWIDYFGSTKKLNLYVSY
SNLKPKEPFLSVTIDISEYVNDFMFVGFSGSTQGSTEIHSIEYWSFTSSFDTNPKSPPAAVAE
QPPPPPTASLMNPTADVTTSVTSPPPEQQQIAPAEANGTANAVQKNSSKCHSSFCKQGAGA
VVGVVTAGAFFLALATLVLIWLYSKKFKHVKASETMLPSDIIKMPKEFSYKELKIATKGFDSTRII
GHGAFGTVYKGILSETGDIVAVKRCSHNGQGKAEFFSELSIIGTLRHRNLVRLQGWCHEKGEI
LLVYDLMPNGSLDKALFESRMILSWQHRQKILLGVASALAYLHQECENQVIHRDIKSSNIMLD
EGFNAKLGDFGLARQIEHDKSPDATVAAGTMGYLAPEYLLTGRATEKTDVFSYGAVVLEVAS
GRRPIDKETTTSGVGLNSNLVEWVWGLHKDGSLLSAVDSKLNLEYDEKEMTRVLLVGLACS
HPDPIARPTMRGVVQMLVGEAEVPIVPRAKPSMSFSTSHLLMTLQDSVSDLNGMITLSTSSS
ENSFNGGGVVGGHDGMDLV

>Solyc09g007510.1.1 GenBank ID: KT232159
ATGGAGATTTACTCATTACTAGTTGTATTGCTCTGTTTCTTTGCTGCTTTAGCTGCTGCCA
CTGAGTTTGATTCTGGTACTTTAACTCTTAGTAGTTTGAAGCTTTTAGGCGATGCCCCATT
CCTTGCTGTTCCAAATTCTGGTGCCGGAAAAGTTTTGTATTCTAAACCAGTAAAGTTCCG
GCAGCCGGGTATTGATTTTCCGGCGAGTTTCTCTACGTTTTTCTCGTTTTCTGTTAGTAAT
TTGAATCCTTCATCGATTGGAGGTGGGTTGGCTTTTGTTATCACGCCGGATGATGAATCG
ATTGGTGATTCTGGTGGCTATTTGGGGATTTTGGATGCAGAAGGAGGGCAAAATGGGAA
TTTTGGAGTTGAATTTGATACACTTATGGATGTTGAATTTAAAGATATTAATGGTAATCATG
TGGGGTTAGATTTAAATTCAATGATTTCAACTCAGGTTGGTGATCTTGATTCTATTGGTGT
TGATTTAAAAAGTGGTGATTTGGTTAATTCTTGGATTGATTATTTTGGTTCAACAAAAAAAT
TGAATCTTTATGTTTCATATTCTAATCTTAAGCCAAAAGAACCATTTTTGTCTGTTACAATT
GATATTTCTGAATATGTAAATGATTTCATGTTTGTGGGGTTTTCTGGTTCAACACAAGGGA
GTACTGAGATTCATAGTATTGAATATTGGAGTTTCACTTCATCATTTGATACAAATCCTAA
ATCTCCGCCTGCAGCGGTGGCGGAGCAGCCTCCGCCCCCGCCAACGGCTAGTTTGATG
AACCCCACGGCGGATGTAACTACATCCGTCACATCGCCCCCTCCGGAGCAGCAACAGAT
TGCTCCGGCGGAGGCTAATGGTACTGCTAATGCAGTACAGAAAAACAGCAGCAAGTGTC
ATAGTAGTTTTTGTAAGCAAGGTGCTGGTGCTGTTGTTGGGGTTGTTACTGCTGGAGCAT
TCTTTCTAGCTTTAGCTACATTGGTACTTATTTGGTTGTATTCGAAAAAATTCAAGCATGT
GAAGGCTTCAGAGACTATGTTGCCTTCTGATATTATCAAAATGCCTAAGGAATTTAGTTAT
AAGGAGCTTAAAATCGCGACGAAAGGGTTTGATTCGACTAGGATTATAGGGCATGGTGC
ATTTGGTACTGTCTATAAAGGGATTTTGTCCGAGACTGGTGATATTGTTGCGGTAAAGAG
GTGTAGTCATAATGGACAAGGGAAAGCTGAGTTCTTTTCTGAATTATCGATAATCGGAAC
ACTTAGACATAGAAATCTTGTTAGACTACAAGGATGGTGTCATGAGAAAGGTGAAATCTT
GTTAGTTTATGATTTGATGCCAAATGGTAGTCTTGACAAGGCGTTATTCGAGTCGAGAAT
GATCCTCTCGTGGCAACACAGGCAAAAAATCTTGTTAGGTGTTGCTTCTGCATTAGCATA
TTTACATCAAGAATGCGAAAACCAGGTGATACATAGGGATATCAAGAGTAGTAACATCAT
GTTGGATGAAGGGTTCAATGCAAAGTTAGGAGATTTTGGACTAGCACGACAAATCGAAC
ACGACAAGTCCCCCGATGCAACGGTAGCAGCCGGTACAATGGGGTATTTAGCACCAGAA
TACTTGTTAACAGGTAGAGCAACTGAGAAAACTGATGTGTTTAGTTATGGGGCAGTGGTT
CTTGAAGTGGCAAGTGGAAGGAGGCCTATTGATAAAGAAACAACAACAAGTGGTGTTGG
ATTAAACAGCAATTTGGTTGAATGGGTGTGGGGATTACACAAAGATGGGAGTTTACTATC
AGCAGTTGATTCAAAATTGAATCTTGAGTATGATGAAAAAGAAATGACAAGAGTGTTGTTA
GTTGGTTTGGCTTGTTCACATCCTGACCCTATAGCTAGGCCAACAATGAGGGGGGTGGT
ACAAATGTTAGTAGGTGAGGCTGAAGTACCAATTGTACCAAGGGCTAAACCATCAATGAG
TTTTAGCACATCTCATTTGTTAATGACATTGCAAGATAGTGTTTCTGACTTGAATGGTATG
ATCACACTTTCAACTTCTTCATCAGAAAACAGTTTCAATGGTGGTGGTGTTGTTGGTGGA
CATGATGGAATGGACCTAGTTTAG

>Solyc03g043710.1.1 GenBank ID: KT232160
MSSNVCCIALFIASLLIPFVNSVSFQISRFGPDVIDILYEGDAVASVGEVEFNKANYLCRVAHAI
YREKVPLWDPDHDSTKLADFSTRFSFTIDTQNRSSYGHGIAFFLAPVGFRIPPNSDGGFLGLF
NTTTSDSAQSQIVSVEFDTFSNVEWDPPFEHVGINNNSIASSVTAPWNVSLHPIETWITYNAT
TNNLSVIWNYGTGPNSSMFYIINLRDVLPPWVTIGFSAATGLNVERHTLESWEFSSSLVITEL
GGNDREKIGLIAGLTTLGGILLVSAILALIVLRKRRRKVKGNPETISLTSFNDDLEKGAGPRKFS
YKELDTSTNHFSEERKLGEGGFGEVYKGYLIDLDIAVAVKKISRGSKQGKKEYITEVKVISRLR
HRNLVQLIGWCHDQGEFLLVYEFMPNGSLDFHLFGKRNPLSWTMRYKISLGLASALLYLHEE

WEQCVIHRDIKSSNIMLDSSFNVKLGDFGLARLMDHELGPQTTGLAGTLGYLAPEYIKTGRAS
KESDVYSFGIVALEIATGRKSVDPGTGNCDAGLVEYVWDFYEKGKLLTVVDEKLNMDFEREQ
VERLIIIALWCAHPESNLRPSIKQAIHVLNFEASLPNLPMKMPVPVYYSPRPCGEPTVSSGEPT
MTYTSIDVGR

>Solyc03g043710.1.1 GenBank ID: KT232160
ATGTCTTCCAATGTATGTTGCATAGCTCTCTTCATTGCCTCCTTGCTAATTCCATTTGTGA
ATTCAGTGTCCTTTCAAATATCTCGATTTGGACCGGACGTGATTGACATACTCTACGAAG
GAGATGCAGTCGCGTCTGTAGGAGAGGTCGAGTTTAACAAAGCGAACTATCTATGTCGC
GTTGCTCATGCCATCTATAGAGAGAAAGTTCCACTATGGGATCCTGATCATGACTCCACA
AAGCTAGCTGATTTCTCTACACGTTTTTCGTTCACTATAGACACTCAAAATCGTTCCTCAT
ATGGTCATGGTATCGCGTTTTTCCTAGCTCCAGTTGGTTTTCGAATTCCACCTAACTCAG
ATGGTGGCTTTCTTGGACTGTTTAATACAACCACTAGTGATTCAGCTCAAAGCCAAATTG
TTTCTGTTGAGTTTGATACATTTTCAAACGTCGAATGGGATCCTCCATTCGAGCACGTTG
GTATCAACAACAATTCAATTGCTTCATCTGTAACAGCTCCTTGGAATGTTAGTTTACATAG
TGGTAATCCTATTGAGACATGGATTACTTACAATGCTACAACAAATAATCTTAGTGTCATT
TGGAACTATGGAACAGGTCCGAATTCGAGCATGTTTTACATAATAAACCTCAGAGATGTT
CTGCCTCCGTGGGTCACAATTGGATTCTCTGCTGCAACAGGACTAAATGTCGAGAGACA
TACACTTGAATCGTGGGAATTCAGCTCGAGTCTTGTTATAACAGAGTTAGGTGGAAATGA
TCGCGAAAAGATTGGACTTATTGCAGGGCTAACAACATTAGGTGGGATTTTGTTAGTGAG
CGCGATTTTGGCTTTGATAGTTTTGAGGAAACGTAGACGAAAGGTGAAGGGAAATCCAG
AGACAATAAGCTTAACATCTTTCAATGATGATCTTGAAAAGGGGGCAGGACCAAGAAAGT
TTTCGTATAAAGAGCTGGATACTTCAACTAATCATTTCTCAGAGGAACGAAAATTAGGTGA
AGGAGGATTTGGAGAAGTTTACAAAGGGTACCTCATTGATCTTGACATAGCGGTTGCTGT
AAAGAAGATCTCGAGAGGGTCTAAGCAGGGGAAAAAGGAATACATAACCGAGGTGAAG
GTTATTAGTCGATTAAGGCATAGAAACCTTGTGCAACTAATCGGTTGGTGTCATGATCAA
GGTGAGTTCTTACTTGTTTACGAATTCATGCCAAATGGTAGCTTAGATTTTCATTTGTTTG
GTAAAAGGAATCCTCTTAGTTGGACTATGAGGTACAAGATTTCACTTGGCTTAGCATCCG
CGTTGCTCTATCTACACGAAGAATGGGAGCAATGTGTGATCCACAGAGACATAAAATCGA
GCAACATAATGCTCGATTCAAGTTTTAATGTCAAGCTTGGTGATTTTGGCTTAGCTAGACT
AATGGACCATGAGTTAGGTCCTCAGACTACAGGGCTAGCCGGAACTCTAGGTTATTTGG
CTCCGGAATACATAAAAACAGGACGAGCAAGCAAAGAGTCGGATGTATACAGCTTCGGA
ATAGTTGCATTAGAAATTGCAACAGGAAGAAAATCAGTTGATCCAGGGACAGGGAATTGT
GATGCAGGATTAGTAGAGTATGTTTGGGATTTTTATGAAAAAGGAAAACTTCTTACTGTTG
TTGATGAGAAATTAAACATGGATTTCGAACGAGAACAAGTAGAACGATTGATCATTATCG
CGTTGTGGTGTGCTCATCCCGAGAGCAATCTAAGGCCATCGATAAAACAAGCAATTCAT
GTTCTGAATTTCGAGGCATCGTTGCCTAATCTTCCAATGAAGATGCCTGTTCCTGTGTAC
TATTCACCTAGGCCATGTGGTGAACCAACAGTTAGCTCCGGGGAGCCTACGATGACTTA
CACAAGCATCGATGTGGGTCGATAA

>Solyc01g106160.1.1 GenBank ID: KT232161
MFLSKFLNILLVLVIFVIPSLYSLNFNITNIDTSHVNLSINVTGDAYVTKEGIQVTPNERNMTLGG
KTGRATYIETLQLWNKATRELTDFTTHFSFVIDSNGNGSFADGLAFFLAPVGSSIPVGSSGSG
LGLVKAETENMPSYESFVAIEFDTFVNTWDPFGIHVGININSMESVATKIWVNDIKLGKKNDA
WISYNASSKVLEVVFTGFQKKYNRDKLSYAVDLRDYLPENVSFGFSASTGQLFQKNNVKSW
DFNSSFDAIIVPEHVNQPSASPPIQLQDPKNHPIQGQTPNDPPITHVQEPKGPPQKVLRTSGK
GSKGLVVGSSIGLPILILGLITASCILWRKKRKGDEKENVFIDLDMDDEFEKGTGPKKFSYGEL
ARATNNFAEGQKLGEGGFGDVYKGLLKECNSYVAVKRVSKGSKQGIKEYASEVKIISRLRHR
NLVQLIGWCHEKEKLHLVYELMPNESLDKHLFKEKSLLVWEIRWKIAQAIASALLYLHEEWEQ
CVVHRDIKASNVMLDSNFNAKLGDFGLARLIDHDKGSQTTMLAGTVGYMAPECIMNGKASK
ESDVYSFGIVALEIASGRRSINIKAPEDQVRLVEWVWSLYGTGELVEATDPRLNKMFNEKEM
ERLMVIGLWCAHPDNKLRPSIRQAIHVLNSEAQLPNLPSRMPVATYSPPPLNMFSSPFSNTY
EVSMQEQTMTYTVSSSVYTSSAASSTKSLL

>Solyc01g106160.1.1 GenBank ID: KT232161
ATGTTTCTCAGCAAATTTTTGAACATCTTGCTAGTTTTAGTAATTTTTGTTATCCCTTCTTT
ATATTCATTGAACTTTAATATCACCAATATTGATACTTCTCATGTTAATCTCTCTATCAATG
TCACTGGAGACGCCTATGTAACGAAAGAGGGCATACAAGTCACGCCTAATGAACGTAAC
ATGACATTAGGTGGAAAAACAGGACGCGCCACATACATTGAGACACTGCAGTTATGGAA
CAAGGCTACAAGGGAATTGACAGATTTTACTACGCATTTCTCTTTTGTTATTGATTCAAAT

GGTAACGGTAGTTTTGCTGATGGACTAGCCTTTTTCTTAGCTCCTGTTGGTTCGAGTATT
CCAGTTGGTTCGTCTGGCAGTGGCCTTGGTCTTGTTAAAGCTGAGACAGAAAACATGCC
ATCATATGAGTCATTTGTTGCTATAGAGTTCGATACATTTGTTAATACCTGGGACCCTTTT
GGCATACATGTAGGCATCAATATAAATTCTATGGAATCTGTTGCTACTAAAATATGGGTAA
ATGACATTAAACTTGGGAAGAAAAATGATGCTTGGATTAGTTACAATGCTAGTTCCAAGG
TTCTTGAAGTTGTTTTCACAGGATTTCAGAAAAAATATAATCGAGACAAACTAAGCTACGC
CGTTGATCTGAGGGATTATTTGCCTGAGAATGTTAGTTTTGGCTTCTCAGCATCAACTGG
ACAATTGTTTCAGAAAAACAATGTCAAGTCTTGGGATTTCAATTCGAGTTTTGATGCTATC
ATAGTTCCAGAACATGTCAATCAGCCTAGTGCAAGTCCTCCTATACAACTTCAAGATCCA
AAAAATCATCCTATTCAAGGTCAAACTCCAAATGATCCTCCAATTACTCATGTTCAAGAAC
CAAAGGGTCCTCCTCAAAAAGTACTCAGGACCTCGGGAAAAGGAAGCAAGGGACTAGTA
GTTGGATCAAGCATAGGATTACCTATTCTGATTCTTGGTTTGATAACTGCCAGTTGCATTT
TGTGGAGGAAAAAGAGAAAAGGAGATGAGAAAGAAAATGTCTTCATTGATCTAGATATGG
ATGATGAATTTGAAAAGGGTACCGGTCCTAAGAAGTTCTCGTATGGTGAATTAGCTCGTG
CAACAAACAACTTTGCTGAGGGACAGAAGCTTGGTGAAGGAGGATTTGGTGATGTTTATA
AAGGTTTATTGAAGGAATGTAACTCATATGTAGCTGTTAAGAGAGTATCAAAAGGGTCTA
AACAAGGGATAAAGGAGTATGCATCAGAAGTGAAGATCATCAGTCGATTAAGGCATAGA
AATTTGGTTCAACTTATCGGTTGGTGCCACGAGAAAGAGAAGCTGCATCTTGTTTACGAA
CTGATGCCCAATGAGAGCCTAGATAAACATCTTTTCAAAGAAAAGTCATTGTTGGTCTGG
GAAATCAGGTGGAAAATTGCTCAAGCAATTGCCTCAGCTTTACTGTATCTACACGAAGAG
TGGGAACAATGCGTAGTCCATAGAGACATAAAAGCAAGTAATGTCATGTTGGATTCAAAC
TTCAACGCTAAATTAGGTGATTTTGGGTTGGCTAGGCTTATTGACCATGACAAAGGATCA
CAAACAACGATGCTAGCAGGGACCGTGGGGTATATGGCACCGGAATGTATTATGAATGG
AAAAGCTAGCAAGGAGTCAGATGTATACAGCTTTGGAATAGTAGCATTGGAAATAGCAAG
TGGAAGAAGATCAATAAATATTAAAGCACCAGAAGATCAAGTGAGATTGGTTGAATGGGT
GTGGAGCCTGTATGGAACGGGAGAGCTAGTCGAAGCAACTGATCCAAGATTGAATAAAA
TGTTTAATGAGAAAGAAATGGAACGTTTAATGGTCATCGGTCTATGGTGTGCTCATCCAG
ACAACAAACTGAGGCCATCAATAAGGCAAGCTATCCATGTTCTAAACTCTGAAGCTCAAT
TACCAAATCTTCCATCAAGAATGCCAGTTGCAACATATTCACCCCCTCCATTGAATATGTT
TTCCTCTCCATTTTCAAATACTTATGAAGTTAGTATGCAGGAGCAGACTATGACTTACACT
GTTTCATCAAGTGTCTATACTTCGTCAGCTGCTTCATCGACAAAATCACTTTTGTAA

>Solyc10g047810.1.1   GenBank ID: KT232162
MASFAYLVCLLLIIPFVNSLSFNFDSFNTNDQNLTYEADAYPANSVIQLTKNQRDSASNDSIGR
VTYSEALYLWDKASRNLTDFTTHFSFGINSQGRNNYADGLAFFLAPAGSRIPDNSAVGGSLG
LAVSGQQNTSRNHSFVAVEFDTFKNFYDPKGDHVGVDINSMVSVVNVTWFSSIPNGKRTDA
WITYNSTSKNLSVVFTGFQQQGNTTLTVLQNLSYNIDLREYLPEWVTFGFSGATGTLFALQTI
YSWNFTSSLKHNDNITDPDVPLPRPVPEDTSSKNKSGLVIGLISGGCVLVALSVIILFAFRRKR
KVREDEDEDEDDDIIDGSMTNEFERSTGPKKFLYSELVRCTNNFSREEMLGQGGFGGVYKG
YLSESNSYIAVKRVSRESKQGIKEYASEVRIISRLRHKHLVQLIGWCHQKRELLLVYEFMPNG
SLDYHLFKGRSHLTWPIRFKIAQGLASALLYLHEEWEQIVVHRDIKSSNIMLDSNFNAKLGDF
GLARLVDHDKESQTTVLAGTMGYMAPECVTTGKASKETDVYSFGVVVLEIGCGRKSIENKAE
EHQVNIIQWVWRLYGMGNLREAVDPRLSSEFNEEEMEHLLIVGLWCSHPDNNCRPSIRQAI
QVLNLEAPLPILPPNMPVPTYCSRSQYGSTTSFTSPYDSNAPWNSEIQSSVTRDYTGSSNNT
AASASSSPSASLLYTC

>Solyc10g047810.1.1   GenBank ID: KT232162
ATGGCTTCTTTTGCATACCTTGTTTGTCTTTTATTAATAATCCCCTTTGTCAATTCATTGTC
CTTCAATTTTGATAGTTTTAACACAAATGATCAGAATTTAACATATGAAGCAGATGCATAT
CCAGCAAACAGTGTAATTCAGCTCACCAAAAACCAGCGAGATAGTGCTTCAAATGATAGC
ATAGGCAGAGTCACATATTCAGAAGCTCTCTATCTTTGGGACAAGGCCTCTAGGAATCTC
ACTGATTTCACAACGCATTTCTCCTTTGGGATCAACTCACAGGGCAGAAATAATTACGCT
GACGGTCTTGCCTTCTTCCTTGCTCCTGCAGGTTCAAGAATTCCTGATAACTCAGCCGTA
GGAGGCAGCCTTGGCCTTGCAGTTAGTGGTCAACAAAATACATCGAGAAATCACTCTTTT
GTTGCTGTGGAGTTTGACACCTTTAAGAACTTTTATGATCCAAAGGGTGATCATGTAGGT
GTAGATATCAACTCTATGGTATCTGTTGTTAATGTGACCTGGTTTAGTAGCATTCCAAATG
GTAAGAGAACTGATGCCTGGATTACTTATAATTCAACTTCAAAAAATCTTAGTGTTGTCTT
CACCGGTTTCCAACAACAAGGTAATACTACACTCACAGTCCTGCAGAACCTATCTTACAA
TATTGATCTGAGGGAATATTTGCCAGAATGGGTCACTTTTGGCTTCTCAGGTGCAACAGG
AACTCTCTTTGCATTACAAACCATATACTCTTGGAATTTTACTTCTTCTTTAAAGCATAATG

ACAATATAACAGATCCAGATGTGCCCCTACCAAGACCTGTGCCAGAGGATACTTCAAGC
AAAAATAAGTCAGGACTAGTGATTGGATTGATTTCTGGCGGTTGTGTTTTGGTAGCACTA
TCTGTTATAATATTGTTTGCATTTAGGAGAAAGAGGAAGGTGCGAGAAGATGAAGATGAA
GATGAAGATGATGATATTATTGATGGTTCCATGACTAATGAATTTGAAAGAAGCACAGGA
CCAAAGAAGTTTTTGTACAGTGAGTTGGTCAGATGTACAAATAACTTTTCGCGGGAAGAG
ATGCTTGGCCAGGGCGGGTTCGGAGGTGTTTATAAAGGATATCTCAGTGAATCCAACTC
CTATATTGCTGTTAAGAGGGTTTCAAGGGAGTCAAAGCAAGGAATAAAAGAGTATGCATC
AGAAGTCAGGATCATCAGCCGGTTAAGACATAAACATTTGGTGCAACTCATTGGTTGGTG
CCATCAAAAAAGAGAACTTCTACTTGTCTACGAGTTTATGCCTAATGGAAGCTTAGATTAC
CATCTTTTCAAAGGAAGAAGCCATTTGACATGGCCAATAAGATTCAAGATTGCTCAAGGT
TTGGCCTCAGCGTTGCTTTATCTACACGAAGAATGGGAACAGATTGTGGTTCATAGGGA
CATAAAGTCCAGCAATATTATGTTGGATTCCAATTTTAATGCTAAACTTGGGGATTTTGGT
TTAGCTAGGCTAGTTGACCATGATAAGGAATCCCAAACAACAGTTTTGGCAGGCACGAT
GGGTTACATGGCTCCTGAATGTGTCACCACTGGCAAAGCTAGCAAGGAAACAGATGTCT
ATAGCTTTGGTGTTGTCGTGTTAGAAATAGGTTGTGGAAGGAAATCTATTGAAAATAAAG
CTGAGGAGCATCAAGTAAATATTATCCAATGGGTTTGGAGACTTTATGGGATGGGAAATC
TTCGTGAAGCAGTTGATCCTAGACTCTCATCAGAGTTCAATGAAGAGGAGATGGAGCAC
TTGCTAATTGTTGGCTTATGGTGTTCTCATCCAGATAATAATTGCAGACCTTCTATAAGGC
AAGCAATTCAGGTGCTTAATTTAGAAGCTCCGTTGCCCATACTCCCTCCAAACATGCCTG
TACCGACATATTGCAGTCGGTCACAATATGGATCAACTACTTCTTTTACATCACCGTATGA
CTCCAACGCTCCTTGGAATTCTGAAATACAGTCTTCAGTGACTAGAGACTACACTGGTTC
TTCAAATAACACAGCAGCTTCTGCATCATCTTCGCCTTCAGCATCACTTCTGTACACATGT
TGA

>Solyc10g047680.1.1 GenBank ID: KT232163
MVVFASLSSLKYFLLIPLVTSLSFNFDTFTPNDHNVTYERDASPENGGETKYGDGFAFFLAPA
GSTIPYNTTRGGSLGLTSNNERLNLSINHFVAVEFDTYQNPYDPKGDHMARKLMPGLTIILYR
KILLLSSLVFNHKRIISVTVLQNLSYNLDLREYLPEWVTFVFTDGAGRRFALQRIYSWNFTSSL
DNMTDTGVTLSNIKPEDAPTKTKLRLVVGLICGGSVLVAVCTLVLFAFWRKSKEEMLGRGGF
GGVHKGYLSESTLIFHEKRELLLVYEFMPNSSLDTHIFSRKIHLEWPIRFKISQGLASALLHLHE
EWEQCVVHRDIKSTNIMLDSNYNAKLGDFGLARLVEHGKGSETTVLAGTMGYMAPECVTTG
KANKETDVYSFGIVVLEIGCGRKPIDPKAEEHQVNIVDWVWRLYEMIKSSLSIQVLNFEAPLPT
LPRNLPVPTYCSFSRHLPTISVSSKHETNFTETALAQIQHSVNKNNSHSSINKTSSSSTSVLYP
SQCCAVITLNFVFR

>Solyc10g047680.1.1 GenBank ID: KT232163
ATGGTTGTTTTTGCCTCTTTATCTTCTCTCAAATACTTCCTTCTTATCCCTTTAGTCACTTC
ACTCTCCTTCAATTTTGATACTTTTACACCCAATGATCACAATGTTACCTATGAGAGAGAT
GCTTCTCCAGAAAATGGTGCAATTCTAATCACCAAAAACCTCCTCAATCATGATTTAATGC
TAGTATTGGTCGAGCTATATATTCAAAACCACTCCATCTCTGGGACAAGGCCTCCCAAAA
TCTCACTGATTTCACTACACATTTCTCCTTTAGCATTAATTCACAGGGAGAAACTAAATAT
GGTGATGGTTTTGCCTTCTTCCTCGCCCCTGCAGGTTCAACCATTCCTTATAACACAACA
AGAGGTGGAAGTCTTGGCCTTACAAGTAATAATGAACGACTAAATTTATCGATTAATCATT
TTGTTGCGGTAGAGTTTGATACCTATCAGAACCCGTATGACCCAAAGGGTGATCATGTAG
GTATCGATATCAACTCAATGCAATCTCTTGTTAATGTGACTTGGTTTAGTAGCATTCCGAA
TGGCACGAAAACTGATGCCTGGATTAACTATAATTCTATATCGAAAAATCTTACTGTTGTC
TTCACTGGTTTTCAACCACAAGAGAATAATATCAGTCACTGTCCTGCAGAATCTTTCTTAC
AATCTTGATCTGAGGGAATATTTGCCAGAATGGGTCACTTTTGTCTTCACTGATGGAGCA
GGAAGGAGATTTGCATTACAAAGAATCTATTCTTGGAATTTTACTTCTTCTTTAGATAATAT
GACAGATACAGGGGTGACCTTATCAAACATCAAGCCAGAGGATGCACCAACAAAAACTA
AGTTAAGACTTGTAGTTGGATTGATTTGTGGTGGTAGTGTTTTGGTTGCAGTATGTACTTT
GGTATTATTTGCTTTTTGGAGAAAGAGTAAGGTCAGAGAAAATGATTAGGTCTTTGATGG
TTCCATGACTAATGAATTTCAAATAAGTGCAGGACCAAAGAAGTTCTTGTACAGTAAGTTG
GTTAAATGTGCAAATAATTTTTCACAGGAAGAGATGCTTGGGCGGGGTGGATTCGGAGG
TGTTCATAAAGGATATCTCAGTGAATCAACTCTTATATTGCTTTTAAGAGGGTTTCAAGGG
AGTCAAAGCAAGGAATAAAAGAGTATGTATCAGAAGTCAGGATCATCAGCCGGCTAAGA
CAATAAACATTTGGTGCAACTCATTGGTTGGAGCCATGAGAAAAGAGAACTTCTACTTGT
TTATGAGTTTATGCCTAATAGTAGCTTAGATACCCATATTTTCAGTAGAAAAATTCATTTG
GAATGGCCAATAAGATTCAAGATTTCTCAAGGCTTGGCCTCAGCGTTGCTACATCTACAT
GAAGAATGGGAACAATGTGTGGTGCATAGGGATATAAAGTCTACCAATATTATGTTGGAT

TCTAATTACAATGCCAAGCTTGGGGATTTTGGGTTAGCTAGGCTAGTTGAACATGGTAAG
GGATCTGAAACAACAGTTTTAGCAGGCACAATGGGTTACATGGCTCCTGAATGTGTCAC
CACTGGTAAAGCTAACAAGGAAACAGACGTCTATAGCTTTGGTATTGTCGTGTTAGAAAT
AGGTTGTGGAAGGAAACCTATTGATCCTAAAGCAGAGGAGCATCAAGTAAACATTGTCG
ATTGGGTTTGGAGACTTTATGAGATGATAAAATCTTCTTTAAGCAGCTAATCCGAAACTAT
CATCAGAGTTCAATGAAGAGGAGATGGAGTATTTGCTAATTGTTGGCTTATGGTGTGCTC
ATCCAGATAATAATTGCAGGCCTTCTATTAGGTAAGCAGTTCAAGTGCTTAATTTTGAAGC
TCCATTACCCACCCTCCCTCGGAACTTGCCTGTGCCCACATATTGCAGCTTCTCACGCCA
TCTTCCAACTATTTCAGTTTCATCAAAACATGAAACCAACTTTACAGAAACCGCCCTTGCC
CAAATCCAGCATTCAGTAAACAAAAACAATAGTCATTCTTCAATAAACAAAACATCTTCAT
CTTCAACCTCAGTTTTGTATCCAAGTCAATGTTGCGCTGTTATAACCTTGAATTTTGTCTT
CCGTTAG

>Solyc10g047700.1.1 GenBank ID: KT232164
MDCVMDIMVDKNQPAFVPGSVFPDKHFAAGEGLGLACVDQQYSSKNHHFVAIEFDIFTNYY
DPRGDHNLSYILDMKEYLLEWVTLGFSSAIAIFYAIHTIYSWNFTSSLNYNGNITYPDIPIPCPVL
NLYVPLQSLVLNPNVPLPRPMLNPDVPLPSHVSEDTSKKKKLVRYTNNFSREKFLGQGGFG
YVCKRYMKESNSYIDVKGISRETNQEIKEYASEVRIIRLTHKHMVQLIGRCHQKRELLHVYEF
MPNGSLDYNLYKGQSHFTCTIRFKNSEGTMCCRDLECVTTSKASKETDIHSFSFVALEIAYG
RKPSDPKAEEHQINIVDWVWKLYRIENISDAVDLRLLS

>Solyc10g047700.1.1 GenBank ID: KT232164
ATGGATTGTGTAATGGATATCATGGTGGATAAAAATCAGCCAGCCTTTGTACCAGGTATA
TTATATTGGATTTTTAATCTTTCTTATTTAGCGTGAATAAAATATTTCACAAAACCTTCAAAT
GGATATATATGTAATTGTTAGTGGATGCATAGATTCTATCATCTATAGTTATCCATATGCC
TCTTGACACATCTAGTATAGCATAACAAAAGGGACAAGGCAAAAGTATACAATGACAAAA
ATCCCTAAGACATATTTTAACTAAACTAAGGACTATAACTCATTTTATTGTATTTTTGTACA
CCTTGTGGTGGCACACTGCATGACTCCACATAGTTGAGTCGTGTGGGAGATGTTTGGGT
ACCACGCAAGCCAAAAAGTATACACAAATATTTAAAAAATGGATCAAGAGGGTAATAATA
CCTTATAATTTAATGTGTCTCTGAAATTTTGCCATAATCTAGGGGATAGATGTATCATATC
CCTAAAAAACAAATTCTACATATTCTAATAGCAATATATAATTAGATTCTTACTAAAAATTA
GATTCTTGTGCAAACAGGGAGTACCCAAAGCACCTATAGTCCTTGAAGGGAGTTTTAAAA
ATAATAATCTTGATGAAAGGAGAGTTTGTTTTTTACCCATTACATGTTTTCTCAATGTAGTT
TAAAATGACTTTCTATATAAGTTATCATCAATGAAAAAATAACTAAGTGTCTCAATTGCAAG
ATTGTTTGTTTTTTCCACTTAATGCAATAAATATAAATATTTTAGACACTTGAAAATATAAAA
CATTACTTTATTATTTTGACTAGTGTAGAAACATACATTTGCCTAACAATGGTTGTTTTTGC
ATCTTTATCATACTTTGTTTTGATAATCACCTTTGTATCTCACTATCCTTCAATTTTAATAGT
TTCAATCCCAATAATGAGAATATAACATATGAAGCAGATGATTATTCAGAAAACGGTGTAA
TTTATCTCACCAAAAATAGTTCAGTCATAGGCTGAGCTACATATTCTGAAACACTTTATAT
AAGAGACGAGGACTCTGGGAATGTCACAGATTTCTCAGTGCATTTTTCCTTCAGAACCAA
TTCTCATGGAAGAAACCTATATTCTGATGGTCTTAACTCTTTCCTCTCCCCTGCAGGTTCA
GTATTTCCCGATAAACACTTTGCTGCAGGAGAGGGACTTGGCCTTGCATGTGTTGATCAA
CAATATTCATCAAAAAATCACCATTTTGTTGCTATCGAATTTGACATTTTTACCAACTATTA
CGATCCACGGGGTGATCATGTGGGTATCAATATAAACTCCATTCAACCTGTTTCTAATGT
GACCTAGTTTAGCGGAACTCCCAATGGTAGATGCCTGGATTACTTATAGTTCAATCTCAA
AAAATCTTAGTGTTGTCTTTAATGGTTTCAAACCACAAGGGAATACTATTTTCACCATTCT
GCAGAACCTATCTTACATACTTGATATGAAGGAATATTTGCTAGAGTGGGTCACTCTTGG
CTTCTCAAGTGCAATAGCAATCTTCTATGCAATACATACCATATATTCTTGGAATTTTACTT
CTTCCTTAAACTATAATGGAAACATAACTTATCCAGATATACCTATACCATGCCCTGTGCT
TAATCTATATGTACCCCTACAAAGCCTTGTGCTAAATCCAAATGTACCCCTACCAAGACCT
ATGCTAAATCCAGATGTACCCCTACCAAGCCACGTGTCAGAGGATACTTCAAAGAAAAAA
AAAGTTAGGAGTAGTGATTGGATTGATTTTTGGTTTTTGTGTTTTGATAGCACTATTTGTTT
TTATATTGTTTGCATTTTTTAAGAAATAGGAAGGTGAGAGAATATGAAGATGATGATGATA
TAATTGATGGTTCCATGACTAATGAATTTGAAGGGAGCACAAGACCAAAGAAGTTTTTGT
AAAGTGAGTTGGTTAGATATACAAATAACTTTTCGCGGGAAAAGTTTCTTGGCCAGGGTG
GGTTTGGATATGTTTGTAAAAGATATATGAAGGAATCCAACTCCTATATTGATGTTAAAGG
GATTTCAAGGGAGACAAACCAAGAAATAAAAGAGTATGCATCAGAAGTTAGGATTATTCG
GCTAACACATAAACATATGGTGCAACTCATTGGTCGGTGCCATCAAAAAAGAGAGCTTCT
ACATGTCTATGAGTTTATGCCTAATGGAAGCTTAGATTACAATCTTTACAAGGGACAAAG
CCATTTTACATGTACAATAAGATTTAAGAATTCTGAAGGCTTGGCCTAAGTGTTGTTTTAT

AAACATGAAGAATCAGAACAACTCCTATATTCTGTTGGATTCCAATTTCAATGCCAAGCTT
GGGGATTTTTTTTTACTAGGCTAGTTGACCATGATAAGGGATCTCAAACAACAGTTTTGG
CAGGCACGATGTGTTGCAGGGATCTTGAATGTGTCACCACTAGCAAAGCTAGCAAGGAA
ACTGACATCCATAGTTTTAGTTTTGTCGCATTAGAGATAGCTTATGGAAGGAAACCTAGT
GATCCCAAAGCTGAGGAGCATCAAATTAATATTGTTGATTGGGTTTGGAAACTTTACAGG
ATAGAAAATATTAGTGATGCAGTTGATCTTAGACTCTTATCATAG

>Solyc03g112310.1.1 GenBank ID: KT232165
MPPLSLLFILINFTIFPSIFAIEFLFNSFTANTTPALTLIDDARLEPPVIRLTNDSNQFSLGRAFYP
SQIPIKSASNSTSISSSFSTQFIFSVLPDDSSSPGFGIAFVLSASTSPPNALSSQYFGLFSNATV
HTVAPLLAVEFDTGRNPEFNDPDRNHIGIDLNSIESIVTQTAGYYSSDSDSFVPLNLRSGQNIH
AWIDFNGPEFEINVTIAPAGMSRPARTLLSYRNPIIANYMSAQMYMGFSASKTQWVEAQRLL
AWSFSDSGVARDINTTNLPVFQLENSGSSLSSGAIAGIVIGSVVAVLGCLFVFYWFWWRKKE
EDDVIEDWELDYWPHRFSNEELSQATKGFSKDELLGAGGFGKVYKGTLANNTEVAVKCVNH
DSRQGIKEFMAEISTIGRLQHKNLVQMRGWCRKGYELMIVYDYMPNGSLNKWIFDKPEKVM
NWVDRRRVLADVAEGLNYLHHGWEQVVVHRDIKSSNVLLDSEMRGRLGDFGLAKLYTHGG
VPNTTRVVGTLGYLAPEVVTRANPTAASDVYSFGVVVLEVACGRRPIDLGIVLEEEEVLIDWV
RQKYREGRLCEAADKRIKEQCSEEEMEAILKLGLTCCHPDPLRRPTMKEVVAVLLGENVDAT
PNELIVELAPTESNTRDRSDWSTEESEPLSAV

>Solyc03g112310.1.1 GenBank ID: KT232165
ATGCCACCATTATCACTGTTGTTCATTCTCATCAATTTTACAATCTTCCCATCCATTTTCGC
CATCGAATTTCTCTTCAACTCCTTCACTGCTAACACCACCCCTGCACTCACCCTCATCGA
CGATGCCCGCCTTGAGCCACCAGTTATCCGCCTCACAAATGACTCCAACCAATTTTCCCT
TGGCCGAGCTTTTTACCCATCTCAAATTCCTATAAAATCCGCTTCAAATTCCACTTCAATT
TCTTCCTCTTTCTCTACCCAGTTCATCTTCTCTGTACTCCCTGATGATTCCTCTAGTCCCG
GATTCGGAATTGCTTTTGTTCTCTCTGCTTCAACTTCTCCGCCTAATGCCCTCTCCAGTCA
ATACTTTGGACTTTTCTCTAACGCCACCGTTCACACGGTGGCCCCACTTCTCGCCGTCGA
GTTTGATACGGGTCGTAACCCGGAATTTAATGACCCAGATAGAAATCATATCGGGATTGA
TCTTAACAGCATTGAATCTATTGTTACCCAAACGGCTGGGTATTATAGTTCTTCTGGTAAT
GATAGTGATTCTTTTGTGCCTTTAAATTTGCGGTCTGGGCAAAATATACATGCTTGGATTG
ATTTTAATGGACCTGAATTTGAGATTAATGTTACTATAGCTCCGGCGGGTATGTCACGGC
CTGCTAGGACTTTGTTGAGTTATAGAAACCCCATAATTGCGAATTACATGTCTGCCCAGA
TGTATATGGGGTTCTCTGCTTCAAAGACTCAATGGGTTGAGGCGCAAAGACTTCTAGCTT
GGAGTTTCAGTGATTCTGGAGTTGCAAGGGATATTAACACTACGAATTTGCCGGTTTTTC
AGCTGGAAAATTCCGGGTCTTCGCTATCTTCTGGTGCTATTGCTGGAATTGTTATTGGTT
CTGTGGTTGCTGTGTTGGGTTGTTTATTTGTATTTTATTGGTTTTGGTGGCGTAAGAAGG
AAGAAGATGATGTGATTGAAGATTGGGAACTTGATTACTGGCCTCATCGATTTTCAAATG
AAGAGCTTAGCCAAGCTACAAAAGGGTTCTCTAAAGATGAGCTACTTGGAGCTGGTGGA
TTCGGTAAAGTATACAAGGGAACATTAGCTAATAACACAGAAGTGGCAGTGAAATGTGTG
AACCATGACTCAAGACAAGGAATTAAGGAATTCATGGCTGAGATATCTACTATCGGAAGG
CTTCAACACAAGAATTTAGTACAAATGAGAGGGTGGTGTAGGAAGGGGTATGAACTTATG
ATTGTGTATGATTACATGCCTAATGGGAGTTTGAATAAATGGATATTCGATAAGCCGGAG
AAGGTTATGAATTGGGTAGACCGGAGGAGGGTCCTAGCTGATGTTGCTGAGGGGTTGAA
CTATTTGCATCATGGTTGGGAACAAGTTGTTGTACATAGGGATATTAAATCTAGCAATGTT
TTGTTAGATAGCGAAATGAGAGGTAGATTGGGTGATTTTGGGCTAGCTAAGTTGTATACT
CATGGTGGTGTCCCTAATACTACTAGAGTAGTAGGTACATTAGGGTACTTGGCACCTGAA
GTGGTGACAAGGGCTAACCCAACTGCTGCTAGTGATGTTTATAGTTTTGGGGTGGTGGT
TTTGGAGGTGGCATGTGGGCGAAGGCCGATTGACTTAGGGATTGTGTTGGAGGAAGAG
GAAGTGTTGATTGATTGGGTTAGACAAAAGTACAGGGAAGGGCGATTATGTGAGGCAGC
AGATAAAAGGATCAAGGAGCAGTGTTCGGAGGAAGAAATGGAAGCTATATTGAAACTAG
GACTTACATGTTGTCACCCTGATCCTCTCCGTCGGCCTACTATGAAGGAGGTTGTTGCTG
TATTGCTTGGTGAAAATGTCGATGCAACGCCAAATGAGTTGATCGTTGAATTAGCACCTA
CTGAAAGCAACACGAGAGATAGAAGCGACTGGTCAACTGAGGAATCAGAGCCACTTTCG
GCTGTGTAG

>Solyc05g053010.1.1 GenBank ID: KT232166
MAKQITLTLFLILCILPSRTVFSDEFVYNGFNGVEASNLTVNGVAQILKNGALKLTNETSRVVG
HVFYKNPIKFKNSQYGKVSSFSTAFAFGIVPEYAKLGGHGFAFTISRSEEMKGALPSQYLGLL

SSSNVGNFSNHVFAVEFDTVQDFEFGDISDNHVGIDLNDLKSNASVNASYFSEGSLSKQKLF
LQSGKTIQAWIDYDSSRNLLNVSLSLSSTKPAFSILSFPVDLSLVFEEFMYVGFSASTGLLASS
HYIFGWSFNMNGIAQSLNLDSLPFLPKTKKDQTILIVATAVSAVVFFAFGLILALYVIWKIKKIDVI
EPWELEIGPHRFSYKELKKATRGFRDKELLGFGGFGRVYKGTLPKTNIVVAVKRIHHEAKQG
LQEFVSEIATIGRLRHRNLVQLLGWCRRRGDLLLVYDFMSNGSLDKYIYDEPRVTLTWDQRF
KVIKGVASGLLYLHEEWEQTVIHRDIKAGNVLLDSDMNGRLGDFGLAKLYEHGENPSTTRVV
GTLGYLAPELTKTGKPTTSSDVFAFGALLLEVVCGRRPIEAKALPEELILVDWVWDKWNDGVI
LEVVDSRLNGEYDEMEAIMVLKLGLMCSNNTPSKRPSTRLVVRYLEGEVTLPETLAAPGEYD
GKKGGTSGMELDDFIDSYPSSSYFEKVSTWSSAYDGEGDIDIEANSVAPLTDSGREDNR

>Solyc05g053010.1.1 GenBank ID: KT232166
ATGGCCAAACAAATTACGCTAACCCTTTTCTTGATTCTCTGTATTTTGCCGTCAAGAACAG
TTTTTTCTGATGAATTTGTGTATAATGGCTTTAATGGGGTTGAAGCTAGTAATCTTACTGT
AAATGGGGTTGCTCAGATTTTAAAGAATGGTGCTTTAAAGCTCACAAATGAAACATCTAG
AGTTGTTGGTCATGTTTTTTACAAAAATCCAATTAAATTCAAGAATTCCCAATATGGTAAA
GTTTCTTCCTTTTCTACTGCTTTTGCTTTTGGGATTGTTCCTGAATATGCTAAGTTAGGTG
GGCATGGTTTTGCTTTTACAATCTCAAGGAGTGAAGAGATGAAAGGGGCTCTTCCTAGTC
AGTATTTAGGTCTTTTAAGTTCAAGTAATGTAGGGAATTTTTCAAATCATGTATTTGCTGTT
GAGTTTGATACTGTTCAAGATTTTGAATTTGGTGATATTAGTGATAATCATGTTGGTATTG
ATTTGAATGATTTGAAGTCTAATGCTTCAGTAAATGCTAGTTACTTCTCTGAAGGAAGTTT
GAGTAAGCAGAAGCTTTTTCTTCAGAGTGGAAAGACAATTCAAGCTTGGATTGATTATGA
TTCAAGTAGAAATCTGTTGAATGTTTCACTTTCACTTTCTTCAACAAAACCAGCTTTTTCAA
TATTGTCCTTCCCTGTGGACCTTTCTCTAGTTTTTGAGGAATTTATGTATGTTGGATTTTCT
GCTTCAACTGGTTTGCTTGCTAGTTCACATTATATATTTGGTTGGAGTTTCAATATGAATG
GGATAGCACAATCTTTGAATCTTGATTCATTGCCTTTTCTCCCTAAGACTAAGAAGGATCA
AACAATCTTGATTGTAGCTACCGCGGTATCGGCTGTTGTGTTTTTTGCATTTGGACTCATT
TTAGCCTTGTATGTCATTTGGAAAATCAAGAAAATAGATGTGATTGAGCCTTGGGAACTT
GAGATAGGTCCACATAGGTTTTCTTACAAGGAGTTAAAGAAGGCTACAAGGGGTTTTAGA
GATAAAGAGTTACTCGGGTTTGGTGGATTTGGTCGAGTTTATAAAGGAACATTGCCTAAG
ACGAATATTGTTGTTGCTGTTAAGCGAATACATCATGAAGCAAAACAGGGTCTTCAAGAA
TTTGTGTCTGAAATCGCGACTATTGGTCGCCTTAGACATAGAAATTTGGTTCAGCTTTTAG
GATGGTGTCGGCGAAGAGGTGATTTGTTACTTGTTTATGACTTTATGTCTAACGGAAGCT
TAGATAAATACATTTACGATGAACCAAGAGTTACTTTAACTTGGGATCAGAGGTTCAAGG
TTATTAAAGGTGTGGCTTCTGGTCTTTTGTATTTACATGAAGAATGGGAACAAACAGTGAT
CCATAGAGATATTAAAGCAGGGAATGTGTTGTTAGATTCCGATATGAATGGACGACTTGG
AGATTTCGGACTTGCTAAGTTATATGAGCATGGAGAAAATCCTAGCACAACAAGAGTAGT
CGGTACGTTGGGTTACTTAGCTCCAGAATTAACTAAGACCGGGAAGCCCACGACAAGTT
CAGATGTTTTTGCCTTTGGTGCGTTGCTGCTAGAAGTTGTTTGTGGGAGGCGGCCGATT
GAGGCAAAGGCATTGCCTGAGGAGTTAATCCTAGTGGATTGGGTATGGGATAAATGGAA
TGACGGTGTCATTCTTGAAGTGGTTGACTCGAGATTAAATGGCGAGTATGATGAGATGG
AGGCCATAATGGTGCTTAAATTAGGACTCATGTGTTCAAATAATACACCATCTAAGAGAC
CTAGCACGAGGCTAGTGGTGAGATACTTGGAAGGGGAGGTGACACTCCCAGAGACGCT
GGCAGCTCCCGGTGAATATGATGGAAAGAAGGGTGGTACTAGCGGTATGGAGTTAGAC
GACTTCATAGATTCCTATCCGTCTTCGTCTTATTTTGAGAAAGTAAGTACATGGTCATCAG
CTTATGACGGTGAAGGCGATATTGATATTGAAGCTAATTCAGTAGCGCCATTAACGGATT
CTGGAAGAGAGGACAACAGATAG

>Solyc10g084860.1.1 GenBank ID: KT232167
MFNFLTLLCFTITLSNLASSQLDEFIFTKFNQPNQNITLSGVAKISQNGFIQLTNDTSRLMGHAF
YSSPFHFKPTTNASTFSFSTCFALAIVPEYPKLGGHGLAFTISPSKDFSTALPSQYLGLLNASD
IGNFSNHIFAVEFDTVRDFEFGDIDDNHVGININSLESNKSAAAGYFINDQNSKQDLNLKSGKV
ILVWVEYDSVTKLVNVTLSPNSLKPKIPLLSYKFDVSSIFEENMYVGFSASTGLLASSHYILGW
SFKLNGEAKLLDLDSLPSLPGSKKSHTGLITIISIIVFVFALCSILIAFYIIQRFKNADVIEPWEVEI
GPHRYSYRELKKGTRGFRESELLGRGGFGKVYKGVLRNSKGDIAVKRISHESKQGLREFVS
EIASIGRLRHRNLVQLLGWCRRRGDLLLVYEFMPNGSLDNFLFDEPKMVLKWEQRFKIIKGV
ASGLLYLHEGYEQVVVHRDVKASNVLLDGELNGKLGDFGLARLYEHGSNPSTTKVVGTLGY
LAPELPRTGRATTKTDVFAFGALLLEVVCGRRPIEHKAGPEEFVLVDLVWDKWREVLMVLKL
GLMCSNSKPLARPSIRQVVRYLEGEIGMPEAPSTQGSDDLEGFGFVESERWNSLASTSFMIK
SSSFTCLSNGDENSTNYVSFSTSPFPLLYSGECT

Solyc10g084860.1 GenBank ID: KT232167
ATGTTCAATTTTCTCACATTACTCTGCTTTACCATAACCTTATCAAATCTTGCTTCATCTCA
ACTTGATGAATTCATTTTCACAAAATTTAATCAACCAAATCAGAACATAACCTTAAGTGGA
GTAGCAAAAATAAGCCAAAATGGGTTTATTCAATTAACAAATGACACAAGTAGACTAATG
GGACATGCTTTTTACTCTTCACCTTTCCACTTCAAGCCTACTACTAATGCCTCTACCTTCT
CTTTCTCAACGTGTTTCGCGTTGGCCATAGTCCCCGAGTATCCAAAACTCGGTGGCCAT
GGCCTAGCATTCACAATTTCTCCATCAAAGGACTTTAGTACCGCTCTTCCGAGTCAGTAC
TTAGGTCTGCTCAATGCTAGTGATATTGGTAACTTCTCTAACCATATTTTTGCTGTTGAAT
TTGACACAGTACGTGATTTTGAGTTTGGTGATATTGATGATAACCATGTTGGTATTAATAT
TAACAGCTTAGAGTCAAATAAATCAGCTGCAGCTGGATATTTTATTAACGATCAAAATTCG
AAACAAGATTTGAATCTTAAGAGTGGGAAAGTCATTCTTGTTTGGGTAGAATATGATTCTG
TTACGAAGTTAGTTAATGTAACTCTTTCACCTAATTCTTTGAAACCAAAAATTCCTCTGTTA
TCTTATAAATTTGATGTCTCTTCAATTTTTGAAGAAAATATGTATGTTGGGTTCTCTGCTTC
TACTGGTTTGCTTGCTAGTTCACATTACATTTTGGGTTGGAGTTTTAAGTTAAATGGAGAA
GCAAAGTTACTTGATTTGGATTCATTACCATCACTTCCTGGATCTAAGAAGAGCCACACA
GGACTAATTACCATCATCTCGATTATTGTGTTTGTGTTCGCGTTATGTTCAATCTTGATTG
CTTTTTATATAATCCAGAGATTCAAGAATGCTGACGTGATTGAGCCGTGGGAGGTTGAGA
TTGGTCCTCATAGATATTCGTATCGTGAACTCAAGAAAGGTACTAGAGGTTTTAGGGAGA
GTGAACTACTCGGGCGAGGTGGATTTGGGAAAGTTTACAAGGGTGTTTTGAGAAATTCA
AAAGGAGACATCGCTGTGAAACGGATTTCTCATGAATCTAAACAAGGGTTGAGGGAATTT
GTGTCTGAGATTGCAAGCATTGGAAGACTTCGTCATAGGAATTTGGTTCAATTGTTAGGA
TGGTGTAGACGTCGTGGTGATTTGTTGCTTGTGTATGAGTTTATGCCTAATGGAAGTTTG
GATAACTTCTTGTTTGATGAGCCAAAAATGGTGTTGAAATGGGAACAAAGGTTCAAGATT
ATCAAAGGGGTTGCTTCTGGTTTACTTTACTTACATGAAGGCTATGAACAAGTCGTGGTA
CATCGAGATGTTAAGGCTAGTAATGTGTTACTAGATGGTGAATTAAATGGTAAGTTAGGG
GATTTTGGACTTGCTAGATTGTATGAGCATGGTTCTAATCCGTCCACGACTAAAGTTGTA
GGTACATTAGGGTACCTTGCACCAGAATTACCAAGAACAGGACGAGCCACTACAAAGAC
TGATGTGTTTGCATTTGGTGCTTTGTTACTCGAGGTGGTTTGTGGACGTAGGCCAATTGA
ACACAAAGCAGGACCCGAGGAGTTTGTACTCGTGGATTTGGTGTGGGATAAATGGAGAG
AAGGTAACATACTTGACATTGTGGACTAAAGATTGAAAGGCGAATACAATGTGATTGAAG
TTTTGATGGTGTTAAAATTGGGACTAATGTGCTCAAATAGCAAGCCATTGGCGCGACCAA
GCATTAGGCAAGTTGTACGATACTTGGAAGGTGAAATCGGGATGCCTGAGGCTCCATCA
ACACAAGGTTCTGATGATCTTGAAGGATTTGGATTCGTTGAAAGTGAACGTTGGAATTCT
TTAGCATCGACTAGTTTTATGATCAAGTCAAGTTCATTTACATGTTTGTCTAATGGAGATG
AAAATAGTACTAATTATGTTTCCTTTTCTACTTCACCATTTCCACTTCTATATAGTGGTGAA
TGCACCTAG

>Solyc09g011070.1.1 GenBank ID: KT232168
MVYSCLVLLCSFISFSILASSQQLDGFIYTRFNEPNNNITLSGAAEISQNGFIQLTNETSRLMGH
AFYSSPFQFKNSTNGSAFSFSTCFALAIVPEYPKLGGHGLAFTISQSNDFSTALPSQYLGLLN
ATDVGNFSNYILAVEFDTVQDFEFGDINDNHVGININSLRSNMSAKASYFDDDLVKQDLNLKC
GKVILAWVDYDSVTNLVNVTLSRFAKKPKLPLFSYHIDLSPFLKENMYVGFSASTGLLASSHY
VFGWSFKLNGEAKFLDLDLLPSLPGLKKKHSGVIVAISVIVVVLVLIGILVAIYLVRRFKNADVIE
SWELEVGPHRYSYEELKQATRSFKDSELLGFGGFGKVYKGVLQSSNMEIAVKRISHESKQG
LREFVSEISSIGRLRHRNLVQLVGWCRRRGDLLLVYDFMPNGSLDNFLFEKPRMLLTWEQRF
KIIKGVASGLLYLHEGYEQVVVHRDVKASNVLLDGELNGRLGDFGLARLYEHGSNPGTTRVV
GTLGYLAPELPRTGRATEKSDVFAFGALLLEVVCGRRPIDSKVGPEELVLVDMVWNKWREG
KILDVIDKRLKGEFNESEVVMVLKLGLMCSNNEASSRPSMRQVMSYLEGEADIPDAPMAPGD
YNGGFGFEENECMHSLASSRGHTSCLANGNVDGTFVSVSTAPLSCLFTDELPR

>Solyc09g011070.1.1 GenBank ID: KT232168
ATGGTTTATAGTTGTCTTGTACTACTCTGTTCATTCATTTCCTTTTCAATCCTAGCTTCATC
TCAGCAACTTGATGGCTTCATTTATACAAGATTCAATGAACCAAACAATAACATTACCTTA
AGTGGAGCTGCTGAGATTAGCCAAAATGGATTCATTCAACTAACCAATGAAACAAGTAGA
TTAATGGGGCATGCTTTCTATTCTTCACCTTTTCAGTTCAAGAACTCAACTAATGGCAGTG
CTTTTTCATTTTCAACATGTTTTGCTCTTGCTATAGTCCCTGAATATCCAAAACTTGGTGG
TCATGGACTTGCTTTTACTATTTCTCAGTCTAATGATTTCAGCACAGCTCTTCCAAGTCAG
TATCTTGGCTTACTAAATGCTACTGATGTTGGTAACTTCTCAAATTACATACTTGCTGTTG
AATTTGATACAGTACAAGATTTTGAGTTTGGGGATATTAATGATAATCATGTTGGTATAAA

CATTAATAGTTTAAGGTCTAATATGTCTGCTAAAGCATCTTACTTTGATGATGATTTGGTA
AAACAAGATCTGAATCTCAAATGTGGTAAAGTTATATTGGCATGGGTTGATTATGATTCTG
TTACAAACTTGGTTAATGTTACACTTTCAAGATTTGCTAAAAAACCAAAGTTGCCACTTTT
CTCTTATCATATAGATCTCTCTCCATTTCTCAAAGAAAATATGTATGTTGGTTTTTCTGCTT
CAACTGGTTTGCTTGCAAGTTCACATTATGTTTTTGGTTGGAGCTTTAAGTTGAATGGCG
AAGCGAAATTTCTAGACTTGGATTTACTTCCATCATTGCCTGGTCTCAAGAAGAAGCACA
GTGGCGTAATTGTAGCTATCTCGGTTATAGTTGTTGTTTTGGTTTTGATTGGTATATTAGT
TGCTATTTATTTAGTTAGGAGATTCAAGAATGCTGATGTTATAGAGTCTTGGGAGCTTGA
GGTTGGTCCTCATAGATACTCTTATGAAGAACTTAAGCAAGCTACTAGAAGTTTTAAGGA
TAGTGAGCTTCTTGGGTTTGGGGGATTTGGTAAAGTTTACAAGGGTGTTTTACAAAGTTC
GAATATGGAAATAGCTGTGAAGCGTATTTCACATGAATCTAAACAAGGCTTACGCGAATT
TGTGTCTGAAATATCTAGCATTGGAAGACTCCGCCATAGGAATTTGGTTCAATTAGTAGG
TTGGTGTAGACGTCGTGGTGACCTTTTACTTGTGTATGATTTTATGCCAAATGGAAGTTT
GGACAATTTCTTGTTCGAAAAACCTAGAATGTTGTTGACATGGGAGCAAAGGTTCAAAAT
CATCAAAGGGGTTGCTTCTGGTTTACTATACTTACATGAAGGTTATGAACAAGTTGTGGT
GCATCGAGACGTTAAGGCTAGTAATGTGCTACTAGATGGAGAGTTAAATGGCAGGCTTG
GAGATTTTGGACTAGCAAGATTATATGAGCACGGATCAAACCCGGGCACGACTAGGGTG
GTAGGCACATTGGGGTACCTTGCACCGGAATTACCAAGAACAGGACGAGCTACTGAAAA
ATCCGATGTTTTTGCCTTTGGTGCGTTGTTGCTTGAAGTGGTATGTGGACGTAGACCGAT
TGATTCAAAGGTGGGGCCAGAGGAATTAGTTCTAGTAGACATGGTGTGGAACAAATGGA
GAGAGGGGAAAATTCTTGATGTTATAGACAAGAGATTGAAAGGTGAGTTCAATGAAAGTG
AAGTTGTGATGGTGTTGAAATTAGGACTAATGTGTTCAAACAATGAGGCGTCGTCTCGAC
CTAGCATGAGACAAGTGATGAGTTACTTGGAAGGTGAAGCTGATATACCTGATGCTCCAA
TGGCTCCTGGTGATTATAATGGAGGATTTGGATTCGAAGAAAACGAATGTATGCATTCTT
TAGCATCTTCAAGAGGACATACATCATGTTTGGCTAATGGAAATGTTGATGGTACATTTGT
TTCTGTTTCTACTGCACCACTTTCATGTTTATTTACTGATGAGTTGCCTAGGTAG

>Solyc10g080510.1.1 GenBank ID: KT232169
MYYLGKISYLTNFVHLLIIFIFLVLRVDCLSFNYQNFTKQNENDFITTEHSYIEFGAIQVTGDARG
TSISNLSGRIWYSQPMNLWNRRKNRTASFNSTFVINIKPESDPWGEGLAFILTKESGYESIPD
ESYGQWLGIVNETTNGSSLNNIFAVEFDTRKSYLDDLDDNHVGIDINSINSVNQVSLMDRGVN
LSRAVDVIASVQYDGESNILKVYTFMSNETGVNERNPIISMPLDLSSYLPEDVFVGFSASTGIY
NQLNCIKAWNFTSTDIGNSNEVSLLWLWILIPVISVLVVFLGGVFVYFSWWRKKKRTQVLDQS
ENIEIQIQNSATAPQRFQLKDLKRATGNFDPKNILGRGGCGVVLKGMLDQKEVAVKRFFNDS
SQGAKDLIAEVTTIGNLHHKNLVKLIGWCYESNELLVVYEFMPNGSLEKLIFCEENGKGLSLN
WEIRYGVICGIAQALDYLHNGCQKRVLHRDIKASNIMLDSELNARLGDFGLARTVQVNGKTH
HSTKEIAGTIGYMAPESFHIGRATVETDVFAFGVLVLEVACGRKPGNRRYEENNYTNRVIEYV
WDLYKIGRIIDAIDVRLDRDFNEEQAECVLILGLACCHPNPYERPSMKTALQILTGELVLPDIPT
EKPAFVWPAKAPSSNEASSDSLQEGQLTPITVLSGR

>Solyc10g080510.1.1 GenBank ID: KT232169
ATGTATTATCTAGGCAAGATTAGTTATTTAACTAACTTTGTTCATCTTTTGATCATATTCAT
ATTTTTGGTTCTTAGAGTGGATTGTCTGAGTTTCAATTACCAAAATTTCACCAAGCAAAAC
GAAAATGATTTCATAACTACTGAGCATTCCTACATAGAATTTGGAGCTATCCAAGTAACTG
GCGATGCTCGTGGTACTTCCATCAGTAATTTGTCTGGAAGGATATGGTATTCACAGCCTA
TGAATCTATGGAACAGAAGGAAAAACAGAACAGCCTCGTTTAATTCAACGTTTGTGATCA
ATATCAAACCTGAGAGTGATCCATGGGGTGAAGGATTGGCTTTCATATTGACTAAAGAAA
GTGGTTATGAGTCAATCCCAGATGAAAGTTATGGACAATGGCTTGGGATTGTGAATGAAA
CTACTAATGGATCTTCTTTAAACAACATATTTGCTGTTGAATTCGATACAAGGAAAAGTTA
CCTTGATGATCTTGATGATAATCATGTTGGCATTGACATAAACAGCATCAACTCTGTCAAT
CAGGTCTCGTTGATGGATCGTGGTGTTAACCTTTCACGAGCTGTTGATGTTATAGCAAGT
GTCCAGTATGATGGAGAATCCAACATCTTGAAGGTCTATACATTCATGAGTAATGAAACT
GGAGTCAATGAAAGAAATCCCATCATTTCCATGCCACTTGATCTTTCTAGCTATCTCCCC
GAGGATGTTTTCGTGGGATTTTCAGCTTCAACTGGGATATATAATCAGCTTAACTGCATA
AAGGCATGGAATTTTACAAGCACAGATATTGGGAACAGTAATGAAGTTAGCTTGTTATGG
TTGTGGATCTTGATACCAGTAATATCGGTTCTGGTTGTGTTCCTTGGTGGGGTTTTCGTA
TATTTCAGCTGGTGGAGAAAAAAGAAGAGGACGCAAGTGTTAGATCAGTCGGAGAATAT
AGAGATTCAGATTCAGAATTCAGCTACTGCACCTCAGAGATTCCAGCTGAAGGACTTGAA
ACGAGCAACGGGGAACTTTGATCCGAAGAACATTCTTGGAAGAGGAGGATGTGGAGTAG
TCCTCAAAGGGATGTTAGATCAAAAAGAGGTGGCTGTGAAGAGATTCTTTAATGATTCAA

GTCAAGGGGCAAAAGATCTCATAGCAGAAGTCACAACTATAGGCAATCTCCATCATAAAA
ACCTTGTCAAATTGATAGGATGGTGCTATGAGAGCAATGAACTCCTTGTGGTCTATGAGT
TCATGCCAAATGGGAGCTTAGAAAAGTTGATATTTTGCGAAGAAAACGGAAAAGGTTTGA
GTCTGAATTGGGAAATAAGGTATGGTGTCATCTGTGGGATTGCTCAAGCACTTGATTATC
TGCACAATGGATGTCAAAAAAGAGTACTTCACAGAGACATCAAAGCCAGCAACATCATGC
TTGACTCAGAACTCAATGCTCGTCTAGGAGATTTCGGATTAGCTAGGACAGTTCAAGTGA
ATGGAAAGACTCACCATTCAACAAAAGAGATTGCTGGTACTATAGGCTATATGGCACCTG
AGAGTTTCCATATAGGTCGAGCCACAGTTGAAACAGACGTCTTTGCATTTGGAGTGCTAG
TTCTTGAAGTTGCTTGTGGTCGAAAACCTGGAAATAGACGATATGAGGAGAATAATTATA
CCAACAGAGTCATTGAATATGTATGGGACTTGTACAAGATTGGAAGGATCATTGATGCCA
TAGATGTCAGATTGGATAGAGATTTCAATGAAGAACAAGCCGAATGTGTGTTAATATTAG
GTTTGGCTTGTTGCCATCCAAATCCTTACGAAAGGCCAAGCATGAAAACTGCTTTACAGA
TTCTTACAGGGGAATTAGTTCTACCAGACATCCCAACTGAGAAACCTGCATTTGTATGGC
CAGCCAAAGCTCCATCGTCCAACGAAGCTTCTAGTGATTCTCTCCAGGAAGGCCAACTT
ACACCGATTACAGTACTCAGTGGCAGATGA

>Solyc02g068300.2.1 GenBank ID: KT232170
MGLLTPTTLTIFIMLFSLLQLKLQAQKMEHLNLKYASFDETFFDIFEVEKPATISNAALQVTPDS
ASSDFNRYNNSGRILFKQPFKLWDGDVFDNTTRVASFNSSFLVNIYRPNNETAAEGLAFLISP
DLEKPDNSQGQYLGLTNGSTDGISSNKVVAVELDTSKQSFDPDDNHIGIDVHSVRSVKVESL
TPHGIELAPIGARFYNVWVQYDGIKKVLDVYIVEQALKNGSTPPIPKTPILTYDLDLKEHVNQE
SYFGFSASTGTNYQLNCVLRWNLTVEYFPEKKNPWLKIVLGVGIPVVSLLILGAACLGYCYHK
KRIDRSQSNILGALKSLPGTPQEFQFKALKKATNNFDEKNKLGQGGYGVVYRGFLAGEENKD
IAVKWFSRESIKGQDDFLAELTIINRLRHKHLVKLLGWCHKNGKLLLVYEYMPNGSLDMHLFA
GPDKEPLSWHVRYKIVQGVASALHYLHNEYEQRVVHRDLKASNIMLDSKFNARLGDFGLAR
ALDNERTSYAEAEGVLGTMGYIAPECFHTGKATQQSDVYAFGAVLLEVVCGQRPGTKINGF
QFFVDWVWYLHRDGRILEAVDPRLGDDYVADEAKKLLLLGLACAHPIATDRPKTQAIVQIISG
SAPAPEVPPFKPAFVWPSMVPIDIDSSVMDTTSITTSHFNSGWSLDYQSRETPTYADHSLV

>Solyc02g068300.2.1 GenBank ID: KT232170
ATGGGGTTGTTAACACCAACAACACTCACCATTTTCATCATGCTCTTCTCTTTACTTCAAC
TCAAGCTTCAAGCTCAGAAAATGGAACATCTCAACTTAAAATATGCATCTTTTGATGAAAC
TTTCTTTGACATCTTTGAAGTTGAAAAGCCTGCAACAATTAGTAACGCTGCTCTTCAAGTA
ACCCCTGATTCTGCTTCTTCAGATTTCAATCGCTACAATAACTCAGGAAGAATCCTCTTTA
AGCAACCATTTAAGCTCTGGGATGGTGATGTTTTCGACAATACAACAAGGGTTGCATCTT
TCAACTCTTCTTTTCTTGTCAATATTTACAGACCAAATAATGAAACAGCTGCCGAAGGGTT
AGCATTCTTGATTTCACCAGATTTGGAGAAGCCAGATAATAGCCAGGGCCAGTATTTAGG
GTTAACAAATGGTTCTACAGATGGGATTTCTAGTAACAAAGTTGTTGCTGTTGAGCTTGAT
ACTTCTAAACAAAGCTTTGACCCTGATGATAATCATATAGGTATTGATGTTCATAGTGTTA
GATCTGTAAAGGTCGAATCTTTAACACCCCATGGTATAGAACTTGCTCCTATTGGTGCAA
GATTCTATAATGTTTGGGTACAGTATGATGGAATCAAGAAAGTTCTTGATGTTTATATTGT
TGAACAAGCACTGAAAAATGGGTCTACCCCACCTATACCAAAAACCCCCATTTTAACATA
TGATCTTGATTTGAAAGAACATGTAAATCAAGAATCATATTTTGGGTTTTCTGCATCAACT
GGGACTAACTACCAACTCAACTGCGTATTGAGATGGAACTTAACAGTTGAGTATTTCCCT
GAAAAGAAAAATCCTTGGTTGAAGATTGTTTTAGGTGTTGGGATTCCTGTAGTGTCTCTTT
TGATTCTTGGCGCGGCGTGTTTAGGGTATTGTTATCATAAGAAAAGGATTGATAGGTCAC
AGTCTAACATATTGGGTGCACTCAAGAGTTTGCCTGGAACTCCACAAGAGTTTCAGTTCA
AGGCTTTGAAGAAAGCTACAAACAATTTTGATGAAAAGAATAAGCTTGGGCAAGGAGGAT
ATGGAGTTGTTTACAGAGGATTTTTAGCTGGTGAGGAGAACAAAGATATTGCTGTCAAAT
GGTTTTCTAGGGAAAGCATCAAAGGGCAAGATGATTTCTTGGCTGAGCTTACTATTATCA
ATCGTCTTCGACATAAACATCTTGTCAAATTGCTTGGTAAGTGTTCTTAGTTTTTCTTCAAA
CATAGATTTTGTGCATTTGTTTGAGTAGAGTATTAGAGTTATATTGTGCATGTTTTGTTTAT
GATCTTTAATAACGTTAGGGAAATGGTACTGATAACTGATAAAGTTGATGTCATGTGATCC
AAAGTTATTAAGACTCTTGTGATCCAATCTTTCTCAGTATAACTACAAGTTTTGAGTCTCA
AATAAGGCCTTATCTCCAATATTTCAATTGAAAATTAAACAAATAGGGTAAAGATTAGTTTT
TTTTGAGTATGTACATGAAATATGTGAATAACCTTTAAGGCAAAGGAAGTTTCAAATGTAG
GGTAAATTTTGCTCAAAATTAGTTGCATTTTCTTGAATTCAGCAATCAGAAATAGAAATTG
TCCTCTTATGTTTTTTGATTCTCAATGAAAGAGAGAATTTATTACCAGGATTCATTGATGT
GACTTGAAATAATGGTGCCAAAAACCTTGTAAAAGTTGCTGACAATTTAAAGCCAAAGATT
GGTGGGACATCAAATTTAATACTCCTTTTGTTTCAATTTATATGGCTTTTTTTCTTCTTTCT

TTTTTTATTATAAAAAAGAATGATTTTTTACCTTTTTTATTTCAAACTCCATCTTTTCAACTTT
ATACGTAATATGTTTAAGAGACTAATATTATAGAATATTTTGTATTTGACATATTTTTTTACT
TTAAAATCATAAAAATCTAAAATTCTCTTTATATCTTAAACTTCGTGTAAATCGACATATAA
ATTGAAACGGAAGGAATTTATGTGATCTGTTTTTCAGACTCTCCAGCAGTGTTGTTGCAC
ATGCATTGGATTCTTTTTAGAAAAAAATACTTTTTTTTTAAGGATCTGATATGTATTCATCG
ACATTTTTAGAGAGTGAGCAACACTATTACAATAGACTCATTAAATGCATGAGGACATGC
CCTTATCTTTTTATGCTTTTCTTTTCCTAGTAACTACTAGTAAGTGATCCATGATCTTACAT
GCTTCTTCAACTAATTTGTGTAATTTTGGCATGGTACTAGTCTTTCTAGTCTGACATTTAG
CATGCTAGGAATTTACTAAGTTGGATCATCTTCTGACAAGCAAATTTCCTCTTTTGAAGAA
TCAAAGTTTTCTTGAACATTTATAATAACTTGGTCCTCCTTTTTGTCTTCAATCTCCTTTTC
CATTCTTAGAATTCTCAAGTCATCCCAAGGATTTGTCTAAGTTTTTGTTACCTGACTAACT
AGATTTGCGTCGCATAGGGTCTATCAAAGTGAGAAACACAATATATCAAGAGTGTCTCCA
ATTTTTTTTAATGTTTGAAGTCGAAACCTCTAGTTAATGAACCACACTCTACTACAGTAAAT
GTTTTAATAGTTGATGGTACGTAAGAGTTCAAGCCAAAAATATCAAGGAAGTTAGAAAAA
CTAATGGAATGCCTTAAAAGTTTTGACCAAGAACCAACTTGTTGCTATGGTCCCTCCAATT
TAGAAGAACAAGGCACTATCTTGAAGTGCTTCATTTTGATTTGCTTAAAAGAAGATGATCA
TCCTTTGTTGACCATGGAATAAAGCAGCCATTCGTCAAAGTTCAATACAATTTTTTTATGT
CAAAATTTTGTGAAGCTTCCTTCTTGTCCTAACATCCCTTTCATTCATTGTCAATGATTAAC
ACAAGTTTTGGATGTTAGTTGAATGATGATAAATTAAATCCAATGAAAAGGACATTAATTC
TGTATTTTAGTATACATTCATATCTTCAAACACCTAATCTTTGTGTCACCTACTCTTGTCAT
CTGGCCCTACAAAGAGATAAGGTTTGTAAAATACAACAAAAAGTTGACAATTTGGGTAGT
GTGCAACTTCTGTATCTAGTTATTTTTACTGATTAAAAAACAAACATAAGCATCTTACAAGA
TTGCATGTTATGGTGACAGGATGGTGTCACAAGAATGGAAAGCTACTGCTTGTATATGAA
TACATGCCTAATGGCAGTCTAGATATGCACCTCTTTGCGGGCCCAGATAAAGAGCCGCT
GAGCTGGCACGTCCGCTACAAGATTGTGCAAGGTGTGGCCTCAGCATTACATTATCTGC
ACAATGAGTATGAGCAGAGGGTGGTCCATCGCGACCTGAAGGCGAGTAACATCATGCTT
GACTCAAAGTTCAATGCGCGCCTTGGGGACTTTGGCCTTGCGCGAGCACTTGACAACGA
AAGGACCTCGTATGCTGAGGCAGAAGGAGTGCTTGGTACAATGGGATACATTGCACCAG
AGTGTTTCCACACAGGGAAAGCCACACAGCAATCTGATGTGTACGCGTTTGGGGCAGTG
TTATTGGAAGTTGTATGTGGCCAGAGACCTGGAACCAAGATTAATGGCTTTCAATTCTTT
GTTGATTGGGTTTGGTACTTGCACCGCGATGGCCGTATCTTGGAAGCTGTTGATCCAAG
GCTTGGAGATGACTATGTTGCTGATGAAGCAAAGAAACTTTTACTACTTGGTTTGGCTTG
TGCACATCCTATAGCTACTGACAGGCCTAAAACACAGGCAATAGTTCAGATTATATCAGG
GTCAGCACCAGCACCAGAAGTTCCACCATTCAAGCCAGCATTTGTGTGGCCATCAATGG
TGCCAATTGACATAGACTCAAGTGTTATGGACACAACATCCATTACTACTTCTCACTTCAA
TTCAGGATGGAGTCTAGACTATCAAAGCAGGGAGACCCCAACATATGCAGATCACTCTTT
GGTGTAG

>Solyc03g031980.2.1 GenBank ID: KT232171
MEFFAPKIFIFFSCLQLISQAKIITFDKQYGDPFDHTYAPLLEIKYPAQISNQALQITPDTASTAY
KTLNNSGRILLKRPFKLWVDDISRTASFNTSFLVNIYRPDNKTGAEGLAFLICPNLDLPLNSQG
QYLGLTNATTNGAPSNKIIAIELDTFKQEFDIDDNHVGIDINNVESVESKSLTPYGIELAPIGARF
YNIWIQYDGIKKILDVYIIEQMTKNGATPTRPKVPILTHNVDLKEVVNEDSYFGFSASTGHFKQ
LNCVLRWNLTVEYFQEKNNQEKVLITSVSVGVTLLIVLLILSGYFGYYYFFNEKKRGDDRSES
TILGALKSLPGMPRDFEFKELKEATNNFDEKNKLGEGGFGVVYKGYLVGEKLEIAVKWFSRE
SIKGQDDFLAELTIINRLRHKHLVKLLGWSHRHGKLLLVYEYMPNGSLDKHLFSSGQPLRWS
VRYNIVSGVASALHYLHNEYEQKVVHRDLKANNIMLDSNFNARLGDFGLARAIDNEKTSYAD
EGEGVLGTMGYIAPECFHTGKATQHSDVYAFGAVLLELICGQRPGTKVNGFQLYLDWVWFL
HRDGRILEAVDTRLGDQYVVEEAERLLLLALACSHPIANQRPKTQTIVQVISGSVPPPQVPPF
KPSFVWPSMVPMDIDSSSIVDTISITTPQYSSENNSIEYQTNSLI

>Solyc03g031980.2.1 GenBank ID: KT232171
ATGGAATTTTTCGCACCAAAAATCTTCATATTTTTCTCTTGTCTTCAACTCATATCACAAGC
CAAAATCATAACATTTGACAAACAATATGGTGATCCTTTTGACCACACATATGCTCCTTTA
CTTGAAATCAAATATCCTGCACAAATAAGCAATCAAGCACTTCAAATCACCCCTGACACA
GCTTCCACTGCTTACAAAACGTTAAATAATTCAGGCAGAATCCTACTAAAACGACCTTTCA
AATTATGGGTAGACGATATTTCAAGGACAGCGTCTTTCAACACGTCATTCCTGGTAAACA
TTTATAGACCGGACAACAAAACAGGAGCCGAAGGTCTAGCATTCTTGATATGTCCAAATT
TGGATTTGCCACTAAACAGCCAGGGTCAGTACTTGGGCCTAACGAACGCTACAACAAAT

GGTGCACCATCCAACAAAATAATCGCGATTGAGCTAGATACATTCAAACAAGAATTCGAT
ATTGATGATAATCATGTTGGGATTGATATCAACAACGTTGAATCCGTTGAATCAAAGTCTT
TAACACCATATGGAATTGAACTAGCTCCAATAGGTGCAAGATTTTACAACATATGGATACA
ATATGATGGAATCAAGAAAATACTTGATGTGTACATTATTGAACAAATGACAAAAAATGGG
GCAACGCCAACAAGGCCAAAAGTACCAATACTAACACACAATGTTGATTTGAAAGAGGTT
GTAAATGAAGATTCATACTTTGGTTTCTCAGCTTCAACAGGGCATTTCAAACAGTTGAATT
GTGTGTTGAGATGGAATTTAACAGTTGAGTATTTTCAAGAGAAGAATAATCAAGAAAAGG
TACTAATAACAAGTGTAAGTGTTGGTGTGACACTATTGATTGTTTTATTAATTTTGTCAGG
GTATTTTGGGTATTATTATTTTTTTAATGAGAAAAAAAGGGGTGATGATAGGTCAGAATCT
ACTATATTAGGTGCATTAAAGAGTTTACCTGGAATGCCTAGGGATTTTGAGTTTAAAGAAT
TGAAAGAAGCTACTAATAATTTTGATGAAAAAAATAAACTTGGTGAAGGTGGATTTGGAGT
TGTGTATAAAGGGTATTTAGTTGGTGAAAAATTGGAAATCGCTGTGAAGTGGTTTTCTAG
GGAAAGTATCAAGGGTCAAGATGATTTCTTGGCTGAGTTGACAATTATCAACCGTTTAAG
GCATAAACATCTTGTCAAATTACTTGGTAAGAATTTTTTTAACTTTTACTTTTTATGTTTTTT
GAGTATTTCTTCTTTGTTTAAGTTCGGATAAAGGATGAAAAGATCAGCAAAAACGAATATT
TCATAAATATCGAGTAATTCTGATCATTCAAATTAATTATATAAAAGAAATTAAGGCTAAAA
TTGTAACCCTACAACATGTCATCATAATTGAGAGAGGTCCACATATTCCAAAATTTGGTCC
TACAAAAAAAGTTGATTGGAACACATATACAACCCAATTTTTATTTTTGTTTTTTTGCCTTG
TCTTGTAAAGGTTTGATATCCTCAAAATAGTATTGATTAAATATATTTATAGATCATTATTAT
TGTAGAAACAATTTAGCTTATATATGCATTAATAATTTTAACTCCATTTGCATGAACTCGCT
CAAATTTTGTCTGTAATTAAGAAGGAGAGTTATAGAGGATCACTCAGAAACACCATCCTC
TATACCATAAGAGTTAGGAATAAGGTTGTATACATTCATAGATCAGTATTATTGTTGTCAT
TATTTGACTTATATGTGCTACTAAGAATTTCGACTCTGTCAGCTGTGTAATCTCGCTCACA
TTGTTGGTCTCAAGATAAATAAAGAATGAGAGTTGTCGAGGGTCAATAAAAAAAAGGGGA
TATGGATATTTACATCTTACCTTTTGTCAAACGTACCCCACTTGTAGGATTTCAGTGAATT
TGTTGTTGTAACAATTTCAAGTGATGTAAAGTAATCCTTTACCTTCTAATGGAAATGTAAC
CTAAATGTTACATGTAGTTATCATTTAGGTGATCTGGTAATGGTATAAAAGTTCTTTTACG
ATGTCAGTGTATATATATACATATATGCTAATATTATTGTTACAGGATGGAGCCATAGGCA
TGGTAAGCTACTCCTTGTATATGAATACATGCCAAATGGTAGCCTGGACAAACATCTCTT
CTCCTCAGGGCAGCCACTCAGGTGGAGTGTGAGGTACAACATTGTCTCGGGAGTCGCG
TCAGCTCTTCACTATCTTCACAACGAATACGAGCAGAAGGTAGTCCATCGCGATCTCAAG
GCAAACAACATCATGCTGGACTCAAACTTCAATGCGCGTTTAGGTGACTTTGGATTAGCA
CGAGCCATTGACAACGAAAAGACCTCCTATGCTGATGAAGGTGAAGGTGTACTTGGCAC
GATGGGATACATCGCGCCAGAATGCTTCCACACAGGAAAAGCAACTCAACATTCTGATG
TCTATGCATTCGGAGCTGTGTTGTTGGAGCTAATATGTGGCCAAAGACCTGGAACCAAA
GTGAATGGCTTTCAACTCTATCTTGATTGGGTTTGGTTCTTGCATCGCGATGGGAGAATC
CTTGAAGCTGTTGACACGAGGCTCGGGGATCAATACGTGGTTGAAGAAGCAGAGAGATT
GTTACTACTTGCTCTGGCTTGCTCCCATCCAATTGCCAATCAAAGACCTAAAACACAAAC
GATAGTTCAAGTTATATCAGGATCAGTACCACCACCACAAGTACCACCATTTAAACCATC
ATTTGTTTGGCCTTCTATGGTTCCAATGGACATAGATTCATCCAGCATCGTCGATACCATA
TCCATCACAACTCCTCAGTACAGTTCAGAGAACAACAGTATTGAATATCAAACCAATAGC
TTGATTTAG

>Solyc04g071000.1.1 GenBank ID: KT232172
MRFCLQNLIRVTLFFIFTINSPSLSVPFLPINNVTLYGDASFTAKSITLTQDRNCSSSSTPPISGI
GRAFYTYPVRFLDSLTNNTASFLCTFSFTILPTPSCPFGDGMAFLVTSDVDSLSISDGYMGLP
NPDSEDSFLAVEFNANDNRIGVDTKEIRSLASASVDSAGIDLKSGKEMAGRIEYKDSEKIIRV
WIGYELQIRPPNPVLSTRIDVSNQLNEFMRIGFTAKGSAVYSISRWRFRTFGLISSPISSSWDQ
SDEGNCLMCFPEEEIGGHISDSHHSSSTSSKSLLKLTYGGLAAIVTLVGCALSVVFVLALRRK
KRDRVGENKERQMCRLQGNRVPQRLSLSEIKSATECFNHERIIGEGASAVVYEGEIPSRGSV
AVKRFVHGSRLGPSHIPFNTEFASMVGCLRHKNLIQLQGWCCERNELVLVYEFMPNGSLDKI
LHERSHLTKFLTWERRLNIVIGVSSALMYLHDECENHIIHRDVKSCNIMLDAEFNAKLGDFGLA
EVFDNSKTRDATVPAGTMGYFAPEYVYTGIPTVKTDVYSFGVVVLEVASGRKPIDEGGGLIT
DWVWDMWEKGRITEAADPKLMGRFQKNEMDRMLIVGLSCVHPDHEKRPRMRDVFRMLKD
EAPLLILPPMKPTVRLQSILPESCEEIMNWAARMEDTPWSTPRTHFSKN

>Solyc04g071000.1.1 GenBank ID: KT232172
ATGCGTTTTTGTTTGCAAAATCTCATAAGGGTCACTCTGTTCTTCATCTTCACCATAAATT
CACCTTCCCTTTCAGTTCCTTTCTTGCCTATAAACAATGTAACTCTTTATGGTGACGCTTC
ATTCACCGCAAAATCGATCACCCTTACCCAAGACCGCAACTGTTCGTCATCATCAACACC

TCCCATTTCCGGCATTGGAAGAGCTTTCTACACATACCCAGTTCGTTTTCTTGATTCTTTA
ACCAATAACACTGCTTCTTTCTTGTGTACTTTCTCTTTTACTATACTTCCAACCCCTTCTTG
CCCTTTTGGTGATGGCATGGCGTTTTTGGTCACTTCTGATGTTGATTCTTTGAGCATCTCT
GATGGGTACATGGGTCTTCCGAATCCCGACTCGGAAGATTCGTTCTTGGCTGTGGAATT
CAACGCCAATGATAACCGTATTGGTGTTGATACTAAAGAAATTAGGTCATTGGCTTCTGC
TAGTGTTGATTCAGCTGGGATTGATTTGAAAAGTGGGAAAGAAATGGCGGGTCGGATTG
AGTATAAAGATTCAGAGAAGATAATCAGAGTTTGGATTGGGTATGAACTGCAAATTAGGC
CTCCTAATCCTGTTCTTTCTACCAGAATTGATGTTTCCAATCAGTTGAATGAGTTTATGAG
GATTGGTTTCACTGCTAAAGGGTCTGCAGTTTATAGCATTAGTCGTTGGCGATTTAGAAC
GTTTGGATTGATTTCGTCTCCAATATCATCGTCTTGGGATCAATCCGATGAAGGAAACTG
TTTGATGTGTTTCCCTGAGGAGGAAATTGGTGGCATATTTCTGATTCTCATCATAGCAG
TAGTACTAGTAGTAAAAGCTTACTGAAATTGACTTATGGAGGGTTAGCTGCAATTGTCAC
ACTTGTTGGTTGTGCTTTGTCTGTTGTGTTTGTTCTTGCGTTGAGAAGGAAAAAACGCGA
TAGAGTAGGGGAGAACAAGGAACGGCAAATGTGTAGATTACAAGGAAATAGAGTGCCTC
AAAGATTGTCATTATCTGAAATAAAATCAGCAACAGAATGTTTTAATCATGAAAGGATAAT
TGGAGAAGGAGCATCTGCTGTTGTGTATGAAGGGGAAATTCCTTCTAGGGGATCTGTGG
CTGTTAAGAGATTTGTCCATGGGAGTAGATTGGGTCCTTCACATATTCCTTTTAATACTGA
ATTTGCTTCTATGGTTGGCTGTTTAAGACACAAGAATTTGATTCAGCTTCAAGGGTGGTG
TTGTGAGAGGAATGAATTGGTGTTAGTGTATGAATTCATGCCTAATGGTAGCCTTGACAA
AATCCTCCACGAGCGATCGCATTTAACTAAGTTTCTGACATGGGAGAGAAGACTGAACAT
AGTTATTGGTGTGTCATCTGCACTTATGTATCTTCATGATGAGTGTGAGAATCATATAATT
CACAGAGATGTGAAGAGTTGCAATATAATGCTTGATGCTGAGTTTAATGCAAAGCTTGGA
GATTTCGGTTTAGCAGAAGTGTTTGATAATTCTAAGACAAGGGATGCTACTGTACCAGCT
GGAACAATGGGATATTTTGCACCTGAGTATGTGTATACTGGTATTCCAACTGTTAAAACA
GATGTGTATAGCTTTGGTGTTGTAGTACTGGAAGTGGCATCAGGTAGAAAGCCTATCGA
CGAAGGTGGTGGTTTGATTACTGATTGGGTGTGGGACATGTGGGAGAAAGGGAGGATA
ACTGAGGCTGCTGATCCTAAACTAATGGGGCGGTTTCAGAAGAACGAGATGGATAGAAT
GCTGATCGTGGGACTTTCTTGTGTGCATCCCGATCATGAGAAGAGACCGCGAATGAGAG
ATGTTTTCCGTATGCTTAAAGATGAAGCTCCACTTCTCATTTTACCTCCAATGAAGCCCAC
TGTGAGACTTCAATCTATTTTACCAGAGAGCTGTGAAGAAATCATGAATTGGGCTGCAAG
AATGGAGGATACACCATGGTCTACTCCAAGAACTCATTTTAGCAAGAACTAG

>Solyc07g065610.1.1 GenBank ID: KT232173
MFVSIFRFLIFFSFLSLVSSQNCSFDLQSFTLRNFTLLGDSYLRNGVVGLTRDLQVPSSSSGS
LIYNNPISFFDPETKKTASFSTRFAFSVTNINPSSFGDGLAFFLSPDNQTLGSPGGFLGLVNSS
QLTKNKFVAVEFDTKQDLHFNDPDDNHVGLDIDSLISIKTANLRLAGVDLKSRNLISCWIDYKS
QEKKLMVFLSYYSLKPKKPILIVDIDLSDYIKEFMYVGFAASTEGSTELHSIENWSFRTYGFGP
VRPPHNVSDNTVIVKPPIIQDSGGHKHHNKSFGLGFGIGGPAFFCAVLVAFGWISVKKWRGL
NTEKNLKAELVTGPRQFSYKELRSATRGFHSSRIIGNGAFGTVYKAFFMESSSIAAVKRSKHS
HESKTEFGAELSIIACLRHKNLVQLQGWCIEKGELLLVYDYMPNGSLDKVLYQESEHGNPLK
WPYRYNIAVGLASVLTYLHQECEQQVIHRDIKASNIMLDASYNARLGDFGLARLMDHDKSPV
STLTAGTMGYLAPEYLQYGKATEKTDVFSYGVVILEVACGRRPIEGEGTGHEMVNLVDWVW
RLYSEGRIIDAADKRLNEDFKEEEMKKLLLVGLSCANPDSTERPCMRRVFQILNNEAEPIFVP
KVKPTLTFSTSIPFNIDDIFSDSEGSEAPEHELEIRID

>Solyc07g065610.1.1 GenBank ID: KT232173
ATGTTTGTTTCCATTTTCAGATTTTTGATTTTCTTCAGTTTCTTGTCATTAGTATCATCACAA
AATTGCAGCTTTGATTTGCAATCTTTTACACTTCGCAATTTCACTCTTCTTGGTGATTCTTA
TCTACGTAATGGTGTTGTTGGTCTAACTAGAGATCTTCAAGTTCCATCTTCAAGCTCTGGT
TCCCTCATTTACAATAACCCCATCTCGTTTTTCGATCCAGAAACCAAGAAAACGGCGTCT
TTTTCAACAAGATTTGCTTTTTCTGTTACTAACATCAACCCCTCTTCATTTGGTGATGGATT
GGCTTTTTTTCTGTCACCTGATAATCAGACATTGGGTAGTCCAGGTGGGTTTTTGGGTTT
GGTGAATTCTTCACAGTTAACTAAGAATAAGTTTGTTGCTGTTGAATTTGATACTAAACAA
GATTTGCATTTTAATGATCCTGATGATAATCATGTTGGTCTTGATATTGATAGTCTTATTTC
AATAAAGACTGCAAATTTGAGGTTAGCTGGTGTAGATTTGAAAAGTAGGAATTTGATTAGT
TGTTGGATTGATTACAAGAGTCAGGAGAAGAAGTTGATGGTTTTCTTGAGTTACTATAGTT
TAAAGCCTAAAAAACCAATCTTGATTGTTGATATTGACTTGTCTGATTATATAAAAGAGTTT
ATGTATGTGGGGTTTGCTGCTTCTACTGAGGGGAGTACTGAATTGCATAGTATTGAGAAT
TGGAGTTTTCGAACTTATGGATTTGGCCCTGTGAGGCCTCCTCACAATGTTTCTGATAAT
ACCGTGATTGTAAAGCCTCCGATTATTCAAGATTCTGGTGGCCATAAACATCATAATAAG

AGTTTTGGGTTGGGTTTTGGAATTGGTGGTCCAGCTTTCTTTTGTGCTGTTCTTGTAGCC
TTTGGTTGGATTTCTGTTAAGAAATGGAGGGGTCTTAACACAGAGAAGAATTTGAAAGCC
GAGCTTGTTACTGGACCGAGGCAGTTCAGTTACAAGGAGCTGAGGTCAGCTACAAGAGG
ATTTCATTCCAGCAGGATTATAGGAAATGGGGCTTTTGGTACTGTTTACAAGGCGTTTTT
CATGGAATCGAGCTCTATTGCTGCAGTGAAAAGATCTAAGCACAGCCATGAAAGTAAGA
CTGAGTTTGGCGCTGAGTTGTCGATCATAGCATGTTTAAGGCACAAAAATTTAGTTCAGC
TGCAAGGGTGGTGTATTGAGAAGGGAGAGTTACTTCTTGTTTATGACTATATGCCTAATG
GGAGTCTTGATAAGGTGCTATACCAGGAATCCGAGCATGGGAATCCGCTGAAATGGCCT
TACAGGTACAATATAGCAGTTGGTTTGGCGTCTGTTCTGACTTATTTGCATCAAGAATGT
GAGCAGCAGGTAATTCACAGAGACATAAAAGCAAGCAATATTATGCTTGATGCAAGCTAC
AATGCGAGGCTTGGCGATTTTGGGCTGGCAAGACTTATGGATCATGACAAGAGTCCAGT
CTCAACACTTACTGCTGGAACAATGGGATACCTTGCTCCTGAGTACCTTCAATACGGAAA
AGCAACCGAGAAGACTGATGTTTTCAGCTACGGTGTGGTTATACTAGAGGTGGCTTGCG
GGAGGAGACCAATTGAAGGAGAAGGTACTGGTCATGAAATGGTGAATTTGGTTGATTGG
GTTTGGAGACTGTACTCTGAAGGTAGGATTATCGATGCAGCAGACAAGAGGCTTAACGA
AGACTTCAAAGAGGAAGAGATGAAAAAGTTGCTACTTGTTGGACTGAGCTGTGCAAATCC
TGATAGCACAGAAAGGCCTTGTATGAGGAGAGTATTTCAGATACTCAACAATGAGGCGG
AACCTATCTTTGTTCCGAAAGTGAAACCAACTCTAACTTTCTCCACTAGCATCCCGTTCAA
CATTGATGACATTTTCTCAGACAGTGAAGGGAGTGAGGCACCAGAACATGAGCTCGAAA
TCAGAATAGATTGA

>Solyc03g080060.1.1
MDGPSINVTKHISFRDFSSINPRLKQDLTLVGSVIVSDEKKSVQIPDPEREGDDLKHLVGRAIY
SSPIRLFDPQTQTPASFETTFSFQFEVKSYEASDQGKYVGGSGLTFIIVPDELTVGRAGPWLG
MLNDLCDEDYKTVAIEFDTRKNPEFGDPNDNHLGINLGSIVSTAAINASDAGVQLNDGSVHR
VWISYDGRKRFVEIRLAPDGRGYPSKPVYSGLLDLSPYLNEYMFVGFSAATGNHTQIHNILS
WNFTSISQASLRIPSTETCQNKIMLQNSTQSENDHRKTPNSFFIFLAVVILLVIVLINLYFSSYKR
DSNSDEAFPLPEKKQRPRPPNKARRFTIAEISIATRNFSELQILGSDEKSITYKATILNGCNVVV
KRFLTQFFNTHGFEKRQFHKEIKAITRIRHPNLVPIRGWCYDNQETIVVYDFIPNGSLDKWLFG
VGVLPWTRRFKVLKDLADSLVYLHSKQLAHKNVKSSSVFLDVSFRAVVGDFGFVLTSAGSTR
FEAMVSQTADVFEFGVVVLEIIAGRSRKSNPGERDLLDLAWAMHEVQQKETLVDRRMGAVV
NLEQAIRALDIGLLCTLNENKGRPTMEEVVEFLNMEKPIPELPSGRPVCLFPYSSTTGLCSGY
ACTTFK

>Solyc03g080060.1.1 Corrected; GenBank ID: KT232174
MDCFFWFFFSFAILVNSYLGSSMDGPSINVTKHISFRDFSSINPRLKQDLTLVGSVIVSDEKKS
VQIPDPEREGDDLKHLVGRAIYSSPIRLFDPQTQTPASFETTFSFQFEVKSYEASDQGKYVG
GSGLTFIIVPDELTVGRAGPWLGMLNDLCDEDYKTVAIEFDTRKNPEFGDPNDNHLGINLGSI
VSTAAINASDAGVQLNDGSVHRVWISYDGRKRFVEIRLAPDGRGYPSKPVYSGLLDLSPYLN
EYMFVGFSAATGNHTQIHNILSWNFTSISQASLRIPSTETCQNKIMLQNSTQSENDHRKTPNS
FFIFLAVVILLVIVLINLYFSSYKRDSNSDEAFPLPEKKQRPRPPNKARRFTIAEISIATRNFSEL
QILGSDEKSITYKATILNGCNVVVKRFLTQFFNTHGFEKRQFHKEIKAITRIRHPNLVPIRGWCY
DNQETIVVYDFIPNGSLDKWLFGVGVLPWTRRFKVLKDLADSLVYLHSKQLAHKNVKSSSVF
LDVSFRAVVGDFGFVLTSAGSTRFEAMVSQTADVFEFGVVVLEIIAGRSRKSNPGERDLLDL
AWAMHEVQQKETLVDRRMGAVVNLEQAIRALDIGLLCTLNENKGRPTMEEVVEFLNMEKPIP
ELPSGRPVCLFPYSSTTGLCSGYACTTFK

>Solyc03g080060.1.1 Corrected; GenBank ID: KT232174
ATGGATTGTTTTTTCTGGTTTTTCTTTTCCTTTGCCATATTAGTTAATAGTTATTTAGGTTC
GTCCATGGACGGACCTTCAATTAATGTGACGAAGCATATCTCTTTTCGAGATTTCAGTTC
TATTAATCCGAGATTGAAGCAAGATCTTACACTTGTTGGTAGTGTTATCGTCTCGGATGA
AAAAAAATCCGTTCAAATTCCTGATCCTGAGCGAGAAGGTGATGATCTAAAGCATCTAGT
AGGACGAGCTATATATTCTTCACCTATCCGTTTGTTTGATCCTCAGACTCAAACACCGGC
TTCTTTTGAAACGACTTTCTCGTTTCAATTTGAAGTGAAGTCCTATTCAGGCAATGAGGCG
TCAGATCAGGGGAAATATGTAGGTGGTAGTGGTCTTACTTTTATAATCGTCCCGGATGAA
TTAACTGTTGGCCGTGCTGGTCCGTGGCTTGGGATGCTGAACGATTTGTGCGATGAGGA
TTATAAAACAGTAGCTATTGAGTTTGATACACGGAAGAACCCTGAATTTGGTGACCCAAA
TGATAATCACTTGGGCATTAATTTGGGTAGCATAGTTTCAACTGCAGCGATTAATGCTTCT
GACGCTGGAGTTCAATTGAATGACGGATCAGTTCACAGAGTTTGGATATCTTACGATGG
GCGAAAGCGATTTGTTGAAATTCGTCTTGCACCTGATGGCAGAGGATATCCTTCTAAACC

AGTTTACTCTGGTTTACTTGATCTTTCACCTTACTTGAATGAGTATATGTTTGTTGGATTTT
CAGCTGCTACTGGAAACCATACACAAATCCACAACATTTTGTCATGGAATTTCACCTCAAT
TAGTCAAGCTTCGCTTCGAATTCCTTCAACAGAGACATGCCAGAACAAAATCATGCTTCA
AAATAGCACACAATCTGAAAATGATCATCGGAAAACGCCTAATAGTTTCTTTATTTTCCTT
GCTGTTGTCATTCTTCTGGTAATCGTTCTTATTAACCTCTATTTCAGTAGCTACAAGCGAG
ATAGCAATTCTGACGAAGCATTCCCTCTGCCTGAGAAAAAGCAGAGACCACGGCCACCA
AACAAGGCACGCCGCTTCACAATAGCTGAGATCTCCATCGCAACAAGGAATTTCAGCGA
GTTACAAATATTAGGCAGTGATGAGAAGAGCATTACGTATAAGGCCACGATACTAAACGG
GTGCAACGTTGTTGTAAAACGATTTTTAACTCAATTTTTCAACACACACGGGTTTGAAAAG
CGTCAATTTCACAAGGAAATCAAGGCTATCACCAGAATTCGTCACCCGAATTTGGTCCCG
ATTAGAGGTTGGTGCTATGACAACCAAGAAACAATAGTTGTGTACGATTTCATCCCAAAT
GGAAGCCTCGATAAGTGGCTGTTTGGCGTTGGCGTCTTGCCTTGGACGAGGCGTTTTAA
GGTCCTTAAAGATTTAGCAGATTCTCTTGTCTACCTTCATTCAAAGCAACTCGCTCACAAA
AACGTTAAAAGTAGCAGTGTGTTTCTTGACGTGAGCTTCAGAGCAGTAGTTGGTGATTTC
GGGTTTGTGCTTACTTCAGCCGGGTCAACCCGGTTTGAGGCCATGGTGAGTCAGACAGC
TGATGTGTTCGAGTTCGGAGTAGTCGTGCTTGAGATCATTGCGGGTCGGAGCAGGAAGT
CCAACCCGGGAGAACGGGACTTATTGGATCTTGCATGGGCAATGCATGAGGTACAACAG
AAGGAAACTCTAGTGGATCGTAGAATGGGCGCGGTCGTGAACCTGGAGCAGGCGATTC
GGGCATTGGATATCGGGTTGCTCTGTACGTTGAACGAGAACAAAGGAAGGCCTACTATG
GAAGAAGTGGTGGAGTTCCTAAACATGGAGAAACCGATACCAGAGTTGCCATCGGGTCG
ACCCGTTTGTTTGTTCCCGTACAGCAGCACCACAGGCTTATGCAGCGGGTACGCTTGTA
CAACATTCAAATGA
