# Supplementary material for: L-type lectin receptor kinases in Nicotiana benthamiana and tomato and their role in Phytophthora resistance
Source: J Exp Bot. 2015 Aug 5;66(21):6731–43. doi: 10.1093/jxb/erv379 (PMC4623685; doi:10.1093/jxb/erv379)
Supplement: Supplementary Data [file supp_erv379_Supplementary_File_S2.pdf]

Supplementary File S2. DNA sequences of the fragments used for TRV-mediated silencing in *N. benthamiana* and tomato.

>TRV:*NbVIII.2*/TRV:*SlVIII.2*
gaattcggcattatttgaatcaagaatgattctaccttggctacataggaggaaaattttgctaggtgttgcttcagccttggcatatttacatcaaga
atgtgaaaatcaggtgattcatagggatataaagagtagtaacattatgttagatgaagggttcaatgcaagattaggtgattttggattagcaag
acaagttgaacatgacaagtcccccgatgcaacggtagcagccgggacaatgggctacttggctcctgaatacttgttaaccggaagagcaa
ccgaaaaaactgatgtttttagctatggagcagttgttcttgaagtggcaagtggaaggaggccaattgagagggaaacaacaagagttgaga
aagttggagtgaatagtaacttagttcccggg

>TRV:*NbIX*
gaattccgattcaagttttaatgtcaagctcggtgattttggcttagctagactaatggaccatgaattaggtcctcagactacagggttggctggaa
ctttaggttatttggctcctgaatacataaaaacaggccgagcaagtaaagagtcagacgtatacagcttcggaatagttgcacaagaaattgc
aaccggaagaaaatcagttgatccggggacggggaaatctgatgcagtgttagtggagtatgtttgggagctttataaaggacaacttctttctg
ctgttgatgagaaattaaacctggattttgacgcgaaacaagtagagcgattgatggctaccaggttatggtgtgctcatccggagagcaatctg
aggccatctataagacaagcaattcccggg

>TRV:*SlIX.1*
ggatccgacatggattacttacaatgctacaacaaataatcttagtgtcatttggaactatggaacaggtccgaattcgagcatgttttacataata
aacctcagagatgttctgcctccgtgggtcacaattggattctctgctgcaacaggactaaatgtcgagagacatacacttgaatcgtgggaattc
agctcgagtcttgttataacagagttaggtggaaatgatcgcgaaaagattggacttattgcagggctaacaacattaggtgggattttgttagtga
gcgcgattttggctttgatagttttgaggaaacgtagacgaaaggtgaagggaaatccagagacaataagcttaacatctttcaatgatgatcttg
aaaagggggcaggaccaagacccggg

>TRV:*NbX*
gaattcatggaagtctgaataaatggatatttgataagccagagaaggttatgaattggctagacaggaggaaggtcctaactgatgttgctgag
ggtttaaactatttacatcatggttgggaacaagtggttgtacatagggatattaaatctagcaatgtcttgttagattgtgaaatgagagggagact
gggagatttcgggctagcaaagttatacactcacggtggtgtaccaaatacaactagggtagtaggtacattagggtacttggcacctgaagttg
tgacaagggctacaccaactgcagctagtgacccggg

>TRV:*NbXIII*
gaattcttgtatatggtggcttagctgcagttgttacacttgttgttggtattctggctattgtgtctgtttttgtgttaaggagaaaaaaaagggatggta
gtagaggggaaaatgaaggtcaaatgtgtagattccaaggaaatagagtgcctcaaagattgtcattatctgaaataaaatcagccacagaa
ggatttaataatgaaaggataattggtgaaggaggatctgctgttgtatatgaaggagatattccttctaagggaactgttgctattaagagatttgtt
caagggactagattaggtccttcacatattccatttaacactgaatttgcttctatggttggctgcttaagacacaagaatttgattcagttccaagga
tggtgttgtgagagcccggg

>TRV:*NbXIV*

gaattcatttggagatggtttggcttttttcctttcacctgataatcagactttaggtagtccaggtggtttcttgggtctggttaattcatcccaattgacta
agaacaagtttgttgctgttgagttcgacacttggcaagatttgcactttaatgatcctgatgataaccatgtaggtcttgatatagatagccttatctc
aatcaagactgctaatgtaaagttggctggtgttgatctcaaaagtaggaatttgattacttcttggattgattacaagagtgaggaaaagcagttgt
ttgtgttcttgagttactcaagttccaagcctaaaaaaccactcttgactgtggatattgacctgtctgattatctaaaggagtttatgtacgtggggttt
gctcccggg

>TRV:*NbXVII*

gaattctaattccttttgcgacttcgctgtccttcaatttcgatagttttagacccagcgatcaaaatgtaacatacgagagagatgcttatccagcaa
atggtgcaattcaacttaccacagaccttatcaatcgtgatataaacgcaactataggtcgagccacatattccaagcttctgcatctttgggaca
aggcctcaggaaatgtcacagatttcagtactcacttctcctttagcatcaattcacaaggcagaacaagatatggtgatggtcttgccttcctgag
aacacaaccagaggtggcagtcttggccatacaagtaatactcaacgactgaatacatcggccaatcattttgtagctgtggagtttgatacgta
ccagaacgtccagtatgatcccggg

>TRV:*NbXVIII*

gaattcctcagcatcaactggacaattgtttcagaaaaacaatgtcaagtcttgggatttcaattcaagtatgagttttgatccaaacaaagttcctg
aacatgctgaaccgccgagtgcaaatcctcctattactcaagttactcctccttctaaccaagaaatcagcaccagaaaaaaaggaaataagg
gactagtcgttggatcgagcataggtttgcctattctggttatcgtattgattactggtagctgctttttatggaagaaaaagagtaaaaggaatgata
aagaccatgtttttattgatctcagtatggacaatgaatttcaaaagggtactggccctaagaagttttcctatggtgaattagctcgtgcaacgaac
aattttgctgaggtacagcccggg
