# Supplementary material for: L-type lectin receptor kinases in Nicotiana benthamiana and tomato and their role in Phytophthora resistance
Source: J Exp Bot. 2015 Aug 5;66(21):6731–43. doi: 10.1093/jxb/erv379 (PMC4623685; doi:10.1093/jxb/erv379)
Supplement: Supplementary Data [file supp_erv379_Supplementary_Table_S1.pdf]

Supplementary Table S1. Primers used in this study.

Primers	5'-3'	Used to
NbS00026192g0010.1-F	TCTCCCCTTAGTCTGTCAGTT	Amplify *NbS00026192g0010.1*
NbS00026192g0010.1-R	ACAATCCACCGAACAAAACCA	
NbLecRK-VIII.2-RT-F	CTGACCCTATGGTTAGACCAACAATG	Check the silencing efficiency of TRV:*NbVIII.2*
NbLecRK-VIII.2-RT-R	GAAGGTCTAGTTCTTGGGACAATTGG	
NbLecRK-IX-RT-F	TGATCTTGAAAAGGGAACAGGACCG	Check the silencing efficiency of TRV:*NbIX*
NbLecRK-IX-RT-R	TTGTAAACTTCTCCAAATCCTCCTTCG	
NbLecRK-X-RT-F	GTTCTCTAAGGATGAGCTACTTGGG	Check the silencing efficiency of TRV:*NbX*
NbLecRK-X-RT-R	GAGTCATGGTTCACACATTTCACTGC	
NbLecRK-XIII-RT-F	CGTCTTTTCTGGCGTTCCAACTG	Check the silencing efficiency of TRV:*NbXIII*
NbLecRK-XIII-RT-R	CCACATTCATCGATAGGCTTTCTCC	
NbLecRK-XIV-RT-F	ATGGGATACCTTGCTCCTGAGTACC	Check the silencing efficiency of TRV:*NbXIV*
NbLecRK-XIV-RT-R	CCCACAAGCCACTTCTAGTATAACCAC	
NbLecRK-XVII-RT-F	TGGTGGTTGTGTTTTGGTTTTTGTATGTG	Check the silencing efficiency of TRV:*NbXVII*
NbLecRK-XVII-RT-R	GTAAAGGAACTTCTTTGGTCCTGTGC	
NbLecRK-XVIII-RT-F	CAAATGGAGCGTTTAATGGTTCTCG	Check the silencing efficiency of TRV:*NbXVIII*
NbLecRK-XVIII-RT-R	TCAGAATTAAGAACATGGATTGCTTGC	
SlLecRK-IX-RT-F	TCAGAGGAACGAAAATTAGGTGAAGG	Check the silencing efficiency of TRV:*SlX*
SlLecRK-IX-RT-R	TCCCCTGCTTAGACCCTCTCG	
Oligo-dT	GACTCGAGTCGACATCGATTTTTTTTTTTTTTTTT	synthesize cDNA
